# Population health outcomes in South Korea 1990–2019, and projections up to 2040: a systematic analysis for the Global Burden of Disease Study 2019

**DOI:** 10.1016/S2468-2667(23)00122-6

**Published:** 2023-07-27

**Authors:** GBD 2019 South Korea BoD Collaborators, Seoyeon Park, Min Seo Kim, Dong Keon Yon, Seung Won Lee, Joseph L Ward, Susan A McLaughlin, Max L Mehlman, Ai Koyanagi, Lee Smith, Louis Jacob, Suneth Buddhika Agampodi, Maryam Beiranvand, Dong-Woo Choi, Sung Hwi Hong, Mehdi Hosseinzadeh, Cho-il Kim, Gyu Ri Kim, Jihee Kim, Kwanghyun Kim, Sungroul Kim, Doo Woong Lee, Hankil Lee, Sang-woong Lee, Yo Han Lee, Ali H Mokdad, Christopher J L Murray, Akinkunmi Paul Okekunle, Eun-Cheol Park, Navid Rabiee, Youn Ho Shin, Simon I Hay, Jae Il Shin

## Abstract

**Background:**

South Korea has one of the longest operating universal health coverage (UHC) systems. A comprehensive analysis of long-term trajectories of morbidity and mortality in the South Korean population after the inception of UHC is needed to inform health-care policy and practice.

**Methods:**

We used data from the Global Burden of Disease Study (GBD) 2019 to present estimates of cause-specific mortality, incidence, prevalence, years of life lost (YLLs), years of life lived with disability, and disability-adjusted life-years (DALYs) in South Korea from 1990 to 2019. We also examined forecasted estimates of YLLs up to 2040 to investigate likely future changes in disease burden. Finally, we evaluated GBD estimates from seven comparator countries to place disease burden in South Korea within a broader context.

**Findings:**

Age-standardised DALYs related to non-communicable diseases (NCDs) decreased by 43·6% (95% uncertainty interval [UI] 39·4–47·9) and mortality by 58·8% (55·9–60·5) from 1990 to 2019. In 2019, the ratio of male to female age-standardised rates of YLLs in South Korea was higher than the global average for 75·9% (22 of 29 diseases) of leading causes, indicating a disproportional disease burden on males in South Korea. Among risk factors, tobacco use accounted for the highest number of 2019 deaths (44 470 [95% UI 37 432–53 989]) in males and high systolic blood pressure for the highest number (21 014 [15 553–26 723]) in females. Among the top ten leading causes of YLLs forecast in South Korea in 2040, nine were NCDs, for both males and females.

**Interpretation:**

Our report shows a positive landscape of population health outcomes in South Korea following the establishment of UHC. However, due in part to the effects of population ageing driving up medical expenditures for NCDs, financial pressures and sustainability challenges associated with UHC are pressing concerns. Policy makers should work to tackle population ageing and allocate resources efficiently by prioritising interventions that address the leading causes of death and disability identified in this study.

**Funding:**

Bill & Melinda Gates Foundation.

## Introduction

Patterns of health and disease in South Korea must be viewed in the context of several unique national characteristics. First, South Korea is a highly urbanised country. Over 80% of the 52 million inhabitants reside in urban areas, with approximately 50% living in the Seoul capital area.^1^ Second, South Korea has experienced rapid economic growth,^2^ transforming the country from one of the poorest in the world at the end of the Korean War in 1953 into an economy with the tenth highest gross domestic product in the world.^2^, ^3^ Finally, during rapid industrialisation, South Korea transitioned from private voluntary health insurance to government-mandated universal health coverage (UHC).^4^ South Korea enacted the Medical Insurance Act in 1963 to establish universal health care.^4^, ^5^ To integrate all forms of health insurance under the umbrella of unified national health insurance, the National Health Insurance Service was founded in 2000.^6^ By 2006, 96·3% of South Koreans had national health insurance (57·7% employed and 38·6% self-employed), with the remaining 3·7% of people covered by medical aid insurance.^5^ Universal health insurance has improved health-care accessibility for all South Koreans, with South Korea having the highest number of health-care-related consultations per citizen (16·9 times a year) among all Organisation for Economic Co-operation and Development (OECD) countries in 2018.^7^

The major funding sources of the National Health Insurance Service are premiums paid by individuals who are insured, government subsidies, and taxes on tobacco sales.^5^ Because funding for the National Health Insurance Service is expected to decrease due to reductions in South Korea's working-age population, the sustainability of the National Health Insurance Service is uncertain, and projections predict cumulative reserves will run out by 2024.^8^ The increasing burden of non-communicable diseases (NCDs) among older people drives up health-care costs, accounting for approximately 41% of total health expenditures in 2019,^9^ which is a number that is expected to grow as the population ages.^10^, ^11^ These factors highlight the need to assess how limited resources can best be distributed to ensure that the health-care system in South Korea remains sustainable. To meet this need, detailed analyses of the nation's epidemiological shift, current and forecasted disease burdens, and relevant risk factors are required to prioritise health needs and guide effective policy actions.

**Research in context**

**Evidence before this study**

Data describing the leading causes of death and disability in South Korea have been compiled by central and local government and public health organisations**.** The Statistics Korea, National Health and Nutrition Examination Survey, Population and Housing Census, and several researches have revealed an increasing trend with unique epidemiological characteristics differing from global patterns. Disability-adjusted life years (DALYs) showed significant increases due to conditions such as low back pain, diabetes, self-harm, and cerebrovascular disease. The results have indicated that the burden of disease in the Korean population is primarily driven by cancer, cardiovascular disease, digestive disease, diabetes, and certain neuro-psychiatric conditions, reflecting the rapid social and economic transitions in South Korea. However, a systematic analysis of the full burden of diseases, injuries, and risk factors has not been done. Comprehensive analyses of burden for causes of mortality and morbidity, stratified by age and sex and including trends over time, are needed to better inform South Korean public health-care policy and practice.

**Added value of this study**

In the 12 years from 1977 to 1989, South Korea was able to achieve universal health coverage (UHC). This study—to the best of our knowledge, the first comprehensive analysis of long-term trends in disease burden in South Korea—covers 30 years of health outcomes since the advent of UHC. We used data from the Global Burden of Disease Study 2019, which provide estimates on 369 diseases and injuries and 87 risk factors in 204 countries from 1990 to 2019. We analysed long-term trends in disease burden from deaths, DALYs, years of life lost (YLLs), and years lived with disability by cause in South Korea from 1990 to 2019, and evaluated mortality rates attributable to population risk factors. We examined differences in disease burden by sex using the ratio of male to female age-standardised YLL rates for the 30 leading causes of death in South Korea in 2019. We also compared outcomes in South Korea with trends globally and within seven Asia-Pacific countries. Finally, we examined forecasted estimates for the leading causes of YLLs in South Korea up to 2040, stratified by sex.

**Implications of all the available evidence**

This study characterises the landscape of population health outcomes in South Korea over the 30 years following the implementation of UHC. South Korea has experienced large reductions in age-standardised mortality and morbidity rates since 1990. However, increasing medical costs and a shrinking workforce due to rapid population ageing are threatening the sustainability of UHC. Health-care resources need to be allocated efficiently, prioritising interventions that address the leading causes of death and disability described in this study. Our results can be used to inform national-level policy responses and provide insights for nations sharing similar features with South Korea.

Previous work has described disease burden in South Korea,^12^, ^13^, ^14^, ^15^ but a comprehensive analysis of long-term trends in mortality and morbidity has not been done. Data from the Global Burden of Disease Study (GBD) have enabled country-specific analyses in multiple nations and can be used to inform public health policy.^16^, ^17^, ^18^, ^19^ In this study, we used data from GBD 2019 to evaluate transitions in population health status in South Korea from 1990 to 2019, and forecasted estimates through to 2040. The aim of the study is to advance national-level policies to improve the accessibility, quality, and equity of health care, examining epidemiological transitions in South Korea.

This manuscript was produced as part of the GBD Collaborator Network and in accordance with the GBD Protocol.

## Methods

### Study design

We used data from GBD 2019, which are publicly available through the Global Health Data Exchange and can be further explored via customised data visualisation tools. Sources of input data are listed in the appendix (pp 4–21). We extracted estimates for deaths, years of life lost (YLLs), years of life lived with disability (YLDs), and disability-adjusted life-years (DALYs) by cause in South Korea from 1990 to 2019, along with forecasted estimates for age-standardised YLLs up to 2040 to examine future trends. DALYs provide an index of overall fatal and non-fatal disease burden and are calculated by summing YLLs (the difference between an individual's age at death due to a condition and life expectancy at the time of death) and YLDs (the number of incident cases of a condition multiplied by the disability weight of that condition and case duration).

We examined the burden of mortality and morbidity by cause across 369 diseases and injuries, categorised within a four-level hierarchy. Level 1 represents the broadest level of organisation in which causes of death and disability are classified as either communicable, maternal, neonatal, and nutritional (CMNN) diseases; NCDs; or injuries (which includes self-harm). Level 2 further subdivides these into 22 causes, Level 3 into 174 causes, and Level 4 into 301 causes.^20^ We present results as total numbers and all-age or age-standardised rates per 100  000 population and include 95% uncertainty intervals (UIs).

Additionally, we extracted the same GBD estimates for seven comparator countries to place data for South Korea in a broader context. We selected seven neighbouring countries across diverse Asia-Pacific regions (four east Asia countries and the most populous country in each Asia-Pacific region): Japan (east Asia), China (east Asia), Mongolia (east Asia), India (south Asia), Australia (Australasia), Russia (northern Asia), and Indonesia (southeast Asia).

The methods used within GBD to estimate mortality and morbidity are refined and expanded with each iteration of the study, and full descriptions are available within the main GBD capstone papers.^20^ Briefly, the three relevant models used in GBD 2019 were the Cause of Death Ensemble model (CODEm), spatiotemporal Gaussian process regression (ST-GPR), and Disease Modelling Meta-Regression (DisMod-MR 2.1).^20^ To estimate cause-specific death rates from heterogeneous data of varying quality, CODEm generates a diverse set of plausible submodels assessing relationships between potential covariates and the response variable, and then constructs a final ensemble model from the submodels based on their out-of-sample predictive validity.^20^ ST-GPR and DisMod-MR 2·1 are meta-regression tools used to model non-fatal health outcomes and relative disease risk. ST-GPR compensates for sparse or heterogeneous data by borrowing strength across location and time, and by incorporating predictive covariates. DisMod-MR 2·1 estimates incidence and prevalence for most causes of disease and injury and their non-fatal outcomes and estimates YLDs by calculating the product of incidence and a specific disability weight for each sequelae, adjusting for comorbidity, and aggregating to the cause level.^20^ YLLs are calculated by multiplying the number of deaths in each age group by a reference life expectancy determined from an analysis of overall mortality rates.

Associations between potential risk factors and disease burden were evaluated using the GBD comparative risk assessment framework in which estimates of theoretical minimum risk exposure level (TMREL), risk exposure, relative disease risk, and attributable burden are generated for 87 behavioural, environmental, occupational, and metabolic risks or groups of risks.^21^ Population attributable fraction—the proportional reduction in disease burden that would occur if risk were reduced to the TMREL—is multiplied by total number of deaths or YLLs to compute the proportion of disease burden attributable to each risk factor.

GBD uses a three-component model of cause-specific mortality to generate the forecasted YLL data presented in this study. The first component incorporates the effects of changes in behavioural, metabolic, and environmental risk factors, along with select interventions;^22^ the second component includes the effect of changes in sociodemographic index (reflecting income per person, educational attainment, and total fertility rate in individuals younger than 25 years);^22^ and the third component represents an autoregressive integrated moving average model to account for unexplained changes that are correlated with time.^22^ Further details on this forecasting model are described elsewhere.^22^

This study complies with the Guidelines for Accurate and Transparent Health Estimates Reporting (appendix p 3).^23^

### Role of the funding source

The funder of this study had no role in study design, data collection, data analysis, data interpretation, or writing of the report.

## Results

**Figure 1:**
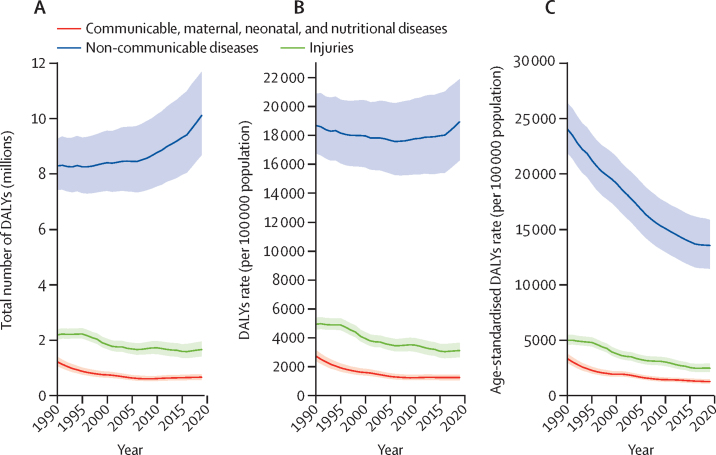
Trends in DALYs in South Korea by GBD Level 1 cause group, 1990–2019 (A) Total number. (B) All-age rates. (C) Age-standardised rates. Cause group included CMND, NCDs, and injuries. The difference in trends between the total number of DALYs and the all-age DALY rate is caused by population growth, and the difference between all-age and age-standardised rates is due to changes in the age distribution within the population. Shaded areas represent 95% uncertainty intervals. DALYs=disability-adjusted life-years. GBD=Global Burden of Diseases, Injuries, and Risk Factors Study.

Age-standardised DALYs and death per 100 000 population for all causes in South Korea decreased by 46·7% (95% UI 43·2–50·3) and 57·9% (56·3–59·3) from 1990 to 2019 (appendix p 59). From 1990 to 2019, the age-standardised DALYs per 100 000 population for NCDs decreased substantially by 43·6% (95% UI 39·4–47·9), from 24 000 (21 900–26 400) to 13 500 (11 400–15 800) per 100 000 population, the greatest decline in the world during this period (appendix pp 23–27). The age-standardised DALYs per 100  000 population for CMNN diseases and injuries also declined during this period and were lower than those for NCDs (figure 1). Age-standardised deaths from NCDs per 100 000 population declined by 58·8% (55·9–60·5), from 774·7 (754·5–782·6) to 319·0 (306·5–334·4) per 100 000 population, again the largest decline in the world during this period (appendix pp 28–32). The age-standardised death rate for CMNN diseases and injuries remained stable during this period (appendix p 122).

**Figure 2:**
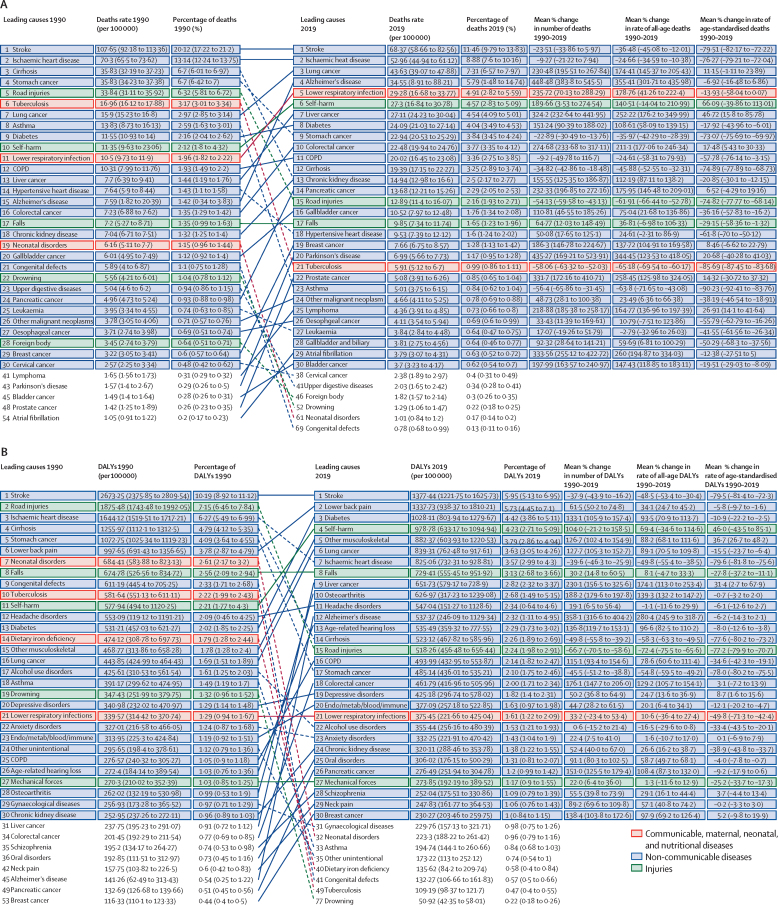
30 leading Level 3 causes of death and DALYs (except prostate cancer) in South Korea in 1990 and 2019 (A) All-age death rates, percentage of all-age deaths, percentage change in number of deaths, percentage change in rate of all-age deaths, and percentage change in rate of age-standardised deaths, 1990–2019. (B) All-age DALY rates, percentage of all-age DALYs, percentage change in number of DALYs, percentage change in rate of all-age DALYs, and percentage change in rate of age-standardised DALYs, 1990–2019. Causes of death and DALYs are connected by arrows between time periods, with solid lines representing increases in rank and dashed lines indicating decreases. Age-related hearing loss=age-related and other hearing loss. Alzheimer's disease=Alzheimer's disease and other dementias. Cirrhosis=cirrhosis and other chronic liver diseases. Colorectal cancer=colon and rectum cancer. Congenital defects=congenital birth defects. COPD=chronic obstructive pulmonary disease. DALY=disability-adjusted life-year. Endo/metab/blood/immune=endocrine, metabolic, blood, and immune disorders. Lung cancer=tracheal, bronchus, and lung cancer. Mechanical forces=exposure to mechanical forces. Other musculoskeletal=other musculoskeletal disorders. Other unintentional=other unintentional injuries.

In 1990, six of the ten leading causes of DALYs in South Korea were NCDs, which increased to eight by 2019 (figure 2). Stroke remained the leading cause of DALYs during the study period even though the number of total DALYs for stroke decreased by 37·9% (95% UI 16·2–43·9) and the percentage of all-cause DALYs due to stroke decreased from 10·2% (8·9–11·1) in 1990 to 6·0% (5·1–7·0) in 2019. In addition to the decrease in total DALYs for stroke, rates of stroke-related age-standardised DALYs decreased by 79·5% (72·3–81·4; figure 2).

CMNN diseases comprised none of the leading ten causes of DALYs in 2019, compared with two in 1990 (figure 2). Neonatal disorders dropped from the seventh leading cause of DALYs in 1990 to the 32nd in 2019. Tuberculosis also dropped from the tenth leading cause in 1990 to the 49th in 2019. Among the 30 leading causes of DALYs in 2019, only one (lower respiratory infections) was a CMNN disease.

Regarding injuries, road injuries dropped from the second leading cause of DALYs in 1990 to the 15th in 2019 (figure 2). Falls remained the eighth leading cause in 2019, although age-standardised rates for falls decreased by 27·8% (95% UI 11·1–37·2). However, self-harm rose from the 11th leading cause of DALYs in 1990 to the fourth in 2019, showing a dramatic increase of 46·0% (–43·5 to 85·1) in age-standardised rates over the study period (figure 2).

**Figure 3:**
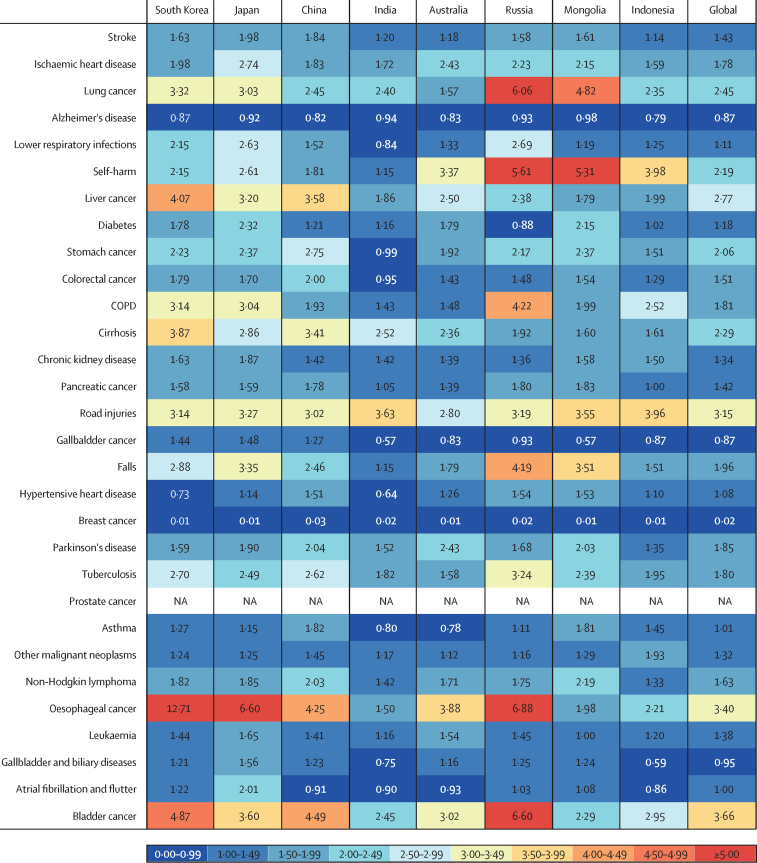
Ratio of male to female age-standardised rates of YLLs in South Korea in 2019 and seven comparator countries for the 30 leading causes of death in South Korea COPD=chronic obstructive pulmonary disease. YLLs=years of life lost.

In 2019, the ratio of male to female age-standardised rates of YLLs in South Korea was higher than 1·00 for 26 of the 29 leading causes of death (excluding prostate cancer; figure 3). These ratios were higher in South Korea than the global average for 22 of the 29 leading causes of death—particularly for lung cancer, liver cancer, chronic obstructive pulmonary disease (COPD), cirrhosis, oesophageal cancer, and bladder cancer—and were equal or lower for seven causes (Alzheimer's disease, self-harm, road injuries, hypertensive heart disease, breast cancer, other malignant neoplasms, and Parkinson's disease). Similar patterns were seen in some comparator countries for several causes of death; Japan and Russia also had high male to female ratios for lung cancer, COPD, oesophageal cancer, and bladder cancer. Additionally, China and Japan had high male to female ratios for liver cancer. Hypertensive heart disease showed lower age-standardised rates of YLLs for males than for females in South Korea, similar to the pattern seen in India (figure 3).

**Figure 4:**
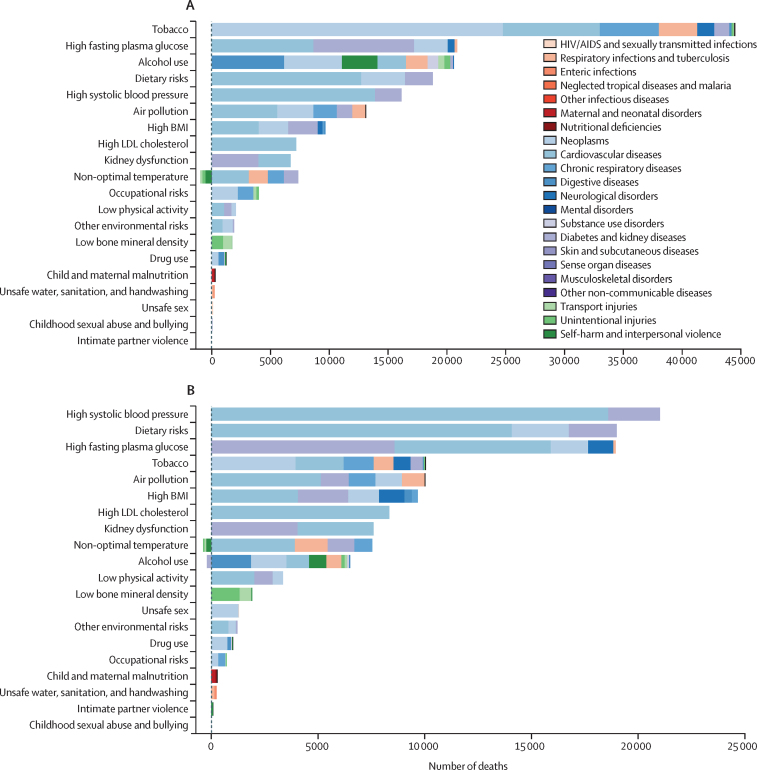
Deaths attributable to Level 2 risk factors in South Korea by sex in 2019 (A) Risk factors for deaths in males. (B) Risk factors for deaths in females.

In terms of risk factors, tobacco use accounted for the highest number of risk-attributable deaths (44 470 [95% UI 37 432–53 989]) among males in South Korea in 2019, contributing substantially to the number of deaths from neoplasms (24 771 [22 331–27 263], accounting for 14·5% of total deaths), cardiovascular diseases (8247 [7364–9990], accounting for 4·8% of total deaths), and, to a lesser degree, chronic respiratory diseases (4986 [4158–5886], accounting for 2·9% of total deaths; figure 4). Among females in South Korea, high systolic blood pressure accounted for the highest number of risk-attributable deaths (21 014 [15 553–26 723]) in 2019, contributing substantially to deaths from cardiovascular diseases (18 594 [13 666–23 799], accounting for 12·6% of total deaths), and, to a lesser extent, diabetes and kidney diseases (2421 [1887–2924], accounting for 1·6% of total deaths; figure 4).

**Figure 5:**
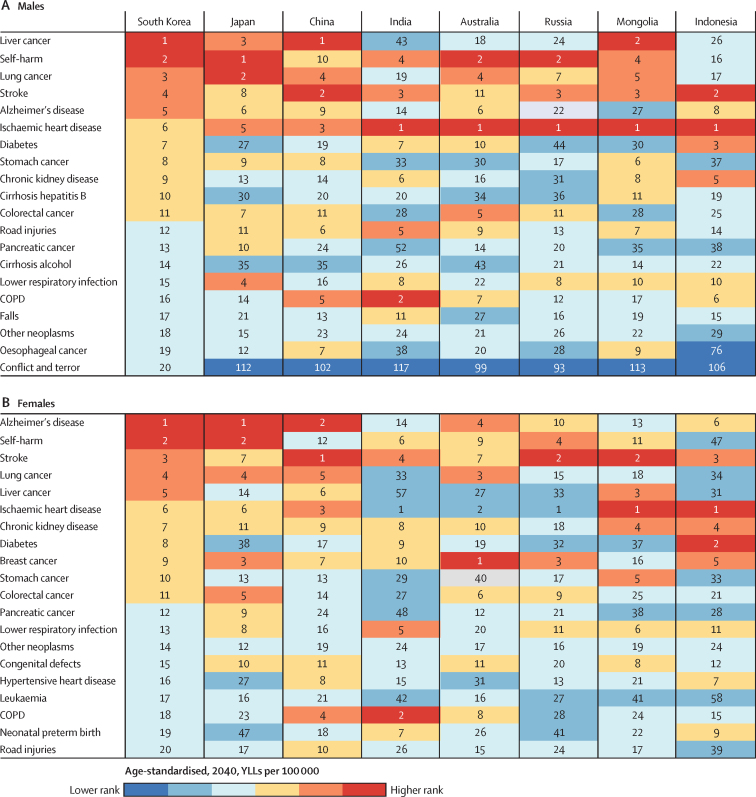
Leading 20 causes of age-standardised YLL rate (per 100 000) in the 2040 forecast for (A) males and (B) females in South Korea The causes are ordered according to rank order in South Korea. COPD=chronic obstructive pulmonary disease. YLLs=years of life lost.

Among the 20 leading causes of age-standardised YLLs in the 2040 forecast, liver cancer, self-harm, lung cancer, stroke, and Alzheimer's disease are projected to be the top five causes in males, a pattern comparable with Japan and Mongolia (figure 5). Although the projected top five causes of YLLs in females in 2040 are identical to those of males, the order is different: Alzheimer's disease is projected to be the leading cause, followed by self-harm, stroke, lung cancer, and liver cancer. This pattern is broadly similar to Japan and China (figure 5).

## Discussion

Our findings show how the epidemiological transition has continued to shift predominant causes of morbidity and mortality in South Korea during the past 30 years; the disease burden in South Korea is now dominated by NCDs. Forecasted estimates of age-standardised YLLs up to 2040 suggest that this trend is likely to continue. Although total deaths and DALYs due to NCDs increased during the study period from 1990 to 2019, the age-standardised death and DALY rates due to NCDs decreased. These findings could reflect that demographic transitions such as population growth and ageing are pushing up the total burden of age-related diseases, combined with improved care for NCDs in the health-care system.^24^ Our study also revealed large differences in the burden of disease in males compared with females, much of which can be attributed to behavioural risk factors such as smoking and alcohol use.

In 2019, eight of the ten leading causes of DALYs in South Korea were NCDs; six of these eight did not rank as a leading cause in 1990. Although stroke was consistently the number one leading cause of death and DALYs in both 1990 and 2019 in South Korea, the age-standardised death and DALY rates due to stroke have substantially decreased over time, as has the age-standardised incidence and prevalence (appendix pp 33–57). During this period, the burden of haemorrhagic stroke has declined to a greater extent compared with ischaemic stroke (appendix pp 33–104). The reduced burden of ischaemic stroke could be attributed to improved pharmaco-prevention for atherosclerosis,^25^ and reductions in haemorrhagic stroke burden are possibly associated with decreases in blood pressure in South Korea.^26^, ^27^ Indeed, our analysis shows that from 1990 to 2019, the age-standardised DALY rates of NCDs attributable to high LDL cholesterol dropped by 73·4% (67·22 to 76·87). Similarly, the rates of NCDs attributable to high systolic blood pressure decreased by 80·4% (74·72–82·88; appendix pp 110–114). Reductions in all-cause stroke burden could also be partly related to rapid increase in average height^26^, ^27^, ^28^, ^29^ and increased accessibility to acute stroke care in South Korea.^30^ Overall cardiovascular disease burden has shown a similar reduction, possibly due to high rates of hypertension treatment and control^31^ and improvement in population risk factors.^21^

Self-harm is projected to continue to rise and become the second highest cause of YLLs in South Korea in 2040 for both males and females. In 2019, death from self-harm exceeded the sum of that from road injuries and falls and increases in the mean percentage change in age-standardised death and DALY rates from suicide between 1990 and 2019 were the highest among the 30 leading causes of death. Suicide is one of the leading causes of death in high-income Asia-Pacific countries including Japan and South Korea. A comparative study observed the highest age-specific suicide rate in Japanese men at middle adulthood (aged 45–64 years) and in Korean men at older age (older than 64 years).^32^ Additionally, suicides among older Korean women were noted.^32^, ^33^ Another study reported that the age-standardised mortality rate for female suicide in South Korea was ranked among the fourth highest globally following Lesotho, Uganda, and Liberia.^34^ Suicide mortality rates increased with age, and the main reason for self-harm in the overall South Korean population was shown to be depression and other psychiatric issues, followed by interpersonal relationships (ie, family and peers), financial problems, and physical illnesses.^35^, ^36^ The South Korean Government has implemented numerous efforts to prevent self-harm-related deaths, including enacting the act on the Prevention of Suicide. However, our data clearly show that suicide is a growing public health concern, especially in vulnerable populations such as older people, and should be a priority for policy makers in South Korea.

Fine particulate matter (PM_2·5_) has been a substantial source of air pollution in South Korea in the past few decades, likely stemming from rapid industrialisation in South Korea and surrounding countries.^37^ The annual average concentration of PM_2·5_ measured from 2005 to 2012 was 27 μg/m^3^, which is much higher than the WHO standard.^37^ In 2015, the annual average PM_2·5_ increased to 30·3 μg/m^3^ in South Korea and 40·5 μg/m^3^ in Seoul, where more than half of the population live. The pattern of leading 30 causes of death in 2019 raises direct concerns about the effect of air pollution on the disease burden in South Korea. GBD 2015 reported that ambient PM_2·5_ was the fifth-ranking mortality risk factor globally, and deaths attributable to ambient PM_2·5_ were highest for ischaemic heart disease, COPD, lower respiratory infection, cerebrovascular disease, and tracheal, bronchial, and lung cancer.^38^ Our study revealed that stroke, ischaemic heart disease, and lung cancer were the top three causes of death, and lower respiratory infection the fifth leading cause in South Korea in 2019; these leading causes of death correspond to the diseases associated with risks due to ambient PM_2·5_.^38^ Our findings urge prompt national interventions to reduce air pollution to mitigate these risks.

We observed large differences in the disease burden for males and females in South Korea. Among the 30 leading causes of death in 2019, age-standardised YLL rates for 26 conditions were higher in men, whereas those for Alzheimer's disease, hypertensive heart disease, and breast cancer were higher in women. Moreover, the ratios of male to female age-standardised YLL rates were higher in South Korea than the global average for 75·9% (22 of 29 diseases) of the leading causes of death, indicating that disease burden falls disproportionally on males. Relative to seven comparator countries, oesophageal cancer, cirrhosis, and liver cancer were more pronounced in males in South Korea, probably due to smoking and alcohol use. Given that major contributors to death in males in South Korea are modifiable behavioural risk factors, interventions targeting smoking and alcohol use are likely to be particularly beneficial.

Since 1989, South Koreans have benefited from UHC.^6^, ^39^ During 30 years of UHC implementation, South Korea has had substantial improvements in age-standardised disease burden, most noticeably with respect to NCDs. Age-standardised DALYs related to NCDs decreased from 1990 to 2019 by 43·6% (39·4–47·9) and for mortality rates by 58·8% (55·9–60·5), showing the greatest declines in the world (appendix pp 23–27, 28–32). Universal health insurance has improved health-care accessibility and quality; South Korea displayed the largest improvements in the Healthcare Access and Quality Index score from 1990 to 2016 among countries with a high sociodemographic index.^40^ Given that continual management and regular medical consultations are required to minimise health loss caused by NCDs, the broad coverage and considerable accessibility to medical services afforded by UHC could have provided the environment to reduce disease burden due to NCDs.^15^

The results described in this study are broadly similar to those reported for Japan (GBD 2015 data).^41^ In both Japan and South Korea, cerebrovascular disease, ischaemic heart disease, lung cancer, Alzheimer's disease, and lower respiratory infection were the top five causes of death; NCDs were predominant among the top 30 causes of death; and the largest contribution to disease burden came from behavioural and metabolic risk factors**.**^41^ This congruence could be explained in part by cultural and demographic features shared by South Korea and Japan. These features include a carbohydrate rich diet, long life expectancy, low fertility rate, and ongoing transition to a super-ageing society.^41^, ^42^ The age-standardised death and DALY rates associated with NCDs have decreased in South Korea and Japan due to multiple factors including improved health-care accessibility and quality, and changes in population risk factors.^41^ Although these rates have decreased, the total number of deaths and DALYs for NCDs has increased probably due to multimorbidity burdens associated with population ageing (figure 1; appendix p 122).^41^ This finding indicates that the UHC systems in South Korea and Japan could be unable to manage the growing disease burden driven by population ageing, despite having one of the best UHC in the world.^42^

The implementation and operation of UHC in South Korea is not without challenges. Inefficient elements of health-care delivery and leakage of health expenditure have been described**.**^4^, ^43^ There is a need to reform the fee-for-service payment system in South Korea and expand health financing.^39^ Indeed, there is growing concern regarding the sustainability of UHC in South Korea. The National Health Insurance Service's cumulative reserves are anticipated to be depleted in the near future, probably due to frequent use of the medical system and a high percentage of expenditures for the age group 65 years and older (reaching approximately 41% in 2019^9^). South Korea ranks highest among all OECD countries for the number of medical consultations per citizen (16·9 times a year in 2019).^44^ Such expenditures are expected to grow as the population ages. Moreover, our forecasting results project that nine of the top ten leading causes of YLLs in 2040 are likely to be NCDs, for both males and females. Therefore, a transition from treatment-based to preventive-based care by implementing rigorous and population-wide policy measures to reduce NCDs and their risk factors could prove to be cost-effective and could improve the sustainability of South Korea's health-care system.^45^

Limitations to our study include those related to GBD methodology, which have been described elsewhere.^16^, ^20^, ^46^ Estimates used here could be incomplete due to time lags and low-quality or a shortage of data from specific regions, age groups, or time periods. Because GBD 2019 did not provide data by subnational regions in South Korea, we were unable to investigate regional variation in the burden of disease. Instead, we compared the disease burden in South Korea with that of other relevant countries, as implemented in previous national GBD studies.^16^, ^17^, ^46^ GBD 2019 used multiple nationally representative data sources, such as South Korea Vital Registration data and Korean National Health and Nutrition Examination Survey data, to estimate the burden of disease in South Korea. Such data sources could be subject to biases associated with coding errors, misdiagnoses, and missing values. The survey data––ie, the Korean National Health and Nutrition Examination Survey data––could be subject to recall bias, non-response bias, and social desirability bias. However, previous work has found South Korea to have one of the highest quality vital registration systems in the world,^47^ and GBD 2019 used robust methods and preprocessing for multiple data sources to address these concerns, as described previously.^20^ Moreover, GBD data for South Korea are limited in their ability to address underlying social and structural factors that can contribute to the observed differences in disease burden between males and females. Future studies should aim to investigate the role of factors such as access to health care, gender-based societal norms, occupational hazards, and biological differences in driving health disparities between males and females. Understanding these factors is crucial for developing targeted interventions and policies aimed at reducing inequities between different population groups. Lastly, changes in health performance and outcomes over the study period resulted from multifactorial causes (ie, population structure and demographic changes, development in medical technologies, and reduced burden attributable to various population risk factors), making it hard to disentangle the unique effect of UHC.

Explanations for the steep reductions in age-standardised morbidity and mortality in South Korea described in this study are multifactorial. The successful implementation of UHC is likely to have contributed to these improvements. However, the benefits to population health associated with UHC are under threat due to rising medical costs and population ageing. There is an urgent need for policy makers in South Korea to consider these effects, and allocate increasingly scarce resources efficiently. Interventions that address the leading causes of death and disability described in this study should be a priority. Our report could also provide insights for nations implementing UHC in the context of population ageing.

## Data sharing

To download the data used in these analyses, please visit the Global Health Data Exchange GBD 2019 website at https://ghdx.healthdata.org/gbd-2019.

For the **Global Health Data Exchange** see https://ghdx.healthdata.org/gbd-2019

For the **customised data visualisation tools** see https://vizhub.healthdata.org/gbd-compare

## Declaration of interests

CK reports support for this study from the Bill & Melinda Gates Foundation, and leadership or fiduciary roles in board, society, committee, or advocacy groups, unpaid with the Korean Society of Community Nutrition, outside this study. All other authors declare no competing interests.
