# Supplementary material for: Population health outcomes in South Korea 1990–2019, and projections up to 2040: a systematic analysis for the Global Burden of Disease Study 2019
Source: Lancet Public Health. 2023 Jul 27;8(8):e639–50. doi: 10.1016/S2468-2667(23)00122-6 (PMC10400799; doi:10.1016/S2468-2667(23)00122-6)
Supplement: Supplementary appendix [file mmc1.pdf]

THE LANCET
Public Health

Supplementary appendix

This appendix formed part of the original submission and has been peer reviewed. We post it as supplied by the authors.

Supplement to: GBD 2019 South Korea BoD Collaborators. Population health outcomes in South Korea 1990–2019, and projections up to 2040: a systematic analysis for the Global Burden of Disease Study 2019. *Lancet Public Health* 2023; **8:** e639–50.

Supplementary appendix

This appendix formed part of the original submission and has been peer reviewed.
We post it as supplied by the authors.

Supplement to: **Population health outcomes in South Korea 1990–2019, and projections up to 2040: a systematic analysis for the Global Burden of Disease Study 2019**

Appendix

Appendix tables

- Appendix table 1. GATHER checklist of information that should be included in reports of global health estimates.
- Appendix table 2. Studies from South Korea used in GBD modeling.
- Appendix table 3. Updates in methodology from GBD 2017 to GBD 2019.
- Appendix table 4. Change (%) in age-standardised DALYs rate from 1990 to 2019 for non-communicable diseases in ascending order. (DALY= Disability-adjusted life years)
- Appendix table 5. Change (%) in age-standardised mortality rate from 1990 to 2019 for non-communicable diseases in ascending order.
- Appendix table 6. Number and age-standardised rate of prevalence and incidence, and percentage change from 1990 to 2019 for both sex by causes.
- Appendix table 7. Number and age-standardised rate of deaths and DALYs, and percentage change from 1990 to 2019 by for both sex causes. (DALY= Disability-adjusted life years)
- Appendix table 8. Number and age-standardised rate of YLLs and YLDs, and percentage change from 1990 to 2019 for both sex by causes. (YLL= Years of life lost, YLD= Years lived with disability)
- Appendix table 9. Age-standardised deaths and DALYs rate, and percentage change from 1990 to 2019 attributed to risk factors for communicable, maternal, neonatal, and nutritional (CMNN) diseases
- Appendix table 10. Age-standardised deaths and DALYs rate, and percentage change from 1990 to 2019 attributed to risk factors for non-communicable diseases (NCDs)
- Appendix table 11. Age-standardised deaths and DALYs rate, and percentage change from 1990 to 2019 attributed to risk factors for injuries
- Appendix table 12. Comparison of estimates between GBD and a national report for the top 10 Level-3 all-ages death rates in South Korea in 2019.
- Appendix table 13. Comparison of lists of International Classification of Diseases (ICD) codes mapped to GBD study and Causes of Death Statistics from Statistics Korea along with the top 10 Level-3 all-ages death rates in South Korea in 2019.

Appendix figures

- Appendix figure 1. Trends in deaths [(A) total number, (B) all-age rates, and (C) age-standardised rates] in South Korea from 1990 to 2019, by GBD Level 1 cause group: communicable, maternal, neonatal, and nutritional (CMNN) diseases; non-communicable diseases (NCDs); and injuries.
- South KoreaAppendix figure 2. Leading 30 Level-3 prevalence in South Korea for 1990 and 2019, with rates of prevalence, percentage of total prevalence and percentage change in number of prevalence, all-age of prevalence, and age-standardised prevalence for both sexes combined for all ages.
- Appendix figure 3. Leading 30 Level-3 incidences in South Korea for 1990 and 2019, with rates of new cases, percentage of total new cases and percentage change in number of new cases, all-age of new cases, and age-standardised new cases for both sexes combined for all ages.
- Appendix figure 4. Trends in age-standardised death and DALY rates of (A) communicable, maternal, neonatal, and nutritional (CMNN) diseases, (B) non-communicable diseases (NCDs), and (C) injuries attributable to GBD level 1 risk factors in South Korea and Global from 1990 to 2019, by GBD Level 1 risk factor group: environmental/occupational risks; behavioral risks; and metabolic risks.

Appendix method 1. Summary of methodology for "Causes of Death Statistics in 2019, South Korea"

Appendix method 2. Summary of redistribution process for cause-of-death modeling in GBD 2019 study

Appendix method 3. Plausible reasons behind the discrepancies between GBD estimates and national report estimates

Author contribution

Appendix table 1. GATHER checklist of information that should be included in reports of global health estimates

#	Checklist item	Section/paragraph/ interpretation
**Objectives and funding**		
1	Define the indicators, populations, and time periods for which estimates were mad e.	Methods
2	List the funding sources for the work.	No funding
**Data Inputs**		
For all data inputs from multiple sources that are synthesized as part of the study:		
3	Describe how the data were identified and how the data were accessed.	As mentioned in the Methods, and the details have been published pre viously.
4	Specify the inclusion and exclusion criteria. Identify all ad-hoc exclusions.	Methods
5	Provide information on all included data sources and their main characteristics. For each data source used, report reference information or contact name/institution, po pulation represented, data collection method, year(s) of data collection, sex and age range, diagnostic criteria or measurement method, and sample size, as relevant.	Available via online data source to ols (http://ghdx.healthdata.org/gbd-2 019/data-input-sources).
6	Identify and describe any categories of input data that have potentially important b iases (e.g., based on characteristics listed in item 5).	As mentioned in the Methods, and the details have been published pre viously.
For data inputs that contribute to the analysis but were not synthesized as part of the study:		
7	Describe and give sources for any other data inputs.	Available via online data source to ols (http://ghdx.healthdata.org/gbd-2 019/data-input-sources).
For all data inputs:		
8	Provide all data inputs in a file format from which data can be efficiently extracte d (e.g., a spreadsheet as opposed to a PDF), including all relevant meta-data listed in item 5. For any data inputs that cannot be shared due to ethical or legal reaso ns, such as third-party ownership, provide a contact name or the name of the insti tution that retains the right to the data.	Available via online data source to ols (http://ghdx.healthdata.org/gbd-2 019/data-input-sources); input data not available in tools will be made available upon request.
**Data analysis**		
9	Provide a conceptual overview of the data analysis method. A diagram may be hel pful.	Flow diagrams of the overall meth odological processes were available online (http://ghdx.healthdata.org/gbd-2019/ code)
10	Provide a detailed description of all steps of the analysis, including mathematical f ormulae. This description should cover, as relevant, data cleaning, data pre-processi ng, data adjustments and weighting of data sources, and mathematical or statistical model(s).	As mentioned in the Methods, and the details have been published pre viously.
11	Describe how candidate models were evaluated and how the final model(s) were s elected.	As mentioned in the Methods, and the details have been published pre viously.
12	Provide the results of an evaluation of model performance, if done, as well as the results of any relevant sensitivity analysis.	As mentioned in the Methods, and the details have been published pre viously.
13	Describe methods for calculating uncertainty of the estimates. State which sources of uncertainty were, and were not, accounted for in the uncertainty analysis.	As mentioned in the Methods, and the details have been published pre viously.
14	State how analytic or statistical source code used to generate estimates can be acc essed.	Methods
**Results and Discussion**		
15	Provide published estimates in a file format from which data can be efficiently ext racted.	Results, and online data tools (data visualization tools, and data query tools, http://ghdx.healthdata.org/gbd-2019)
16	Report a quantitative measure of the uncertainty of the estimates (e.g. uncertainty i ntervals).	Results, and online data tools (data visualization tools, and data query tools, http://ghdx.healthdata.org/gbd-2019)
17	Interpret results in light of existing evidence. If updating a previous set of estimat es, describe the reasons for changes in estimates.	Discussion
18	Discuss limitations of the estimates. Include a discussion of any modelling assumpt ions or data limitations that affect interpretation of the estimates.	Discussion

Appendix table 2. Studies from South Korea used in GBD modeling

The number of total citations for modeling of South Korea is 592 and 1,706,936 of metadata rows are used.

Citations
National Bureau of Statistics (South Korea). South Korea Population and Housing Census 1980
National Bureau of Statistics (South Korea). South Korea Population and Housing Census 1985
National Statistical Office (South Korea). South Korea Population and Housing Census 1990
National Bureau of Statistics (South Korea), Economic Planning Board, International Statistical Institute. Korea, Rep. World Fertility Survey 1974. Voorburg, Netherlands: International Statistical Institute
South Korea Vital Registration - Deaths 1995 ICD10
South Korea Vital Registration - Deaths 1996 ICD10
South Korea Vital Registration - Deaths 1997 ICD10
South Korea Vital Registration - Deaths 1998 ICD10
South Korea Vital Registration - Deaths 1999 ICD10
South Korea Vital Registration - Deaths 2000 ICD10
South Korea Vital Registration - Deaths 2001 ICD10
South Korea Vital Registration - Deaths 2002 ICD10
South Korea Vital Registration - Deaths 2003 ICD10
South Korea Vital Registration - Deaths 2004 ICD10
South Korea Vital Registration - Deaths 2005 ICD10
South Korea Vital Registration - Deaths 2006 ICD10
International Road Federation. World Road Statistics 1963-1999. Geneva, Switzerland: International Road Federation
Centers for Disease Control and Prevention (CDC) and World Health Organization (WHO). Korea, South Global Youth Tobacco Survey 2005. United States: Centers for Disease Control and Prevention (CDC), 2005
South Korea Vital Registration - Deaths 2009 ICD10
United Nations Statistics Division (UNSD). United Nations Demographic Yearbook 2000. New York, United States of America: United Nations (UN), 2002
United Nations Statistics Division (UNSD). United Nations Demographic Yearbook 2001. New York, United States of America: United Nations (UN), 2003
United Nations Statistics Division (UNSD). United Nations Demographic Yearbook 2003. New York, United States of America: United Nations (UN), 2006
United Nations Statistics Division (UNSD). United Nations Demographic Yearbook 2004. New York, United States of America: United Nations (UN), 2007
United Nations Statistics Division (UNSD). United Nations Demographic Yearbook 2007. New York, United States of America: United Nations (UN), 2009
United Nations Statistics Division (UNSD). United Nations Demographic Yearbook 2008. New York, United States of America: United Nations (UN), 2010
United Nations Statistics Division (UNSD). United Nations Demographic Yearbook 2009-2010. New York, United States of America: United Nations (UN), 2011
Organization for Economic Co-operation and Development (OECD). OECD Health Statistics. Paris, France: Organization for Economic Co-operation and Development (OECD)
World Health Organization (WHO). WHO Household Energy Database 1974-2008. Geneva, Switzerland: World Health Organization (WHO), 2010
International Monetary Fund (IMF). World Economic Outlook Database. Washington, D.C., United States of America: International Monetary Fund (IMF)
World Health Organization (WHO). Control of Foodborne Trematode Infections 1995. Geneva, Switzerland: World Health Organization (WHO), 1995
British Geological Survey, Natural Environment Research Council. World Mineral Production 2007-2011. Nottingham, United Kingdom: British Geological Survey, Natural Environment Research Council, 2013
United Nations Office on Drugs and Crime (UNODC). World Drug Report 2012. Vienna, Austria: United Nations Office on Drugs and Crime (UNODC), 2012
US Geological Survey (USGS). United States Geological Survey Minerals Yearbook 2011. Reston, United States: US Geological Survey (USGS)
United Nations Statistics Division (UNSD). United Nations Demographic Yearbook 2011. New York, United States of America: United Nations (UN), 2012
United Nations Children's Fund (UNICEF), World Health Organization (WHO). WHO and UNICEF Reported Disease Incidence Time Series. Geneva, Switzerland: World Health Organization (WHO)
United Nations Statistics Division (UNSD). National Accounts Main Aggregates Database. New York City, United States of America: United Nations Statistics Division (UNSD)
World Health Organization (WHO). WHO Global Health Expenditure Database. Geneva, Switzerland: World Health Organization (WHO)

Center for Human Resource Research, Ohio State University, Korea Labor Institute (South Korea), RAND Corporation, Statistics Netherlands. Korean Longitudinal Study of Ageing 2006. Seoul, South Korea: Korea Labor Institute (South Korea)
Korea Central Cancer Registry, Ministry of Health and Welfare (South Korea), National Cancer Center (South Korea). South Korea - Annual Report of Cancer Statistics in Korea in 2010. Goyang-si, South Korea: National Cancer Center (South Korea), 2012
Korea Institute for Health and Social Affairs (KIHASA). South Korea National Fertility and Family Health Survey 2003
Korea Centers for Disease Control and Prevention (KCDC). South Korea National Health and Nutrition Examination Survey 2005
South Korea Vital Registration - Deaths 2007 ICD10
South Korea Vital Registration - Deaths 2010 ICD10
South Korea Vital Registration - Deaths 2011 ICD10
South Korea Vital Registration - Deaths 2008 ICD10
Jeong JH. Prevalence of and Risk Factors for Atrial Fibrillation in Korean Adults Older than 40 Years. J Korean Med Sci. 2005; 20(1): 26-30
Shin DH, Yu HS, Park JH, Shin JH, Kim SJ. Recently occurring adult tetanus in Korea: emphasis on immunization and awareness of tetanus. J Korean Med Sci. 2003; 18(1): 11--6
Kim CS, Jung HW, Yoo KY. Prevalence of otitis media and allied diseases in Korea--results of a nation-wide survey, 1991. J Korean Med Sci. 1993; 8(1): 34-40
Williams H, Stewart A, Von Mutius E, Cookson W, Anderson HR. Is eczema really on the increase worldwide. J Allergy Clin Immunol. 2008; 121(4): 947-954
United Nations Office on Drugs and Crime (UNODC). United Nations Office on Drugs and Crime Global Study on Homicide 2011. Vienna, Austria: United Nations Office on Drugs and Crime (UNODC), 2011
Centers for Disease Control and Prevention (CDC), World Health Organization (WHO). South Korea Global Youth Tobacco Survey 2008. Atlanta, United States of America: Centers for Disease Control and Prevention (CDC)
United Nations Statistics Division (UNSD). United Nations Demographic Yearbook Special Census Topic, 2000 Round. New York, United States of America: United Nations (UN)
Cho HK, Lee H, Kim K-H, Kang JH, Kim J-H, Hur JK, Kim KN, Kim DS, Kim YK, Kim JS, Kim CH, Kim HM, Park S-E, Oh SH, Chung EH, Cha SH, Choi YY, Hong YJ, Lee HJ. The causative organisms of bacterial meningitis in Korean children in 1996-2005. J Korean Med Sci. 2010; 25(6): 895-9
Kim JS, Jang YT, Kim JD, Park TH, Park JM, Kilgore PE, Kennedy WA, Park E, Nyambat B, Kim DR, Hwang PH, Kim SJ, Eun SH, Lee HS, Cho JH, Kim YS, Chang SJ, Huang HF, Clemens JD, Ward JI. Incidence of Haemophilus influenzae type b and other invasive diseases in South Korean children. Vaccine. 2004; 22(29-30): 3952-62
Lee SY, Hong SK, Lee SG, Suh CI, Park SW, Lee JH, Kim JH, Kim DS, Kim HM, Jang YT, Ma SH, Kim SY, Sohn YS, Kang JH, Paik SY. Human rotavirus genotypes in hospitalized children, South Korea, April 2005 to March 2007. Vaccine. 2009; 27(Suppl 5): 97-101
Cheun H-I, Cho S-H, Lee J-H, Lim Y-Y, Jeon J-H, Yu J-R, Kim T-S, Lee W-J, Cho S-H, Lee D-Y, Park M-S, Jeong H-S, Chen D-S, Ji Y-M, Kwon M-H. Infection status of hospitalized diarrheal patients with gastrointestinal protozoa, bacteria, and viruses in the South Korea. Korean J Parasitol. 2010; 48(2): 113-20
Worldwide variations in the prevalence of asthma symptoms: the International Study of Asthma and Allergies in Childhood (ISAAC). Eur Respir J. 1998; 12(2): 315-35
Lai CK, Beasley R, Crane J, Foliaki S, Shah J, Weiland S. Global variation in the prevalence and severity of asthma symptoms: phase three of the International Study of Asthma and Allergies in Childhood (ISAAC). Thorax. 2009; 64(6): 476-483
Korea Centers for Disease Control and Prevention (KCDC). South Korea National Health and Nutrition Examination Survey 2001
Korea Centers for Disease Control and Prevention (KCDC). South Korea National Health and Nutrition Examination Survey 1998
Korea Centers for Disease Control and Prevention (KCDC). South Korea National Health and Nutrition Examination Survey 2007
Korea Centers for Disease Control and Prevention (KCDC). South Korea National Health and Nutrition Examination Survey 2008
Korea Centers for Disease Control and Prevention (KCDC). South Korea National Health and Nutrition Examination Survey 2009
Park Y, Lee H, Koh CS, Min H, Yoo K, Kim Y, Shin Y. Prevalence of diabetes and IGT in Yonchon County, South Korea. Diabetes Care. 1995; 18(4): 545-8
Roh JK, Kim JS, Ahn YO. Epidemiologic and clinical characteristics of migraine and tension-type headache in Korea. Headache. 1998; 38(5): 356-65
Hwang G-S, Choi J-W, Yoo Y, Choung J-T, Yoon C-S. Residential environmental risk factors for childhood asthma prevalence in metropolitan and semirural cities in Korea. Asia Pac J Public Health. 2012; 24(1): 58-67
Kim B-J, Kwon J-W, Seo J-H, Kim H-B, Lee S-Y, Park K-S, Yu J, Kim H-C, Leem J-H, Sakong J, Kim S-Y, Lee C-G, Kang D-M, Ha M, Hong Y-C, Kwon H-J, Hong S-J. Association of ozone exposure with asthma, allergic rhinitis, and allergic sensitization. Ann Allergy Asthma Immunol. 2011; 107(3): 214-219e1
Song W-J, Kim S-H, Lim S, Park Y-J, Kim M-H, Lee S-M, Lee S-B, Kim K-W, Jang H-C, Cho S-H, Min K-U, Chang Y-S. Association between obesity and asthma in the elderly population: potential roles of abdominal subcutaneous adiposity and sarcopenia. Ann Allergy Asthma Immunol. 2012; 109(4): 243-8
Suh M, Kim H-H, Choi DP, Kim KW, Sohn MH, Ha KH, Hwang WJ, Kim C, Kim K-E, Shin DC. Association between body mass index and asthma symptoms among Korean children: a nation-wide study. J Korean Med Sci. 2011; 26(12): 1541-7
Suh M, Kim H-H, Sohn MH, Kim K-E, Kim C, Shin DC. Prevalence of allergic diseases among Korean school-age children: a nationwide cross-sectional questionnaire study. J Korean Med Sci. 2011; 26(3): 332-8
Cho HJ, Chang CB, Kim KW, Park JH, Yoo JH, Koh IJ, Kim TK. Gender and prevalence of knee osteoarthritis types in elderly Koreans. J Arthroplasty. 2011; 26(7): 994-9
Kim I, Kim HA, Seo Y-I, Song YW, Jeong J-Y, Kim DH. The prevalence of knee osteoarthritis in elderly community residents in Korea. J Korean Med Sci. 2010; 25(2): 293-8
Lee S, Kim T-N, Kim S-H. Sarcopenic obesity is more closely associated with knee osteoarthritis than is nonsarcopenic obesity: a cross-sectional study. Arthritis Rheum. 2012; 64(12): 3947-54

Chung CY, Park MS, Lee KM, Lee SH, Kim TK, Kim KW, Park JH, Lee JJ. Hip osteoarthritis and risk factors in elderly Korean population. Osteoarthr Cartil. 2010; 18(3): 312-6
Lee SS, Byoun Y-S, Jeong S-H, Kim YM, Gil H, Min B-Y, Seong MH, Jang ES, Kim J-W. Type and cause of liver disease in Korea: single-center experience, 2005-2010. Clin Mol Hepatol. 2012; 18(3): 309-15
Joo H, Park J, Lee SD, Oh Y-M. Comorbidities of chronic obstructive pulmonary disease in Koreans: a population-based study. J Korean Med Sci. 2012; 27(8): 901-6
Yoo KH, Kim YS, Sheen SS, Park JH, Hwang YI, Kim S-H, Yoon HI, Lim SC, Park JY, Park SJ, Seo KH, Kim KU, Oh Y-M, Lee NY, Kim JS, Oh KW, Kim YT, Park I-W, Lee S-D, Kim SK, Kim YK, Han SK. Prevalence of chronic obstructive pulmonary disease in Korea: the fourth Korean National Health and Nutrition Examination Survey, 2008. Respirol. 2011; 16(4): 659-65
Choi WJ, Ko JY, Kim JW, Lee KH, Park CW, Kim KH, Kim MN, Lee AY, Cho SH, Park YL, Choi JH, Seo SJ, Lee YW, Roh JY, Park YM, Kim DJ, Ro YS. Prevalence and risk factors for atopic dermatitis: a cross-sectional study of 6,453 Korean preschool children. Acta Derm Venereol. 2012; 92(5): 467-71
Lee J-Y, Seo J-H, Kwon J-W, Yu J, Kim B-J, Lee S-Y, Kim H-B, Kim W-K, Kim K-W, Shin Y-J, Hong S-J. Exposure to gene-environment interactions before 1 year of age may favor the development of atopic dermatitis. Int Arch Allergy Immunol. 2012; 157(4): 363-71
Odhiambo JA, Williams HC, Clayton TO, Robertson CF, Asher MI, ISAAC Phase Three Study Group. Global variations in prevalence of eczema symptoms in children from ISAAC Phase Three. J Allergy Clin Immunol. 2009; 124(6): 1251-1258
Han D-H, Kim J-B, Park D-Y. The decline in dental caries among children of different ages in Korea, 2000-2006. Int Dent J. 2010; 60(5): 329-35
Forman D, Bray F, Brewster DH, Gombe Mbalawa C, Kohler B, Piñeros M, Steliarova-Foucher E, Swaminathan R and Ferlay J, eds (2013). Cancer Incidence in Five Continents, Vol. X Summary Database (electronic version). Lyon, IARC. http://ci5.iarc.fr
South Korea Cancer Registry 2003-2007 - CI5 as it appears in Forman D, Bray F, Brewster DH, Gombe Mbalawa C, Kohler B, Piñeros M, Steliarova-Foucher E, Swaminathan R and Ferlay J, eds (2013). Cancer Incidence in Five Continents, Vol. X Summary Database (electronic version). Lyon, IARC. http://ci5.iarc.fr
Zimmermann A, Bernuit D, Gerlinger C, Schaefers M, Geppert K. Prevalence, symptoms and management of uterine fibroids: an international internet-based survey of 21,746 women. BMC Womens Health. 2012; 12: 6
Cho NH, Jung YO, Lim SH, Chung C-K, Kim HA. The prevalence and risk factors of low back pain in rural community residents of Korea. Spine. 2012; 37(24): 2001-10
Huh J-W, Kim W-H, Moon S-G, Lee J-B, Lim Y-H. Viral etiology and incidence associated with acute gastroenteritis in a 5-year survey in Gyeonggi province, South Korea. J Clin Virol. 2009; 44(2): 152-6
Lee JI, Lee G-C, Chung JY, Han TH, Lee YK, Kim MS, Lee CH. Detection and molecular characterization of adenoviruses in Korean children hospitalized with acute gastroenteritis. Microbiol Immunol. 2012; 56(8): 523-8
Park SH, Kim EJ, Oh SA, Kim CK, Choi SS, Cho SJ, Han KY, Lee JI, Kim MY, Jung HS, Chun DS, Kim HS. Viral agents associated with acute gastroenteritis in Seoul, Korea. Clin Lab. 2011; 57(1-2): 59-65
Kim KW, Park JH, Kim M-H, Kim MD, Kim B-J, Kim S-K, Kim JL, Moon SW, Bae JN, Woo JI, Ryu S-H, Yoon JC, Lee N-J, Lee DY, Lee DW, Lee SB, Lee JJ, Lee J-Y, Lee C-U, Chang SM, Jhoo JH, Cho MJ. A nationwide survey on the prevalence of dementia and mild cognitive impairment in South Korea. J Alzheimers Dis. 2011; 23(2): 281-91
Kim J, Jeong I, Chun J-H, Lee S. The prevalence of dementia in a metropolitan city of South Korea. Int J Geriatr Psychiatry. 2003; 18(7): 617-22
Lee DY, Lee JH, Ju Y-S, Lee KU, Kim KW, Jhoo JH, Yoon JC, Ha J, Woo JI. The prevalence of dementia in older people in an urban population of Korea: the Seoul study. J Am Geriatr Soc. 2002; 50(7): 1233-9
Suh G-H, Kim JK, Cho MJ. Community study of dementia in the older Korean rural population. Aust N Z J Psychiatry. 2003; 37(5): 606-12
Kim RB, Kim B-G, Kim Y-M, Seo JW, Lim YS, Kim HS, Lee HJ, Moon JY, Kim KY, Shin J-Y, Park H-K, Song J-K, Park K-S, Jeong BG, Park CG, Shin H-Y, Kang J-W, Oh G-J, Lee Y-H, Seong I-W, Yoo W-S, Hong Y-S. Trends in the Incidence of Hospitalized Acute Myocardial Infarction and Stroke in Korea, 2006-2010. J Korean Med Sci. 2013; 28(1): 16
Lee J-H, Cho HK, Kim K-H, Kim CH, Kim DS, Kim KN, Cha S-H, Oh SH, Hur JK, Kang JH, Kim JH, Kim Y-K, Hong YJ, Chung EH, Park S-E, Choi YY, Kim JS, Kim HM, Choi EH, Lee HJ. Etiology of invasive bacterial infections in immunocompetent children in Korea (1996-2005): a retrospective multicenter study. J Korean Med Sci. 2011; 26(2): 174-83
Cho MJ, Kim J-K, Jeon HJ, Suh T, Chung I-W, Hong JP, Bae J-N, Lee D-W, Park J-I, Cho S-J, Lee C-K, Hahm B-J. Lifetime and 12-month prevalence of DSM-IV psychiatric disorders among Korean adults. J Nerv Ment Dis. 2007; 195(3): 203-10
Korea Centers for Disease Control and Prevention (KCDC). South Korea National Health and Nutrition Examination Survey 2011
Kim DS, Kim YS, Jung KS, Chang JH, Lim CM, Lee JH, Uh ST, Shim JJ, Lew WJ, Korean Academy of Tuberculosis and Respiratory Diseases. Prevalence of chronic obstructive pulmonary disease in Korea: a population-based spirometry survey. Am J Respir Crit Care Med. 2005; 172(7): 842-7
Kim SJ, Suk MH, Choi HM, Kimm KC, Jung KH, Lee SY, Lee SY, Kim JH, Shin C, Shim JJ, In KH, Kang KH, Yoo SH. The local prevalence of COPD by post-bronchodilator GOLD criteria in Korea. Int J Tuberc Lung Dis. 2006; 10(12): 1393-8
Kim YS, Leventhal BL, Koh Y-J, Fombonne E, Laska E, Lim E-C, Cheon K-A, Kim S-J, Kim Y-K, Lee H, Song D-H, Grinker RR. Prevalence of Autism Spectrum Disorders in a Total Population Sample. Am J Psychiatry. 2011; 168(9): 904-12
Yoo HI, Cho SC, Kim BN, Kim SY, Shin MS, Hong KE. Psychiatric Morbidity of Second and Third Grade Primary School Children in Korea. Child Psychiatry Hum Dev. 2005; 36(2): 215-25
Choi D, Lee D-Y, Lehert P, Lee IS, Kim SH, Dennerstein L. The impact of premenstrual symptoms on activities of daily life in Korean women. J Psychosom Obstet Gynaecol. 2010; 31(1): 10-5
Franceschi S, Smith JS, van den Brule A, Herrero R, Arslan A, Anh P-T-H, Bosch FX, Hieu N-T, Matos E, Posso H, Qiao Y-L, Shin H-R, Sukvirach S, Thomas JO, Snijders PJF, Muñoz N, Meijer CJLM. Cervical infection with Chlamydia trachomatis and Neisseria gonorrhoeae in women from ten areas in four continents. A cross-sectional study. Sex Transm Dis. 2007; 34(8): 563-9
Lee S-J, Cho Y-H, Ha U-S, Kim SW, Yoon MS, Bae K. Sexual behavior survey and screening for chlamydia and gonorrhea in university students in South Korea. Int J Urol. 2005; 12(2): 187-93
United Nations Statistics Division (UNSD). United Nations Demographic Yearbook 2012. New York, United States of America: United Nations (UN), 2013
Bhang S-Y, Cho S-C, Kim J-W, Hong Y-C, Shin M-S, Yoo HJ, Cho IH, Kim Y, Kim B-N. Relationship between blood manganese levels and children's attention, cognition, behavior, and academic performance-A nationwide cross-sectional study. Environ Res. 2013; 9-16

Cho S-C, Kim B-N, Hong Y-C, Shin M-S, Yoo HJ, Kim J-W, Bhang S-Y, Cho IH, Kim H-W. Effect of environmental exposure to lead and tobacco smoke on inattentive and hyperactive symptoms and neurocognitive performance in children. J Child Psychol Psychiatry. 2010; 51(9): 1050-7

Hong Y-C, Oh S-Y, Kwon S-O, Park M-S, Kim H, Leem J-H, Ha E-H. Blood lead level modifies the association between dietary antioxidants and oxidative stress in an urban adult population. Br J Nutr. 2013; 109(1): 148-54

Kim Y, Cho S-C, Kim B-N, Hong Y-C, Shin M-S, Yoo H-J, Kim J-W, Bhang S-Y. Association between blood lead levels (<5 μg/dL) and inattention-hyperactivity and neurocognitive profiles in school-aged Korean children. Sci Total Environ. 2010; 408(23): 5737-43

Lee JW, Lee CK, Moon CS, Choi IJ, Lee KJ, Yi S-M, Jang B-K, Yoon BJ, Kim DS, Peak D, Sul D, Oh E, Im H, Kang HS, Kim J, Lee J-T, Kim K, Park KL, Ahn R, Park SH, Kim SC, Park C-H, Lee JH. Korea National Survey for Environmental Pollutants in the Human Body 2008: heavy metals in the blood or urine of the Korean population. Int J Hyg Environ Health. 2012; 215(4): 449-57

Min J-Y, Min K-B, Kim R, Cho S-I, Paek D. Blood lead levels and increased bronchial responsiveness. Biol Trace Elem Res. 2008; 123(1-3): 41-6

Yang S-K, Yun S, Kim J-H, Park JY, Kim HY, Kim Y-H, Chang DK, Kim JS, Song IS, Park JB, Park E-R, Kim KJ, Moon G, Yang SH. Epidemiology of inflammatory bowel disease in the Songpa-Kangdong district, Seoul, Korea, 1986-2005: a KASID study. Inflamm Bowel Dis. 2008; 14(4): 542-9

The INTERSALT Co-operative Research Group. Appendix tables. Centre-specific results by age and sex. J Hum Hypertens 1989;3(5):331-407

Kwon J-W, Lee HJ, Hyun MK, Choi JE, Kim JH, Lee NR, Hwang JS, Lee EJ. Trends in the incidence of subarachnoid hemorrhage in South Korea from 2006-2009: an ecological study. World Neurosurg. 2013; 79(3-4): 499-503

Kim S, Lim CS, Han DC, Kim GS, Chin HJ, Kim SJ, Cho WY, Kim YH, Kim YS. The prevalence of chronic kidney disease (CKD) and the associated factors to CKD in urban Korea: a population-based cross-sectional epidemiologic study. J Korean Med Sci. 2009; 24(Suppl): S11-21

Weissman MM, Bland RC, Canino GJ, Faravelli C, Greenwald S, Hwu HG, Joyce PR, Karam EG, Lee CK, Lellouch J, Lépine JP, Newman SC, Rubio-Stipec M, Wells JE, Wickramaratne PJ, Wittchen H, Yeh EK. Cross-national epidemiology of major depression and bipolar disorder. JAMA. 1996; 276(4): 293-9

Han Y, Kim B, Baek I, Lee D, Kim K, Dong S, Kim H, Chang Y, Lee J, Chang R. [The change of the etiology, complications and cause of death of the liver cirrhosis in 1990s]. Korean J Hepatol. 2000; 6(3): 328-39

Kim YS, Um SH, Ryu HS, Lee JB, Lee JW, Park DK, Kim YS, Jin YT, Chun HJ, Lee HS, Lee SW, Choi JH, Kim CD, Hyun JH. The prognosis of liver cirrhosis in recent years in Korea. J Korean Med Sci. 2003; 18(6): 833-41

Shim J, Kim B-H, Kim NH, Dong SH, Kim HJ, Chang YW, Lee JI, Chang R. Clinical features of HBsAg-negative but anti-HBc-positive hepatocellular carcinoma in a hepatitis B virus endemic area. J Gastroenterol Hepatol. 2005; 20(5): 746-51

Park HA. The Korea National Health and Nutrition Examination Survey as a Primary Data Source. Korean J Fam Med. 2013; 34(2): 79

Korea Centers for Disease Control and Prevention (KCDC). South Korea Youth Risk Behavior Web-Based Survey 2012

Hong YP, Kim SJ, Lew WJ, Lee EK, Han YC. The seventh nationwide tuberculosis prevalence survey in Korea, 1995. Int J Tuberc Lung Dis. 1998; 2(1): 27-36

World Health Organization (WHO). WHO Tuberculosis Case Notifications. Geneva, Switzerland: World Health Organization (WHO)

Cho S-C, Kim B-N, Kim J-W, Rohde LA, Hwang J-W, Chungh D-S, Shin M-S, Lyoo IK, Go B-J, Lee S-E, Kim H-W. Full syndrome and subthreshold attention-deficit/hyperactivity disorder in a Korean community sample: comorbidity and temperament findings. Eur Child Adolesc Psychiatry. 2009; 18(7): 447-57

Chu MK, Oh K, Kim B-K. Prevalence and impact of chronic daily headache in Korea: Results from Korean headache survey. Cephalalgia. 2011; 31(Suppl 1): 138-9

Choe H-S, Lee S-J, Kim CS, Cho Y-H. Prevalence of sexually transmitted infections and the sexual behavior of elderly people presenting to health examination centers in Korea. J Infect Chemother. 2011; 17(4): 456-61

Choe H-S, Lee DS, Lee S-J, Lee CB, Lee WC, Cho Y-H. Prevalence of sexually transmitted infections and sexual behavior of young adults and middle-aged people presenting to health examination centers in Korea. J Infect Chemother. 2012; 18(2): 207-12

Kim O, Kim S-S, Park M-S, Suh S-D, Lee M-W, Kim K-S, Yoon J-D, Lee J-S. Seroprevalence of sexually transmitted viruses in Korean populations including HIV-seropositive individuals. Int J STD AIDS. 2003; 14(1): 46-9

Lee HY, Won JC, Kang YJ, Yoon SH, Choi EO, Bae JY, Sung MH, Kim H-R, Yang JH, Oh J, Lee YM, Park NH, Ko KS, Rhee BD. Type 2 diabetes in urban and rural districts in Korea: factors associated with prevalence difference. J Korean Med Sci. 2010; 25(12): 1777-83

Song K-H, Nam-Goomg IS, Han S-M, Kim M-S, Lee E-J, Lee YS, Lee MS, Yoon S, Lee K-U, Park J-Y. Change in prevalence and 6-year incidence of diabetes and impaired fasting glucose in Korean subjects living in a rural area. Diabetes Res Clin Pract. 2007; 78(3): 378-84

Park K, Kim YS, Kim SI, Kim MS, Kim HJ, Jeon KO. Kidney transplantation in Korea: past and present. Clin Transpl. 2000; 376-8

Curado MP, Edwards B, Shin HR, Storm H, Ferlay J, Heanue M and Boyle P, eds (2007). Cancer Incidence in Five Continents, Vol. IX Periodic Data (electronic version). Lyon, IARC. http://ci5.iarc.fr

South Korea - Jeju Cancer Registry 2000-2002 - CI5 as it appears in Curado MP, Edwards B, Shin HR, Storm H, Ferlay J, Heanue M and Boyle P, eds (2007). Cancer Incidence in Five Continents, Vol. IX Periodic Data (electronic version). Lyon, IARC. http://ci5.iarc.fr

South Korea Cancer Registry 1998-2002 - CI5 as it appears in Curado MP, Edwards B, Shin HR, Storm H, Ferlay J, Heanue M and Boyle P, eds (2007). Cancer Incidence in Five Continents, Vol. IX Periodic Data (electronic version). Lyon, IARC. http://ci5.iarc.fr

Parkin DM, International Agency for Research on Cancer, International Association of Cancer Registries. Cancer Incidence in Five Continents. Vol. I to VIII. Lyon, France, IARCPress, 2005

South Korea - Busan Cancer Registry 1996-1997 - CI5 as it appears in Parkin DM, International Agency for Research on Cancer, International Association of Cancer Registries. Cancer Incidence in Five Continents. Vol. I to VIII. Lyon, France, IARCPress, 2005

South Korea - Daegu Cancer Registry 1997-1998 - CI5 as it appears in Parkin DM, International Agency for Research on Cancer, International Association of Cancer Registries. Cancer Incidence in Five Continents. Vol. I to VIII. Lyon, France, IARCPress, 2005

South Korea - Kangwha County Cancer Registry 1986-1992 - CI5 as it appears in Parkin DM, International Agency for Research on Cancer, International Association of Cancer Registries. Cancer Incidence in Five Continents. Vol. I to VIII. Lyon, France, IARCPress, 2005

South Korea - Kangwha County Cancer Registry 1993-1997 - CI5 as it appears in Parkin DM, International Agency for Research on Cancer, International Association of Cancer Registries. Cancer Incidence in Five Continents. Vol. I to VIII. Lyon, France, IARCPress, 2005

South Korea - Seoul Cancer Registry 1993-1997 - CI5 as it appears in Parkin DM, International Agency for Research on Cancer, International Association of Cancer Registries. Cancer Incidence in Five Continents. Vol. I to VIII. Lyon, France, IARCPress, 2005

Korea Central Cancer Registry, Ministry of Health and Welfare (South Korea). Annual Report of Cancer Statistics in Korea in 2008. Goyang-si, South Korea: National Cancer Center (South Korea), 2010
Korea Central Cancer Registry, Ministry of Health and Welfare (South Korea). KOSIS Database - Incidence of 61 Cancers by Sex and 5-Year Age Groups - Korean Statistical Information Service. Seoul, South Korea: Statistics Korea (South Korea)
Lee HK, Chou SP, Cho MJ, Park J-I, Dawson DA, Grant BF. The prevalence and correlates of alcohol use disorders in the United States and Korea – a cross-national comparative study. Alcohol. 2010; 44(4): 297-306
Min S, Noh S, Shin J, Ahn J-S, Kim T-H. Alcohol dependence, mortality, and chronic health conditions in a rural population in Korea. J Korean Med Sci. 2008; 23(1): 1-9
Seo W-K, Koh S-B, Kim B-J, Yu S-W, Park M-H, Park K-W, Lee D-H. Prevalence of Parkinson's disease in Korea. J Clin Neurosci. 2007; 14(12): 1155-7
Statistics Korea (South Korea). South Korea KOSIS Database - Deaths and Death Rates by Sex and Age Group. Seoul, South Korea: Statistics Korea (South Korea)
Shin CS, Choi HJ, Kim MJ, Kim JT, Yu SH, Koo BK, Cho HY, Cho SW, Kim SW, Park YJ, Jang HC, Kim SY, Cho NH. Prevalence and risk factors of osteoporosis in Korea: a community-based cohort study with lumbar spine and hip bone mineral density. Bone. 2010; 47(2): 378-87
Yang JS, Kang SK, Park IJ, Rhee KY, Moon YH, Sohn DH. Lead concentrations in blood among the general population of Korea. Int Arch Occup Environ Health. 1996; 68(3): 199-202
Moon CS, Zhang ZW, Shimbo S, Watanabe T, Moon DH, Lee CU, Lee BK, Ahn KD, Lee SH, Ikeda M. Dietary intake of cadmium and lead among the general population in Korea. Environ Res. 1995; 71(1): 46-54
Lee R, Lee H, Yoo I, Kim S-R. Trend of blood lead levels in children in an industrial complex and its suburban area in Ulsan, Korea. Int Arch Occup Environ Health. 2002; 75(7): 507-10
Min K-B, Min J-Y, Cho S-I, Kim R, Kim H, Paek D. Relationship between low blood lead levels and growth in children of white-collar civil servants in Korea. Int J Hyg Environ Health. 2008; 211(1-2): 82-7
Rho Y-I, Chung H-J, Lee K-H, Eun B-L, Eun S-H, Nam S-O, Kim W-S, Kim Y-O, Park H-J, Kim H-S. Prevalence and clinical characteristics of primary headaches among school children in South Korea: a nationwide survey. Headache. 2012; 52(4): 592-9
Kim B-K, Chung YK, Kim J-M, Lee K-S, Chu MK. Prevalence, clinical characteristics and disability of migraine and probable migraine: a nationwide population-based survey in Korea. Cephalalgia. 2013; 33(13): 1106-16
Korea Centers for Disease Control and Prevention (KCDC). South Korea National Health and Nutrition Examination Survey 2010
Korea Centers for Disease Control and Prevention (KCDC). South Korea National Health and Nutrition Examination Survey 2012
World Health Organization (WHO). WHO Global Database on Child Growth and Malnutrition. Geneva, Switzerland: World Health Organization (WHO)
Kweon S, Kim Y, Jang MJ, Kim Y, Kim K, Choi S, Chun C, Khang YH, Oh K. Data resource profile: the Korea National Health and Nutrition Examination Survey (KNHANES). Int J Epidemiol. 2014; 43(1): 69-77 as it appears in World Health Organization (WHO). WHO Global Database on Child Growth and Malnutrition. Geneva, Switzerland: World Health Organization (WHO)
Choi S-H, Hong S-B, Ko G-B, Lee Y, Park HJ, Park S-Y, Moon SM, Cho O-H, Park K-H, Chong YP, Kim S-H, Huh JW, Sung H, Do K-H, Lee S-O, Kim M-N, Jeong J-Y, Lim C-M, Kim YS, Woo JH, Koh Y. Viral infection in patients with severe pneumonia requiring intensive care unit admission. Am J Respir Crit Care Med. 2012; 186(4): 325-32
Lee JE, Choe K-W, Lee SW. Clinical and radiological characteristics of 2009 H1N1 influenza associated pneumonia in young male adults. Yonsei Med J. 2013; 54(4): 927-34
World Health Organization (WHO). Global leprosy situation, 2004. Wkly Epidemiol Rec. 2005: 80(13): 118-24
World Health Organization (WHO). Global leprosy situation, 2005. Wkly Epidemiol Rec. 2005: 80(34): 289-95
World Health Organization (WHO). Global leprosy situation, 2006. Wkly Epidemiol Rec. 2006: 81(32): 309-16
World Health Organization (WHO). Global leprosy situation, 2007. Wkly Epidemiol Rec. 2007: 82(25): 225-32
World Health Organization (WHO). Global leprosy situation, 2009. Wkly Epidemiol Rec. 2009: 84(33): 333-40
World Health Organization (WHO). Global leprosy situation, 2010. Wkly Epidemiol Rec. 2010: 85(35): 337-48
World Health Organization (WHO). Global leprosy situation, 2012. Wkly Epidemiol Rec. 2012: 87(34): 317-28
World Health Organization (WHO). Global leprosy situation, beginning of 2008. Wkly Epidemiol Rec. 2008: 83(33): 293-300
World Health Organization (WHO). Global leprosy: update on the 2012 situation. Wkly Epidemiol Rec. 2013: 88(35): 365-79
World Health Organization (WHO). Leprosy update, 2011. Wkly Epidemiol Rec. 2011: 86(36): 389-99
World Health Organization (WHO). WHO Global Health Observatory - Cholera: Number of Reported Cases by Country. Geneva, Switzerland: World Health Organization (WHO)
Chung ML, Lee BS, Kim EA-R, Kim K-S, Pi S-Y, Oh YM, Park IS, Seo DM, Won HS. Impact of fetal echocardiography on trends in disease patterns and outcomes of congenital heart disease in a neonatal intensive care unit. Neonatology. 2010; 98(1): 41-6
Lee JE, Jung K-L, Kim S-E, Nam S-H, Choi S-J, Oh S-Y, Roh C-R, Kim J-H. Prenatal diagnosis of congenital heart disease: trends in pregnancy termination rate, and perinatal and 1-year infant mortalities in Korea between 1994 and 2005. J Obstet Gynaecol Res. 2010; 36(3): 474-8
Lim C, Lee JY, Kim W-H, Kim S-C, Song J-Y, Kim S-J, Choh J-H, Kim CW. Early replacement of pulmonary valve after repair of tetralogy: is it really beneficial?. Eur J Cardiothorac Surg. 2004; 25(5): 728-34
World Health Organization Regional Office for the Western Pacific (WPRO-WHO). Number of Reported Cases of Dengue Fever and Dengue Hemorrhagic Fever in the Western Pacific Region, by Country 2000. Manila, Philippines: World Health Organization Regional Office for the Western Pacific (WPRO-WHO)
World Health Organization Regional Office for the Western Pacific (WPRO-WHO). Number of Reported Cases of Dengue Fever and Dengue Hemorrhagic Fever in the Western Pacific Region, by Country 2001. Manila, Philippines: World Health Organization Regional Office for the Western Pacific (WPRO-WHO)

World Health Organization Regional Office for the Western Pacific (WPRO-WHO). Number of Reported Cases of Dengue Fever and Dengue Hemorrhagic Fever in the Western Pacific Region, by Country 2002. Manila, Philippines: World Health Organization Regional Office for the Western Pacific (WPRO-WHO)

World Health Organization Regional Office for the Western Pacific (WPRO-WHO). Number of Reported Cases of Dengue Fever and Dengue Hemorrhagic Fever in the Western Pacific Region, by Country 2003. Manila, Philippines: World Health Organization Regional Office for the Western Pacific (WPRO-WHO)

World Health Organization Regional Office for the Western Pacific (WPRO-WHO). Number of Reported Cases of Dengue Fever and Dengue Hemorrhagic Fever in the Western Pacific Region, by Country 2004. Manila, Philippines: World Health Organization Regional Office for the Western Pacific (WPRO-WHO)

World Health Organization Regional Office for the Western Pacific (WPRO-WHO). Number of Reported Cases of Dengue Fever and Dengue Hemorrhagic Fever in the Western Pacific Region, by Country 2005. Manila, Philippines: World Health Organization Regional Office for the Western Pacific (WPRO-WHO)

World Health Organization Regional Office for the Western Pacific (WPRO-WHO). Number of Reported Cases of Dengue Fever and Dengue Hemorrhagic Fever in the Western Pacific Region, by Country 2006. Manila, Philippines: World Health Organization Regional Office for the Western Pacific (WPRO-WHO)

World Health Organization Regional Office for the Western Pacific (WPRO-WHO). Number of Reported Cases of Dengue Fever and Dengue Hemorrhagic Fever in the Western Pacific Region, by Country 2007. Manila, Philippines: World Health Organization Regional Office for the Western Pacific (WPRO-WHO)

World Health Organization Regional Office for the Western Pacific (WPRO-WHO). Number of Reported Cases of Dengue Fever and Dengue Hemorrhagic Fever in the Western Pacific Region, by Country 2009. Manila, Philippines: World Health Organization Regional Office for the Western Pacific (WPRO-WHO)

World Health Organization Regional Office for the Western Pacific (WPRO-WHO). Number of Reported Cases of Dengue Fever and Dengue Hemorrhagic Fever in the Western Pacific Region, by Country 2010. Manila, Philippines: World Health Organization Regional Office for the Western Pacific (WPRO-WHO)

Jhoo JH, Kim KW, Huh Y, Lee SB, Park JH, Lee JJ, Choi EA, Han C, Choo IH, Youn JC, Lee DY, Woo JI. Prevalence of dementia and its subtypes in an elderly urban korean population: results from the Korean Longitudinal Study on Health And Aging (KLoSHA). Dement Geriatr Cogn Disord. 2008; 26(3): 270-6

Lee HR, Lee MJ, Hahm JH. Clinical Study and Acyclovir Therapy of Herpes Zoster. Korean J Dermatol. 1995; 33(4): 661–88

Kang C-I, Choi C-M, Park T-S, Lee D-J, Oh M, Choe K-W. Incidence of herpes zoster and seroprevalence of varicella-zoster virus in young adults of South Korea. Int J Infect Dis. 2008; 12(3): 245-7

Kim B-K, Chu MK, Lee TG, Kim J-M, Chung C-S, Lee K-S. Prevalence and impact of migraine and tension-type headache in Korea. J Clin Neurol. 2012; 8(3): 204-11

Sung Y, Kim D, Yoo S, Baik S, Oh J, Lee H. The prevalence and phenotypes of polycystic ovary syndrome in Korean women. Endocr Abstr. 2010; 22: 486

Kweon S-S, Shin M-H, Park K-S, Nam H-S, Jeong S-K, Ryu S-Y, Chung E-K, Choi J-S. Distribution of the ankle-brachial index and associated cardiovascular risk factors in a population of middle-aged and elderly koreans. J Korean Med Sci. 2005; 20(3): 373-8

United Nations Statistics Division (UNSD). United Nations Demographic Yearbook - Historical Supplement 1997. New York, United States of America: United Nations (UN)

South Korea Population and Housing Census 1970 as it appears in United Nations Statistics Division (UNSD). United Nations Demographic Yearbook - Historical Supplement 1997. New York, United States of America: United Nations (UN)

South Korea Population and Housing Census 1975 as it appears in United Nations Statistics Division (UNSD). United Nations Demographic Yearbook - Historical Supplement 1997. New York, United States of America: United Nations (UN)

South Korea Vital Registration Death Data 1977 as it appears in United Nations Statistics Division (UNSD). United Nations Demographic Yearbook - Historical Supplement 1997. New York, United States of America: United Nations (UN)

South Korea Vital Registration Death Data 1978 as it appears in United Nations Statistics Division (UNSD). United Nations Demographic Yearbook - Historical Supplement 1997. New York, United States of America: United Nations (UN)

South Korea Vital Registration Death Data 1979 as it appears in United Nations Statistics Division (UNSD). United Nations Demographic Yearbook - Historical Supplement 1997. New York, United States of America: United Nations (UN)

South Korea Vital Registration Death Data 1980 as it appears in United Nations Statistics Division (UNSD). United Nations Demographic Yearbook - Historical Supplement 1997. New York, United States of America: United Nations (UN)

South Korea Vital Registration Death Data 1981 as it appears in United Nations Statistics Division (UNSD). United Nations Demographic Yearbook - Historical Supplement 1997. New York, United States of America: United Nations (UN)

South Korea Vital Registration Death Data 1982 as it appears in United Nations Statistics Division (UNSD). United Nations Demographic Yearbook - Historical Supplement 1997. New York, United States of America: United Nations (UN)

South Korea Vital Registration Death Data 1957 as it appears in United Nations Statistics Division (UNSD). United Nations Demographic Yearbook - Historical Supplement 1997. New York, United States of America: United Nations (UN)

South Korea Vital Registration Death Data 1960 as it appears in United Nations Statistics Division (UNSD). United Nations Demographic Yearbook - Historical Supplement 1997. New York, United States of America: United Nations (UN)

South Korea Vital Registration Death Data 1962 as it appears in United Nations Statistics Division (UNSD). United Nations Demographic Yearbook - Historical Supplement 1997. New York, United States of America: United Nations (UN)

South Korea Vital Registration Death Data 1963 as it appears in United Nations Statistics Division (UNSD). United Nations Demographic Yearbook - Historical Supplement 1997. New York, United States of America: United Nations (UN)

South Korea Vital Registration Death Data 1964 as it appears in United Nations Statistics Division (UNSD). United Nations Demographic Yearbook - Historical Supplement 1997. New York, United States of America: United Nations (UN)

South Korea Vital Registration Death Data 1967 as it appears in United Nations Statistics Division (UNSD). United Nations Demographic Yearbook - Historical Supplement 1997. New York, United States of America: United Nations (UN)

United Nations Statistics Division (UNSD). United Nations Demographic Yearbook. New York City, United States of America: United Nations Statistics Division (UNSD)

South Korea Population and Housing Census 1949 as it appears in United Nations Statistics Division (UNSD). United Nations Demographic Yearbook. New York City, United States of America: United Nations Statistics Division (UNSD)

South Korea Population and Housing Census 1955 as it appears in United Nations Statistics Division (UNSD). United Nations Demographic Yearbook. New York City, United States of America: United Nations Statistics Division (UNSD)

South Korea Population and Housing Census 1960 as it appears in United Nations Statistics Division (UNSD). United Nations Demographic Yearbook. New York City, United States of America: United Nations Statistics Division (UNSD)
South Korea Population and Housing Census 1966 as it appears in United Nations Statistics Division (UNSD). United Nations Demographic Yearbook. New York City, United States of America: United Nations Statistics Division (UNSD)
South Korea Population and Housing Census 1995 as it appears in United Nations Statistics Division (UNSD). United Nations Demographic Yearbook. New York City, United States of America: United Nations Statistics Division (UNSD)
South Korea Population and Housing Census 2000 as it appears in United Nations Statistics Division (UNSD). United Nations Demographic Yearbook. New York City, United States of America: United Nations Statistics Division (UNSD)
South Korea Population and Housing Census 2005 as it appears in United Nations Statistics Division (UNSD). United Nations Demographic Yearbook. New York City, United States of America: United Nations Statistics Division (UNSD)
South Korea Population and Housing Census 2010 as it appears in United Nations Statistics Division (UNSD). United Nations Demographic Yearbook. New York City, United States of America: United Nations Statistics Division (UNSD)
South Korea Population and Housing Census 2015 as it appears in United Nations Statistics Division (UNSD). United Nations Demographic Yearbook. New York City, United States of America: United Nations Statistics Division (UNSD)
Park S, Oh S, Lee J, Park G, Choi S, Chae Y, Kim H. Genotypes of rotavirus associated with acute gastroenteritis in Seoul, Korea. Microbiol Immunol. 2011; 55(9): 641-4
Huh J-W, Kim W-H, Yoon M-H, Lim Y-H. Genotypic distribution of rotavirus strains causing severe gastroenteritis in Gyeonggi province, South Korea, from 2003 to 2005. Arch Virol. 2009; 154(1): 167-70
Kang JO, Kim CR, Kilgore PE, Choi TY. G and P genotyping of human rotavirus isolated in a university hospital in Korea: implications for nosocomial infections. J Korean Med Sci. 2006; 21(6): 983-8
Kim JS, Kang JO, Cho SC, Jang YT, Min SA, Park TH, Nyambat B, Jo DS, Gentsch J, Bresee JS, Mast TC, Kilgore PE. Epidemiological profile of rotavirus infection in the South Korea: results from prospective surveillance in the Jeongeub District, 1 July 2002 through 30 June 2004. J Infect Dis. 2005; S49-56
Le VP, Kim J-Y, Cho S-L, Nam S-W, Lim I, Lee H-J, Kim K, Chung S-I, Song W, Lee KM, Rhee M-S, Lee J-S, Kim W. Detection of unusual rotavirus genotypes G8P[8] and G12P[6] in South Korea. J Med Virol. 2008; 80(1): 175-82
Moon S-S, Green YS, Song J-W, Ahn CN, Kim H, Park KS, Song K-J, Lee JH, Baek LJ. Genetic distribution of group A human rotavirus types isolated in Gyunggi province of Korea, 1999-2002. J Clin Virol. 2007; 38(1): 57-63
Song M-O, Kim K-J, Chung S-I, Lim I, Kang S-Y, An C-N, Kim W. Distribution of human group a rotavirus VP7 and VP4 types circulating in Seoul, Korea between 1998 and 2000. J Med Virol. 2003; 70(2): 324-8
Chang K, Kim C, Oh S, Lee H, Lee K. A clinical and epidemiological study on rotavirus gastroenteritis in children. J Korean Pediatr Soc. 1988; 961-76
Bae GY, Oh ET, Jung WS, Kim SS. A Study of Serum Transaminase Level and It's Correlation with Several Symptoms in Children with HRV Gastroenteritis. J Korean Pediatr Soc. 1993; 36(8): 1146-55
Han S, Seo S, Min K, Kim J, Kim K, Ryoo K. Clinical observations on human rotavirus (HRV) gastroenteritis. J Korean Pediatr Soc. 1992; 226-33
Hong G, Jeon H, Choi W, Namgoong M, Kim J. A study of relation between serum aminotransferase elevation and clinical symptoms from human rotavirus gastroenteritis. J Korean Pediatr Soc. 1995; 54-60
Kim D, Park B, Jung D, Ahn J, Kim C, Kang S. Prevalence and identification of rotaviruses in stool specimens of patients with acute diarrhea from several regions of Korea. J Korean Pediatr Soc. 1999; 501-9
Chung GE, Yim JY, Kim D, Lim SH, Park MJ, Kim YS, Yang SY, Yang JI, Cho S-H. Seroprevalence of hepatitis a and associated socioeconomic factors in young healthy korean adults. Gut Liver. 2011; 5(1): 88-92
Yun H, Lee H-J, Cheon D, Chu C, Oh KW, Kim YT, Jee Y. Seroprevalence of Hepatitis A and E Viruses Based on the Third Korea National Health and Nutrition Survey in Korea. Osong Public Health Res Perspect. 2011; 2(1): 46-50
Jones DW, Kim JS, Andrew ME, Kim SJ, Hong YP. Body mass index and blood pressure in Korean men and women: the Korean National Blood Pressure Survey. J Hypertens. 1994; 12(12): 1433-7
Lee K, Lee S, Kim SY, Kim SJ, Kim YJ. Percent body fat cutoff values for classifying overweight and obesity recommended by the International Obesity Task Force (IOTF) in Korean children. Asia Pac J Clin Nutr. 2007; 16(4): 649-55
Yoo S, Lee S-Y, Kim K-N, Sung E. Obesity in Korean pre-adolescent school children: comparison of various anthropometric measurements based on bioelectrical impedance analysis. Int J Obes (Lond). 2006; 30(7): 1086-90
Chang Y, Woo H-Y, Sung E, Kim CH, Kang H, Ju YS, Park KH. Prevalence of acanthosis nigricans in relation to anthropometric measures: community-based cross-sectional study in Korean pre-adolescent school children. Pediatr Int. 2008; 50(5): 667-73
Lee K, Song Y-M. Parent-reported appetite of a child and the child's weight status over a 2-year period in Korean children. J Am Diet Assoc. 2007; 107(4): 678-80
ISSP Research Group (2009): International Social Survey Programme: Health and Health Care - ISSP 2011. GESIS Data Archive, Cologne. ZA5800 Data file version 3.0.0, doi:10.4232/1.12252
ISSP Research Group (2009): International Social Survey Programme: Leisure Time and Sports - ISSP 2007. GESIS Data Archive, Cologne. ZA4850 Data file version 2.0.0, doi:10.4231/1.10079
Yang S-K, Hong W-S, Min YI, Kim HY, Yoo JY, Rhee P-L, Rhee JC, Chang DK, Song IS, Jung SA, Park E-B, Yoo HM, Lee DK, Kim YK. Incidence and prevalence of ulcerative colitis in the Songpa-Kangdong District, Seoul, Korea, 1986-1997. J Gastroenterol Hepatol. 2000; 15(9): 1037-42
Choi CW, Lee J, Park KH, Yoon SY, Choi IK, Oh SC, Seo JH, Kim BS, Shin SW, Kim YH, Kim JS. Prevalence and characteristics of anemia in the elderly: cross-sectional study of three urban Korean population samples. Am J Hematol. 2004; 77(1): 26-30
Choi JW, Kim CS, Pai SH. Erythropoietic activity and soluble transferrin receptor level in neonates and maternal blood. Acta Paediatr. 2000; 89(6): 675-9
South Korea National Nutrition Survey 1993 as it appears in World Health Organization (WHO). WHO Global Database on Anemia, Nutrition Landscape Information System. Geneva, Switzerland: World Health Organization (WHO)
South Korea National Nutrition Survey 1994 as it appears in World Health Organization (WHO). WHO Global Database on Anemia, Nutrition Landscape Information System. Geneva, Switzerland: World Health Organization (WHO)
South Korea National Nutrition Survey 1995 as it appears in World Health Organization (WHO). WHO Global Database on Anemia, Nutrition Landscape Information System. Geneva, Switzerland: World Health Organization (WHO)
Chitsulo L, Engels D, Montresor A, Savioli L. The global status of schistosomiasis and its control. Acta Trop. 2000; 77(1): 41-51

Lee SS, Jeong S-H, Byoun Y-S, Chung SM, Seong MH, Sohn HR, Min B-Y, Jang ES, Kim J-W, Park GJ, Lee YJ, Lee KH, Ahn S. Clinical features and outcome of cryptogenic hepatocellular carcinoma compared to those of viral and alcoholic hepatocellular carcinoma. BMC Cancer. 2013; 335
International Labour Organization (ILO). International Labour Organization Database (ILOSTAT) - Employment by Sex and Economic Activity. International Labour Organization (ILO)
International Labour Organization (ILO). International Labour Organization Database (ILOSTAT) - Employment by Sex and Occupation. International Labour Organization (ILO)
Choi I-S, Kwon H-J, Shin N-R, Yoo HS. Identification of swine hepatitis E virus (HEV) and prevalence of anti-HEV antibodies in swine and human populations in Korea. J Clin Microbiol. 2003; 41(8): 3602-8
Yoon YS, Oh SW, Baik HW, Park HS, Kim WY. Alcohol consumption and the metabolic syndrome in Korean adults: the 1998 Korean National Health and Nutrition Examination Survey. Am J Clin Nutr. 2004; 80(1): 217-24
Park Y, Lee H, Koh CS, Min H. Community-based epidemiologic study on atherosclerotic cardiovascular risk factors. Diabetes Res Clin Pract. 1996; S65-72
Kim JM. Vascular disease/risk and late-life depression in a Korean community population. Br J Psychiatry. 2004; 185(2): 102-7
Park C-H, Cho Y-K, Park J-H, Jun J-S, Park E-S, Seo J-H, Lim J-Y, Woo H-O, Youn H-S, Ko G-H, Kang H-L, Baik S-C, Lee W-K, Cho M-J, Rhee K-H. Changes in the age-specific prevalence of hepatitis A virus antibodies: a 10-year cohort study in Jinju, South Korea. Clin Infect Dis. 2006; 42(8): 1148-50
Sohn YM, Rho HO, Park MS, Park JH, Choi BY, Ki M, Jang WI. The changing epidemiology of hepatitis A in children and the consideration of active immunization in Korea. Yonsei Med J. 2000; 41(1): 34-9
Kim TY, Sohn JH, Ahn SB, Son BK, Lee HL, Eun CS, Jeon CS, Han DS. Comparison of Recent IgG Anti-HAV Prevalence between Two Hospitals in Seoul and Gyeonggi area. Korean J Hepatol. 2007; 13(3): 363
Song YB, Lee JH, Choi MS, Koh KC, Paik SW, Yoo BC, Choi Y-H, Sohn HJ, Lee KH, Rhee JC. [The age-specific seroprevalence of hepatitis A virus antibody in Korea]. Korean J Hepatol. 2007; 13(1): 27-33
Ahn J-M, Kang S-G, Lee D-Y, Shin SJ, Yoo HS. Identification of novel human hepatitis E virus (HEV) isolates and determination of the seroprevalence of HEV in Korea. J Clin Microbiol. 2005; 43(7): 3042-8
Jang MK, Lee JY, Lee JH, Kim YB, Kim HY, Lee MS, Park CK, Yoo JY. Seroepidemiology of HBV infection in South Korea, 1995 through 1999. Korean J Intern Med. 2001; 16(3): 153-9
Shin H-R, Hwang SY, Nam C-M. The Prevalence of Hepatitis C Virus Infection in Korea: Pooled Analysis. J Korean Med Sci. 2005; 20(6): 985-8
Shin HR, Kim JY, Kim JI, Lee DH, Yoo KY, Lee DS, Franceschi S. Hepatitis B and C virus prevalence in a rural area of South Korea: the role of acupuncture. Br J Cancer. 2002; 87(3): 314-8
Cho YH, Kim HO, Lee JB, Lee MG. Syphilis prevalence has rapidly decreased in South Korea. Sex Transm Infect. 2003; 79(4): 323-4
Kim C-K, Lee S-C, Lee D-M, Chang B-U, Rho B-H, Kang H-D. Nationwide survey of radon levels in Korea. Health Phys. 2003; 84(3): 354-60
Kim Y, Chang B-U, Park H-M, Kim C-K, Tokonami S. National radon survey in Korea. Radiat Prot Dosimetry. 2011; 146(1-3): 6-10
Chai J-Y, Lee S-H, Choi S-Y, Lee J-S, Yong T-S, Park K-J, Yang K-A, Lee K-H, Park M-J, Park H-R, Kim M-J, Rim H-J. A survey of Brugia malayi infection on the Heugsan Islands, Korea. Korean J Parasitol. 2003; 41(1): 69-73
Cheun H-I, Kong Y, Cho S-H, Lee J-S, Chai J-Y, Lee J-S, Lee J-K, Kim T-S. Successful control of lymphatic filariasis in the South Korea. Korean J Parasitol. 2009; 47(4): 323-35
Korea Association of Health. Collected Papers on Parasite Control in Korea. Seoul, South Korea: Korea Association of Health, 1994
Food and Agriculture Organization of the United Nations (FAO), World Health Organization (WHO). Joint WHO/FAO Workshop on Foodborne Trematode Infections in Asia. Manila, Philippines: World Health Organization Regional Office for the Western Pacific (WPRO-WHO), 2004
Cho SY, Kang SY, Lee JB. Metagonimiasis in Korea. Arzneimittelforschung. 1984; 34(9B): 1211-3
Korea Centers for Disease Control and Prevention (KCDC), Korea National Institute of Health, Ministry of Health and Welfare (South Korea). South Korea National Survey of the Prevalence of Intestinal Parasitic Infections 2004
Catholic University of Leuven, Organization for Economic Co-operation and Development (OECD), United Nations Population Division. Mortality in Developing Countries: Tome I Data Bank. Paris, France: Organization for Economic Co-operation and Development (OECD), 1980
Pew Research Center. The Future of the Global Muslim Population. Washington, DC, United States: Pew Research Center, 2011
South Korea Vital Registration - Deaths 2012 ICD10
Korea, South Household Cooking Fuels Data 1983
Pullan RL, Smith JL, Jasrasaria R, Brooker SJ. Global numbers of infection and disease burden of soil transmitted helminth infections in 2010 [Unpublished data]. Parasit Vectors. 2014; 7(37). [Unpublished data as provided by the Global Burden of Disease 2010 soil transmitted helminths expert group]
Graduate School of Public Health, Seoul National University, World Health Organization (WHO). South Korea WHO Multi-country Survey Study on Health and Health System Responsiveness 2000-2001. Geneva, Switzerland: World Health Organization (WHO)
Park BC, Han BH, Ahn SY, Lee SW, Lee DH, Lee YN, Seo JH, Kim KW. Prevalence of hepatitis C antibody in patients with chronic liver disease and hepatocellular carcinoma in Korea. J Viral Hepat. 1995; 2(4): 195-202
Shin S-Y, Kwon M-J, Park H, Woo H-Y. Comparison of chronic kidney disease prevalence examined by the chronic kidney disease epidemiology collaboration equation with that by the modification of diet in renal disease equation in Korean adult population. J Clin Lab Anal. 2014; 28(4): 320–7
Kang YU, Kim HY, Choi JS, Kim CS, Bae EH, Ma SK, Kim SW. Metabolic syndrome and chronic kidney disease in an adult Korean population: results from the Korean National Health Screening. PLoS One. 2014; 9(5): e93795
Korea Central Cancer Registry, Ministry of Health and Welfare (South Korea), National Cancer Center (South Korea). South Korea - Annual Report of Cancer Statistics in Korea in 2012. Goyang-si, South Korea: National Cancer Center (South Korea), 2014

Kim do Y, Kim IH, Jeong SH, Cho YK, Lee JH, Jin YJ, Lee D, Suh DJ, Han KH, Park NH, Kang HY, Jung YK, Kim YS, Kim KA, Lee YJ, Lee BS, Yim HJ, Lee HJ, Baik SK, Tak WY, Lee SJ, Chung WJ, Choi SK, Cho EY, Heo J, Kim DJ, Song BC, Kim MW, Lee J, Chae HB, Choi DH, Choi HY, Ki M. A nationwide seroepidemiology of hepatitis C virus infection in South Korea. Liver Int. 2013; 33.0(4): 586-94
World Health Organization Regional Office for the Western Pacific (WPRO-WHO). WPRO-WHO Health Information and Intelligence Platform - Antenatal Care Coverage - At Least 1 Visit. Manila, Philippines: World Health Organization Regional Office for the Western Pacific (WPRO-WHO)
Rim TH, Nam JS, Choi M, Lee SC, Lee CS. Prevalence and risk factors of visual impairment and blindness in Korea: the Fourth Korea National Health and Nutrition Examination Survey in 2008-2010. Acta Ophthalmol. 2014; 92(4.0): 317-25
Park SH, Lee JS, Heo H, Suh YW, Kim SH, Lim KH, Moon NJ, Lee SJ, Park SH, Baek SH. A nationwide population-based study of low vision and blindness in South Korea. Invest Ophthalmol Vis Sci. 2015; 56(1.0): 484-93
Yim SY, Yu HH, Lee IY. The prevalence of mental retardation among third grade elementary school children in the Suwon area, Korea. J Korean Med Sci. 2002; 17(1): 86-90
Cho MJ, Chang SM, Lee YM, Bae A, Ahn JH, Son J, Hong JP, Bae JN, Lee DW, Cho SJ, Park JI, Lee JY, Kim JY, Jeon HJ, Sohn JH, Kim BS. Prevalence of DSM-IV major mental disorders among Korean adults: A 2006 National Epidemiologic Survey (KECA-R). Asian J Psychiatr. 2010; 3(1): 26–30
Han TH, Park SH, Hwang ES, Reuter G, Chung JY. Detection of Aichi virus in South Korea. Arch Virol. 2014; 159(7): 1835-9
Aartun E, Hartvigsen J, Wedderkopp N, Hestbaek L. Spinal pain in adolescents: prevalence, incidence, and course: a school-based two-year prospective cohort study in 1,300 Danes aged 11-13. BMC Musculoskelet Disord. 2014; 187
Shin DH, Sinn DH, Kim Y-H, Kim JY, Chang DK, Kim EJ, Ryu HY, Song HU, Kim IY, Kim DH, Kim YY, Kim SH, Seo YB, Hwang KW, Kim JJ. Increasing incidence of inflammatory bowel disease among young men in Korea between 2003 and 2008. Dig Dis Sci. 2011; 56(4): 1154–9
Kim JK, Jeon JS, Kim JW, Rheem I. Epidemiology of respiratory viral infection using multiplex rt-PCR in Cheonan, Korea (2006-2010). J Microbiol Biotechnol. 2013; 23(2): 267-73
Cho HJ, Shim SY, Son DW, Sun YH, Tchah H, Jeon IS. Respiratory viruses in neonates hospitalized with acute lower respiratory tract infections. Pediatr Int. 2013; 55(1): 49-53
Hoo Lee J, Hyung Kim Y. Comparison of clinical characteristics between healthcare-associated pneumonia and community-acquired pneumonia in patients admitted to secondary hospitals. Braz J Infect Dis. 2012; 16(4): 321-8
Kim Y, Kim J, Lee K-A. Prevalence of sexually transmitted infections among healthy Korean women: implications of multiplex PCR pathogen detection on antibiotic therapy. J Infect Chemother. 2014; 20(1): 74–6
Choi JY, Cho I-C, Lee GI, Min SK. Prevalence and associated factors for four sexually transmissible microorganisms in middle-aged men receiving general prostate health checkups: a polymerase chain reaction-based study in Korea. Korean J Urol. 2013; 54(1): 53–8
Kim ID, Chang HS, Hwang KJ. Herpes simplex virus 2 infection rate and necessity of screening during pregnancy: a clinical and seroepidemiologic study. Yonsei Med J. 2012; 53(2): 401–7
Shin HS, Park JJ, Chu C, Song HJ, Cho KS, Lee JS, Kim SS, Kee MK. Herpes simplex virus type 2 seroprevalence in Korea: rapid increase of HSV-2 seroprevalence in the 30s in the southern part. J Korean Med Sci. 2007; 22(6): 957–62
Vaccarella S, Franceschi S, Herrero R, Muñoz N, Snijders PJF, Clifford GM, Smith JS, Lazcano-Ponce E, Sukvirach S, Shin H-R, de Sanjosé S, Molano M, Matos E, Ferreccio C, Anh PTH, Thomas JO, Meijer CJLM, IARC HPV Prevalence Surveys Study Group. Sexual behavior, condom use, and human papillomavirus: pooled analysis of the IARC human papillomavirus prevalence surveys. Cancer Epidemiol Biomarkers Prev. 2006; 15(2): 326–33
Kim BH, Park JW, Nam BH, Kwak HW, Kim WR. Validation of a model to estimate survival in ambulatory patients with hepatocellular carcinoma: a single-centre cohort study. Liver Int. 2014; 34(7): e317-23
Park S, Kim SY, Hong JP. Cause-specific mortality of psychiatric inpatients and outpatients in a general hospital in Korea. Asia Pac J Public Health. 2015; 27(2): 164–75
Son KM, Cho NH, Lim SH, Kim HA. Prevalence and risk factor of neck pain in elderly Korean community residents. J Korean Med Sci. 2013; 28(5): 680–6
Kim SS, Won JC, Kwon HS, Kim CH, Lee JH, Park TS, Ko KS, Cha BY. Prevalence and clinical implications of painful diabetic peripheral neuropathy in type 2 diabetes: results from a nationwide hospital-based study of diabetic neuropathy in Korea. Diabetes Res Clin Pract. 2014; 103(3): 522–9
Won JC, Kwon HS, Kim CH, Lee JH, Park TS, Ko KS, Cha BY. Prevalence and clinical characteristics of diabetic peripheral neuropathy in hospital patients with Type 2 diabetes in Korea. Diabet Med. 2012; 29(9): e290–6
Oh J-W, Pyun B-Y, Choung J-T, Ahn K, Kim C-H, Song S-W, Son J-A, Lee S, Lee S-I. Epidemiological change of atopic dermatitis and food allergy in school-aged children in Korea between 1995 and 2000. J Korean Med Sci. 2004; 19(5): 716–23
Cho MJ, Nam JJ, Suh GH. Prevalence of symptoms of depression in a nationwide sample of Korean adults. Psychiatry Res. 1998; 81(3): 341–52
Cho MJ, Seong SJ, Park JE, Chung IW, Lee YM, Bae A, Ahn JH, Lee DW, Bae JN, Cho SJ, Park JI, Son J, Chang SM, Hahm BJ, Lee JY, Sohn JH, Kim JS, Hong JP. Prevalence and Correlates of DSM-IV Mental Disorders in South Korean Adults: The Korean Epidemiologic Catchment Area Study 2011. Psychiatry Investig. 2015; 12(2): 164–70
Oh DH, Kim SA, Lee HY, Seo JY, Choi BY, Nam JH. Prevalence and correlates of depressive symptoms in korean adults: results of a 2009 korean community health survey. J Korean Med Sci. 2013; 28(1): 128–35
Jeong HG, Lee JJ, Lee SB, Park JH, Huh Y, Han JW, Kim TH, Chin HJ, Kim KW. Role of severity and gender in the association between late-life depression and all-cause mortality. Int Psychogeriatr. 2013; 25(4): 677–84
Lee HK. Epidemiology of fetal alcohol syndrome in Korea. Alcohol Clin Exp Res. 2012; 36(Spplement s2): 34A
Cho NH. Diabetes burden and prevention in Korea and the Western Pacific Region. Diabetes Res Clin Pract. 2014; S282-7
Kim SG, Yang SW, Jang AS, Seo JP, Han SW, Yeom CH, Kim YC, Oh SH, Kim JS, Nam HS, Chung DJ, Chung MY. Prevalence of diabetes mellitus in the elderly of Namwon County, South Korea. Korean J Intern Med. 2002; 17(3): 180-90
Han SY, Cho MJ, Won S, Hong JP, Bae JN, Cho S-J, Park J-I, Lee J-Y, Jeon HJ, Chang SM. Sociodemographic Factors and Comorbidities Associated with Remission from Alcohol Dependence: Results from a Nationwide General Population Survey in Korea. Psychiatry Investig. 2015; 12(3): 316–23
South Korea National Vaccine Coverage Survey 2008-2009

Hwang Y, Kim CH, Kang HR, Shin T, Park SM, Jang SH, Park YB, Kim CH, Kim DG, Lee MG, Hyun IG, Jung KS. Comparison of the prevalence of chronic obstructive pulmonary disease diagnosed by lower limit of normal and fixed ratio criteria. J Korean Med Sci. 2009; 24(4): 621-626
Chung SJ, Kim TY, Kim SM, Roh M, Yu MY, Lee JH, Oh C, Lee EY, Lee S, Jeon YC, Yoo K-S, Sohn JH. Changes in the seroprevalence of IgG anti-hepatitis A virus between 2001 and 2013: experience at a single center in Korea. Clin Mol Hepatol. 2014; 20(2): 162–7
South Korea Vital Registration - Deaths 2013 ICD10
United Nations Statistics Division (UNSD). United Nations Demographic Yearbook 2013. New York, United States of America: United Nations (UN), 2014
United Nations Statistics Division (UNSD). United Nations Demographic Yearbook 2014. New York, United States of America: United Nations (UN), 2015
World Health Organization (WHO). WHO Global Health Observatory - Recorded Alcohol Per Capita Consumption 1960-1979. Geneva, Switzerland: World Health Organization (WHO)
World Health Organization (WHO). WHO Global Health Observatory - Recorded Alcohol Per Capita Consumption 1980-1999. Geneva, Switzerland: World Health Organization (WHO)
World Health Organization (WHO). WHO Global Health Observatory - Recorded Alcohol Per Capita Consumption 2000-2009 by country. Geneva, Switzerland: World Health Organization (WHO)
World Health Organization (WHO). Global leprosy update, 2013; reducing disease burden. Wkly Epidemiol Rec. 2014; 89(36): 389-400
World Health Organization (WHO). Global leprosy update, 2014: need for early case detection. Wkly Epidemiol Rec. 2015; 90(36): 461–74
FAO Supply Utilization Accounts 1961-2013. Personal Correspondence with Dr. Josef Schmidhuber, 2016. [Unpublished]
World Bank. World Development Indicators - Hospital Beds (per 1,000 People). Washington DC, United States of America: World Bank
Korea Institute for Health and Social Affairs (KIHASA). South Korea National Fertility and Family Health Survey 2009
Korea Institute for Health and Social Affairs (KIHASA). South Korea National Fertility and Family Health Survey 2012
International Road Federation. World Road Statistics 2015. Geneva, Switzerland: International Road Federation, 2015
Joint United Nations Program on HIV/AIDS (UNAIDS), Korea Centers for Disease Control and Prevention (KCDC). South Korea HIV/AIDS Control 2011
Vedamurthy I, Nahum M, Bavelier D, Levi DM. Mechanisms of recovery of visual function in adult amblyopia through a tailored action video game. Sci Rep. 2015; 5: 8482
Yoon K, Jang S-N, Chun H, Cho S-I. Self-reported anthropometric information cannot vouch for the accurate assessment of obesity prevalence in populations of middle-aged and older Korean individuals. Arch Gerontol Geriatr. 2014; 59(3): 584–92
Lee MY, Kim MY, Kim SY, Kim JH, Kim BH, Shin JY, Shin YG, Yun JH, Ryu SY, Lee TY, Koh SB, Chung CH. Association between alcohol intake amount and prevalence of metabolic syndrome in Korean rural male population. Diabetes Res Clin Pract. 2010; 88(2): 196–202
Korean Society of Nephrology. Current Renal Replacement Therapy in Korea: Insan Memorial Dialysis Registry 2015. Seoul, South Korea: Korean Society of Nephrology, 2015
U.S. Department of Agriculture (USDA). USDA Global Tobacco Database 1960-2005. Washington D.C. , United States: U.S. Department of Agriculture (USDA)
Food and Agriculture Organization of the United Nations (FAO). FAOSTAT Commodity Balances - Crops Primary Equivalent. Rome, Italy: Food and Agriculture Organization of the United Nations (FAO)
Gombojav B, Yi S-W, Sull JW, Nam CM, Ohrr H. Combined effects of cognitive impairment and hypertension on total mortality in elderly people: the Kangwha Cohort study. Gerontology. 2011; 57(6): 490–6
Kim YC, Koo HS, Kim S, Chin HJ. Estimation of daily salt intake through a 24-hour urine collection in Pohang, Korea. J Korean Med Sci. 2014; 29 Suppl 2: S87–90
Lim N-K, Park S-H, Choi S-J, Lee K-S, Park H-Y. A risk score for predicting the incidence of type 2 diabetes in a middle-aged Korean cohort: the Korean genome and epidemiology study. Circ J. 2012; 76(8): 1904–10
Lee H-S, Park Y-M, Kwon H-S, Lee J-H, Park YJ, Lim SY, Lee S-H, Yoon K-H, Son H-Y, Kim DS, Yim HW, Lee W-C. Prevalence, awareness, treatment, and control of hypertension among people over 40 years old in a rural area of South Korea: The Chungju Metabolic Disease Cohort (CMC) Study. Clin Exp Hypertens. 2010; 32(3): 166–78
Hwang JH, Kam S, Shin J, Kim J-Y, Lee K-E, Kwon G-H, Chun B-Y, Chae SC, Yang DH, Park HS, Hwang T-Y. Incidence of metabolic syndrome and relative importance of five components as a predictor of metabolic syndrome: 5-year follow-up study in Korea. J Korean Med Sci. 2013; 28(12): 1768–73
Lee JH, Yang DH, Park HS, Cho Y, Jun JE, Park WH, Chun BY, Shin J-Y, Shin DH, Lee KS, Kim K-S, Kim K-B, Kim YJ, Chae SC, HYpertension-Diabetes Daegu Initiative Study Investigators. Incidence of hypertension in Korea: 5-year follow-up study. J Korean Med Sci. 2011; 26(10): 1286–92
Moon H-W, Park CM, Hong SN, Park S, Hur M, Yun Y-M. Assessment of apoB dyslipoproteinemia in Korean population. Clin Biochem. 2013; 46(12): 1041–6
Shin CY, Yun KE, Park HS. Blood pressure has a greater impact on cardiovascular mortality than other components of metabolic syndrome in Koreans. Atherosclerosis. 2009; 205(2): 614–9
Cystic Echinococcosis Endemicity Estimates identified through systematic review and personal communication, as provided by GBD 2015 expert. [Unpublished]
World Health Organization (WHO). Global leprosy update, 2015: time for action, accountability and inclusion. Wkly Epidemiol Rec. 2016; 92(35): 405–20
Korea Employment Information Service (KEIS), TNS Korea. Korean Longitudinal Study of Ageing 2010, Wave 3. Seoul, South Korea: Korea Employment Information Service (KEIS)
World Health Organization (WHO). WHO Mortality Database Version September 2016. Geneva, Switzerland: World Health Organization (WHO)
South Korea Vital Registration - Deaths 1985 ICD9 as it appears in World Health Organization (WHO). WHO Mortality Database Version September 2016. Geneva, Switzerland: World Health Organization (WHO)

South Korea Vital Registration - Deaths 1986 ICD9 as it appears in World Health Organization (WHO). WHO Mortality Database Version September 2016. Geneva, Switzerland: World Health Organization (WHO)
South Korea Vital Registration - Deaths 1987 ICD9 as it appears in World Health Organization (WHO). WHO Mortality Database Version September 2016. Geneva, Switzerland: World Health Organization (WHO)
South Korea Vital Registration - Deaths 1988 ICD9 as it appears in World Health Organization (WHO). WHO Mortality Database Version September 2016. Geneva, Switzerland: World Health Organization (WHO)
South Korea Vital Registration - Deaths 1989 ICD9 as it appears in World Health Organization (WHO). WHO Mortality Database Version September 2016. Geneva, Switzerland: World Health Organization (WHO)
South Korea Vital Registration - Deaths 1990 ICD9 as it appears in World Health Organization (WHO). WHO Mortality Database Version September 2016. Geneva, Switzerland: World Health Organization (WHO)
South Korea Vital Registration - Deaths 1991 ICD9 as it appears in World Health Organization (WHO). WHO Mortality Database Version September 2016. Geneva, Switzerland: World Health Organization (WHO)
South Korea Vital Registration - Deaths 1992 ICD9 as it appears in World Health Organization (WHO). WHO Mortality Database Version September 2016. Geneva, Switzerland: World Health Organization (WHO)
South Korea Vital Registration - Deaths 1993 ICD9 as it appears in World Health Organization (WHO). WHO Mortality Database Version September 2016. Geneva, Switzerland: World Health Organization (WHO)
South Korea Vital Registration - Deaths 1994 ICD9 as it appears in World Health Organization (WHO). WHO Mortality Database Version September 2016. Geneva, Switzerland: World Health Organization (WHO)
Choi SH, Huh JW, Hong SB, Lee JY, Kim SH, Sung H, Do KH, Lee SO, Kim MN, Jeong JY, Lim CM, Kim YS, Woo JH, Koh Y. Clinical characteristics and outcomes of severe rhinovirus-associated pneumonia identified by bronchoscopic bronchoalveolar lavage in adults: comparison with severe influenza virus-associated pneumonia. J Clin Virol. 2015; 62: 41-7
Song JY, Lee JS, Wie SH, Kim HY, Lee J, Seo YB, Jeong HW, Kim SW, Lee SH, Park KH, Noh JY, Choi WS, Cheong HJ, Kim WJ. Prospective cohort study on the effectiveness of influenza and pneumococcal vaccines in preventing pneumonia development and hospitalization. Clin Vaccine Immunol. 2015; 22(2): 229-34
Max Planck Institute for Demographic Research, Vienna Institute of Demography. Human Fertility Collection - ASFR, original age scale. Rostock, Germany: Max Planck Institute for Demographic Research
United Nations Population Division. World Abortion Policies 2007. New York, United States of America: United Nations (UN), 2007
United Nations Population Division. World Abortion Policies 2013. New York, United States of America: United Nations (UN), 2013
Garcia-Marcos L, Robertson CF, Ross Anderson H, Ellwood P, Williams HC, Wong GW. Does migration affect asthma, rhinoconjunctivitis and eczema prevalence? Global findings from the international study of asthma and allergies in childhood. Int J Epidemiol. 2014; 43(6): 1846-54
Kim DW, Lee SY, Chung SE, Cheong HK, Jung KY. Clinical characteristics of patients with treated epilepsy in Korea: a nationwide epidemiologic study. Epilepsia. 2014; 55(1): 67-75
Lee JK, Song HJ, Yu JR. Prevalence of diarrhea caused by Cryptosporidium parvum in non-HIV patients in Jeollanam-do, Korea. Korean J Parasitol. 2005; 43(3): 111–4
Kang YH, Jeong DW, Son SM. Prevalence of Reduced Kidney Function by Estimated Glomerular Filtration Rate Using an Equation Based on Creatinine and Cystatin C in Metabolic Syndrome and Its Components in Korean Adults. Endocrinol Metab (Seoul). 2016; 31(3): 446-453
Lee SC, Bae JM, Lee HJ, Kim HJ, Kim BS, Li K, Cho JW, Park CO, Cho SH, Lee KH, Kim DW, Park CW, Kim KH. Introduction of the Reliable Estimation of Atopic Dermatitis in ChildHood: Novel, Diagnostic Criteria for Childhood Atopic Dermatitis. Allergy Asthma Immunol Res. 2016; 8(3): 230-8
Yang HK, Choi J, Kim WK, Lee SY, Park YM, Han MY, Kim HY, Hahm MI, Chae Y, Lee KJ, Kwon HJ, Ahn K, Kim J. The association between hypovitaminosis D and pediatric allergic diseases: A Korean nationwide population-based study. Allergy Asthma Proc. 2016; 37(4): 64-9
Park SY, Kwon HH, Min S, Yoon JY, Suh DH. Epidemiology and risk factors of childhood acne in Korea: a cross-sectional community based study. Clin Exp Dermatol. 2015; 40(8): 844-50
Chul Kim S, Kyoung Kwon S, Pyo Hong Y. Trends in the incidence of cryptorchidism and hypospadias of registry-based data in Korea: a comparison between industrialized areas of petrochemical estates and a non-industrialized area. Asian J Androl. 2011; 13(5): 715-8
Seo S, Chun S, Newell MA, Yun M. Association between alcohol consumption and Korean young women's bone health: a cross sectional study from the 2008 to 2011 Korea National Health and Nutrition Examination Survey. BMJ Open. 2015; 5(10): e007914
Hong S-W, Linton JA, Shim J-Y, Kang H-T. High-risk drinking is associated with a higher risk of diabetes mellitus in Korean men, based on the 2010-2012 KNHANES. Alcohol. 2015; 49(3): 275–81
Kim M, Choi KH, Hwang SW, Lee YB, Park HJ, Bae JM. Inflammatory bowel disease is associated with an increased risk of inflammatory skin diseases: A population-based cross-sectional study. J Am Acad Dermatol. 2016; nan
Choi Y, Park E-C, Kim J-H, Yoo K-B, Choi J-W, Lee K-S. A change in social activity and depression among Koreans aged 45 years and more: analysis of the Korean Longitudinal Study of Aging (2006-2010). Int Psychogeriatr. 2015; 27(4): 629–37
Sohn JH, Ahn SH, Seong SJ, Ryu JM, Cho MJ. Prevalence, work-loss days and quality of life of community dwelling subjects with depressive symptoms. J Korean Med Sci. 2013; 28(2): 280–6
Popova S, Lange S, Probst C, Gmel G, Rehm J, Centre for Addiction and Mental Health (Canada). Estimation of national, regional and global prevalence of alcohol use during pregnancy and fetal alcohol syndrome: a systematic review and meta-analysis. Lancet Glob Health. [Forthcoming]
Lee SB, Kim KM, An J, Lee D, Shim JH, Lim YS, Lee HC, Chung YH, Lee YS. Clinical characteristics and potential aetiologies of non-B non-C hepatocellular carcinoma in hepatitis B virus endemic area. Liver Int. 2016; 36(9): 1351-61
Park JH, Bang YR, Kim CK. Sex and age differences in psychiatric disorders among children and adolescents: High-risk students study. Psychiatry Investig. 2014; 11(3): 251–7
Park S, Kim BN, Cho SC, Kim JW, Shin MS, Yoo HJ. Prevalence, correlates, and comorbidities of DSM-IV psychiatric disorders in children in Seoul, Korea. Asia Pac J Public Health. 2015; 27(2): NP1942-51
Kim J, Han Y, Seo SC, Lee JY, Choi J, Kim KH, Woo SY, Kim EH, Kwon HJ, Cheong HK, Oh I, Ahn K. Association of carbon monoxide levels with allergic diseases in children. Allergy Asthma Proc. 2016; 37(1): e1-7
Yoon S, Kim JM, Kang HJ, Bae KY, Kim SW, Shin IS, Yoon JS. Associations of Pulmonary Function with Dementia and Depression in an Older Korean Population. Psychiatry Investig. 2015; 12(4): 443-50

Park JH, Seo JH, Hong YS, Kim YM, Kang JW, Yoo JH, Chueh HW, Lee JH, Kwak MJ, Kim J, Woo HD, Kim DW, Bang YR, Choe BM. Blood lead concentrations and attention deficit hyperactivity disorder in Korean children: a hospital-based case control study. BMC Pediatr. 2016; 16(1): 156
Joo H, Lim MH, Ha M, Kwon HJ, Yoo SJ, Choi KH, Paik KC. Secondhand Smoke Exposure and Low Blood Lead Levels in Association With Attention-Deficit Hyperactivity Disorder and Its Symptom Domain in Children: A Community-Based Case-Control Study. Nicotine Tob Res. 2016; nan
Kim YD, Eom SY, Yim DH, Kim IS, Won HK, Park CH, Kim GB, Yu SD, Choi BS, Park JD, Kim H. Environmental Exposure to Arsenic, Lead, and Cadmium in People Living near Janghang Copper Smelter in Korea. J Korean Med Sci. 2016; 31(4): 489-96
Seo J, Lee BK, Jin SU, Jang KE, Park JW, Kim YT, Park SJ, Jeong KS, Park J, Kim A, Kim Y, Chang Y. Altered executive function in the lead-exposed brain: A functional magnetic resonance imaging study. Neurotoxicology. 2015; 50: 1-9
Park S, Sim CS, Lee H, Kim Y. Effects of iron therapy on blood lead concentrations in infants. J Trace Elem Med Biol. 2014; 28(1): 56-9
Hong YC, Kulkarni SS, Lim YH, Kim E, Ha M, Park H, Kim Y, Kim BN, Chang N, Oh SY, Kim YJ, Park C, Ha EH. Postnatal growth following prenatal lead exposure and calcium intake. Pediatrics. 2014; 134(6): 1151-9
Jeong KS, Park H, Ha E, Hong YC, Ha M, Park H, Kim BN, Lee SJ, Lee KY, Kim JH, Kim Y. Evidence that cognitive deficit in children is associated not only with iron deficiency, but also with blood lead concentration: a preliminary study. J Trace Elem Med Biol. 2015; 29: 336-41
Choi WJ, Kwon HJ, Lim MH, Lim JA, Ha M. Blood lead, parental marital status and the risk of attention-deficit/hyperactivity disorder in elementary school children: A longitudinal study. Psychiatry Res. 2016; 236: 42-6
Kim KN, Kwon HJ, Hong YC. Low-level lead exposure and autistic behaviors in school-age children. Neurotoxicology. 2016; 53: 193-200
Burm E, Song I, Ha M, Kim YM, Lee KJ, Kim HC, Lim S, Kim SY, Lee CG, Kim SY, Cheong HK, Sakong J, Kang HT, Son M, Oh GJ, Kim Y, Yang JY, Hong SJ, Seo JH, Kim J, Oh S, Yu J, Chang SS, Kwon HJ, Choi YH, Choi W, Kim S, Yu SD. Representative levels of blood lead, mercury, and urinary cadmium in youth: Korean Environmental Health Survey in Children and Adolescents (KorEHS-C), 2012-2014. Int J Hyg Environ Health. 2016; 219(4-5): 412-8
Korean National Tuberculosis Association. South Korea Tuberculosis Prevalence Survey 1990. 1993
Chronic Kidney Disease Prognosis Consortium (CKD-PC). Korea, South National Health and Examination Survey Chronic Kidney Disease Prevalence Data 2004-2011 [Unpublished]
Lee CW, Hwang SM, Lee YS, Kim MA, Seo K. Prevalence of orofacial clefts in Korean live births. Obstet Gynecol Sci. 2015; 58(3): 196-202
Kwon SU, Yun SC, Kim MC, Kim BJ, Lee SH, Lee SO, Choi SH, Kim YS, Woo JH, Kim SH. Risk of stroke and transient ischaemic attack after herpes zoster. Clin Microbiol Infect. 2016; 22(6): 542-8
Kim YJ, Lee CN, Lim CY, Jeon WS, Park YM. Population-based study of the epidemiology of herpes zoster in Korea. J Korean Med Sci. 2014; 29(12): 1706-10
Euromonitor International. Euromonitor Passport - Fresh Foods Market Statistics. London, United Kingdom: Euromonitor International
Goo YK, Ji SY, Shin HI, Moon JH, Cho SH, Lee WJ, Kim JY. First evaluation of glucose-6-phosphate dehydrogenase (G6PD) deficiency in vivax malaria endemic regions in the South Korea. PLoS One. 2014; 9(5): e97390
Abe K, Edamoto Y, Park YN, Nomura AM, Taltavull TC, Tani M, Thung SN. In situ detection of hepatitis B, C, and G virus nucleic acids in human hepatocellular carcinoma tissues from different geographic regions. Hepatology. 1998; 28(2): 568-72
Ding X, Park YN, Taltavull TC, Thung SN, Jin X, Jin Y, Trung NS, Edamoto Y, Sata T, Abe K. Geographic characterization of hepatitis virus infections, genotyping of hepatitis B virus, and p53 mutation in hepatocellular carcinoma analyzed by in situ detection of viral genomes from carcinoma tissues: comparison among six different countries. Jpn J Infect Dis. 2003; 56(1): 12-8
Kim BK, Kim SU, Park JY, Kim DY, Ahn SH, Park MS, Kim EH, Seong J, Lee DY, Han K-H. Applicability of BCLC stage for prognostic stratification in comparison with other staging systems: single centre experience from long-term clinical outcomes of 1717 treatment-naïve patients with hepatocellular carcinoma. Liver Int. 2012; 32(7): 1120-7
Kim YJ, Jang BK, Kim ES, Chung WJ, Park KS, Cho KB, Hwang JS. Hepatocellular Carcinoma in the Elderly: Clinical Characteristics, Treatment, Survival Analysis in Korean Patients Older than 70 Years. J Korean Med Sci. 2012; 27(10): 1147-54
United Nations Statistics Division (UNSD). United Nations Demographic Yearbook 2015. New York, United States of America: United Nations (UN), 2016
Hong JW, Noh JH, Kim D-J. Factors Associated With High Sodium Intake Based on Estimated 24-Hour Urinary Sodium Excretion: The 2009-2011 Korea National Health and Nutrition Examination Survey. Medicine (Baltimore). 2016; 95(9): e2864
Kim KH, Sohn YM, Kang JH, Kim KN, Kim DS, Kim JH, Kim CH, Shin YK, Oh SH, Lee HJ, Cha SH, Hong YJ, Sohn KC. The Causative Organisms of Bacterial Meningitis in Korean Children, 1986-1995. J Korean Med Sci. 1998; 13(1): 60-4
Kim HY, Kim CW, Choi JY, Lee CD, Lee SH, Kim MY, Jang BK, Wo HY. Complications Requiring Hospital Admission and Causes of In-Hospital Death over Time in Alcoholic and Nonalcoholic Cirrhosis Patients. Gut Liver. 2016; 10(1): 95-100
KIM J-S. Prevalence and Factors Associated With Hearing Loss and Hearing Aid Use in Korean Elders. Iran J Public Health. 2015; 44(3): 308–17
Jun HJ, Hwang SY, Lee SH, Lee JE, Song J-J, Chae S. The prevalence of hearing loss in South Korea: data from a population-based study. Laryngoscope. 2015; 125(3): 690–4
World Health Organization (WHO). WHO Mortality Database Version March 2017. Geneva, Switzerland: World Health Organization (WHO)
World Health Organization (WHO). WHO Global Project on Anti-Tuberculosis Drug Resistance Surveillance Data 1988-2015
Kim TS, Kim MJ, Kim SH, Seo JJ, Kee HY, Jung JK, Ha DR, Kim ES, Moon YW, Lim SK, Kim MK, Nam HM. Serotypes of Salmonella isolated from faeces of patients with acute diarrhoea in Gwangju Area, Korea, during 2000-2009. Zoonoses Public Health. 2012; 59(7): 482–9
Shin HR, Lee CU, Park HJ, Seol SY, Chung JM, Choi HC, Ahn YO, Shigemastu T. Hepatitis B and C virus, Clonorchis sinensis for the risk of liver cancer: a case-control study in Pusan, Korea . Int J Epidemiol. 1996; 25(5): 933-40
Yun EH, Lim MK, Oh J-K, Park JH, Shin A, Sung J, Park E-C. Combined effect of socioeconomic status, viral hepatitis, and lifestyles on hepatocelluar carcinoma risk in Korea . Br J Cancer. 2010; 103(5): 741-6
Min YW. Clinical course of sub-centimeter-sized nodules detected during surveillance for hepatocellular carcinoma . World J Gastroenterol. 2012; 18(21): 2654

Oh J-K, Shin H-R, Lim MK, Cho H, Kim D-I, Jee Y, Yun H, Yoo K-Y. Multiplicative synergistic risk of hepatocellular carcinoma development among hepatitis B and C co-infected subjects in HBV endemic area: a community-based cohort study . BMC Cancer. 2012; 12(1): 452

Park C-H, Jeong S-H, Yim H-W, Kim JD, Bae SH, Choi JY, Yoon SK. Family history influences the early onset of hepatocellular carcinoma . World J Gastroenterol. 2012; 18(21): 2661-7

Ryu SH, Chung Y-H, Lee H, Kim JA, Shin HD, Min HJ, Seo DD, Jang MK, Yu E, Kim K-W. Metastatic tumor antigen 1 is closely associated with frequent postoperative recurrence and poor survival in patients with hepatocellular carcinoma . Hepatology. 2008; 47(3): 929-36

World Health Organization (WHO). Sodium Intakes Around the World. 2007

Hong JS, Kang HC, Lee SH, Kim J. Long-term trend in the incidence of acute myocardial infarction in Korea: 1997-2007. Korean Circ J. 2009; 39(11): 467–76

Kim K-I, Chang H-J, Cho Y-S, Youn T-J, Chung W-Y, Chae I-H, Choi D-J, Kim C-H. Current status and characteristics of hypertension control in community resident elderly Korean people: data from a Korean longitudinal study on health and aging (KLoSHa study). Hypertens Res. 2008; 31(1): 97–105

World Health Organization (WHO). WHO Global Health Observatory - Cholera: Number of Reported Deaths by Country. Geneva, Switzerland: World Health Organization (WHO)

International Road Federation. World Road Statistics 2009 Database and World Road Statistics 2007

BP. BP Statistical Review of World Energy 2016. London, United Kingdom: BP, 2016

Kim DH, Kim HJ, Park S-K, Kong S-J, Kim YS, Kim T-H, Kim EK, Lee KM, Lee S-S, Park JS, Koh W-J, Lee C-H, Shim TS. Treatment Outcomes and Survival Based on Drug Resistance Patterns in Multidrug-resistant Tuberculosis. Am J Respir Crit Care Med. 2010; 182(1): 113–9

Kwon Y-S, Kim YH, Song J-U, Jeon K, Song J, Ryu YJ, Choi JC, Kim HC, Koh W-J. Risk Factors for Death during Pulmonary Tuberculosis Treatment in Korea: A Multicenter Retrospective Cohort Study. J Korean Med Sci. 2014; 29(9): 1226

Song S, Jeon D, Kim JW, Kim YD, Kim S-P, Cho JS, Lee SE, Cho WH, Park H-K, Kim YS, Lee MK, Lee JH. Performance of Confirmatory Interferon-γ Release Assays in School TB Outbreaks. Chest. 2012; 141(4): 983–8

International Labour Organization (ILO). International Labour Organization Database (ILOSTAT) - Employment to Population Ratio by Sex and Age. International Labour Organization (ILO)

Global Burden of Disease Collaborative Network. Global Burden of Disease Study 2016 (GBD 2016) Covariates 1980-2016. Seattle, United States of America: Institute for Health Metrics and Evaluation (IHME), 2017

Organization for Economic Co-operation and Development (OECD). South Korea Survey of Adult Skills Round I 2011-2012. Paris, France: Organization for Economic Co-operation and Development (OECD)

Malaria Atlas Project. Malaria Atlas Project Annual Parasite Incidence Database

World Malaria Report 2015 as it appears in Malaria Atlas Project. Malaria Atlas Project Annual Parasite Incidence Database

World Malaria Report 2016 as it appears in Malaria Atlas Project. Malaria Atlas Project Annual Parasite Incidence Database

South Korea Infectious Diseases Surveillance Yearbook 2013 as it appears in Malaria Atlas Project. Malaria Atlas Project Annual Parasite Incidence Database

Multinational Annual Parasite Incidence Data, Personal Communication with Malaria Atlas Project Group 2016 as it appears in Malaria Atlas Project. Malaria Atlas Project Annual Parasite Incidence Database

South Korea Infectious Diseases Statistics System Online Database - Malaria Cases as it appears in Malaria Atlas Project. Malaria Atlas Project Annual Parasite Incidence Database

South Korea Infectious Diseases Surveillance Yearbook 2018 as it appears in Malaria Atlas Project. Malaria Atlas Project Annual Parasite Incidence Database

World Malaria Report 2019 as it appears in Malaria Atlas Project. Malaria Atlas Project Annual Parasite Incidence Database

ISSP Research Group (2008): International Social Survey Programme: Role of Government IV - ISSP 2006. GESIS Data Archive, Cologne. ZA4700 Data file Version 1.0.0, doi:10.4232/1.4700

International Social Survey Programme (ISSP). South Korea International Social Survey Programme: Religion III 2008. Mannheim, Germany: GESIS - Leibniz Institute for the Social Sciences, 2012

ISSP Research Group (2017): International Social Survey Programme: Social Inequality IV - ISSP 2009. GESIS Data Archive, Cologne. ZA5400 Data file Version 4.0.0, doi:10.4232/1.12777.

ISSP Research Group (2012): International Social Survey Programme: Environment III - ISSP 2010. GESIS Data Archive, Cologne. ZA5500 Data file Version 2.0.0, doi:10.4232/1.11418.

ISSP Research Group (2016): International Social Survey Programme: Family and Changing Gender Roles IV - ISSP 2012. GESIS Data Archive, Cologne. ZA5900 Data file Version 4.0.0, doi:10.4232/1.12661

ISSP Research Group (2012): International Social Survey Programme: National Identity II - ISSP 2003. GESIS Data Archive, Cologne. ZA3910 Data file Version 2.1.0, doi:10.4232/1.11449

International Social Survey Programme (ISSP). South Korea International Social Survey Programme: Citizenship 2004. Mannheim, Germany: GESIS - Leibniz Institute for the Social Sciences, 2012

International Social Survey Programme (ISSP). South Korea International Social Survey Programme: Work Orientation III 2005. Mannheim, Germany: GESIS - Leibniz Institute for the Social Sciences, 2013

Gjonbrataj J, Choi WI, Bahn YE, Rho BH, Lee JJ, Lee CW. Incidence of idiopathic pulmonary fibrosis in Korea based on the 2011 ATS/ERS/JRS/ALAT statement. Int J Tuberc Lung Dis. 2015; 19(6): 742-6

Han S, Mok Y, Jee SH, Danoff SK. Incidence And Mortality Of Idiopathic Pulmonary Fibrosis In South Korea. Am J Respir Crit Care Med. 2013; a1460

Kim YH, Kim NH, Jung MH, Kim HJ. Sex differences in metabolic risk indicator of dementia in an elderly urban Korean population: A community-based cross-sectional study. Geriatr Gerontol Int. 2017

Han JW, So Y, Kim TH, Lee DY, Ryu SH, Kim SY, Kim SG, Kim SK, Youn JC, Jhoo JH, Kim JL, Lee SB, Lee JJ, Kwak KP, Moon SW, Kim BJ, Bae JN, Woo JI, Jeong H, Park JH, Kim YJ, Kim KW. Prevalence Rates of Dementia and Mild Cognitive Impairment Are Affected

by the Diagnostic Parameter Changes for Neurocognitive Disorders in the DSM-5 in a Korean Population. Dement Geriatr Cogn Disord. 2017; 43(3-4): 193-203
World Health Organization (WHO). Global leprosy update, 2016: accelerating reduction of disease burden. Wkly Epidemiol Rec. 2017; 92(35): 501-20
World Health Organization (WHO). WHO Mortality Database Version October 2017. Geneva, Switzerland: World Health Organization (WHO)
South Korea Vital Registration - Deaths 2014 ICD10 as it appears in World Health Organization (WHO). WHO Mortality Database Version October 2017. Geneva, Switzerland: World Health Organization (WHO)
South Korea Vital Registration - Deaths 2015 ICD10 as it appears in World Health Organization (WHO). WHO Mortality Database Version October 2017. Geneva, Switzerland: World Health Organization (WHO)
Kim J, Cho SJ, Kim WJ, Yang KI, Yun CH, Chu MK. Insomnia in tension-type headache: a population-based study. J Headache Pain. 2017; 18(1): 95
Bray F, Colombet M, Mery L, Piñeros M, Znaor A, Zanetti R and Ferlay J, editors (2017). Cancer Incidence in Five Continents, Vol. XI (electronic version). Lyon: International Agency for Research on Cancer. Available from: http://ci5.iarc.fr
South Korea Cancer Registry 2008-2012 - CI5 as it appears in Bray F, Colombet M, Mery L, Piñeros M, Znaor A, Zanetti R and Ferlay J, editors (2017). Cancer Incidence in Five Continents, Vol. XI (electronic version). Lyon: International Agency for Research on Cancer. Available from: http://ci5.iarc.fr
Food Fortification Initiative (United States), Global Alliance for Improved Nutrition (GAIN), Iodine Global Network (Canada), Micronutrient Forum (Canada). Global Fortification Data Exchange: Legislation and Standards, Year When Food Fortification Mandated, and Count of Nutrients In Fortification Standards
ISSP Research Group (2015): International Social Survey Programme: National Identity III - ISSP 2013. GESIS Data Archive, Cologne. ZA5950 Data file Version 2.0.0, doi:10.4232/1.12312
ISSP Research Group (2016): International Social Survey Programme: Citizenship II - ISSP 2014. GESIS Data Archive, Cologne. ZA6670 Data file Version 2.0.0, doi:10.4232/1.12590
Kim SH, Yun JM, Chang CB, Piao H, Yu SJ, Shin DW. Prevalence of upper gastrointestinal bleeding risk factors among the general population and osteoarthritis patients. World J Gastroenterol. 2016; 22(48): 10643-52
Min BH, Huh KC, Jung HK, Yoon YH, Choi KD, Song KH, Keum B, Kim JW. Prevalence of uninvestigated dyspepsia and gastroesophageal reflux disease in Korea: a population-based study using the Rome III criteria. Dig Dis Sci. 2014; 59(11): 2721-9
Lee SY, Lee KJ, Kim SJ, Cho SW. Prevalence and risk factors for overlaps between gastroesophageal reflux disease, dyspepsia, and irritable bowel syndrome: a population-based study. Digestion. 2009; 79(3): 196-201
Kim N, Lee SW, Cho SI, Park CG, Yang CH, Kim HS, Rew JS, Moon JS, Kim S, Park SH, Jung HC, Chung IS. The prevalence of and risk factors for erosive oesophagitis and non-erosive reflux disease: a nationwide multicentre prospective study in Korea. Aliment Pharmacol Ther. 2008; 27(2): 173-85
Yang SY, Lee OY, Bak YT, Jun DW, Lee SP, Lee SH, Park GT, Yoon BC, Choi HS, Hahm JS, Lee MH, Lee DH. Prevalence of gastroesophageal reflux disease symptoms and uninvestigated dyspepsia in Korea: a population-based study. Dig Dis Sci. 2008; 53(1): 188-93
Kim W, Jang SY, Chun SY, Lee TH, Han KT, Park EC. Mortality in Schizophrenia and Other Psychoses: Data from the South Korea National Health Insurance Cohort, 2002-2013. J Korean Med Sci. 2017; 32(5): 835–42
Kim IJ, Kim DH, Song YW, Guermazi A, Crema MD, Hunter DJ, Seo Y-I, Kim HA. The prevalence of periarticular lesions detected on magnetic resonance imaging in middle-aged and elderly persons: a cross-sectional study. BMC Musculoskelet Disord. 2016; 17: 186
Cho HJ, Morey V, Kang JY, Kim KW, Kim TK. Prevalence and Risk Factors of Spine, Shoulder, Hand, Hip, and Knee Osteoarthritis in Community-dwelling Koreans Older Than Age 65 Years. Clin Orthop Relat Res. 2015; 473(10): 3307-3314
Statistics Korea (South Korea). South Korea KOSIS Database - Deaths by Age and Sex: Province
Kim MJ, Lim NK, Park HY. Relationship between prehypertension and chronic kidney disease in middle-aged people in Korea: the Korean genome and epidemiology study. BMC Public Health. 2012; 12: 960
Shamputa IC, Lee J, Allix-Béguec C, Cho E-J, Lee J, Rajan V, Lee EG, Min JH, Carroll MW, Goldfeder LC, Kim JH, Kang HS, Hwang S, Eum S-Y, Park SK, Lee H, Supply P, Cho S-N, Via LE, Barry CE. Genetic diversity of Mycobacterium tuberculosis isolates from a tertiary care tuberculosis hospital in South Korea. J Clin Microbiol. 2010; 48(2): 387–94
Choi GE, Jang MH, Song EJ, Jeong SH, Kim J-S, Lee WG, Uh Y, Roh KH, Lee HS, Shin JH, Ryoo NH, Kim YR, Jeong J, Kim JH, Lee SM, Yi J, Hwang SH, Kim HH, Lee EY, Chang CL, Kim M-B, Kim YD. IS6110-restriction fragment length polymorphism and spoligotyping analysis of Mycobacterium tuberculosis clinical isolates for investigating epidemiologic distribution in Korea. J Korean Med Sci. 2010; 25(12): 1716–21
Kim BJ, Cheong ES, Kang JG, Kim BS, Kang JH. Relationship of epicardial fat thickness and nonalcoholic fatty liver disease to coronary artery calcification: From the CAESAR study. J Clin Lipidol. 2016; 10(3): 619-626e1
Chung GE, Kim D, Kwak MS, Yang JI, Yim JY, Lim SH, Itani M. The serum vitamin D level is inversely correlated with nonalcoholic fatty liver disease. Clin Mol Hepatol. 2016; 22(1): 146-51
Ryu S, Chang Y, Jung HS, Yun KE, Kwon MJ, Choi Y, Kim CW, Cho J, Suh BS, Cho YK, Chung EC, Shin H, Kim YS. Relationship of sitting time and physical activity with non-alcoholic fatty liver disease. J Hepatol. 2015; 63(5): 1229-37
Kim JY, Lee C, Oh M, Im JA, Lee JW, Chu SH, Lee H, Jeon JY. Relationship between non-alcoholic fatty liver disease, metabolic syndrome and insulin resistance in Korean adults: A cross-sectional study. Clin Chim Acta. 2016; 458: 12-7
United Nations Statistics Division (UNSD). United Nations Demographic Yearbook 2016. New York, United States of America: United Nations (UN), 2017
Lee YI, Lim YS, Park HS. Colorectal neoplasms in relation to non-alcoholic fatty liver disease in Korean women: a retrospective cohort study. J Gastroenterol Hepatol. 2012; 27(1): 91-5
Kim SH, Lee JW, Hwang HJ. Associations between combinations of body mass index plus non-alcoholic fatty liver disease and diabetes mellitus among Korean adults. Asia Pac J Clin Nutr. 2011; 20(1): 14-20
Park SH, Jeon WK, Kim SH, Kim HJ, Park DI, Cho YK, Sung IK, Sohn CI, Keum DK, Kim BI. Prevalence and risk factors of non-alcoholic fatty liver disease among Korean adults. J Gastroenterol Hepatol. 2006; 21(1 Pt 1): 138-43
Lee HS, Kim YJ, You SH, Jang YG, Rhee WT, Lee SY. The Incidence of Aneurysmal Subarachnoid Hemorrhage in Youngdong District, Korea. J Korean Neurosurg Soc. 2007; 42(4): 258-64
Jeong JJ, Choi MG, Cho YS, Lee SG, Oh JH, Park JM, Cho YK, Lee IS, Kim SW, Han SW, Choi KY, Chung IS. Chronic gastrointestinal symptoms and quality of life in the Korean population. World J Gastroenterol. 2008; 14(41): 6388-94

Seo BR, Kim TS, Joo SP, Jang SP, Lim JS, Oh CW. Incidence Rate of Aneurysmal SAH in Gwangju City and Jeollanamdo Province in 2007. J Korean Neurosurg Soc. 2010; 47(2): 124-7
Kim YJ, Lee BE, Park HS, Kang JG, Kim JO, Ha EH. Risk factors for preterm birth in Korea: a multicenter prospective study. Gynecol Obstet Invest. 2005; 60(4): 206-12
Kim G, Lee YH, Lee BW, Kang ES, Lee IK, Cha BS, Kim DJ. Diabetes self-assessment score and the development of diabetes: A 10-year prospective study. Medicine (Baltimore). 2017; 96(23): e7067
Sung Y-K, Cho S-K, Choi C-B, Bae S-C. Prevalence and incidence of rheumatoid arthritis in South Korea. Rheumatol Int. 2013; 33(6): 1525–32
Kim JH, Lee CG, Lee YA, Yang SW, Shin CH. Increasing incidence of type 1 diabetes among Korean children and adolescents: analysis of data from a nationwide registry in Korea. Pediatr Diabetes. 2016; 17(7): 519–24
World Health Organization (WHO). WHO Mortality Database Version April 2018. Geneva, Switzerland: World Health Organization (WHO)
World Health Organization (WHO). WHO International Travel and Health, Annex 1, Countries With Risk of Yellow Fever Transmission and Countries Requiring Yellow Fever Vaccination 2017 Update. Geneva, Switzerland: World Health Organization (WHO), 2017
Gallup. South Korea World Poll 2005-2006
Gallup. South Korea World Poll 2007
Gallup. South Korea World Poll 2012
Centers for Disease Control and Prevention (CDC), Korea Centers for Disease Control and Prevention (KCDC), World Health Organization (WHO). South Korea Global Youth Tobacco Survey 2013. Atlanta, United States of America: Centers for Disease Control and Prevention (CDC)
Han DH, Khang YH. Lifecourse socioeconomic position indicators and tooth loss in Korean adults. Community Dent Oral Epidemiol. 2016
Kim EK, Jung YS, Kim KH, Kim KR, Kwon GH, Choi YH, Lee HK. Social capital and oral health: The association of social capital with edentulism and chewing ability in the rural elderly. Arch Gerontol Geriatr. 2018; 74: 100-105
Kim AH, Shim YS, Kim JB, An SY. Caries Prevalence in Korean Children and Adolescents from 2000 to 2012. J Clin Pediatr Dent. 2017; 41(1): 32-7
Kim K, Kim Y. Clinical Comparison of 30-Day Mortalities and 6-Month Functional Recoveries after Spontaneous Intracerebral Hemorrhage in Patients with or without End-Stage Renal Disease. J Korean Neurosurg Soc. 2013; 54(3): 164-74
Kim K, Kim H, Kim Y. Comparisons of 30-day mortalities and 90-day functional recoveries after first and recurrent primary intracerebral hemorrhage attacks: a multiple-institute retrospective study. World Neurosurg. 2013; 79(3-4): 489-98
Lim J-S, Kwon H-M, Kim S-E, Lee J, Lee Y-S, Yoon B-W. Effects of Temperature and Pressure on Acute Stroke Incidence Assessed Using a Korean Nationwide Insurance Database. J Stroke. 2017; 19(3): 295–303
Kim T-S, Cho S-H, Huh S, Kong Y, Sohn W-M, Hwang S-S, Chai J-Y, Lee S-H, Park Y-K, Oh D-K, Lee J-K, Working Groups in National Institute of Health, Korea Association of Health Promotion. A nationwide survey on the prevalence of intestinal parasitic infections in the South Korea, 2004. Korean J Parasitol. 2009; 47(1): 37–47
Byoung-Hak J, Eun, LS, Ran KJ, Hyun KJ, Han LD, Division of Infectious Diseases Surveillance, Center of Infectious Diseases Control, KCDC. An Epidemiological Survey for Zika Virus Infection Cases in Korea, 2016-2017. Osong-eup, South Korea: Korea Centers for Disease Control and Prevention (KCDC). KCDC Public Health Weekly Report. 2008; 11(8): 2012-6
Sung JH, Oh I, Kim A, Lee J, Sim CS, Yoo C, Park SJ, Kim GB, Kim Y. Environmental and Body Concentrations of Heavy Metals at Sites Near and Distant from Industrial Complexes in Ulsan, Korea. J Korean Med Sci. 2018; 33(5): e33
Kim KN, Lee MR, Lim YH, Hong YC. Blood lead levels, iron metabolism gene polymorphisms and homocysteine: a gene-environment interaction study. Occup Environ Med. 2017; 74(12): 899-904
Kim SH, Kim YH, An HC, Sung JH, Sim CS. Levels of blood lead and urinary cadmium in industrial complex residents in Ulsan. Ann Occup Environ Med. 2017; 29: 26
Eom SY, Hwang MS, Lim JA, Choi BS, Kwon HJ, Park JD, Kim YD, Kim H. Exome-wide association study identifies genetic polymorphisms of C12orf51, MYL2, and ALDH2 associated with blood lead levels in the general Korean population. Environ Health. 2017; 16(1): 11
Lee S, Shin M, Hong YC, Kim JH. Temporal variability of blood lead, mercury, and cadmium levels in elderly panel study (2008-2014). Int J Hyg Environ Health. 2017; 220(2 Pt B): 407-414
Choi W, Kim S, Baek YW, Choi K, Lee K, Kim S, Yu SD, Choi K. Exposure to environmental chemicals among Korean adults-updates from the second Korean National Environmental Health Survey (2012-2014). Int J Hyg Environ Health. 2017; 220(2 Pt A): 29-35
Han DH, Khang YH, Lee HJ. Association between adult height and tooth loss in a representative sample of Koreans. Community Dent Oral Epidemiol. 2015; 43(5): 479-88
Kim H-J, Choi SM, Lee J, Park YS, Lee C-H, Yim J-J, Yoo C-G, Kim YW, Han SK, Lee S-M. Respiratory virus of severe pneumonia in South Korea: Prevalence and clinical implications. PLoS One. 2018; 13(6): e0198902
International Fuel Quality Center (IFQC). Overview of Leaded Gasoline and Sulfur Levels in Gasoline and Diesel . 2002
Global Burden of Disease Collaborative Network. Global Burden of Disease Study 2017 (GBD 2017) Cause-Specific Mortality 1980-2017. Seattle, United States of America: Institute for Health Metrics and Evaluation (IHME), 2018
International Agency for Research on Cancer (IARC), International Association of Cancer Registries (IACR), World Health Organization (WHO). International Incidence of Childhood Cancer Volume III. Lyon, France: International Agency for Research on Cancer (IARC), 2017
South Korea Cancer Registry 1999-2012 - IICC-3 as it appears in International Agency for Research on Cancer (IARC), International Association of Cancer Registries (IACR), World Health Organization (WHO). International Incidence of Childhood Cancer Volume III. Lyon, France: International Agency for Research on Cancer (IARC), 2017
Organization for Economic Co-operation and Development (OECD). Organization for Economic Co-operation and Development Data - Hospital Beds. Paris, France: Organization for Economic Co-operation and Development (OECD)
World Health Organization (WHO). WHO Global Health Observatory - Hospital Bed Density Data by Country . Geneva, Switzerland: World Health Organization (WHO)
World Health Organization (WHO). Global leprosy update, 2017: reducing the disease burden due to leprosy. Wkly Epidemiol Rec. 2018; 93(35): 445-56

World Health Organization (WHO). WHO Mortality Database Version December 2018. Geneva, Switzerland: World Health Organization (WHO)
South Korea Vital Registration - Deaths 2016 ICD10 as it appears in World Health Organization (WHO). WHO Mortality Database Version December 2018. Geneva, Switzerland: World Health Organization (WHO)
Bae KJ, Gong HS, Kim KW, Kim TK, Chang CB, Jang HC, Baek GH. Evaluation of femoral neck bone mineral density and radiographic hand and knee osteoarthritis in a Korean elderly population. Clin Orthop Surg. 2014; 6(3): 343-9
Chung W, Park Y, Jeon C, Jung J, Ko K, Choi S, Seo H, Lee J, Jung H. Baseline Characteristics of the Korean Registry of Pulmonary Arterial Hypertension. J Korean Med Sci. 2015; 30(10): 1429-38
Kim H, Kim G, Je H, Beak J, Bae E, Noh C, Choi J, Yun Y. Pulmonary Arterial Hypertension in Children: A Single Center Experience. Korean Circ J. 2008; 3838(12): 644 - 650
Cho HJ, Jin BH, Park DY, Jung SH, Lee HS, Paik DI, Bae KH. Systemic effect of water fluoridation on dental caries prevalence. Community Dent Oral Epidemiol. 2014; 42(4): 341-8
Kodama R, Muraki S, Oka H, Iidaka T, Teraguchi M, Kagotani R, Asai Y, Yoshida M, Morizaki Y, Tanaka S, Kawaguchi H, Nakamura K, Akune T, Yoshimura N. Prevalence of hand osteoarthritis and its relationship to hand pain and grip strength in Japan: The third survey of the ROAD study. Mod Rheumatol. 2016; 26(5): 767-73
Kim HJ, Hann HJ, Hong SN, Kim KH, Ahn IM, Song JY, Lee SH, Ahn HS. Incidence and natural course of inflammatory bowel disease in Korea, 2006-2012: a nationwide population-based study. Inflamm Bowel Dis. 2015; 21(3): 623-30
Kim JH, Lee Y. Dementia and Death After Stroke in Older Adults During a 10-year Follow-up: Results from a Competing Risk Model. J Nutr Health Aging. 2018; 22(2): 297-301
South Korea World Poll 2016 as it appears in Gallup. Gallup Analytics - World Poll Negative Experience Index. Washington, D.C., United States of America: Gallup, 2019
South Korea World Poll 2017 as it appears in Gallup. Gallup Analytics - World Poll Negative Experience Index. Washington, D.C., United States of America: Gallup, 2019
South Korea World Poll 2008 as it appears in Gallup. Gallup Analytics - World Poll Negative Experience Index. Washington, D.C., United States of America: Gallup, 2019
South Korea World Poll 2009 as it appears in Gallup. Gallup Analytics - World Poll Negative Experience Index. Washington, D.C., United States of America: Gallup, 2019
South Korea World Poll 2010 as it appears in Gallup. Gallup Analytics - World Poll Negative Experience Index. Washington, D.C., United States of America: Gallup, 2019
South Korea World Poll 2011 as it appears in Gallup. Gallup Analytics - World Poll Negative Experience Index. Washington, D.C., United States of America: Gallup, 2019
South Korea World Poll 2013 as it appears in Gallup. Gallup Analytics - World Poll Negative Experience Index. Washington, D.C., United States of America: Gallup, 2019
South Korea World Poll 2014 as it appears in Gallup. Gallup Analytics - World Poll Negative Experience Index. Washington, D.C., United States of America: Gallup, 2019
South Korea World Poll 2015 as it appears in Gallup. Gallup Analytics - World Poll Negative Experience Index. Washington, D.C., United States of America: Gallup, 2019
South Korea World Poll 2018 as it appears in Gallup. Gallup Analytics - World Poll Negative Experience Index. Washington, D.C., United States of America: Gallup, 2019
Seo HJ, Jung YE, Kim MD, Bahk WM. Factors associated with bullying victimization among Korean adolescents. Neuropsychiatr Dis Treat. 2017; 13: 2429-2435
Shin C, Kim Y, Park S, Yoon S, Ko YH, Kim YK, Kim SH, Jeon SW, Han C. Prevalence and Associated Factors of Depression in General Population of Korea: Results from the Korea National Health and Nutrition Examination Survey, 2014. J Korean Med Sci. 2017; 32(11): 1861-1869
Song TJ, Cho SJ, Kim WJ, Yang KI, Yun CH, Chu MK. Anxiety and depression in probable migraine: A population-based study. Cephalalgia. 2017; 37(9): 845-854
Choi YH, Kim JH, Kim DK, Kim JW, Kim DK, Lee MS, Kim CH, Park SC. Distributions of ACE and APOE polymorphisms and their relations with dementia status in Korean centenarians. J Gerontol A Biol Sci Med Sci. 2003; 58(3): 227-31
Kim H, Jhoo JH, Jang JW. The effect of telemedicine on cognitive decline in patients with dementia. J Telemed Telecare. 2017; 23(1): 149-54
Moon Y, Lee H, Namgung OK, Han SH. Which Stratum of Urban Elderly Is Most Vulnerable for Dementia?. J Korean Med Sci. 2016; 31(10): 1635-40
Sohn WS, Yoo K, Na DL, Jeong Y. Progressive changes in hippocampal resting-state connectivity across cognitive impairment: a cross-sectional study from normal to Alzheimer disease. Alzheimer Dis Assoc Disord. 2014; 28(3): 239-46
Youn JC, Lee DY, Jhoo JH, Kim KW, Choo IH, Woo JI. Prevalence of neuropsychiatric syndromes in Alzheimer's disease (AD). Arch Gerontol Geriatr. 2011; 52(3): 258-63
International Road Federation. World Road Statistics 2018. Geneva, Switzerland: International Road Federation, 2018
Kim MJ, Park I, Lim MH, Paik KC, Cho S, Kwon HJ, Lee SG, Yoo SJ, Ha M. Prevalence of Attention-Deficit/Hyperactivity Disorder and its Comorbidity among Korean Children in a Community Population. J Korean Med Sci. 2017; 32(3): 401-406
United Nations Children's Fund (UNICEF), World Health Organization (WHO). WHO and UNICEF Reported Official Target Population, Number of Doses Administered and Official Coverage 1966-2018. Geneva, Switzerland: World Health Organization (WHO), 2019
Park JE, Yuk JS, Cho IA, Baek JC, Lee JH, Park JK. Ectopic pregnancy incidence in the South Korea in 2009-2015: A population-based cross-sectional study. Sci Rep. 2018; 8(1): 17308
Kim MK, Lee SM, Bae SH, Kim HJ, Lim NG, Yoon SJ, Lee JY, Jo MW. Socioeconomic status can affect pregnancy outcomes and complications, even with a universal healthcare system. Int J Equity Health. 2018; 17(1): 2
Oh Y-S, Kim J-S, Park I-S, Shim Y-S, Song I-U, Park J-W, Lee P-H, Lyoo C-H, Ahn T-B, Ma H-I, Kim Y-D, Koh S-B, Lee S-J, Lee K-S. Prevalence and treatment pattern of Parkinsons disease dementia in Korea. Geriatr Gerontol Int. 2016; 16(2): 2306
Cho Y, Cudhea F, Park JH, Mozaffarian D, Singh G, Shin MJ. Burdens of Cardiometabolic Diseases Attributable to Dietary and Metabolic Risks in Korean Adults 2012-2013. Yonsei Med J. 2017; 58(3): 540-551
Won JC, Hong JW, Noh JH, Kim DJ. Association Between Estimated 24-h Urinary Sodium Excretion and Metabolic Syndrome in Korean Adults: The 2009 to 2011 Korea National Health and Nutrition Examination Survey. Medicine (Baltimore). 2016; 95(15): e3153

Han DH, Kim DH, Kim MJ, Kim JB, Jung-Choi K, Bae KH. Regular dental checkup and snack-soda drink consumption of preschool children are associated with early childhood caries in Korean caregiver/preschool children dyads. Community Dent Oral Epidemiol. 2014; 42(1): 70-8
Malaria Atlas Project. Sickle Haemoglobin (Hbs) Allele Frequency Layer. Oxford, United Kingdom: Malaria Atlas Project, 2013
Yoon YS, Oh SW. Sodium density and obesity; the Korea National Health and Nutrition Examination Survey 2007-2010. Eur J Clin Nutr. 2013; 67(2): 141-6
World Health Organization (WHO). WHO Distribution of Measles Cases by Country and by Month 2011-2020
Euromonitor International. Euromonitor Passport - Smoking Tobacco Statistics . London, United Kingdom: Euromonitor International
Euromonitor International. Euromonitor Passport - Alcoholic Drinks Statistics . London, United Kingdom: Euromonitor International
Shaddick G, Thomas ML. Particulate Matter 2.5 and 10 Surface Monitor Station Expanded Database 2008-2017. [Unpublished]
South Korea Ground Monitor Station PM2.5 and PM10 Data 2014 as it appears in Shaddick G, Thomas ML. Particulate Matter 2.5 and 10 Surface Monitor Station Expanded Database 2008-2017. [Unpublished]
Shon ZH, Kim KH, Song SK, Jung K, Kim NJ, Lee JB. Relationship between water-soluble ions in PM2.5 and their precursor gases in Seoul megacity. Atmos Environ. 2012; 59: 540-50 as it appears in Shaddick G, Thomas ML. Particulate Matter 2.5 and 10 Surface Monitor Station Expanded Database 2008-2017. [Unpublished]
South Korea AirKorea Monitoring Network 2015 as it appears in Shaddick G, Thomas ML. Particulate Matter 2.5 and 10 Surface Monitor Station Expanded Database 2008-2017. [Unpublished]
South Korea AirKorea Monitoring Network 2016 as it appears in Shaddick G, Thomas ML. Particulate Matter 2.5 and 10 Surface Monitor Station Expanded Database 2008-2017. [Unpublished]
Asian Initiative for Research on Climate and Air Pollution Health Impact Assessment as it appears in Shaddick G, Thomas ML. Particulate Matter 2.5 and 10 Surface Monitor Station Expanded Database 2008-2017. [Unpublished]
United Nations Children's Fund (UNICEF). UNICEF Maternal and Newborn Health Coverage Database as of November 2019. New York, United States of America: United Nations Children's Fund (UNICEF), 2019
Global Burden of Disease Collaborative Network. Global Health Spending 1995-2017. Seattle, United States of America: Institute for Health Metrics and Evaluation (IHME), 2020
International Road Federation. World Road Statistics 2019. Geneva, Switzerland: International Road Federation, 2019
Feenstra, Robert C., Robert Inklaar and Marcel P. Timmer (2015), "The Next Generation of the Penn World Table" American Economic Review, 105(10), 3150-3182, available for download at www.ggdc.net/pwt
Maddison Project Database, version 2018. Bolt, Jutta, Robert Inklaar, Herman de Jong and Jan Luiten van Zanden (2018), “Rebasing ‘Maddison’: new income comparisons and the shape of long-run economic development”, Maddison Project Working paper 10
World Bank. World Development Indicators - Gross Domestic Product (GDP). Washington DC, United States of America: World Bank
United Nations Environment Programme. Leaded Petrol Phase-out: Global Status, March 2018. Nairobi, Kenya: United Nations Environment Programme
South Korea Vital Registration - Deaths 2017 ICD10 as it appears in World Health Organization (WHO). WHO Mortality Database Version December 2019. Geneva, Switzerland: World Health Organization (WHO)
Kim JY, Sung K. Marital Violence Among Korean Elderly Couples: A Cultural Residue. J Elder Abuse Negl. 2001; 13(4): 73-89
Kim KI, Cho YG. Epidemiological Survey of Spousal Abuse in Korea. In: Intimate Violence: Interdisciplinary Perspectives. Viano E, ed. Washington, D.C., United States: Hemisphere Publishing Corporation, 1992. p. 277-82
Korean Medical Insurance Corporation Study 1990-1992 as it appears in Singh GM, Danaei G, Farzadfar F, Stevens GA, Woodward M, Wormser D, Kaptoge S, Whitlock G, Qiao Q, Lewington S, Di Angelantonio E, Vander Hoorn S, Lawes CM, Ali MK, Mozaffarian D, Ezzati M; Global Burden of Metabolic Risk Factors of Chronic Diseases Collaborating Group; Asia-Pacific Cohort Studies Collaboration (APCSC); Diabetes Epidemiology: Collaborative analysis of Diagnostic criteria in Europe (DECODE); Emerging Risk Factor Collaboration (ERFC); Prospective Studies Collaboration (PSC). The age-specific quantitative effects of metabolic risk factors on cardiovascular diseases and diabetes: a pooled analysis. PLoS One. 2013; 8(7): e65174
Song Y-M, Sung J, Ha M. Obesity and risk of cancer in postmenopausal Korean women. J Clin Oncol. 2008; 26(20): 3395–402
Oh SW, Yoon YS, Shin S-A. Effects of excess weight on cancer incidences depending on cancer sites and histologic findings among men: Korea National Health Insurance Corporation Study. J Clin Oncol. 2005; 23(21): 4742–54
Korea Centers for Disease Control and Prevention (KCDC). South Korea National Health and Nutrition Examination Survey 2013. Osong-eup, South Korea: Korea Centers for Disease Control and Prevention (KCDC)
Food and Agriculture Organization of the United Nations (FAO). FAOSTAT Food Balance Sheets, October 2014. Rome, Italy: Food and Agriculture Organization of the United Nations (FAO)
Youn S, Woo HD, Cho YA, Shin A, Chang N, Kim J. Association between dietary carbohydrate, glycemic index, glycemic load, and the prevalence of obesity in Korean men and women. Nutr Res. 2012; 32(3): 153–9
Lim H, Kim SY, Wang Y, Lee SJ, Oh K, Sohn CY, Moon YM, Jee SH. Preservation of a traditional Korean dietary pattern and emergence of a fruit and dairy dietary pattern among adults in South Korea: secular transitions in dietary patterns of a prospective study from 1998 to 2010. Nutr Res. 2014; 34(9): 760–70
Baik I, Abbott RD, Curb JD, Shin C. Intake of fish and n-3 fatty acids and future risk of metabolic syndrome. J Am Diet Assoc. 2010; 110(7): 1018–26
Joshi S, Song YM, Kim TH, Cho SI. Socio-economic status and the risk of liver cancer mortality: a prospective study in Korean men. Public Health. 2008; 122(11): 1144-51
Korean Women's Development Institute, Ministry of Gender Equality and Family (South Korea). South Korea National Survey on Domestic Violence 2013
Korean Women's Development Institute, Ministry of Gender Equality and Family (South Korea). South Korea National Survey on Domestic Violence 2016
Jee SH, Foong AW, Hur NW, Samet JM. Smoking and risk for diabetes incidence and mortality in Korean men and women. Diabetes Care. 2010; 33(12): 2567-72

Ko KP, Min H, Ahn Y, Park SJ, Kim CS, Park JK, Kim SS. A prospective study investigating the association between environmental tobacco smoke exposure and the incidence of type 2 diabetes in never smokers. Ann Epidemiol. 2011; 21(1): 42-7
Lawlor DA, Song YM, Sung J, Ebrahim S, Smith GD. The association of smoking and cardiovascular disease in a population with low cholesterol levels: a study of 648,346 men from the Korean national health system prospective cohort study. Stroke. 2008; 39(3): 760-7
Korea Centers for Disease Control and Prevention (KCDC). South Korea National Health and Nutrition Examination Survey 2016. Osong-eup, South Korea: Korea Centers for Disease Control and Prevention (KCDC)
Park CH, Ga H, Leem JH, Kwak SM, Kim HC, Choi JH. [The effect of smoking status upon occurrence of impaired fasting glucose or type 2 diabetes in Korean men]. J Prev Med Public Health. 2008; 41(4): 249-54
Cho NH, Chan JC, Jang HC, Lim S, Kim HL, Choi SH. Cigarette smoking is an independent risk factor for type 2 diabetes: a four-year community-based prospective study. Clin Endocrinol (Oxf). 2009; 71(5): 679-85
Parr CL, Batty GD, Lam TH, Barzi F, Fang X, Ho SC, Jee SH, Ansary-Moghaddam A, Jamrozik K, Ueshima H, Woodward M, Huxley RR. Body-mass index and cancer mortality in the Asia-Pacific Cohort Studies Collaboration: pooled analyses of 424 519 participants. Lancet Oncol. 2010; 11(8): 741–52
Leem AY, Park B, Kim YS, Jung JY, Won S. Incidence and risk of chronic obstructive pulmonary disease in a Korean community-based cohort. Int J Chron Obstruct Pulmon Dis. 2018; 13: 509–17
Park HJ, Byun MK, Kim HJ, Kim JY, Kim Y-I, Yoo K-H, Chun EM, Jung JY, Lee SH, Ahn CM. Dietary vitamin C intake protects against COPD: the Korea National Health and Nutrition Examination Survey in 2012. Int J Chron Obstruct Pulmon Dis. 2016; 11: 2721–8
Wie G-A, Cho Y-A, Kang H, Ryu K-A, Yoo M-K, Kim Y-A, Jung K-W, Kim J, Lee J-H, Joung H. Red meat consumption is associated with an increased overall cancer risk: a prospective cohort study in Korea. Br J Nutr. 2014; 112(2): 238–47
Jee SH, Yun JE, Park EJ, Cho ER, Park IS, Sull JW, Ohrr H, Samet JM. Body mass index and cancer risk in Korean men and women. Int J Cancer. 2008; 123(8): 1892-6
Lee JE, McLerran DF, Rolland B, Chen Y, Grant EJ, Vedanthan R, Inoue M, Tsugane S, Gao YT, Tsuji I, Kakizaki M, Ahsan H, Ahn YO, Pan WH, Ozasa K, Yoo KY, Sasazuki S, Yang G, Watanabe T, Sugawara Y, Parvez F, Kim DH, Chuang SY, Ohishi W, Park SK, Feng Z, Thornquist M, Boffetta P, Zheng W, Kang D, Potter J, Sinha R. Meat intake and cause-specific mortality: a pooled analysis of Asian prospective cohort studies. Am J Clin Nutr. 2013; 98(4): 1032-41
Shin CS, Kim MJ, Shim SM, Kim JT, Yu SH, Koo BK, Cho HY, Choi HJ, Cho SW, Kim SW, Kim SY, Yang SO, Cho NH. The prevalence and risk factors of vertebral fractures in Korea. J Bone Miner Metab. 2012; 30(2): 183-92
European Centre for Medium-Range Weather Forecasts (ECMWF). European Reanalysis-5. United Kingdom: European Centre for Medium-Range Weather Forecasts (ECMWF)
Han KM, Jee HJ, An H, Shin C, Yoon HK, Ko YH, Ham BJ, Kim YK, Han C. Intimate partner violence and incidence of depression in married women: A longitudinal study of a nationally representative sample. J Affect Disord. 2019; 245: 305-311

Appendix table 3. Updates in methodology from GBD 2017 to GBD 2019

Category	Changes in methodology
**Overall changes in methodology in GBD 2019**	- Nine countries and territories (Tuvalu, Tokelau, Saint Kitts and Nevis, Palau, Niue, Nauru, Monaco, Cook Islands, and San Marino) as well as subnational estimates for nine countries (India, Brazil, Mexico, the UK, the USA, Sweden, Kenya, Indonesia, and Japan) were added to the GBD framework.<br>- Twelve new causes were added to the GBD framework (pulmonary arterial hypertension, eye cancer, soft tissue and other extraosseous sarcomas, malignant neoplasm of bone and articular cartilage, and neuroblastoma and other peripheral nervous cell tumours at Level 3, and hepatoblastoma, Burkitt lymphoma, other non-Hodgkin lymphoma, retinoblastoma, other eye cancers, and two sites of osteoarthritis (hand and other joints) at Level 4).<br>- Deaths attributed to multiple or unspecified causes were analyzed through more empirical redistribution algorithms.<br>- Case definitions and measurement methods were standardised through implementing network meta-regression to estimate correction factors for alternative case definition or measurement methods.<br>- Modelling strategies were modified to include a set of standard locations encompassing all of the countries, territories, and subnational locations in the GBD framework. Coefficients of covariates in the three main models were only estimated for these standard locations.<br>- Count models were added to the CODEm model; simulation testing results were incorporated in modifying the priors in the DisMod-MR model.

Appendix table 4. Change (%) in age-standardised DALYs rate from 1990 to 2019 for non-commun icable diseases in ascending order

Rank	Location	Change (%) in age-standardised DALYs rate from 1990 to 2019
**1**	**South Korea**	**-43.6% (-47.9, -39.4)**
2	Maldives	-41.4% (-47.9, -34.5)
3	Singapore	-39.4% (-43.5, -35.3)
4	Ethiopia	-34.1% (-42.4, -24.1)
5	Czechia	-33.9% (-41.1, -26.4)
6	Bahrain	-33.1% (-40.8, -24.7)
7	China	-32.9% (-39.9, -25.2)
8	Rwanda	-32.6% (-41.5, -21.5)
9	Bermuda	-31.5% (-37.8, -24.2)
10	Slovenia	-30.6% (-43, -17.5)
11	Poland	-30.5% (-36.9, -24)
12	Turkey	-29.5% (-37.6, -20.5)
13	Luxembourg	-29.2% (-34.2, -24.3)
14	Hungary	-28.8% (-36.6, -20)
15	Algeria	-28.1% (-38.1, -17.8)
16	Equatorial Guinea	-28% (-41.2, -9.9)
17	Saint Kitts and Nevis	-27.9% (-37, -18.1)
18	Portugal	-27.8% (-31.5, -24.4)
19	Estonia	-27.5% (-36.5, -17.3)
20	Ireland	-27.1% (-31.1, -23.2)
21	Slovakia	-27% (-36.1, -16.5)
22	Jordan	-26.8% (-35.1, -18.1)
23	Mauritania	-26.5% (-37.8, -13.7)
24	Greenland	-26.4% (-35.2, -17)
25	Myanmar	-26.1% (-38.4, -11.4)
26	Peru	-26.1% (-36.8, -12.8)
27	Bangladesh	-26% (-36, -14)
28	Croatia	-25.9% (-35.1, -15.3)
29	New Zealand	-25.9% (-28.8, -22.9)
30	Iran (Islamic Republic of)	-25.9% (-31.4, -20)
31	Cyprus	-25.7% (-30.5, -21.1)
32	Switzerland	-25.1% (-28.5, -21.9)
33	Austria	-25% (-28.4, -22)
34	Burundi	-24.9% (-37.5, -9.1)
35	Denmark	-24.7% (-28, -21.7)
36	Italy	-24.5% (-27.9, -21.5)
37	Brazil	-24.4% (-28.7, -19.5)
38	Norway	-24.1% (-27.6, -21)
39	Qatar	-24% (-34.5, -13)
40	Brunei Darussalam	-23.9% (-30.1, -16.9)

41	Malta	-23.8% (-29, -18.8)
42	Israel	-23.6% (-27.1, -20.5)
43	Colombia	-23.4% (-32.3, -13.2)
44	Spain	-23.3% (-26.9, -20)
45	Finland	-23.2% (-26.8, -20)
46	Australia	-23.2% (-26.8, -20.2)
47	Lao People's Democratic Republic	-23.1% (-36.3, -8)
48	Republic of Moldova	-23% (-28.9, -16.7)
49	Sudan	-22.9% (-34.4, -8.5)
50	Germany	-22.5% (-26, -19.5)
51	Congo	-22.4% (-34.6, -8.6)
52	Oman	-22.3% (-33.3, -9.8)
53	Thailand	-22.1% (-33.1, -9.4)
54	Japan	-21.9% (-25, -19.3)
55	Iceland	-21.9% (-26.4, -17.5)
56	Belgium	-21.8% (-25.2, -18.4)
57	United Arab Emirates	-21.8% (-32.2, -10.2)
58	Chile	-21.7% (-25.1, -18.2)
59	Latvia	-21.7% (-29.3, -12.5)
60	Liberia	-21.4% (-34.9, -4.2)
61	Romania	-21.4% (-29.6, -11.7)
62	United Kingdom	-21% (-24, -18.5)
63	France	-20.9% (-24.1, -18.1)
64	Syrian Arab Republic	-20.7% (-34.5, -2.9)
65	Trinidad and Tobago	-20.5% (-32.6, -6.2)
66	Netherlands	-20.5% (-23.7, -17.4)
67	Afghanistan	-20.3% (-33.1, -4.2)
68	Sweden	-20.1% (-23.1, -17.4)
69	Haiti	-19.9% (-33.4, -4.2)
70	Taiwan (Province of China)	-19.9% (-29.1, -8.6)
71	Puerto Rico	-19.6% (-28.8, -9.1)
72	Saint Lucia	-19.5% (-27.2, -11)
73	Cambodia	-19.4% (-30.1, -8.1)
74	Mauritius	-18.9% (-28.1, -8.6)
75	Kuwait	-18.5% (-25.2, -10.9)
76	Lithuania	-18.5% (-26.8, -8.6)
77	Cook Islands	-18.4% (-28.2, -7.1)
78	Sri Lanka	-18.3% (-29.9, -5)
79	Palestine	-18.3% (-29, -6)
80	Angola	-18.3% (-31.9, -1.9)
81	Serbia	-18.2% (-28.2, -6.5)
82	Argentina	-17.9% (-20.8, -15.1)
83	Bolivia (Plurinational State of)	-17.4% (-29.5, -3.3)

84	Uruguay	-17.3% (-20.7, -14)
85	India	-16.9% (-24.4, -9.1)
86	Iraq	-16.8% (-28.8, -4.3)
87	Saudi Arabia	-16.7% (-27.6, -3.9)non
88	Canada	-16.5% (-19.6, -13.9)
89	Lebanon	-16.5% (-25.4, -8.3)
90	Albania	-16.2% (-27, -3.9)
91	Yemen	-16% (-29.3, 1.4)
92	Tunisia	-15.7% (-27.5, -1.2)
93	Tuvalu	-15.6% (-30.5, 5)
94	Malawi	-15.4% (-26.8, -2)
95	Kyrgyzstan	-15.1% (-21.6, -8.2)
96	Gabon	-15% (-27.2, -2.6)
97	Guyana	-15% (-29.5, 1.8)
98	Bhutan	-14.6% (-29.4, 4.8)
99	Mongolia	-14.6% (-28.7, 2.7)
100	South Sudan	-14.5% (-26.6, 0.5)
101	Democratic Republic of the Congo	-14.5% (-27.8, 0.6)
102	Comoros	-14.4% (-29.6, 21.9)
103	Niger	-14.3% (-26.5, 1.5)
104	Guinea-Bissau	-14.3% (-30.1, 3.2)
105	Grenada	-14.2% (-20.3, -7.9)
106	Armenia	-14.2% (-22.3, -5.9)
107	Egypt	-14.2% (-26, 0.6)
108	Nepal	-14.1% (-26.7, -0.9)
109	Malaysia	-14% (-23.9, -3.1)
110	Greece	-13.9% (-16.9, -11.2)
111	Democratic People's Republic of Korea	-13.7% (-25.9, -0.7)
112	Russian Federation	-13.4% (-20.3, -5.8)
113	Mali	-13.3% (-25.8, 1.2)
114	Cuba	-13.2% (-21.7, -3.4)
115	Madagascar	-12.7% (-26, 2.6)
116	Georgia	-12.6% (-21.6, -3.1)
117	Tokelau	-12.2% (-26.1, 4)
118	Bosnia and Herzegovina	-12.2% (-22.7, 0)
119	Andorra	-12.1% (-26.3, 0.9)
120	Nigeria	-11.9% (-24.8, 3.2)
121	Bulgaria	-11.9% (-23.5, 1.9)
122	Guatemala	-11.8% (-23.7, 3.2)
123	Benin	-11.5% (-25, 3.9)
124	Côte d’Ivoire	-11.4% (-24.6, 2)
125	Senegal	-11.2% (-24.5, 4.8)
126	Somalia	-11.2% (-25.5, 7)

127	El Salvador	-10.8% (-23.2, 4.2)
128	North Macedonia	-10.6% (-21.4, 2.5)
129	Bahamas	-10.5% (-21.5, 2.2)
130	Sierra Leone	-10.4% (-26, 8.5)
131	Kiribati	-10.3% (-25.1, 6.5)
132	Belarus	-10.3% (-22.4, 5.3)
133	Panama	-10.2% (-20.5, 2)
134	Costa Rica	-10% (-20.2, 2)
135	Viet Nam	-9.9% (-22, 2.3)
136	Togo	-9.6% (-22.1, 4)
137	Samoa	-9.4% (-24.9, 10)
138	Guam	-9.3% (-18.7, 1)
139	Zambia	-9.2% (-23.2, 7.2)
140	Seychelles	-9.1% (-15.2, -2.9)
141	South Africa	-9% (-12.5, -5.3)
142	Central African Republic	-9% (-23.1, 8)
143	Libya	-8.9% (-21.4, 7.1)
144	United Republic of Tanzania	-8.6% (-19.3, 3.1)
145	Namibia	-8.6% (-22.7, 9.8)
146	Kazakhstan	-8.5% (-16, -0.7)
147	Morocco	-8.4% (-19.1, 1.7)
148	Northern Mariana Islands	-7.9% (-19.2, 2.4)
149	San Marino	-7.8% (-23.1, 11.2)
150	Barbados	-7.8% (-17.3, 2.2)
151	Antigua and Barbuda	-7.6% (-15.6, 1)
152	Venezuela (Bolivarian Republic of)	-7.6% (-20.9, 8.3)
153	Monaco	-7.5% (-18.8, 4.3)
154	Eritrea	-7.1% (-23, 13.5)
155	Ecuador	-7.1% (-16.9, 5.6)
156	Saint Vincent and the Grenadines	-6.7% (-14.7, 2)
157	Mexico	-6.7% (-14.2, 0.3)
158	United States of America	-5.9% (-8, -4.2)
159	Niue	-5.9% (-20.3, 10.6)
160	Djibouti	-5.6% (-20.8, 14.1)
161	Suriname	-5.6% (-15.5, 5.9)
162	Indonesia	-5.3% (-15.2, 3.7)
163	Guinea	-4.9% (-19.9, 11.9)
164	Ghana	-4.7% (-18, 9.7)
165	Turkmenistan	-4.3% (-17.2, 11.1)
166	Fiji	-4% (-20.7, 18.1)
167	Palau	-4% (-21.8, 14.4)
168	Cameroon	-3.8% (-18.9, 13.5)
169	Uganda	-3.7% (-16.1, 10.6)

170	Dominica	-3.4% (-14.9, 10.9)
171	Timor-Leste	-3.3% (-19.6, 12.6)
172	Sao Tome and Principe	-3.3% (-15.8, 12.4)
173	Micronesia (Federated States of)	-3.2% (-26.4, 18.7)
174	Marshall Islands	-2.1% (-17.3, 15.4)
175	Botswana	-2% (-19.9, 19)
176	Chad	-1.8% (-13.8, 12)
177	Tonga	-1.7% (-15.6, 14.8)
178	Nauru	-1.4% (-12.4, 11.9)
179	United States Virgin Islands	-1.3% (-13, 11.2)
180	Montenegro	-1.1% (-10.3, 9.6)
181	American Samoa	-1% (-11.5, 11)
182	Burkina Faso	-0.3% (-13, 13.3)
183	Azerbaijan	0.2% (-8.2, 10.5)
184	Gambia	0.3% (-17.4, 19.8)
185	Belize	0.6% (-7.7, 9.6)
186	Jamaica	1.7% (-10.3, 16)
187	Cabo Verde	1.7% (-6.2, 10.7)
188	Kenya	1.9% (-6.1, 11.2)
189	Solomon Islands	2.2% (-17.4, 23.4)
190	Papua New Guinea	2.3% (-12.6, 20.8)
191	Honduras	3% (-7.5, 18.1)
192	Paraguay	3.2% (-10.6, 19)
193	Ukraine	3.4% (-6.5, 14.9)
194	Philippines	3.6% (-7.2, 15.3)
195	Mozambique	4.4% (-11, 23)
196	Eswatini	4.7% (-14.8, 29.3)
197	Vanuatu	4.7% (-13.7, 29.6)
198	Nicaragua	5.1% (-4.9, 16.3)
199	Pakistan	6% (-4.7, 19.1)
200	Dominican Republic	6.1% (-9.4, 24.7)
201	Zimbabwe	13.5% (-1.7, 30.8)
202	Tajikistan	14.4% (0.5, 30.6)
203	Lesotho	24.8% (1.7, 50.6)
204	Uzbekistan	29% (16.6, 42.5)

Appendix table 5. Change (%) in age-standardised mortality rate from 1990 to 2019 for non-communicable diseases in ascending order

Rank	Location	Change (%) in age-standardised mortality rate from 1990 to 2019
**1**	**South Korea**	**-58.8% (-60.5, -55.9)**
2	Singapore	-56.4% (-57.8, -54.8)
3	Maldives	-48.9% (-56, -41.6)
4	Luxembourg	-47.6% (-52.4, -41.8)
5	Bermuda	-46.7% (-53.3, -36.9)
6	Czechia	-45.2% (-53.5, -35.8)
7	Portugal	-45.1% (-46.4, -43.5)
8	Ireland	-44.6% (-46.5, -42.6)
9	Slovenia	-42.9% (-58.3, -24.1)
10	Malta	-42.7% (-47.7, -37.5)
11	Cyprus	-42.5% (-46.8, -37.8)
12	Bahrain	-39.9% (-49.6, -28.8)
13	Australia	-39.4% (-40.7, -38.1)
14	Austria	-39.2% (-40.4, -38)
15	Japan	-39% (-39.8, -38.1)
16	Iceland	-38.9% (-43.6, -33.7)
17	Israel	-38.7% (-40.5, -37)
18	Spain	-38.3% (-39.5, -37.1)
19	Estonia	-38.3% (-49.8, -25.2)
20	Italy	-38.2% (-38.8, -37.6)
21	Colombia	-38.2% (-50.5, -23.3)
22	Poland	-37.9% (-46.5, -28.8)
23	China	-37.7% (-45.8, -27.7)
24	Norway	-37.7% (-39, -36.3)
25	Switzerland	-37% (-38.6, -35.4)
26	Belgium	-37% (-38.5, -35.4)
27	Jordan	-36.9% (-46.3, -25.8)
28	New Zealand	-36.8% (-38.1, -35.5)
29	Finland	-36% (-38.1, -33.8)
30	Denmark	-35.7% (-37.6, -33.7)
31	Germany	-35.7% (-36.7, -34.6)
32	France	-35.5% (-36.8, -34.2)
33	Algeria	-35.4% (-46.6, -22.8)
34	United Kingdom	-35% (-35.7, -34.3)
35	Saint Kitts and Nevis	-34.9% (-42.6, -24.8)
36	Puerto Rico	-34.5% (-47.6, -19.3)
37	Hungary	-34.4% (-44.3, -23)
38	Thailand	-34.3% (-49.4, -15.7)
39	Brazil	-34.1% (-36.4, -31.7)
40	Peru	-34.1% (-49.9, -12.8)
41	Greenland	-34% (-44.7, -22.8)

42	Rwanda	-34% (-43.6, -20.4)
43	Croatia	-33.7% (-45.4, -19.5)
44	Ethiopia	-33.6% (-43.1, -22)
45	Turkey	-33.6% (-45.2, -20.9)
46	Slovakia	-33% (-44.9, -18.8)
47	Sweden	-32.8% (-33.7, -31.7)
48	Canada	-32.6% (-33.6, -31.6)
49	Chile	-32.5% (-34.4, -30.4)
50	Taiwan (Province of China)	-32.2% (-44.9, -15.5)
51	Netherlands	-32.1% (-33.8, -30.3)
52	Iran (Islamic Republic of)	-31.9% (-35.1, -28.4)
53	United Arab Emirates	-31.8% (-42.4, -18.4)
54	Trinidad and Tobago	-31.6% (-46.6, -13.5)
55	Republic of Moldova	-30.7% (-37.5, -23.1)
56	Saint Lucia	-30.7% (-39.7, -21)
57	Mauritania	-30.7% (-42.9, -16.5)
58	Brunei Darussalam	-29.7% (-36.8, -21.6)
59	Guam	-29.7% (-39.8, -17.5)
60	Kuwait	-29.5% (-38.4, -18.7)
61	Bangladesh	-29.3% (-42.1, -13.6)
62	Mauritius	-28.4% (-40.2, -15.1)
63	Romania	-28.3% (-38.9, -16.5)
64	Greece	-27.8% (-29.5, -25.9)
65	Latvia	-27.6% (-37.4, -15.3)
66	Cook Islands	-26.7% (-37.5, -13.3)
67	Uruguay	-26.1% (-28.4, -23.6)
68	Sri Lanka	-26.1% (-42.3, -6.8)
69	Myanmar	-26% (-38.1, -10.4)
70	Equatorial Guinea	-25.6% (-43.2, -0.6)
71	Argentina	-25.4% (-27.1, -23.6)
72	Burundi	-24.7% (-39.1, -5.4)
73	Lebanon	-23.8% (-33.3, -14.8)
74	Lithuania	-23.3% (-35.1, -10.1)
75	Andorra	-23% (-43.6, -0.5)
76	Albania	-22.7% (-38.7, -4.1)
77	Congo	-22.7% (-36, -6.1)
78	India	-22.5% (-32.9, -10.9)
79	Sudan	-22.3% (-33.9, -6.7)
80	Georgia	-21.8% (-32.4, -10.1)
81	Saudi Arabia	-21.5% (-35.4, -4.5)
82	Oman	-21.2% (-33.4, -4.5)
83	Guyana	-21.1% (-37.7, -2.2)
84	Lao People's Democratic Republic	-20.7% (-35.6, -2.8)

85	Qatar	-20.6% (-34.7, -4.4)
86	Haiti	-20% (-35.6, 0.3)
87	Palestine	-19.6% (-33.5, -0.8)
88	Cuba	-19.5% (-32.4, -5)
89	Russian Federation	-19.3% (-27.6, -9.8)
90	United States of America	-19.1% (-19.7, -18.4)
91	Panama	-18.9% (-35.3, 0.2)
92	Costa Rica	-18.7% (-34.4, 1.2)
93	Grenada	-18.4% (-25.1, -11.3)
94	San Marino	-18% (-43.6, 15.2)
95	Liberia	-17.9% (-32.9, 2.4)
96	Tunisia	-17.8% (-35.6, 4.9)
97	Malaysia	-17.7% (-32.1, -1.9)
98	Afghanistan	-17.5% (-33.7, -1.7)
99	Syrian Arab Republic	-17.1% (-35.6, 8.4)
100	Angola	-16.7% (-32.4, 5.6)
101	Armenia	-16.2% (-27.2, -4.6)
102	Yemen	-16.2% (-30.6, 5)
103	Bulgaria	-16.2% (-30, 0.2)
104	South Sudan	-16.1% (-32.1, 2.8)
105	Comoros	-15.8% (-32.1, 21.6)
106	Bahamas	-15.5% (-29.3, 0.8)
107	Tokelau	-15.3% (-31, 3.3)
108	Cambodia	-15.2% (-29.4, -3.4)
109	Monaco	-14.9% (-31.3, 5)
110	Democratic Republic of the Congo	-14.9% (-30.6, 4.4)
111	Côte d'Ivoire	-14.9% (-28.9, 0.7)
112	Tuvalu	-14.8% (-32.4, 9.3)
113	Iraq	-14.7% (-29.7, 0.9)
114	Nigeria	-14.4% (-31.3, 6.5)
115	Gabon	-14.2% (-29.4, 0.3)
116	Mali	-14.1% (-28.1, 2.5)
117	Serbia	-13.9% (-27.5, 1.6)
118	Democratic People's Republic of Korea	-13.9% (-27.3, 1.2)
119	Malawi	-13.1% (-25.8, 3.4)
120	Bolivia (Plurinational State of)	-13.1% (-28.4, 5.9)
121	Seychelles	-12.8% (-20.4, -4.9)
122	Benin	-12.8% (-27, 5.5)
123	Niger	-12.8% (-27.3, 5.3)
124	Egypt	-12.8% (-28.2, 5)
125	Bosnia and Herzegovina	-12.8% (-28.1, 4.8)
126	Samoa	-12.5% (-28.5, 8.2)
127	Mongolia	-12.3% (-28.5, 8.5)

128	Barbados	-12.2% (-24.9, 0.2)
129	Northern Mariana Islands	-12% (-24.4, -1.4)
130	Guinea-Bissau	-11.8% (-29.1, 9.8)
131	Guatemala	-11.7% (-28, 9.4)
132	Viet Nam	-11.5% (-25.4, 3.1)
133	Suriname	-11.1% (-23.4, 3)
134	Mexico	-10.9% (-21.2, 0)
135	Venezuela (Bolivarian Republic of)	-10.7% (-29.4, 12.6)
136	Somalia	-10.6% (-27.7, 13.1)
137	Saint Vincent and the Grenadines	-10.6% (-20.1, -0.1)
138	Belarus	-10.4% (-26.9, 11.1)
139	Senegal	-10.4% (-24.8, 9.5)
140	Bhutan	-10.3% (-27.8, 15.5)
141	Togo	-10.1% (-24.5, 8.7)
142	Sierra Leone	-9.8% (-25.4, 12.7)
143	Libya	-9.7% (-26.8, 14)
144	Namibia	-9.3% (-25.7, 12.4)
145	Central African Republic	-9.2% (-26.9, 12.8)
146	Niue	-9.1% (-24.5, 9.5)
147	Turkmenistan	-9% (-24.1, 9.8)
148	Kyrgyzstan	-9% (-18.3, 1.2)
149	Nepal	-8.8% (-28.2, 10.5)
150	Dominica	-8.7% (-22.4, 8)
151	Antigua and Barbuda	-7.9% (-18, 3.4)
152	Madagascar	-7.7% (-26.4, 13.6)
153	United Republic of Tanzania	-7.6% (-19.7, 6.7)
154	El Salvador	-7.6% (-27.4, 16)
155	Kiribati	-7.5% (-24, 11.7)
156	American Samoa	-6.4% (-18.3, 7.1)
157	Palau	-6.3% (-27, 16.2)
158	Kazakhstan	-5.8% (-15.3, 4.8)
159	Cameroon	-5.8% (-23, 16.1)
160	Zambia	-5.6% (-22.7, 15.7)
161	Morocco	-5.2% (-21.2, 6.8)
162	Ghana	-5.1% (-20.4, 12.8)
163	Marshall Islands	-5.1% (-22.9, 15)
164	Fiji	-4.9% (-24.3, 21.5)
165	Ecuador	-4.4% (-21.1, 16.7)
166	North Macedonia	-4% (-18.8, 13.1)
167	Uganda	-3.8% (-18.9, 14.1)
168	Djibouti	-3.7% (-21.6, 21.5)
169	Belize	-3.5% (-14.4, 9.4)
170	Tonga	-3.3% (-20, 17.1)

171	Nauru	-2.1% (-13.8, 12.4)
172	Botswana	-2% (-24.2, 26.3)
173	Guinea	-1.9% (-20.7, 18.8)
174	Micronesia (Federated States of)	-1.1% (-24, 24.1)
175	United States Virgin Islands	-1% (-14.4, 14)
176	Jamaica	-1% (-18.1, 19.2)
177	Chad	0.2% (-15.1, 19.8)
178	Eritrea	0.3% (-23.2, 34)
179	Philippines	0.5% (-14.7, 17.6)
180	South Africa	0.6% (-4.8, 6.2)
181	Burkina Faso	0.8% (-14.2, 17.7)
182	Ukraine	0.9% (-11, 14.9)
183	Vanuatu	3.2% (-16.9, 31.2)
184	Sao Tome and Principe	3.7% (-12.2, 20.5)
185	Papua New Guinea	4.1% (-14.3, 26.3)
186	Gambia	4.6% (-18.7, 30.3)
187	Paraguay	4.8% (-17.4, 31.2)
188	Solomon Islands	5.1% (-15.3, 28.2)
189	Montenegro	5.1% (-8.2, 20.8)
190	Indonesia	6.1% (-9.4, 18.9)
191	Eswatini	6.7% (-18.5, 39)
192	Kenya	7.3% (-4.2, 21.8)
193	Timor-Leste	7.8% (-13.3, 27.9)
194	Pakistan	7.9% (-7.6, 27.5)
195	Mozambique	10.1% (-10.6, 34.9)
196	Zimbabwe	14.7% (-5.5, 38.7)
197	Dominican Republic	15.6% (-7, 42.7)
198	Cabo Verde	18.4% (7, 30.7)
199	Azerbaijan	24.6% (12.1, 38.5)
200	Nicaragua	26.2% (9.1, 42.1)
201	Honduras	26.5% (12.4, 47.2)
202	Lesotho	35.5% (3.2, 72.1)
203	Tajikistan	41.9% (20.7, 66.3)
204	Uzbekistan	67.4% (49.6, 86.2)

Appendix table 6. Number and age-standardised rate of prevalence and incidence, and percentage change from 1990 to 2019 for both sex by causes

Causes	Prevalence (95% UI)					Incidence (95% UI)				
	Counts 1990	Counts 2019	Age-standardised estimates 1990	Age-standardised estimates 2019	Percentage change of age-standardised rates between 1990 to 2019 (%)	Counts 1990	Counts 2019	Age-standardised estimates 1990	Age-standardised estimates 2019	Percentage change of age-standardised rates between 1990 to 2019 (%)
All causes	42093965·44 (41786003·78 to 42362431·77)	51057699·25 (50800046·62 to 51300453·95)	94617·13 (93877·26 to 95254·56)	92183 (91276·34 to 92998·05)	-2·57 (-3·08 to -2·07)	221502813·2 (206079109·1 to 239990516·5)	254526791·6 (240220412·5 to 271529504·9)	501773·37 (468100·64 to 540466·14	498757·83 (466805·03 to 538359·25	-0·6 (-2·98 to 1·9)
Communicable, maternal, neonatal, and nutritional diseases	29141375·55 (28338121·46 to 30034831·97)	24218774·77 (22855824·86 to 25584631·7)	65204·3 (63476·76 to 67104·13)	40213·98 (37886·65 to 42461·63)	-38·33 (-41·73 to -34·93)	143576238·6 (129397833 to 161216212·1)	158813394·6 (145057413 to 175207736·1)	324024·35 (292144·71 to 362214·27	320775·11 (289196·74 to 358836·05	-1 (-4·72 to 2·95)
HIV/AIDS and sexually transmitted infections	8613409·33 (8311922·63 to 8912452·64)	9553234·24 (8342340·63 to 10882454·94)	19783·8 (19146·3 to 20428·83)	13278·69 (11582·68 to 15121·16)	-32·88 (-40·49 to -25·34)	3009359·31 (2586286·85 to 3523873·88)	3481691·79 (2934273·53 to 4066843·43)	6052·32 (5203·47 to 7038·71	5738·55 (4848·98 to 6755·41	-5·18 (-8·73 to -1·54)
HIV/AIDS	2704·03 (0 to 4813·91)	29976·86 (17559·23 to 47045·64)	5·61 (0 to 9·9)	40·71 (24·3 to 61·75)	625·62 (0 to 785·44)	344·23 (0 to 1091·33)	1257·55 (317·94 to 2409·23)	0·71 (0 to 2·41	2·08 (0·77 to 3·65	191·57 (-59·45 to 524·89)
HIV/AIDS - drug-susceptible tuberculosis	551·45 (0 to 713·29)	400·88 (332·46 to 480·15)	1·15 (0 to 1·49)	0·54 (0·45 to 0·65)	-52·72 (-65·66 to 0)	587·5 (0 to 765·81)	598·56 (509·66 to 705·92)	1·23 (0 to 1·6	0·81 (0·69 to 0·96	-33·74 (-51·03 to 0)
HIV/AIDS– multidrug-resistant tuberculosis without extensive drug resistance	3·39 (0 to 12·4)	6·65 (0·95 to 26·08)	0·01 (0 to 0·03)	0·01 (0 to 0·04)	27·51 (-89·29 to 727·18)	3·75 (0 to 13·67)	10·7 (1·49 to 42·31)	0·01 (0 to 0·03	0·01 (0 to 0·06	85·8 (-84·03 to 1103·45)
HIV/AIDS - extensively drug-resistant tuberculosis	0 (0 to 0)	0·84 (0·12 to 3·28)	0 (0 to 0)	0 (0 to 0)	0 (0 to 0)	0 (0 to 0)	1·35 (0·19 to 5·31)	0 (0 to 0	0 (0 to 0·01	0 (0 to 0)
HIV/AIDS resulting in other diseases	2149·19 (0 to 4184·56)	29568·49 (17121·63 to 46679·61)	4·45 (0 to 8·48)	40·15 (23·76 to 61·24)	802·01 (0 to 1087·26)	344·23 (0 to 1091·33)	1257·55 (317·94 to 2409·23)	0·71 (0 to 2·41	2·08 (0·77 to 3·65	191·57 (-59·45 to 524·89)
Sexually transmitted infections excluding HIV	8613209·29 (8311886·47 to 8912123·79)	9533287·8 (8313997·59 to 10859076·57)	19783·46 (19145·56 to 20428·45)	13253·03 (11547·83 to 15096·46)	-33·01 (-40·67 to -25·5)	3009015·07 (2586072·43 to 3523650·32)	3480434·25 (2933526·68 to 4065490·92)	6051·6 (5203·05 to 7038·7	5736·47 (4847·12 to 6752·87	-5·21 (-8·74 to -1·56)
Syphilis	116048·59 (89402·13 to 152931·81)	158977·44 (121241·51 to 211780·69)	235·57 (183·8 to 307·49)	268·46 (202·68 to 364·37)	13·96 (3·03 to 28·03)	36729·03 (28095·32 to 48154·26)	43964·44 (33237·13 to 58541·04)	71·63 (55·42 to 92·91	81·34 (61·08 to 109·56	13·55 (3·43 to 26·66)
Chlamydial infection	365995·99 (284252·4 to 465374·33)	421430·29 (327860·84 to 534787·46)	715·01 (555·49 to 906·79)	741·64 (575·2 to 957·96)	3·72 (-2·04 to 9·64)	520938·03 (388609·18 to 687453·31)	607255·87 (454178·55 to 786810·94)	1011·99 (765·86 to 1313·84	1073·54 (797·57 to 1418·66	6·08 (-0·19 to 12·17)

**Gonococcal infection**	176676·24 (130910·59 to 245034·52)	159238·18 (122471·44 to 210204·05)	343·1 (258·78 to 467·24)	311·96 (232·91 to 428·35)	-9·08 (-15·52 to -2·8)	331431·67 (232897·57 to 479246·39)	307453·49 (227847·18 to 418237·47)	636·36 (457·28 to 906·04	613·57 (438·53 to 861·29	-3·58 (-10·97 to 3·97)
**Trichomoniasis**	658313·11 (491832·03 to 868400·27)	890264·05 (685985·62 to 1131407·75)	1410·33 (1094·06 to 1823·87)	1335·13 (1025·29 to 1725·09)	-5·33 (-12·6 to 2·85)	1430664·28 (1052201·53 to 1931379·24)	2067722·19 (1533491·05 to 2641707·02)	3038·35 (2266·47 to 3974·08	3093·46 (2297·32 to 4040·52	1·81 (-3·47 to 7·19)
**Genital herpes**	7782342·36 (7513405·12 to 8051138·18)	8270422·01 (6968274·93 to 9733730·05)	18164·22 (17550·81 to 18755·2)	11102·38 (9292·11 to 13116·72)	-38·88 (-47·66 to -29·74)	689252·07 (649862·84 to 727631·03)	454038·26 (388207·47 to 537856·19)	1293·27 (1220·03 to 1369·25	874·57 (734·58 to 1033·17	-32·38 (-41·53 to -22·59)
**Other sexually transmitted infections**	121165·93 (80050·17 to 161317·07)	126628·33 (78714·27 to 172240·24)	247·95 (160·51 to 329·06)	208·48 (134·4 to 278·47)	-15·92 (-25·36 to -5·15)	0 (0 to 0)	0 (0 to 0)	0 (0 to 0	0 (0 to 0	0 (0 to 0)
**Respiratory infections and tuberculosis**	18762520·06 (17702423·55 to 19733187·83)	11692213·33 (10425090·78 to 13351159·45)	41472·91 (39232·73 to 43494·91)	19182·24 (17351·93 to 21431·29)	-53·75 (-57·56 to -49·29)	129198448·1 (114611077·2 to 146961009·4)	137715261·7 (124007647·8 to 154251068·2)	290295·01 (258785·09 to 327070·36	289662·17 (258236·25 to 328787·12	-0·22 (-4·22 to 4·33)
**Tuberculosis**	17373980·67 (16310778·78 to 18397334·01)	9842950·09 (8510424·9 to 11497801·22)	38286·29 (35988·03 to 40396·82)	15097·01 (13266·06 to 17416·47)	-60·57 (-64·78 to -55·56)	48626·98 (43369·54 to 54219·21)	29738·45 (25567·04 to 34764·96)	127·92 (115·16 to 141·57	39·69 (34·34 to 46·04	-68·98 (-72·5 to -64·56)
**Latent tuberculosis infection**	17328358·65 (16268841·78 to 18347379·26)	9823055·69 (8493210·98 to 11476899·28)	38165·4 (35869·23 to 40269·26)	15070·47 (13240·81 to 17385·85)	-60·51 (-64·73 to -55·49)	NA	NA	NA	NA	NA
**Drug-susceptible tuberculosis**	45389·17 (40566·33 to 50182·12)	19596·6 (16304·05 to 23484·07)	120·27 (107·53 to 133·76)	26·15 (21·98 to 30·99)	-78·26 (-80·95 to -75·15)	48370 (43095·55 to 53806·04)	29259·51 (24999·39 to 34470·18)	127·25 (114·75 to 140·52	39·05 (33·53 to 45·31	-69·31 (-72·98 to -64·96)
**Multidrug-resistant tuberculosis without extensive drug resistance**	232·85 (42·06 to 788·99)	264·54 (38·32 to 1054·47)	0·62 (0·11 to 2·09)	0·35 (0·05 to 1·4)	-42·82 (-94·42 to 399·31)	256·98 (45·61 to 889·88)	425·45 (62·22 to 1637·04)	0·68 (0·12 to 2·33	0·57 (0·08 to 2·25	-16·02 (-91·84 to 667·4)
**Extensively drug-resistant tuberculosis**	0 (0 to 0)	33·26 (4·82 to 132·59)	0 (0 to 0)	0·04 (0·01 to 0·18)	0 (0 to 0)	0 (0 to 0)	53·49 (7·82 to 205·84)	0 (0 to 0	0·07 (0·01 to 0·28	0 (0 to 0)
**Lower respiratory infections**	25177·66 (22662·94 to 27931·5)	20873·93 (19138 to 22702·19)	64·31 (58·49 to 70·98)	45·06 (39·99 to 50·7)	-29·93 (-34·4 to -25·47)	1092719·98 (971546·01 to 1214387·7)	970097·41 (887785·93 to 1056905·57)	2817·86 (2533·14 to 3113·64	2036·41 (1799·39 to 2292·82	-27·73 (-32·16 to -23·24)
**Upper respiratory infections**	1747796·94 (1549254·92 to 1993762·71)	1866811·82 (1678347·48 to 2094243·73)	3918·16 (3489·33 to 4430·48)	3911·45 (3475·42 to 4445·23)	-0·17 (-4·15 to 4·44)	126841275·5 (112268623·8 to 144709051·6)	135538031·5 (121856848·9 to 152169585·4)	284327·44 (252952·09 to 321482·26	283844·59 (252086·41 to 323092·26	-0·17 (-4·23 to 4·45)
**Otitis media**	502794·51 (413705·65 to 634651·95)	396617·83 (331736·19 to 478099·48)	1136·31 (945·72 to 1443·94)	864·05 (738·89 to 1035·89)	-23·96 (-38·45 to -14·83)	1215825·7 (908367·41 to 1574600·92)	1177394·41 (884749·03 to 1513762·18)	3021·79 (2202 to 4009·04	3741·48 (2526·05 to 5146·55	23·82 (11·87 to 38·13)
**Enteric infections**	129574·7 (113893·25 to 146436·85)	247632·08 (218184·98 to 278437·34)	321·98 (285·42 to 362·87)	323·83 (288·14 to 361·9)	0·57 (-9·48 to 12·18)	8560994·67 (7530018·65 to 9683220·6)	15365748·16 (13485541·93 to 17418145·99)	21108·61 (18635·83 to 23795·38	20399·01 (18027·01 to 23088·7	-3·36 (-11·93 to 5·44)

**Diarrheal diseases**	130913·14 (116767·83 to 147929·18)	248957·29 (220997·57 to 277859·42)	324·77 (290·54 to 365·05)	327·14 (291·79 to 363·45)	0·73 (-8·76 to 10·98)	8560854·64 (7529864·81 to 9683093·92)	15365598·64 (13485416·22 to 17417921·03)	21108·28 (18635·43 to 23795·09	20398·69 (18026·6 to 23088·46	-3·36 (-11·93 to 5·44)
**Typhoid and paratyphoid**	0 (0 to 0)	0 (0 to 0)	0 (0 to 0)	0 (0 to 0)	0 (0 to 0)	5·62 (3·11 to 9·13)	6·45 (3·92 to 9·97)	0·01 (0·01 to 0·02	0·01 (0·01 to 0·02	-4·87 (-20·14 to 2·79)
**Typhoid fever**	0·08 (0·03 to 0·14)	0·09 (0·04 to 0·17)	0 (0 to 0)	0 (0 to 0)	6·19 (-21·47 to 41·16)	1·19 (0·49 to 2·34)	1·48 (0·64 to 2·68)	0 (0 to 0·01	0 (0 to 0·01	6·52 (-17·98 to 37·41)
**Paratyphoid fever**	0·19 (0·11 to 0·32)	0·22 (0·13 to 0·35)	0 (0 to 0)	0 (0 to 0)	-7·89 (-24·16 to 6·8)	4·43 (2·41 to 7·29)	4·97 (3·02 to 7·87)	0·01 (0·01 to 0·02	0·01 (0·01 to 0·02	-7·82 (-22·44 to 2·4)
**Invasive non-typhoidal Salmonella (iNTS)**	3·07 (0·4 to 8·71)	3·26 (0·43 to 9·67)	0·01 (0 to 0·02)	0·01 (0 to 0·02)	-0·1 (-0·58 to 0·35)	134·4 (80·67 to 202·7)	143·06 (85·01 to 219·16)	0·31 (0·19 to 0·45	0·31 (0·19 to 0·45	-0·09 (-0·58 to 0·35)
**Other intestinal infectious diseases**	NA	NA	NA	NA	NA	NA	NA	NA	NA	NA
**Neglected tropical diseases and malaria**	5303861·52 (4139619·28 to 6823062·59)	2984862·39 (2454250·95 to 3660647·94)	11634·87 (9168·01 to 14811)	5831·53 (4642·59 to 7351·95)	-49·88 (-64·01 to -31·58)	26263·5 (8578·19 to 63324·52)	573·99 (98·54 to 2090·18)	57·46 (18·77 to 138·59	0·96 (0·16 to 3·51	-98·33 (-99·74 to -90·75)
**Malaria**	62085·47 (37862·89 to 96862·02)	8008·56 (3495·83 to 16431·95)	144·65 (88·01 to 225·92)	19·19 (8·38 to 39·37)	-86·74 (-94·48 to -67·91)	26238·37 (8557·68 to 63298·81)	545·41 (70·99 to 2058·04)	57·4 (18·72 to 138·53	0·92 (0·12 to 3·46	-98·4 (-99·79 to -90·87)
**Chagas disease**	0 (0 to 0)	0 (0 to 0)	0 (0 to 0)	0 (0 to 0)	0 (0 to 0)	0 (0 to 0)	0 (0 to 0)	0 (0 to 0	0 (0 to 0	0 (0 to 0)
**Leishmaniasis**	0 (0 to 0)	0 (0 to 0)	0 (0 to 0)	0 (0 to 0)	0 (0 to 0)	0 (0 to 0)	0 (0 to 0)	0 (0 to 0	0 (0 to 0	0 (0 to 0)
**Visceral leishmaniasis**	0 (0 to 0)	0 (0 to 0)	0 (0 to 0)	0 (0 to 0)	0 (0 to 0)	0 (0 to 0)	0 (0 to 0)	0 (0 to 0	0 (0 to 0	0 (0 to 0)
**Cutaneous and mucocutaneous leishmaniasis**	0 (0 to 0)	0 (0 to 0)	0 (0 to 0)	0 (0 to 0)	0 (0 to 0)	0 (0 to 0)	0 (0 to 0)	0 (0 to 0	0 (0 to 0	0 (0 to 0)
**African trypanosomiasis**	0 (0 to 0)	0 (0 to 0)	0 (0 to 0)	0 (0 to 0)	0 (0 to 0)	0 (0 to 0)	0 (0 to 0)	0 (0 to 0	0 (0 to 0	0 (0 to 0)
**Schistosomiasis**	0 (0 to 0)	0 (0 to 0)	0 (0 to 0)	0 (0 to 0)	0 (0 to 0)	NA	NA	NA	NA	NA

**Cysticercosis**	21803·01 (15223·31 to 30455·76)	42445·28 (29047·65 to 61407·17)	65·12 (46·71 to 88·82)	49·58 (34·38 to 70·28)	-23·87 (-33·03 to -13·18)	NA	NA	NA	NA	NA
**Cystic echinococcosis**	63·01 (44·23 to 89·63)	86·71 (63·29 to 115·07)	0·14 (0·1 to 0·19)	0·14 (0·1 to 0·19)	-0·56 (-6·65 to 5·97)	13·62 (9·83 to 18·44)	18·32 (13·92 to 24·03)	0·03 (0·02 to 0·04	0·03 (0·02 to 0·04	-0·62 (-6·38 to 5·49)
**Lymphatic filariasis**	0 (0 to 0)	0 (0 to 0)	0 (0 to 0)	0 (0 to 0)	0 (0 to 0)	NA	NA	NA	NA	NA
**Onchocerciasis**	0 (0 to 0)	0 (0 to 0)	0 (0 to 0)	0 (0 to 0)	0 (0 to 0)	NA	NA	NA	NA	NA
**Trachoma**	0 (0 to 0)	0 (0 to 0)	0 (0 to 0)	0 (0 to 0)	0 (0 to 0)	NA	NA	NA	NA	NA
**Dengue**	0 (0 to 0)	0 (0 to 0)	0 (0 to 0)	0 (0 to 0)	0 (0 to 0)	0 (0 to 0)	0 (0 to 0)	0 (0 to 0	0 (0 to 0	0 (0 to 0)
**Yellow fever**	0 (0 to 0)	0 (0 to 0)	0 (0 to 0)	0 (0 to 0)	0 (0 to 0)	0 (0 to 0)	0 (0 to 0)	0 (0 to 0	0 (0 to 0	0 (0 to 0)
**Rabies**	0·06 (0 to 0·09)	0·01 (0 to 0·01)	0 (0 to 0)	0 (0 to 0)	-95·68 (-97·9 to -10·7)	1·47 (0·02 to 2·44)	0·15 (0·03 to 0·28)	0 (0 to 0·01	0 (0 to 0	-95·68 (-97·9 to -10·7)
**Intestinal nematode infections**	4208371·5 (3012815·85 to 5755184·02)	1716556·16 (1213399·23 to 2381352·68)	8950·69 (6434·2 to 12254·45)	3921·24 (2760·4 to 5486·03)	-56·19 (-72·19 to -31·32)	NA	NA	NA	NA	NA
**Ascariasis**	1023922·54 (599299·93 to 1585609·99)	161863·77 (96887·99 to 256898·7)	2267·39 (1327·1 to 3511·2)	343·88 (205·84 to 545·78)	-84·83 (-92·3 to -70·86)	NA	NA	NA	NA	NA
**Trichuriasis**	3016406·12 (1907237·29 to 4552049·4)	1314016·16 (816179·46 to 1979540·38)	6304·4 (3986·2 to 9513·95)	3066·99 (1905·01 to 4620·36)	-51·35 (-73 to -8·29)	NA	NA	NA	NA	NA
**Hookworm disease**	276792·03 (180901·03 to 401491·05)	258686·75 (172708·43 to 377598·81)	610·16 (398·78 to 885·05)	554·06 (369·89 to 808·66)	-9·19 (-48·39 to 61·61)	NA	NA	NA	NA	NA
**Food-borne trematodiases**	701910·67 (680757·56 to 723546·41)	1073816·87 (954280·12 to 1197857·91)	1775·46 (1722·83 to 1830·49)	1494·79 (1335·75 to 1653·83)	-15·81 (-23·84 to -7·94)	NA	NA	NA	NA	NA
**Leprosy**	193·21 (152·8 to 240·89)	246·84 (197·27 to 306·55)	0·5 (0·4 to 0·62)	0·31 (0·25 to 0·39)	-37·92 (-43·42 to -30·65)	10·04 (8·31 to 12·11)	10·1 (8·28 to 12·3)	0·02 (0·02 to 0·03	0·01 (0·01 to 0·02	-40·73 (-44·99 to -35·85)

**Ebola virus disease**	0 (0 to 0)	0 (0 to 0)	0 (0 to 0)	0 (0 to 0)	0 (0 to 0)	0 (0 to 0)	0 (0 to 0)	0 (0 to 0	0 (0 to 0	0 (0 to 0)
**Zika virus disease**	0 (0 to 0)	0 (0 to 0)	0 (0 to 0)	0 (0 to 0)	0 (0 to 0)	0 (0 to 0)	0 (0 to 0)	0 (0 to 0	0 (0 to 0	0 (0 to 0)
**Guinea worm disease**	0 (0 to 0)	0 (0 to 0)	0 (0 to 0)	0 (0 to 0)	0 (0 to 0)	0 (0 to 0)	0 (0 to 0)	0 (0 to 0	0 (0 to 0	0 (0 to 0)
**Other neglected tropical diseases**	434203·98 (402913·37 to 468773·11)	201203·85 (172326·49 to 234376·78)	981·06 (908·83 to 1059·11)	443·79 (370·45 to 534·18)	-54·76 (-62·75 to -45·11)	NA	NA	NA	NA	NA
**Other infectious diseases**	608730·41 (571939·74 to 649994·15)	360664·15 (326023·19 to 398982·81)	1372·02 (1293·26 to 1456·05)	698·16 (630·14 to 776·17)	-49·11 (-54·53 to -42·66)	2163290·46 (2000784·3 to 2353764·26)	1748280·14 (1621953·32 to 1889467·65)	5124·04 (4763·09 to 5504·92	3915·11 (3663·82 to 4181·81	-23·59 (-29·12 to -18·51)
**Meningitis**	60604·36 (49526·92 to 74549·97)	36844·25 (29907·88 to 45893·47)	135·46 (110·28 to 166·92)	60·66 (49·37 to 75·3)	-55·22 (-63·45 to -46·35)	18826·69 (14983·06 to 23498·27)	10428·69 (8127·63 to 13204·78)	43·78 (35·53 to 53·79	26·7 (20·59 to 33·96	-39·02 (-44·26 to -33·57)
**Encephalitis**	9552·02 (7291·18 to 12050·7)	10512·22 (8089·26 to 13036·45)	21·33 (16·12 to 27·02)	17·43 (13·59 to 21·58)	-18·28 (-23·33 to -12·51)	2785·46 (2293·46 to 3349·16)	3021·9 (2625·56 to 3440·18)	6·82 (5·6 to 8·17	7·1 (5·87 to 8·41	4·17 (-0·58 to 10·31)
**Diphtheria**	1·44 (0·9 to 2·17)	0·11 (0·06 to 0·18)	0 (0 to 0·01)	0 (0 to 0)	-93·59 (-96·59 to -87·61)	19·15 (11·97 to 28·92)	1·42 (0·85 to 2·34)	0·04 (0·03 to 0·07	0 (0 to 0	-93·59 (-96·59 to -87·61)
**Whooping cough**	17925·09 (13663·94 to 22884·31)	2888·56 (2178·99 to 3748·95)	52·19 (39·78 to 66·63)	13·08 (9·87 to 16·98)	-74·94 (-77·66 to -72·03)	130853·13 (99746·75 to 167055·45)	21086·45 (15906·64 to 27367·33)	381 (290·43 to 486·4	95·48 (72·03 to 123·92	-74·94 (-77·66 to -72·03)
**Tetanus**	39·26 (25·52 to 56·42)	17·57 (9·57 to 29·19)	0·1 (0·06 to 0·13)	0·03 (0·02 to 0·06)	-65·29 (-75·89 to -55·27)	130·58 (91·49 to 180·45)	28·4 (21·95 to 37·66)	0·43 (0·3 to 0·59	0·05 (0·04 to 0·07	-87·59 (-91·11 to -81·55)
**Measles**	93·55 (90·41 to 96·94)	9·2 (8·16 to 10·18)	0·26 (0·26 to 0·27)	0·04 (0·04 to 0·05)	-84·64 (-86·43 to -82·92)	3414·69 (3299·91 to 3538·49)	335·83 (298·02 to 371·57)	9·67 (9·34 to 10·02	1·49 (1·32 to 1·64	-84·64 (-86·43 to -82·92)
**Varicella and herpes zoster**	31597·56 (24930·9 to 37716·47)	59162·3 (47922·65 to 71246·03)	81·72 (65·92 to 97·31)	92·62 (78·86 to 107·68)	13·34 (2·62 to 45·5)	481538·39 (433397·34 to 524695·98)	549525·52 (479467·43 to 627388·56)	1276·92 (1171·99 to 1370·74	1342·32 (1248·95 to 1443·49	5·12 (0·9 to 16·61)
**Acute hepatitis**	145398·77 (128345·34 to 165267·19)	110722·42 (100380·67 to 124710·57)	321·14 (287·99 to 358·38)	216·25 (195·98 to 237·43)	-32·66 (-40·53 to -24·86)	1525722·38 (1367782·03 to 1698836·77)	1163851·93 (1068471·09 to 1295039·74)	3405·38 (3097·14 to 3750·9	2441·97 (2206·24 to 2676·58	-28·29 (-35·58 to -21·29)
**Acute hepatitis A**	57333·26 (52289·45 to 62970·64)	43527·47 (39350·7 to 47889·17)	134·2 (121·09 to 148·88)	122·14 (105·91 to 138·83)	-8·99 (-14·59 to -3·43)	745332·32 (679762·82 to 818618·29)	565857·13 (511559·09 to 622559·18)	1744·66 (1574·2 to 1935·5	1587·81 (1376·88 to 1804·78	-8·99 (-14·59 to -3·43)

**Acute hepatitis B**	82134·94 (66379·56 to 101360·81)	61021·96 (51335·32 to 73917·1)	172·36 (142·43 to 208·44)	81·69 (69·62 to 97·3)	-52·61 (-62·26 to -40·89)	711836·14 (575289·52 to 878460·39)	528857·02 (444906·1 to 640614·88)	1493·81 (1234·41 to 1806·45	707·97 (603·39 to 843·25	-52·61 (-62·26 to -40·89)
**Acute hepatitis C**	2000·46 (1786·09 to 2239·02)	2580·7 (2143·08 to 3096·2)	5·29 (4·7 to 5·97)	3·63 (3·13 to 4·24)	-31·46 (-36·38 to -24·86)	17337·36 (15479·48 to 19404·81)	22366·06 (18573·36 to 26833·76)	45·85 (40·76 to 51·76	31·42 (27·12 to 36·72	-31·46 (-36·38 to -24·86)
**Acute hepatitis E**	3930·12 (3177·6 to 4786·47)	3592·28 (2973·76 to 4271·76)	9·28 (7·52 to 11·28)	8·8 (7·08 to 10·68)	-5·2 (-12·44 to 1·59)	51216·56 (41435·03 to 62328·88)	46771·72 (38755·63 to 55632·58)	121·06 (98·03 to 147·07	114·76 (92·38 to 139·27	-5·2 (-12·45 to 1·6)
**Other unspecified infectious diseases**	387540·14 (361716·03 to 415030·98)	186367·17 (159350 to 217508·43)	859·95 (802·83 to 920·67)	378·94 (320·09 to 445·15)	-55·93 (-63·33 to -47·04)	0 (0 to 0)	0 (0 to 0)	0 (0 to 0	0 (0 to 0	0 (0 to 0)
**Maternal and neonatal disorders**	312472·18 (273412·96 to 357093·88)	412912·52 (367936·56 to 468617·01)	707·68 (620·72 to 806·06)	898·57 (812·53 to 1006·34)	26·97 (10·25 to 44·38)	288116·14 (252063·43 to 332055·74)	154265·31 (137832·5 to 173929·41)	571·95 (508·23 to 647·96	410·44 (376·37 to 451·46	-28·24 (-34·15 to -21·2)
**Maternal disorders**	22389·89 (15989·88 to 30738·75)	13937·03 (9532·45 to 19004·31)	38·83 (27·72 to 53·27)	27·25 (18·51 to 37·17)	-29·83 (-49·65 to -3·04)	235971·67 (198780·47 to 280697·57)	120529·92 (104789·34 to 140071·55)	411·13 (346·69 to 488·58	239·27 (208·15 to 278·84	-41·8 (-48·53 to -33·66)
**Maternal hemorrhage**	18117·8 (12352·5 to 26112·24)	13305·77 (9298·4 to 18351·04)	31·55 (21·59 to 45·42)	25·96 (18·04 to 35·8)	-17·7 (-39·45 to 8·6)	17726·14 (12116·23 to 25486·81)	13022·2 (9096·65 to 17934·19)	30·87 (21·18 to 44·29	25·41 (17·62 to 35·08	-17·68 (-39·45 to 8·6)
**Maternal sepsis and other maternal infections**	4089·93 (2209·92 to 6866·26)	1924·77 (1220·77 to 2880·37)	7·27 (4·01 to 12·1)	3·62 (2·19 to 5·5)	-50·19 (-61·67 to -34·17)	45778·75 (31532 to 65253·47)	17217·03 (12335·51 to 23156·38)	79·78 (54·91 to 113·69	34·94 (24·97 to 46·99	-56·21 (-67·58 to -40·81)
**Maternal hypertensive disorders**	1369·42 (728·43 to 2282·8)	819·13 (445·91 to 1363·4)	2·4 (1·29 to 3·99)	1·58 (0·86 to 2·59)	-34·26 (-59·13 to 7·26)	12077·36 (9474 to 15171·75)	8830·51 (7153·35 to 11063·12)	21·19 (16·68 to 26·53	17·03 (13·87 to 21·42	-19·6 (-34·03 to -0·88)
**Maternal obstructed labor and uterine rupture**	618·49 (350·15 to 1009·27)	379·41 (210·45 to 613·29)	1·08 (0·61 to 1·76)	0·74 (0·41 to 1·19)	-31·15 (-47·67 to -11·42)	44888·99 (31677·17 to 62897·49)	27550·02 (19218·64 to 38137·78)	78·26 (55·49 to 109·4	53·91 (37·76 to 74·82	-31·11 (-47·67 to -11·42)
**Maternal abortion and miscarriage**	775·06 (474·72 to 1195·79)	349·35 (216·07 to 538·23)	1·35 (0·83 to 2·08)	0·7 (0·43 to 1·09)	-47·8 (-57·99 to -34·28)	94257·37 (67471·35 to 126410·93)	42430·15 (30895·04 to 57546·09)	164·05 (117·45 to 220·02	85·53 (62·25 to 117·05	-47·87 (-57·99 to -34·28)
**Ectopic pregnancy**	174·86 (106·33 to 279·17)	94·44 (63·06 to 128·45)	0·3 (0·19 to 0·49)	0·18 (0·12 to 0·25)	-39·34 (-53·84 to -17·18)	21243·05 (14862·19 to 29929·89)	11480·02 (9876·54 to 13359·5)	36·99 (25·92 to 52	22·45 (19·27 to 26·2	-39·31 (-53·84 to -17·18)
**Indirect maternal deaths**	NA	NA	NA	NA	NA	NA	NA	NA	NA	NA
**Late maternal deaths**	NA	NA	NA	NA	NA	NA	NA	NA	NA	NA

**Maternal deaths aggravated by HIV/AIDS**	NA	NA	NA	NA	NA	NA	NA	NA	NA	NA
**Other maternal disorders**	NA	NA	NA	NA	NA	NA	NA	NA	NA	NA
**Neonatal disorders**	290218·67 (251237·37 to 334515·44)	399078·29 (354827·57 to 455153·45)	669·09 (583·84 to 767·64)	871·52 (785·33 to 975·36)	30·25 (12·51 to 48·35)	52144·47 (48454·52 to 56021·72)	33735·38 (31385·6 to 36071·92)	160·82 (149·46 to 172·75	171·17 (159·27 to 182·99	6·44 (-2·39 to 15·89)
**Neonatal preterm birth**	262589·15 (225039·38 to 305596·06)	332350·96 (280779·94 to 391925·15)	607·82 (524·08 to 703·45)	721·87 (623·12 to 834·7)	18·76 (2·09 to 36·44)	42026·37 (38951·68 to 45070·67)	26849·76 (25035·33 to 28731·4)	129·67 (120·19 to 139·07	136·33 (127·12 to 145·89	5·14 (-5·5 to 16·87)
**Neonatal encephalopathy due to birth asphyxia and trauma**	55802·3 (36502·73 to 79529·18)	94015·85 (79377·59 to 110374·13)	123·1 (81·71 to 173·98)	188·81 (159·81 to 220·79)	53·38 (13·7 to 119·58)	3123·83 (2183·76 to 4202·26)	1707·66 (1177·3 to 2322·23)	9·64 (6·74 to 12·97	8·67 (5·98 to 11·79	-10·04 (-26·19 to 9·25)
**Neonatal sepsis and other neonatal infections**	19501·85 (14527·67 to 25450·67)	29896·06 (22466·62 to 38719·98)	44·66 (33·52 to 58·09)	61·73 (47·4 to 78·58)	38·22 (14·81 to 65·38)	6708·32 (4838·44 to 8866·52)	5010·32 (3770·56 to 6479·79)	20·62 (14·88 to 27·25	25·31 (19·06 to 32·72	22·73 (3·49 to 47·08)
**Hemolytic disease and other neonatal jaundice**	2305·55 (2031·8 to 2588·12)	2418·41 (2146·77 to 2712·57)	5·06 (4·47 to 5·69)	4·86 (4·3 to 5·47)	-4·05 (-8·43 to 0·89)	285·95 (35·9 to 931·64)	167·63 (22·31 to 545·41)	0·88 (0·11 to 2·87	0·85 (0·11 to 2·77	-3·53 (-4·29 to 0·39)
**Other neonatal disorders**	NA	NA	NA	NA	NA	NA	NA	NA	NA	NA
**Nutritional deficiencies**	8239887·35 (7741271·34 to 8730059·96)	4608943·9 (4000582·5 to 5246051·46)	18700·97 (17664·91 to 19753·63)	8516·73 (7402·72 to 9768·8)	-54·46 (-61·04 to -47·06)	329175·14 (277165·16 to 385856·83)	346962·9 (279575·11 to 431087·96)	813·73 (690·13 to 944·38	648·05 (530·53 to 794·66	-20·36 (-30·28 to -8·28)
**Protein-energy malnutrition**	296198·85 (249981·43 to 343482·42)	322172·66 (263457·39 to 389509·88)	748·8 (639·66 to 860·55)	581·91 (482·8 to 704·52)	-22·29 (-32·33 to -10·81)	320172·56 (267919·37 to 377589·68)	340471·37 (271636·73 to 423786·71)	794·62 (670·73 to 925·27	634·17 (517·6 to 782·15	-20·19 (-30·41 to -7·86)
**Iodine deficiency**	158474·3 (120829·64 to 206459·7)	145608·43 (112314·38 to 182893·66)	343·54 (263·73 to 441·62)	236·13 (178·77 to 300·05)	-31·27 (-36·27 to -25·86)	9002·57 (7002·21 to 11452·42)	6491·53 (5074·93 to 8234·38)	19·11 (14·96 to 24·02	13·88 (10·73 to 17·49	-27·38 (-31·98 to -22·69)
**Vitamin A deficiency**	905252·92 (732660·23 to 1099304·63)	281655·07 (241222·44 to 330920·97)	2229·59 (1767·28 to 2746·11)	620·71 (521·64 to 750·32)	-78·12 (-87·33 to -62·38)	905252·92 (732660·23 to 1099304·63)	281655·07 (241222·44 to 330920·97)	2229·59 (1767·28 to 2746·11	620·71 (521·64 to 750·32	0 (0 to 0)
**Dietary iron deficiency**	8257171·4 (7722914·81 to 8790496·24)	4264523·47 (3640223·29 to 4948106·17)	18690·63 (17585·83 to 19843·93)	7936·15 (6786·42 to 9260·89)	-57·54 (-64·42 to -49·78)	NA	NA	NA	NA	NA
**Other nutritional deficiencies**	NA	NA	NA	NA	NA	NA	NA	NA	NA	NA

**Non-communicable diseases**	39711875·44 (39196296·11 to 40179268·67)	49904478·67 (49567099·11 to 50226065·98)	89371·76 (88150·5 to 90444·28)	88892·63 (87707·78 to 89941·67)	-0·54 (-1·01 to -0·09)	70439073·6 (67018512·26 to 73810852·56)	88479101·83 (85159901·88 to 92172235·43)	161785·8 (154190·67 to 169574·75	163255·43 (156094·27 to 171271·92	0·91 (-0·96 to 2·59)
**Neoplasms**	8010783·41 (6830302·42 to 9335858·45)	12477890·41 (10785315·34 to 14321199·9)	18846·68 (16208·11 to 21783·86)	19216·92 (16631·5 to 22134·02)	1·96 (0·95 to 3·09)	4161598·02 (3495361·46 to 5002412·6)	6146479·1 (5164946·78 to 7392671·85)	9918·83 (8435·35 to 11777·91	9939·06 (8460·78 to 11788·85	0·2 (-0·25 to 0·72)
**Lip and oral cavity cancer**	1296·72 (1197·21 to 1415·23)	5072·07 (4121·42 to 6185·43)	3·65 (3·39 to 3·97)	5·96 (4·87 to 7·22)	63·35 (31·72 to 103·1)	424·25 (399·7 to 454·39)	1401·62 (1150·7 to 1682·75)	1·35 (1·27 to 1·45	1·62 (1·33 to 1·94	20·21 (-1·9 to 45·84)
**Nasopharynx cancer**	846·04 (674·17 to 1041·46)	7126·94 (5679·62 to 8861·46)	2·2 (1·79 to 2·69)	8·58 (6·88 to 10·53)	289·23 (186·49 to 434·33)	203·66 (168·17 to 241·38)	1086·86 (869·38 to 1345·81)	0·56 (0·47 to 0·67	1·3 (1·05 to 1·59	130·57 (72·78 to 205·82)
**Other pharynx cancer**	98·87 (90·77 to 107·55)	2400·76 (1842·66 to 3047·24)	0·29 (0·27 to 0·32)	2·68 (2·08 to 3·37)	809·75 (597·08 to 1063·8)	135·44 (124·47 to 147·31)	1089·15 (855·12 to 1354·92)	0·42 (0·39 to 0·46	1·2 (0·94 to 1·49	182·81 (118·54 to 259·56)
**Esophageal cancer**	3117·84 (2140·61 to 3378·3)	10744·94 (8346·17 to 14943·94)	9·44 (6·79 to 10·19)	11·76 (9·14 to 16·44)	24·62 (-5·54 to 145·39)	1783·96 (1282·73 to 1920·78)	3419·23 (2691·47 to 4689·31)	5·75 (4·38 to 6·16	3·76 (2·96 to 5·16	-34·6 (-49·45 to 20·69)
**Stomach cancer**	36485·25 (34529·79 to 38541·48)	81159·12 (66763·95 to 97309·53)	105·73 (99·93 to 111·92)	92·73 (76·47 to 110·7)	-12·29 (-28·31 to 6·14)	19679·29 (18764·85 to 20539·93)	25074·17 (20655·61 to 29936·02)	61·57 (58·72 to 64·45	28·67 (23·65 to 34·17	-53·44 (-61·36 to -44·04)
**Colon and rectum cancer**	24584·05 (23143·66 to 26150·74)	200482·24 (168785·88 to 237781·48)	78·15 (73·44 to 83·82)	224·22 (188·77 to 265·05)	186·91 (139·16 to 243·52)	5026·54 (4772·78 to 5316·16)	32929·03 (27484·11 to 39146·08)	16·97 (16·02 to 18·04	37·16 (31·14 to 44·01	119·04 (83·62 to 160·27)
**Liver cancer**	4163·32 (3432·74 to 5094·46)	41324·59 (33439·75 to 50085·28)	12·21 (10·1 to 14·94)	46·57 (37·77 to 56·34)	281·54 (182·06 to 408·35)	3547·65 (2935·49 to 4338·57)	20367·31 (16677·58 to 24503·6)	10·99 (9·18 to 13·42	22·8 (18·72 to 27·32	107·42 (57·03 to 173·79)
**Liver cancer due to hepatitis B**	2838·78 (2258·44 to 3535·34)	24076·36 (18583·81 to 30953·69)	7·89 (6·3 to 9·79)	27·31 (21·28 to 34·75)	246·2 (151·77 to 369·27)	2353·62 (1877·74 to 2934·99)	11449·42 (8884·66 to 14577·82)	6·83 (5·43 to 8·45	12·82 (9·97 to 16·31	87·84 (38·04 to 152·44)
**Liver cancer due to hepatitis C**	510·27 (327·53 to 733·58)	6651·43 (4372·57 to 9273·98)	1·79 (1·2 to 2·53)	7·36 (4·89 to 10·22)	310·32 (195·77 to 470·24)	476·14 (313·81 to 678·89)	3548·78 (2361·2 to 4873·81)	1·81 (1·23 to 2·55	3·98 (2·69 to 5·45	119·48 (61·2 to 197·07)
**Liver cancer due to alcohol use**	505·95 (317·03 to 762·86)	7122·97 (4637·27 to 10387·18)	1·56 (0·99 to 2·3)	7·89 (5·16 to 11·4)	405·66 (265·88 to 585·86)	447·21 (285·03 to 662·43)	3603·17 (2376·5 to 5240·04)	1·44 (0·93 to 2·12	3·98 (2·6 to 5·79	175·61 (104·17 to 270·87)
**Liver cancer due to NASH**	163·61 (107·36 to 237·3)	2141·97 (1395·75 to 3146·56)	0·54 (0·36 to 0·78)	2·39 (1·58 to 3·51)	341·26 (215·18 to 522·6)	148·2 (98·08 to 213·13)	1118·59 (730·77 to 1649·53)	0·53 (0·36 to 0·76	1·25 (0·83 to 1·83	136·64 (71·72 to 227·12)
**Liver cancer due to other causes**	144·69 (96·49 to 207·63)	1331·86 (865·57 to 1933·59)	0·42 (0·28 to 0·6)	1·62 (1·09 to 2·33)	284·63 (178·78 to 436·59)	122·49 (81·92 to 174)	647·35 (422·54 to 944·99)	0·38 (0·25 to 0·54	0·76 (0·52 to 1·09	101·52 (48·2 to 173·55)

**Gallbladder and biliary tract cancer**	3433·99 (2831·74 to 4107·95)	9537·42 (6988·65 to 11963·14)	11·74 (9·75 to 14·8)	10·74 (7·89 to 13·46)	-8·48 (-37·69 to 28·07)	2918·98 (2417·19 to 3556·99)	6968·68 (5150·4 to 8642·46)	10·6 (8·83 to 13·68)	7·93 (5·85 to 9·79)	-25·2 (-50·41 to 1·83)
**Pancreatic cancer**	1847·77 (1763·6 to 1945·22)	7871·71 (6543·91 to 9451·42)	6·02 (5·73 to 6·36)	8·81 (7·33 to 10·57)	46·4 (21·16 to 77·41)	2234·41 (2131·59 to 2356·98)	7982·24 (6671·18 to 9525·32)	7·65 (7·26 to 8·09)	8·98 (7·51 to 10·71)	17·46 (-1·43 to 39·99)
**Larynx cancer**	8564·98 (7718·83 to 9314·63)	16753·12 (13615·81 to 20341·92)	25·39 (23·01 to 27·49)	19·05 (15·61 to 22·97)	-24·97 (-39 to -3·99)	1357·39 (1221·12 to 1467·76)	2234·28 (1773·97 to 2794·6)	4·32 (3·92 to 4·65)	2·46 (1·94 to 3·06)	-43·17 (-55·84 to -25·18)
**Tracheal, bronchus, and lung cancer**	9380·52 (8910·86 to 9928·6)	68345·39 (55468·15 to 83426·36)	28·66 (27·24 to 30·46)	75·66 (61·36 to 92·07)	163·98 (113·49 to 226·31)	7551·17 (7221·43 to 7985·81)	31824·76 (26456·32 to 37538·08)	24·52 (23·44 to 26·13)	35·61 (29·58 to 41·94)	45·24 (21·09 to 74·29)
**Malignant skin melanoma**	788·01 (576·2 to 1514·7)	11558·14 (5132·09 to 15807·23)	1·88 (1·39 to 3·55)	14·7 (6·99 to 19·87)	682·53 (146·69 to 1235·72)	158·37 (120·32 to 301·59)	1441·83 (644·58 to 1968·12)	0·46 (0·35 to 0·85)	1·82 (0·87 to 2·45)	298·05 (27·27 to 554·12)
**Non-melanoma skin cancer**	69·77 (39·33 to 105·25)	364·35 (219·13 to 530·01)	0·61 (0·38 to 0·87)	0·49 (0·31 to 0·71)	-19·31 (-52·08 to 41·35)	1132·1 (1045·93 to 1224·81)	4942·9 (4681·1 to 5193·16)	4·55 (4·26 to 4·88)	5·77 (5·46 to 6·06)	26·9 (19·71 to 34·16)
**Non-melanoma skin cancer (squamous-cell carcinoma)**	281·23 (240·58 to 322·27)	950·18 (822·84 to 1091·95)	1·36 (1·17 to 1·58)	1·16 (1 to 1·33)	-15·28 (-26·18 to -1·84)	335·21 (300·75 to 371·75)	1078·96 (966·96 to 1196·8)	1·81 (1·64 to 2	1·33 (1·2 to 1·47	-26·96 (-34·52 to -18·85)
**Non-melanoma skin cancer (basal-cell carcinoma)**	133·63 (111·45 to 158·82)	614·4 (523·51 to 717·06)	0·46 (0·39 to 0·55)	0·72 (0·61 to 0·83)	55·34 (42·84 to 68·64)	796·89 (719·33 to 880)	3863·94 (3623·01 to 4086·88)	2·74 (2·5 to 2·99	4·45 (4·17 to 4·7	62·6 (51·77 to 73·73)
**Breast cancer**	44949·41 (39714·05 to 51207·71)	218096·94 (184775·63 to 253670·09)	135·32 (117·77 to 156·11)	252·29 (214·73 to 294·32)	86·45 (52·2 to 127·1)	3460·02 (3239·44 to 3716·34)	20328·25 (16423·11 to 24533·25)	9·5 (8·9 to 10·19	23·79 (19·29 to 28·65	150·27 (99·32 to 205·19)
**Cervical cancer**	19120·35 (14970·39 to 22125·13)	22827·51 (17517·8 to 30105·01)	44·9 (36·22 to 52·1)	30·15 (23·03 to 39·38)	-32·85 (-48·74 to -6·84)	3109·97 (2587·85 to 3641·45)	3596·92 (2787·09 to 4661·05)	7·93 (6·98 to 9·95	4·59 (3·53 to 5·99	-42·07 (-56·08 to -23·45)
**Uterine cancer**	8870·94 (3563·61 to 10544·35)	15843·42 (10165·34 to 20545·68)	22·74 (9·53 to 26·84)	18·27 (11·76 to 23·88)	-19·68 (-40·99 to 98·12)	1349·71 (577·06 to 1585·84)	2035·16 (1335·05 to 2625·08)	3·75 (1·69 to 4·38	2·34 (1·54 to 3·03	-37·6 (-53·63 to 42·89)
**Ovarian cancer**	2432·59 (2133·24 to 3824·92)	11155·11 (6565·08 to 14238·94)	6·02 (5·24 to 9·82)	13·99 (8·46 to 17·67)	132·48 (-22·54 to 218·48)	556·66 (484·31 to 923·47)	2363·84 (1376·09 to 2981·04)	1·56 (1·33 to 2·7	2·86 (1·71 to 3·57	83·48 (-42·34 to 154·11)
**Prostate cancer**	6358·75 (5460·02 to 8278·76)	86173·13 (65270·91 to 115080·29)	23·48 (20·18 to 30·78)	94·7 (71·71 to 125·84)	303·27 (161·02 to 411·07)	1015·46 (876·79 to 1376·86)	10608·99 (7917·82 to 14185·12)	4·02 (3·48 to 5·55	11·75 (8·74 to 15·62	192·35 (78·14 to 268·84)
**Testicular cancer**	619·42 (225·23 to 1263·93)	3569·17 (2083·69 to 5540·05)	1·37 (0·46 to 2·93)	8·37 (4·4 to 13·76)	510·2 (124·95 to 1851·93)	96·46 (57·24 to 161·99)	403·91 (239·89 to 617·5)	0·22 (0·13 to 0·38	0·93 (0·49 to 1·51	319·02 (88·07 to 722·56)

**Kidney cancer**	1901·22 (1737·53 to 2079·18)	17328·56 (14009·7 to 21145·85)	5·16 (4·74 to 5·58)	21·25 (17·27 to 25·75)	311·96 (228·58 to 413·95)	488·74 (455·16 to 523·41)	3443·21 (2801·88 to 4158·98)	1·5 (1·4 to 1·61	4·09 (3·37 to 4·9	172·28 (119·84 to 236·44)
**Bladder cancer**	6945·99 (6461·69 to 7444·87)	41383·78 (34389·49 to 49891·63)	22·35 (20·75 to 24·07)	46·17 (38·33 to 55·52)	106·59 (72·29 to 150·72)	1388·76 (1300·24 to 1491·7)	6441·44 (5305·96 to 7742·37)	5·13 (4·76 to 5·61	7·28 (6·01 to 8·74	42·02 (18·53 to 71·66)
**Brain and central nervous system cancer**	4874·2 (3531·09 to 6648·1)	30978·53 (19100·36 to 38927·33)	12·41 (9·07 to 16·62)	44·57 (28·04 to 55·64)	259·03 (78·76 to 419·88)	1513·02 (1080·29 to 2004·28)	4805·51 (2898·99 to 5975·96)	4·11 (2·98 to 5·35	6·7 (4·16 to 8·27	62·83 (-17·43 to 129·35)
**Thyroid cancer**	5407·44 (4266·07 to 11434·3)	44075·03 (29771·22 to 56704·35)	13·25 (10·51 to 28·13)	57·73 (37·52 to 74·76)	335·69 (34·2 to 553·03)	693·4 (555·87 to 1481·42)	5208·57 (3564·48 to 6623·39)	1·88 (1·52 to 4·05	6·77 (4·46 to 8·69	260·12 (10·09 to 433·55)
**Mesothelioma**	119·79 (91·65 to 171·43)	312·63 (237·08 to 406·65)	0·36 (0·28 to 0·51)	0·35 (0·27 to 0·46)	-3·2 (-43·7 to 39·29)	58·54 (46·83 to 80·37)	156·11 (122·42 to 196·73)	0·19 (0·15 to 0·26	0·18 (0·14 to 0·22	-4·57 (-40·8 to 32·46)
**Hodgkin lymphoma**	1084 (790·08 to 1786·07)	12756·98 (6968·47 to 17641·78)	2·94 (2·14 to 4·78)	16·44 (9·08 to 22·62)	459·75 (137·35 to 873·52)	151·86 (111·95 to 249·62)	1584·13 (860·96 to 2176·02)	0·44 (0·32 to 0·71	2·03 (1·12 to 2·78	366·09 (98·75 to 710·05)
**Non-Hodgkin lymphoma**	1920·3 (1055·37 to 2895·98)	30444·91 (23807·26 to 37714·1)	4·25 (2·41 to 6·26)	36·85 (29·01 to 45·14)	767·84 (449·35 to 1477·31)	1162·22 (1062·81 to 1266·73)	5456·33 (4494·43 to 6543·34)	3·09 (2·85 to 3·36	6·7 (5·54 to 8	116·71 (77·28 to 163·01)
**Multiple myeloma**	831·02 (602·04 to 1432·62)	5214·38 (2846·41 to 6977·88)	2·66 (1·91 to 4·69)	5·74 (3·17 to 7·67)	115·89 (-22·36 to 254·77)	373·52 (275·94 to 647·84)	1629·22 (911·63 to 2108·68)	1·28 (0·94 to 2·29	1·81 (1·02 to 2·34	41·06 (-49·92 to 126·43)
**Leukemia**	15899·48 (9787·01 to 19925·48)	35825·43 (24677·69 to 45417·59)	39·33 (24·61 to 50·46)	58·64 (42·27 to 74·75)	49·1 (0·21 to 159·34)	3100·01 (2345·13 to 3660·81)	5983·49 (4174·11 to 7467·31)	7·8 (6·02 to 9·45	9·19 (6·7 to 11·45	17·81 (-20·9 to 71·03)
**Acute lymphoid leukemia**	1106·17 (572·72 to 1553·46)	22162·69 (13471·46 to 29077·79)	2·46 (1·27 to 3·45)	37·34 (23·98 to 49·63)	1418·55 (727·12 to 3136·05)	356·12 (189·63 to 502·63)	2737·73 (1666·89 to 3573·81)	0·81 (0·42 to 1·14	4·58 (2·93 to 6·07	467·72 (197·99 to 1044·4)
**Chronic lymphoid leukemia**	153·25 (115·02 to 257·53)	1860·39 (1238·01 to 2420)	0·42 (0·31 to 0·71)	2·21 (1·5 to 2·85)	427·75 (137·52 to 717·89)	29·67 (22·54 to 50·26)	252·23 (166·65 to 326·65)	0·09 (0·07 to 0·16	0·3 (0·2 to 0·38	227·43 (41·83 to 398·32)
**Acute myeloid leukemia**	828·5 (490·35 to 1236·18)	1350·91 (852·97 to 1832·31)	1·85 (1·21 to 2·71)	2·12 (1·31 to 2·94)	14·16 (-33·38 to 79·24)	506·2 (364·57 to 658·63)	1114·25 (747·29 to 1433·75)	1·21 (0·96 to 1·57	1·5 (1 to 1·92	23·63 (-28·29 to 69·52)
**Chronic myeloid leukemia**	336·2 (258·17 to 562·94)	4825·26 (3233·34 to 6570·32)	0·76 (0·58 to 1·28)	6·45 (4·35 to 8·8)	748·77 (292·82 to 1189·58)	195·92 (151·73 to 328·6)	881·24 (590·34 to 1189·98)	0·47 (0·37 to 0·79	1·16 (0·78 to 1·56	144·91 (12·91 to 266·74)
**Other leukemia**	13475·36 (7004·27 to 17588·41)	5626·17 (4177·76 to 8050·44)	33·84 (18·28 to 45·03)	10·53 (7·66 to 15·03)	-68·88 (-80·29 to -26)	2012·1 (1115·38 to 2615·06)	998·03 (739·05 to 1379·32)	5·22 (3·1 to 6·85	1·66 (1·22 to 2·33	-68·17 (-79·49 to -32·11)

**Other malignant neoplasms**	10213·28 (8756·6 to 11699·29)	33552·14 (27211·64 to 40791·27)	24·21 (20·93 to 27·5)	52·66 (43·67 to 62·79)	117·48 (72·5 to 183·26)	2742·67 (2291·81 to 2992·44)	6335·17 (5176·44 to 7536·95)	7·39 (6·19 to 8	9·15 (7·56 to 10·85	23·9 (0·67 to 64·06)
**Other neoplasms**	7880112·11 (6702226·63 to 9211100·58)	11774805·37 (10061393·72 to 13628051·06)	18458·18 (15804·88 to 21430·95)	18380·92 (15758·55 to 21334·11)	-0·42 (-1·15 to 0·29)	4094183·81 (3428144·71 to 4934678·02)	5925336·81 (4934197·35 to 7171045·55)	9709·32 (8228·52 to 11569·7	9679·84 (8204·07 to 11552·63	-0·3 (-0·51 to -0·11)
**Myelodysplastic, myeloproliferative, and other haematopoietic neoplasms**	37298·54 (31219·38 to 44643·34)	101532·98 (81847·94 to 124497·37)	113·02 (93·32 to 136·25)	123·06 (101·14 to 148·86)	8·88 (2·49 to 16·21)	2655·85 (2194·13 to 3214·98)	7010·32 (5573·81 to 8656·25)	7·58 (6·26 to 9·12	8·34 (6·83 to 10·08	10·01 (2·79 to 19·02)
**Benign and in situ intestinal neoplasms**	1266426·86 (935519·9 to 1672565·89)	3153676·26 (2300768·72 to 4223043·94)	3514·92 (2601·37 to 4646·06)	3543·2 (2627·2 to 4686·17)	0·8 (0·22 to 1·41)	309286·92 (236296·63 to 405802·3)	687589·08 (518052·86 to 913264·68)	805·18 (611·38 to 1059·23	810·24 (615·66 to 1067·01	0·63 (0·29 to 1·03)
**Benign and in situ cervical and uterine neoplasms**	860445·78 (572150·23 to 1239889·1)	955866·17 (635286·32 to 1384423·48)	1687·33 (1116·38 to 2429·7)	1626·13 (1074·78 to 2340·15)	-3·63 (-3·97 to -3·31)	197476·63 (131127·38 to 279005·75)	165164·15 (111746·4 to 231737·17)	353·12 (235·88 to 495·99	336·57 (225·11 to 472·09	-4·69 (-4·92 to -4·25)
**Other benign and in situ neoplasms**	6127632·52 (4951334·52 to 7459681·91)	8394378·1 (6798110·23 to 10347714·27)	14168·67 (11603·83 to 17075·64)	14111·25 (11549·85 to 16995·07)	-0·41 (-0·6 to -0·17)	3584764·4 (2940655·89 to 4432033·06)	5065573·26 (4114327·86 to 6226885·58)	8543·44 (7087·11 to 10346·72	8524·69 (7066·61 to 10334·64	-0·22 (-0·44 to -0·01)
**Cardiovascular diseases**	1815391·4 (1724402·75 to 1901723·11)	3925065·02 (3728444·2 to 4126258·54)	5980·63 (5704·09 to 6258·19)	4517·72 (4299·91 to 4747·63)	-24·46 (-26·68 to -22·12)	222048·73 (210010·21 to 235280·16)	360762·84 (339774·49 to 383550·09)	751·31 (712 to 794·22	423·81 (400·55 to 449·74	-43·59 (-45·46 to -41·67)
**Rheumatic heart disease**	10273·53 (8574·76 to 12154·53)	20325·74 (16591·12 to 24532·87)	29·38 (24·77 to 34·37)	25·08 (20·75 to 29·76)	-14·65 (-23·88 to -5·31)	730·03 (633·25 to 838·63)	1257·04 (1050·23 to 1486·97)	2·13 (1·89 to 2·38	1·53 (1·3 to 1·79	-27·88 (-34·42 to -20·64)
**Ischemic heart disease**	307796·46 (280987·52 to 336330)	839303·98 (766300·72 to 920059·39)	1103·58 (1007·02 to 1200·38)	949·64 (871·03 to 1039·14)	-13·95 (-18·33 to -7·78)	44908·87 (40070·45 to 50378·3)	91191·83 (79408·89 to 103974)	176·7 (158·68 to 197·05	105·68 (92·77 to 119·9	-40·19 (-43·75 to -36·07)
**Stroke**	727038·87 (677346·82 to 779702·5)	1019291·64 (935025·03 to 1128950·99)	2374·42 (2209·67 to 2546·74)	1175·79 (1084·27 to 1298·92)	-50·48 (-53·28 to -47·02)	92316·64 (84615·04 to 101719·07)	92934·15 (83777·11 to 102520·33)	305·86 (280·56 to 338·03	109·23 (98·67 to 120·05	-64·29 (-66·47 to -62·06)
**Ischemic stroke**	519406·73 (474241·73 to 567950·03)	759142·28 (674522·38 to 872825·21)	1774·64 (1613·18 to 1947·51)	884·37 (790·28 to 1009·59)	-50·17 (-53·77 to -45·54)	53401·56 (46250·66 to 61839·04)	57137·67 (48608·28 to 65636·1)	182·93 (157·96 to 212·98	67·03 (57·08 to 76·56	-63·36 (-66·47 to -59·93)
**Intracerebral hemorrhage**	169254·77 (151765·35 to 188107·03)	153285·34 (136106·27 to 172451·16)	506·58 (451·58 to 564·63)	183·98 (163·27 to 206·19)	-63·68 (-66·25 to -60·92)	24597·56 (22462·07 to 27071·46)	15112·58 (13645·16 to 17001·39)	80·56 (74·01 to 87·97	18·38 (16·59 to 20·63	-77·19 (-78·94 to -75·45)
**Subarachnoid hemorrhage**	85854·39 (70808·19 to 100336·01)	145931·61 (125653·29 to 168945·22)	251·76 (205·66 to 302·98)	167·46 (144·89 to 192·46)	-33·49 (-42·84 to -25·05)	14317·53 (12249·34 to 16733·42)	20683·9 (17088·02 to 24476·93)	42·37 (36·06 to 48·9	23·83 (19·89 to 27·97	-43·77 (-49·68 to -37·76)
**Hypertensive heart disease**	27757·91 (19665·53 to 38138·45)	93991·02 (65770·02 to 128763·35)	120·67 (85·42 to 167·15)	111·62 (78·04 to 153·02)	-7·5 (-16·48 to 2·99)	NA	NA	NA	NA	NA

**Non-rheumatic valvular heart disease**	195849·65 (161798·71 to 234883·99)	735283·16 (635221·02 to 853488·95)	572·03 (476·37 to 683·47)	856·56 (743·32 to 994·59)	49·74 (36·64 to 65·42)	11542·38 (9762·07 to 13660·06)	33023·12 (28608·01 to 38367·43)	28·14 (23·85 to 33·19	41·83 (36·67 to 48·22	48·61 (36·15 to 63·53)
**Non-rheumatic calcific aortic valve heart disease**	28187·68 (22924·39 to 34052·97)	268834·29 (226639·75 to 317267·62)	92·66 (75·6 to 111·97)	310·27 (261·14 to 364·94)	234·86 (186·12 to 295·02)	2202·91 (1820·13 to 2650·65)	14718·91 (12134·8 to 17671·54)	6·74 (5·58 to 8·2	17·58 (14·74 to 20·76	160·77 (117·84 to 215·58)
**Non-rheumatic degenerative mitral valve heart disease**	171767·51 (139475·89 to 210310·99)	479023·88 (392193·24 to 595097·97)	490·11 (398·25 to 602·76)	561·27 (459·64 to 695·76)	14·52 (5·53 to 25·11)	9339·47 (7593·95 to 11539·18)	18304·21 (14653·78 to 23125·07)	21·41 (17·32 to 26·67	24·25 (19·8 to 30·02	13·29 (4·69 to 23·35)
**Other non-rheumatic valve heart diseases**	71·65 (53·41 to 91·33)	131·36 (103·33 to 167·59)	0·2 (0·15 to 0·25)	0·17 (0·13 to 0·21)	-13·79 (-22·1 to -4·46)	NA	NA	NA	NA	NA
**Cardiomyopathy and myocarditis**	6650·99 (4590·13 to 9461·82)	21074·53 (14670·54 to 29674·94)	24·56 (17·18 to 33·94)	23·59 (16·73 to 32·78)	-3·94 (-20·82 to 17·81)	7371·09 (5788·23 to 9055·68)	12401·47 (9907·86 to 15236·61)	18·75 (15·18 to 22·67	19·08 (15·45 to 23·08	1·76 (1·43 to 2·15)
**Myocarditis**	3960·82 (3219·26 to 4824)	5671·22 (4736·07 to 6564·41)	9·62 (7·99 to 11·53)	9·41 (7·82 to 11·14)	-2·13 (-7·22 to 3·03)	NA	NA	NA	NA	NA
**Alcoholic cardiomyopathy**	520·91 (380·98 to 692·91)	978·04 (724·13 to 1322·83)	1·29 (0·95 to 1·69)	1·26 (0·95 to 1·65)	-2·07 (-12·21 to 10·91)	NA	NA	NA	NA	NA
**Other cardiomyopathy**	11694·26 (8752·34 to 15201·59)	25519·77 (19114·83 to 33901·14)	34·14 (25·76 to 44·64)	32·87 (25·31 to 42·36)	-3·72 (-13·54 to 9·31)	NA	NA	NA	NA	NA
**Atrial fibrillation and flutter**	108190·83 (82457·32 to 137452·53)	319063·75 (244234·16 to 404256·85)	383·49 (296·62 to 483·7)	354·55 (271·6 to 448·59)	-7·54 (-11·87 to -3·84)	7371·09 (5788·23 to 9055·68)	12401·47 (9907·86 to 15236·61)	18·75 (15·18 to 22·67	19·08 (15·45 to 23·08	1·76 (1·43 to 2·15)
**Aortic aneurysm**	NA	NA	NA	NA	NA	8934·03 (6887·71 to 11345·82)	21840·17 (16460·05 to 28433·24)	27·61 (21·38 to 35·44	24·56 (18·62 to 31·52	-11·06 (-14·72 to -7·55)
**Peripheral artery disease**	521975·11 (449839 to 594595·6)	1082553·57 (938137·32 to 1236333·95)	1953·96 (1698·42 to 2217·26)	1212·81 (1052·69 to 1381·32)	-37·93 (-40·88 to -35·05)	51691·37 (44587·47 to 59197·86)	99178·11 (85614·31 to 114035·25)	178·1 (154·77 to 203·34	110·19 (95·23 to 126·21	-38·13 (-41·09 to -35·11)
**Endocarditis**	2079·32 (1648·39 to 2564·75)	5607·08 (4427·47 to 7193·41)	8·2 (6·41 to 10·38)	7·05 (5·64 to 8·88)	-14·04 (-22·26 to -5·25)	4554·31 (3852·3 to 5326·21)	8936·97 (7303·32 to 10947·23)	14·02 (12·03 to 16·1	11·71 (9·65 to 14·13	-16·46 (-24·24 to -8·41)
**Other cardiovascular and circulatory diseases**	114011·22 (82026·53 to 156331·21)	197299·93 (148443·5 to 262079·04)	270·93 (195·51 to 371·55)	275·52 (209·89 to 363·59)	1·69 (-7·8 to 14·84)	0 (0 to 0)	0 (0 to 0)	0 (0 to 0	0 (0 to 0	0 (0 to 0)
**Chronic respiratory diseases**	2125934·17 (1881638·36 to 2458567·78)	3426022·08 (3100205·71 to 3805665·89)	5921·03 (5325·63 to 6617·75)	5408·67 (4763·02 to 6283·3)	-8·65 (-16·82 to -0·24)	291449·51 (252614·33 to 341680·63)	441955·26 (400498·47 to 488856·39)	808·69 (703·98 to 940·92	787·47 (674·82 to 931	-2·62 (-12·51 to 6·76)

**Chronic obstructive pulmonary disease**	538427·53 (498278·4 to 577132·59)	1669944·36 (1582267·87 to 1761411·69)	1828·38 (1720·91 to 1945·98)	2027·25 (1921·51 to 2140·83)	10·88 (6·25 to 15·4)	42097·93 (39787·9 to 44403·34)	143629·62 (137817·19 to 149402·02)	154·91 (146·56 to 163·03	171·98 (165·5 to 178·22	11·02 (6·2 to 16·03)
**Pneumoconiosis**	2316·85 (1952·8 to 2800·34)	6700·96 (5537·46 to 8272·99)	8·33 (7·03 to 9·98)	7·51 (6·23 to 9·26)	-9·81 (-23·15 to 6·92)	282·5 (241·25 to 333·48)	738·56 (620·09 to 883·71)	0·9 (0·77 to 1·05	0·83 (0·69 to 0·98	-7·83 (-18·14 to 5·93)
**Silicosis**	478·16 (351·41 to 696·63)	1805·3 (1295·65 to 2488·8)	1·88 (1·39 to 2·6)	2·04 (1·47 to 2·8)	8·18 (-23·69 to 29·38)	62·89 (46·45 to 84·33)	201·4 (146·93 to 268·99)	0·22 (0·16 to 0·28	0·22 (0·16 to 0·3	2·21 (-15·44 to 19·25)
**Asbestosis**	28·23 (15·25 to 44·62)	82·27 (52·75 to 117·25)	0·08 (0·05 to 0·12)	0·1 (0·06 to 0·14)	21·18 (1·09 to 51·39)	22·26 (11·37 to 35·79)	61·5 (36·73 to 87·07)	0·07 (0·04 to 0·09	0·08 (0·05 to 0·11	15·72 (-5·06 to 44·36)
**Coal workers pneumoconiosis**	183·43 (137·28 to 236·06)	1394·19 (1056·07 to 1781·91)	0·59 (0·44 to 0·76)	1·54 (1·17 to 1·98)	160·61 (104·67 to 232·16)	32·88 (24·63 to 41·38)	154·42 (117·42 to 198·91)	0·11 (0·08 to 0·14	0·17 (0·13 to 0·22	57·52 (26·76 to 98·56)
**Other pneumoconiosis**	1627·03 (1277·64 to 2051·83)	3419·21 (2506·77 to 4795·05)	5·77 (4·55 to 7·24)	3·83 (2·81 to 5·36)	-33·64 (-48·62 to -10·95)	164·48 (129·64 to 206·05)	321·24 (232·14 to 447·67)	0·5 (0·4 to 0·63	0·36 (0·26 to 0·5	-29·29 (-42·98 to -8·07)
**Asthma**	1673791·92 (1416284·09 to 2002797·53)	1915846·03 (1611521·12 to 2324501·29)	4491·3 (3824·36 to 5330·33)	3608·56 (2959·47 to 4514·64)	-19·65 (-32·15 to -7·23)	225485·04 (186171·2 to 274991·59)	211096·03 (177799·79 to 250592·98)	586·87 (486·52 to 715·45	510·52 (400·45 to 652·89	-13·01 (-24·79 to 0·17)
**Interstitial lung disease and pulmonary sarcoidosis**	18294·99 (15212·74 to 21369·44)	89113·23 (76325·28 to 101212·45)	52·88 (44·99 to 60·7)	102·19 (87·52 to 115·41)	93·23 (78·17 to 110·44)	23584·04 (19073·93 to 28646·49)	86491·05 (66966·79 to 107462·83)	66·02 (52·79 to 80·36	104·15 (82·19 to 128·24	57·76 (43·33 to 73·3)
**Other chronic respiratory diseases**	NA	NA	NA	NA	NA	NA	NA	NA	NA	NA
**Digestive diseases**	9350509·02 (8826443·04 to 9865265·3)	13764569·12 (12965375·23 to 14552940·78)	22074·09 (20958·86 to 23177·74)	18966·38 (17845·6 to 20042·23)	-14·08 (-16·32 to -11·69)	2039125·94 (1859971·13 to 2241647·17)	3388732·18 (3112457·99 to 3744604·46)	4875·5 (4495·63 to 5318·58	4920·56 (4542·44 to 5381·88	0·92 (-0·96 to 3·04)
**Cirrhosis and other chronic liver diseases**	5754861·56 (5342009·62 to 6212263·19)	8049983·89 (7429077·46 to 8777701·26)	13269·87 (12378·38 to 14218·89)	10883·36 (9990·71 to 11886·07)	-17·98 (-21·2 to -14·67)	30126·55 (26539·9 to 33492·23)	16886·53 (14580·13 to 19355·5)	67·54 (58·79 to 75·39	25·54 (22·46 to 28·91	-62·18 (-65·53 to -58·1)
**Cirrhosis and other chronic liver diseases due to hepatitis B**	2332154·03 (2153262·3 to 2521741·11)	1574686·95 (1499108·46 to 1657839·82)	5034·91 (4659 to 5408·89)	2055·3 (1952·11 to 2175·08)	-59·18 (-61·53 to -56·6)	15813·63 (12974·07 to 18596·65)	6094·28 (4599·46 to 7805·25)	34·96 (28·61 to 41·36	8·78 (6·72 to 11·2	-74·87 (-78·63 to -70·49)
**Cirrhosis and other chronic liver diseases due to hepatitis C**	259671·21 (217473·85 to 306393·83)	319416·16 (258928·98 to 392963·93)	684·87 (577·74 to 811·71)	432·89 (351·99 to 527·52)	-36·79 (-42·63 to -29·28)	2514·72 (1611·05 to 3691·39)	1690·14 (1071·8 to 2506·83)	5·57 (3·54 to 8·24	2·46 (1·6 to 3·61	-55·82 (-62·29 to -48·3)
**Cirrhosis and other chronic liver diseases due to alcohol use**	213282·67 (167168·83 to 262055·22)	269969·18 (221129·94 to 321908·83)	567·55 (446·29 to 700·34)	312·06 (257·62 to 371·04)	-45·02 (-51·19 to -37·64)	9593·64 (7089·43 to 12442·67)	7552·52 (5650·75 to 9394·8)	21·75 (15·93 to 28·32	10·44 (7·97 to 13·03	-52·03 (-59·22 to -43·8)

**Cirrhosis and other chronic liver diseases due to NAFLD**	2915593·23 (2559512·63 to 3318461·35)	5850628·82 (5220242·48 to 6576835·44)	6897·41 (6117·72 to 7777·7)	8014·34 (7122·55 to 8999·63)	16·19 (11·9 to 20·56)	445·61 (300·9 to 641·56)	392·06 (239·44 to 636·23)	1 (0·66 to 1·43	0·55 (0·34 to 0·91	-44·38 (-55·52 to -29·93)
**Cirrhosis and other chronic liver diseases due to other causes**	34160·42 (27033·03 to 43141·37)	35282·78 (28148·9 to 44984·49)	85·13 (67·71 to 107·63)	68·77 (54·4 to 86·58)	-19·22 (-29·58 to -7·81)	1758·94 (1348·82 to 2228·79)	1157·53 (897·4 to 1460·1)	4·26 (3·2 to 5·45	3·31 (2·41 to 4·36	-22·32 (-32·92 to -10·9)
**Upper digestive system diseases**	2875447·14 (2498253·93 to 3259774·52)	4915938·35 (4358467·82 to 5611462·87)	6843·3 (6030·23 to 7748·58)	6735·98 (5927·31 to 7612·09)	-1·57 (-2·88 to -0·47)	1279863·65 (1111322·11 to 1469364·78)	2108397·41 (1855526·56 to 2433087·28)	3014·06 (2661·57 to 3452·59	2951·81 (2600·08 to 3397·46	-2·07 (-2·57 to -1·58)
**Peptic ulcer disease**	49126·4 (41375·48 to 58182·72)	72545·78 (56823·23 to 89914·43)	134·27 (114·99 to 155·29)	101·15 (80·62 to 124·6)	-24·66 (-33·68 to -14·01)	19524·71 (16589·79 to 22977·76)	28352·03 (23010·84 to 34534·21)	55·3 (47·76 to 64·02	39·53 (31·86 to 47·54	-28·51 (-36·1 to -18·73)
**Gastritis and duodenitis**	89246·57 (72162·63 to 109645·81)	94718·6 (75474·97 to 118073·42)	229·62 (188·8 to 277·71)	155·97 (124·57 to 193·41)	-32·08 (-38·98 to -24·95)	62395·02 (50723·15 to 74854·03)	70113·16 (56033·05 to 85664·69)	156·9 (129·96 to 184·14	115·04 (93·35 to 139·8	-26·68 (-32·34 to -21·05)
**Gastroesophageal reflux disease**	2863966·81 (2457965·7 to 3276779·61)	4982275·68 (4378193·66 to 5742303·05)	6802·59 (5958·48 to 7784·93)	6784·53 (5942·11 to 7757·37)	-0·27 (-0·48 to -0·06)	1197943·91 (1030385 to 1384711·05)	2009932·22 (1761911·19 to 2339294·52)	2801·86 (2455·35 to 3241·54	2797·25 (2451·56 to 3239·66	-0·16 (-0·33 to -0·01)
**Appendicitis**	7872·79 (5797·97 to 10501·52)	7103·16 (5457·24 to 9048·75)	15·97 (11·87 to 20·99)	16·99 (12·59 to 22·39)	6·37 (0·04 to 12·33)	204644·13 (151347·92 to 270222·86)	183446·56 (140793·7 to 233087·67)	417·24 (311·09 to 545·92	443·27 (327·57 to 582·64	6·24 (-0·14 to 12·14)
**Paralytic ileus and intestinal obstruction**	1606·71 (1537·46 to 1674·92)	5111·21 (4885·21 to 5332·78)	4·47 (4·28 to 4·66)	9·4 (8·98 to 9·85)	110·26 (102·47 to 119·37)	32416·47 (31095·69 to 33751·48)	120474·94 (115403·13 to 125748·94)	88·49 (85 to 92·13	175·41 (168·64 to 182·02	98·23 (92·11 to 104·89)
**Inguinal, femoral, and abdominal hernia**	242062·13 (199064·13 to 290766·52)	265592·09 (215847·6 to 319544·01)	605·92 (501·88 to 718·94)	492·65 (412·82 to 582·56)	-18·69 (-24·02 to -12·59)	72082·52 (59914·67 to 85531·55)	113934·54 (92119·96 to 137806·95)	186·9 (157 to 218·67	207·23 (172·89 to 244·67	10·88 (4·16 to 18·9)
**Inflammatory bowel disease**	17167·24 (15173·62 to 19498·03)	46956·28 (44197·19 to 49996·79)	37·89 (33·49 to 42·88)	70·21 (66·03 to 74·79)	85·29 (67·2 to 107·71)	1586·68 (1390·2 to 1776·81)	4407·24 (4219·72 to 4589·27)	3·37 (2·96 to 3·78	7·33 (7·03 to 7·63	117·87 (97·12 to 143·69)
**Vascular intestinal disorders**	1117·15 (875·76 to 1466·02)	3818·02 (3175·23 to 4603·93)	2·79 (2·23 to 3·5)	4·89 (4·11 to 5·88)	75·03 (61·12 to 87·12)	10300·92 (8074·52 to 13083·23)	36147·48 (29169·18 to 43031·37)	26·4 (21·01 to 32·79	45·53 (37·09 to 54·37	72·5 (61·25 to 83·63)
**Gallbladder and biliary diseases**	1794764·85 (1521057·6 to 2109919·89)	2577994·1 (2170816·49 to 3120630·41)	4823·33 (4119·7 to 5573·91)	3340·27 (2810·47 to 3979·41)	-30·75 (-36·84 to -24)	397572·07 (338528·9 to 463389·19)	788133·21 (667010·22 to 947723·98)	1045·05 (900·33 to 1200·77	1040·4 (878·58 to 1230·76	-0·45 (-9·01 to 8·23)
**Pancreatitis**	12601·37 (11244·17 to 14080·86)	30415·72 (26761·73 to 34042·29)	32·11 (29·02 to 35·51)	40·02 (35·31 to 44·86)	24·62 (18·91 to 30·12)	10532·95 (8720·11 to 12721·37)	16904·26 (14038·59 to 19885·07)	26·46 (22·27 to 31·18	24·02 (20·14 to 28·61	-9·22 (-11·65 to -6·55)
**Other digestive diseases**	NA	NA	NA	NA	NA	NA	NA	NA	NA	NA

**Neurological disorders**	16342950·49 (14802403·43 to 17909064·21)	21843857·68 (20118060·59 to 23539892·79)	35257·32 (32160·94 to 38437·54)	36355·33 (33690·47 to 39174·81)	3·11 (-1·17 to 7·87)	5280224·64 (4657441·07 to 5923105·46)	6380274·2 (5664235·33 to 7132803·12)	11353·02 (10119·69 to 12603·28	11409·58 (10099·8 to 12729·98	0·5 (-3·97 to 5·11)
**Alzheimer's disease and other dementias**	134156·73 (115505·68 to 152743·18)	597508·07 (520822·45 to 685442·14)	732·14 (633·8 to 839·74)	703·94 (614·61 to 806)	-3·85 (-9·1 to 1·7)	20492·93 (17608·75 to 23620·15)	83488·73 (72592·8 to 95838·78)	105·91 (91·87 to 120·14	97·87 (85·49 to 111·12	-7·59 (-12·15 to -3·14)
**Parkinson's disease**	15472·52 (12781·96 to 18601·21)	72582·88 (61424·3 to 88221·72)	59·42 (49·57 to 71·61)	81·15 (68·62 to 98·4)	36·58 (14·66 to 59·61)	2028·93 (1779·03 to 2325·95)	9064·78 (8129·48 to 10372·13)	7·8 (6·95 to 8·77	10·14 (9·13 to 11·51	30·01 (13·53 to 47·18)
**Idiopathic epilepsy**	122401·21 (29713·64 to 203707·25)	142681·93 (45383·59 to 219225·44)	279·01 (68·55 to 460·29)	270·27 (89·76 to 423·44)	-3·13 (-70·98 to 263·94)	17641·25 (4264·41 to 29647·39)	18358·81 (6021·31 to 28817·96)	40·87 (9·73 to 68·75	41·38 (13·49 to 66·13	1·24 (-68·28 to 282·47)
**Multiple sclerosis**	3506·07 (2703·94 to 4385·67)	6624·02 (5186·16 to 8107·25)	8·4 (6·55 to 10·38)	8·71 (6·8 to 10·72)	3·74 (-0·2 to 8·1)	183·63 (144·44 to 225·16)	221·87 (180·83 to 270·21)	0·38 (0·3 to 0·46	0·37 (0·3 to 0·45	-2·3 (-5·54 to 1·31)
**Motor neuron disease**	1396·3 (1149·41 to 1676·91)	2794·75 (2272·37 to 3390)	3·34 (2·78 to 3·96)	4·2 (3·45 to 4·99)	25·9 (19·81 to 32·84)	211·38 (177·38 to 252·04)	555·53 (448·55 to 713·14)	0·62 (0·52 to 0·74	0·71 (0·58 to 0·88	14·21 (7·59 to 20·99)
**Headache disorders**	16167469·73 (14616564·38 to 17725908·41)	21323334·51 (19623125·97 to 23030646·02)	34557·07 (31374·11 to 37726·14)	35670·2 (32990·19 to 38477·15)	3·22 (-1·04 to 8·11)	5239666·52 (4615431·55 to 5885550·6)	6268584·49 (5558238·92 to 7019464·94)	11197·44 (9953·67 to 12469·84	11259·12 (9970·29 to 12572·02	0·55 (-3·99 to 5·22)
**Migraine**	5970873·03 (5054446·27 to 7100004·16)	6609292·17 (5743040·59 to 7642390·06)	12388·67 (10547·54 to 14596·69)	11300·03 (9782·2 to 13138·01)	-8·79 (-15·07 to -2·73)	475130·79 (398939·82 to 554785·39)	408450·5 (352195·91 to 469025·49)	966·05 (814·35 to 1119·82	912·3 (780·55 to 1049·65	-5·56 (-10·63 to -1·16)
**Tension-type headache**	13666570·39 (11729558·81 to 15782613·96)	19195780·84 (16998370·58 to 21355186·61)	29345·37 (25404·91 to 33500·5)	31833·68 (28384·47 to 35299·65)	8·48 (1·44 to 17·39)	4764535·73 (4148095·83 to 5381921·64)	5860133·99 (5154449·16 to 6629013·64)	10231·38 (8999·79 to 11498·06	10346·82 (9073·26 to 11647·15	1·13 (-3·72 to 6·24)
**Other neurological disorders**	1072·6 (730·77 to 1496·33)	1285·66 (859·26 to 1777·41)	2·38 (1·61 to 3·25)	2·39 (1·62 to 3·26)	0·43 (0·22 to 0·67)	0 (0 to 0)	0 (0 to 0)	0 (0 to 0	0 (0 to 0	0 (0 to 0)
**Mental disorders**	4742744·78 (4325357·4 to 5245690·73)	5707968·25 (5238261·92 to 6264145·25)	10251·38 (9366·63 to 11276·5)	10033·55 (9165·43 to 11041·24)	-2·12 (-5·62 to 1·57)	1423067·75 (1281095·72 to 1575632·54)	1869646·34 (1694262·8 to 2052999·69)	3090·34 (2806·33 to 3401·56	3323·36 (3011·5 to 3664·66	7·54 (2·48 to 12·63)
**Schizophrenia**	133929·58 (106955·44 to 166172·77)	209232·12 (169513·43 to 251834·41)	292·69 (235·76 to 357·18)	300·63 (241 to 366·39)	2·71 (-2·51 to 8·85)	9279·93 (7348·53 to 11786·53)	9209·09 (7433·94 to 11255·73)	17·46 (14·02 to 21·74	17·73 (14·25 to 21·98	1·51 (-3·76 to 7·91)
**Depressive disorders**	895157·19 (777670·63 to 1027913·05)	1347881·33 (1203177·7 to 1516315·59)	1965·03 (1730·89 to 2240·18)	2091·18 (1858·52 to 2363·13)	6·42 (1·2 to 11·86)	929542·33 (809561·08 to 1062927·58)	1419062·5 (1262215·41 to 1581401·88)	2043·51 (1795·18 to 2323·17	2241·21 (1991·18 to 2513·99	9·67 (2·53 to 17·09)
**Major depressive disorder**	584845·38 (506451·22 to 674936·91)	908890·02 (807726·98 to 1015320·06)	1287·63 (1129·91 to 1474·15)	1420·9 (1258·66 to 1594·52)	10·35 (2·84 to 18·28)	873666·25 (755148·02 to 1006240·07)	1346936·8 (1191286·16 to 1510550·83)	1923·9 (1674·88 to 2202·08	2122·29 (1877·56 to 2388·11	10·31 (2·75 to 18·26)

Dysthymia	320514·62 (240815·43 to 429810·56)	455015·3 (355770·63 to 584956·1)	700·24 (540·57 to 908·92)	695·76 (536·9 to 902·2)	-0·64 (-0·92 to -0·41)	55876·08 (42455·35 to 72693·94)	72125·7 (57925·85 to 89075)	119·6 (94·56 to 149·7	118·92 (93·91 to 148·82	-0·57 (-0·76 to -0·4)
Bipolar disorder	227870·77 (174806·9 to 286387·69)	320363·55 (253559·55 to 397220·43)	489·41 (381·87 to 603·5)	500·3 (391·5 to 620·24)	2·23 (-3·17 to 7·43)	19796·17 (15575·24 to 24635·19)	22997·41 (18383·53 to 28411·25)	42·01 (33·31 to 51·81	42·85 (33·86 to 52·9	2·01 (-3·34 to 7·4)
Anxiety disorders	1500797·52 (1165565·06 to 1888956·98)	1862218·82 (1479473·85 to 2342306·73)	3238·05 (2563·84 to 4037·18)	3221·32 (2563·35 to 4047·1)	-0·52 (-6·46 to 6·41)	238296·62 (183355·51 to 302562·67)	258590·08 (202542·88 to 325499·92)	508·73 (396·89 to 629·29	507·93 (396·08 to 633·85	-0·16 (-5·95 to 5·41)
Eating disorders	127809·02 (95470·63 to 164622·85)	166858·55 (121179·13 to 217616·6)	237·24 (177·53 to 305·74)	351·35 (259·95 to 452·81)	48·1 (33·72 to 64·04)	102321·63 (69259·06 to 143388·42)	91117·44 (61598·2 to 124936·39)	188·81 (128·05 to 266·72	241·15 (164·47 to 336·49	27·72 (18·08 to 37·88)
Anorexia nervosa	41633·73 (29063·58 to 59919·84)	49243·6 (35199·49 to 68913·11)	77·31 (54·65 to 110·05)	109·23 (77·4 to 155·47)	41·28 (23·16 to 60·06)	10799·44 (7451·76 to 15372·25)	9160·47 (6402·63 to 13106·95)	20·43 (14·17 to 28·99	25·29 (17·24 to 36·73	23·76 (7·69 to 40·48)
Bulimia nervosa	86693·34 (57747·26 to 118726·2)	118020·1 (77782·88 to 160956·48)	160·99 (108·57 to 221·17)	243·48 (162·97 to 331·74)	51·24 (38·05 to 66·01)	91522·19 (58430·46 to 132713·36)	81956·97 (53103·31 to 116416·46)	168·38 (108·12 to 244·14	215·87 (140·83 to 309·09	28·2 (17·59 to 39·81)
Autism spectrum disorders	244421·2 (201799·66 to 291106·24)	271134·58 (224314·05 to 324211·88)	540·39 (445·69 to 643·75)	548·87 (452·67 to 653·65)	1·57 (1·44 to 1·7)	4760·53 (3935·44 to 5664·17)	2733·87 (2260·33 to 3250·83)	14·69 (12·14 to 17·48	13·88 (11·48 to 16·51	-5·49 (-5·61 to -5·38)
Attention-deficit/hyperactivity disorder	704585·95 (507521·48 to 990238·65)	522226·28 (386012·57 to 690802·33)	1447·22 (1035·03 to 2020·86)	1372·32 (1012·68 to 1832·47)	-5·18 (-19·4 to 9·32)	33887·95 (22149·07 to 50598·05)	18830·03 (12763·97 to 26823·01)	86·13 (56·26 to 128·72	78·46 (53·17 to 111·8	-8·9 (-25·3 to 7·69)
Conduct disorder	252063·18 (181357·37 to 336016·77)	138885·29 (96704·24 to 185254·74)	527·81 (381·04 to 699·37)	502·41 (349·15 to 665·47)	-4·81 (-12·5 to 1·46)	85182·6 (60864·36 to 112725·87)	47105·92 (33154·59 to 62565·06)	189 (134·24 to 249·38	180·14 (126·56 to 238·25	-4·69 (-9·75 to 0·88)
Idiopathic developmental intellectual disability	229535·73 (78782·06 to 377884·09)	47528·41 (6035·45 to 105658·21)	500·25 (170·67 to 825·93)	91·52 (10·08 to 209·46)	-81·71 (-93·98 to -73·77)	NA	NA	NA	NA	NA
Other mental disorders	700206·92 (535107·22 to 905322·81)	1135326·33 (888760·45 to 1447505·66)	1590·94 (1227·4 to 2049·15)	1609·11 (1241·81 to 2072·63)	1·14 (0·86 to 1·44)	0 (0 to 0)	0 (0 to 0)	0 (0 to 0	0 (0 to 0	0 (0 to 0)
Substance use disorders	1792232·78 (1518760·32 to 2112930·87)	1937953·36 (1666207·58 to 2231576·91)	3737·41 (3200·47 to 4325·12)	3066·02 (2616·55 to 3568·45)	-17·96 (-27·7 to -4·34)	693466·63 (550363·64 to 821044·27)	729248·68 (597948·92 to 872481·71)	1405·85 (1126·97 to 1644·04	1200·99 (979·04 to 1445·93	-14·57 (-26·87 to 1·51)
Alcohol use disorders	1437526·65 (1176539·24 to 1721325·06)	1575695·42 (1303479·14 to 1874366·62)	3082·9 (2563·46 to 3639·97)	2331·83 (1907·64 to 2814·68)	-24·36 (-35·93 to -7·48)	576844·95 (441106·66 to 700061·03)	602625·58 (473003·74 to 738769·72)	1178·37 (913·61 to 1409·21	959·54 (741·33 to 1198·09	-18·57 (-32·72 to 0·02)
Drug use disorders	407232·36 (312716·39 to 530076·04)	403881·8 (330578·06 to 496479·44)	762·75 (592·92 to 986·56)	801·72 (628·65 to 1022·15)	5·11 (-0·26 to 11)	116621·69 (93799·66 to 143168·64)	126623·1 (100802·72 to 153991·92)	227·48 (182·92 to 279·2	241·46 (196·29 to 295·1	6·14 (1·03 to 11·55)

**Opioid use disorders**	21167·73 (15737·31 to 28478·45)	44301·56 (33608·44 to 58014·49)	42·77 (32·31 to 56·09)	76·36 (56·73 to 103)	78·56 (62·09 to 94·17)	3943·49 (2928·48 to 5291·94)	6918·27 (5288·25 to 8903·41)	7·78 (5·88 to 10·17	13·26 (9·86 to 17·87	70·51 (56·46 to 84·41)
**Cocaine use disorders**	71838·11 (51917·72 to 97146·77)	77320·69 (57418·75 to 103326·71)	140·74 (103·05 to 187·56)	140·6 (103·13 to 193·76)	-0·1 (-11·25 to 13·2)	6029·71 (4199·53 to 8746·89)	4160·05 (3027·23 to 5841·8)	11·36 (7·94 to 16·55	11·34 (7·96 to 16·55	-0·16 (-15·11 to 17·06)
**Amphetamine use disorders**	62402·42 (39643·03 to 91651·33)	55578·3 (36579·04 to 79390·68)	113·22 (72·82 to 164·04)	112·58 (71·69 to 163·52)	-0·56 (-8·71 to 8·58)	11504·24 (7676·35 to 15963·69)	9574·54 (6744·29 to 12859·38)	20·9 (14·1 to 28·68	20·83 (14·2 to 28·7	-0·35 (-7·18 to 8·6)
**Cannabis use disorders**	248118·02 (157571·61 to 372474·87)	219443·1 (152071·97 to 307395·27)	459·78 (297·76 to 680·96)	461·5 (298·75 to 683·19)	0·37 (0·11 to 0·62)	39231·82 (26463·37 to 60415·44)	29819·23 (21306·35 to 43940·88)	73·9 (50·35 to 111·65	74·08 (50·45 to 112·04	0·25 (0·1 to 0·41)
**Other drug use disorders**	10691·32 (7654·6 to 14510·05)	16591·78 (11839·95 to 22172·21)	22·17 (15·98 to 29·79)	25·21 (18·12 to 33·71)	13·69 (1·78 to 24·9)	55912·43 (39608·2 to 75295·86)	76151·01 (54120·31 to 102591·02)	113·54 (80·83 to 151·42	121·94 (84·5 to 165·56	7·4 (-3·32 to 17·04)
**Diabetes and kidney diseases**	3329075·84 (3151343·82 to 3522038·08)	8303565·76 (7926557·8 to 8686028·34)	9369·94 (8888·2 to 9839·44)	10171·89 (9713·34 to 10629·1)	8·56 (4·69 to 12·75)	151136·6 (142769·24 to 160623·68)	417570·65 (393937·04 to 442073·38)	435·36 (410·26 to 466·23	513·47 (487·48 to 542·32	17·94 (12·25 to 23·77)
**Diabetes mellitus**	1280303·29 (1185654·25 to 1378836·5)	4216438·64 (3884082·95 to 4596810·99)	3697·54 (3428·37 to 3979·42)	4986·94 (4602·47 to 5433·87)	34·87 (26·07 to 43·26)	79597·87 (73868·91 to 85596·45)	198196·4 (181001·8 to 216676·9)	196·04 (182·61 to 210·08	251·97 (233·67 to 273·63	28·53 (21·08 to 36·54)
**Diabetes mellitus type 1**	163926·03 (130879·51 to 199864·48)	238857·31 (194478·49 to 292531·9)	358·55 (287·07 to 436·4)	394·31 (318·13 to 484·71)	9·97 (2·06 to 18·33)	3530·14 (2823·47 to 4280·36)	2917·12 (2430·6 to 3449·54)	8·57 (6·87 to 10·42	9·57 (7·86 to 11·67	11·67 (4·07 to 20·55)
**Diabetes mellitus type 2**	1116377·27 (1023255·9 to 1213718·51)	3977581·33 (3634911·46 to 4347288·78)	3338·99 (3069·5 to 3624·5)	4592·64 (4191·1 to 5017·88)	37·55 (27·67 to 47·06)	76067·73 (70543·96 to 81875·58)	195279·28 (178361·61 to 213857·88)	187·47 (174·21 to 201·66	242·4 (224·41 to 263·45	29·3 (21·5 to 37·65)
**Chronic kidney disease**	2418698·87 (2228405·08 to 2617929·62)	5531529·85 (5230407·84 to 5841387·91)	7003·73 (6498·76 to 7541·39)	6855·36 (6492·3 to 7224·91)	-2·12 (-6·63 to 2·78)	61658·54 (55552·89 to 69099·49)	210090·9 (193312·38 to 227333·85)	216·8 (196·41 to 241·73	238·61 (220·54 to 256·88	10·06 (1·24 to 18·45)
**Chronic kidney disease due to diabetes mellitus type 1**	30752·6 (19518·88 to 47048·97)	41020·28 (29821·75 to 55030·31)	64·49 (43·24 to 95·72)	65·79 (46·04 to 93·69)	2·02 (-8·58 to 14·42)	500·54 (256·14 to 952·16)	716·13 (465·86 to 1067·82)	1·23 (0·66 to 2·28	1·17 (0·68 to 2·06	-5·04 (-14·87 to 11·4)
**Chronic kidney disease due to diabetes mellitus type 2**	435588·37 (385700·28 to 490440·16)	1047141·41 (974139·9 to 1127293·57)	1273·05 (1146·93 to 1403·66)	1241·2 (1150·53 to 1339·9)	-2·5 (-7·35 to 2·92)	8014·35 (6976·67 to 9230·57)	29455·01 (26593·66 to 32189·85)	28·61 (25·21 to 32·59	32·36 (29·26 to 35·37	13·09 (2·58 to 23·53)
**Chronic kidney disease due to hypertension**	85710·16 (75383·3 to 96621·2)	305076·57 (279399·32 to 330438·27)	327·98 (293·56 to 365·9)	355·83 (324·78 to 386·36)	8·49 (0·67 to 16·54)	4913·17 (4278·01 to 5637·16)	19378·27 (17547·71 to 21335·2)	18·84 (16·63 to 21·38	21·65 (19·62 to 23·81	14·91 (5·7 to 23·58)
**Chronic kidney disease due to glomerulonephritis**	63407·11 (49403·02 to 81784·32)	86970·41 (73456·16 to 103110·38)	144·29 (114·6 to 179·34)	132·65 (107·54 to 164·69)	-8·07 (-12·73 to -3·37)	2120·57 (1576·06 to 2883·44)	3180·31 (2616·72 to 3826·7)	5·34 (4·07 to 7·03	4·94 (3·86 to 6·32	-7·61 (-13·65 to -0·61)

Chronic kidney disease due to other and unspecified causes	1803240·63 (1656437·05 to 1960310·12)	4051321·18 (3837176·04 to 4276061·98)	5193·92 (4804·13 to 5606·71)	5059·9 (4797·56 to 5352·74)	-2·58 (-7·09 to 2·4)	46109·92 (41471·48 to 51785·12)	157361·18 (144829·43 to 170331·26)	162·78 (147·65 to 181·97)	178·49 (164·92 to 192·81)	9·65 (0·95 to 18·13)
Acute glomerulonephritis	667·37 (477·09 to 895·46)	624·36 (472·53 to 789·3)	1·51 (1·09 to 2·05)	1·54 (1·11 to 2·05)	2·12 (-4·49 to 8·48)	9880·2 (7073·04 to 13415·55)	9283·35 (7027·67 to 11903·34)	22·52 (16·28 to 31·07	22·89 (16·54 to 30·93	1·65 (-3·85 to 6·68)
Skin and subcutaneous diseases	11164945·33 (10862554·6 to 11479883·59)	15404970·79 (15022681·04 to 15787186·1)	26333·01 (25677·58 to 27022·62)	27484·74 (26814·75 to 28162·68)	4·37 (3·27 to 5·58)	23327830·34 (22487616·39 to 24264164·69)	32777309·68 (31398930·87 to 34330353·6)	56413·78 (54469·69 to 58673·28	57522·7 (55599·3 to 59757·45	1·97 (0·99 to 2·96)
Dermatitis	1940512·06 (1834659·87 to 2049630·01)	1893505·7 (1800206·77 to 1985043·2)	4594·76 (4358·27 to 4843·14)	4618·53 (4369·5 to 4865·25)	0·52 (-3·06 to 4·44)	1504926·38 (1372571·65 to 1637952·28)	1819006·15 (1653250·34 to 1989222·1)	3307·65 (3033·92 to 3582·35	3300·27 (3020·56 to 3572·36	-0·22 (-0·94 to 0·51)
Atopic dermatitis	1688076·43 (1586225·97 to 1790375·21)	1505877·11 (1421559·43 to 1588919·6)	4010·99 (3775·71 to 4252·66)	4036·29 (3801·42 to 4273·94)	0·63 (-3·34 to 4·91)	184101·95 (169867·24 to 199185·87)	183563·49 (171268·65 to 196193·05)	473·22 (436·19 to 513·63	475·65 (437·09 to 517·9	0·51 (-4·25 to 5·79)
Contact dermatitis	141717·34 (112885·84 to 175962·61)	213984 (170171·11 to 263351·57)	325·63 (264·86 to 399·51)	323·83 (263·41 to 396·78)	-0·55 (-0·81 to -0·32)	394311·98 (300721·93 to 489505·49)	600062·33 (466655·12 to 737424·55)	905·48 (714·36 to 1104·07	900·53 (711·19 to 1098·62	-0·55 (-0·78 to -0·37)
Seborrhoeic dermatitis	128457·83 (118171·02 to 138408·43)	190784·59 (177523·76 to 204979·63)	301·72 (280·64 to 321·99)	302·52 (281·76 to 322·61)	0·27 (0·11 to 0·45)	926512·45 (839617·04 to 1009238·12)	1035380·33 (946341·37 to 1127844·43)	1928·95 (1762·95 to 2097·98	1924·09 (1759·01 to 2091·48	-0·25 (-0·34 to -0·16)
Psoriasis	128954·98 (124006·55 to 133566·9)	171174·52 (164493·04 to 177834·41)	298·59 (287·87 to 308·4)	259·43 (249·92 to 269·08)	-13·12 (-15·12 to -11·14)	19576·17 (18789·94 to 20436·91)	24621·31 (23554·7 to 25645·63)	44·19 (42·5 to 45·92	39·9 (38·31 to 41·5	-9·7 (-11·59 to -7·7)
Bacterial skin diseases	310012·42 (294434·19 to 325754·58)	317762·42 (303855·12 to 330915·88)	705·62 (670·6 to 740·41)	712·09 (678·22 to 744·25)	0·92 (-3·86 to 6·19)	8025497·84 (7733280·41 to 8332927·46)	8029684·4 (7782794·84 to 8300087·71)	18286·74 (17601·46 to 19019·92	18376·35 (17697·81 to 19130·64	0·49 (-2·14 to 3)
Cellulitis	18385·33 (17122·48 to 19737·15)	23730·47 (22267·14 to 25294·1)	43·49 (40·62 to 46·59)	44·43 (41·44 to 47·8)	2·16 (-1·12 to 5·58)	438040·3 (406165·96 to 469221·78)	530894·89 (496272·74 to 568478·95)	1029·44 (956·81 to 1101·9	1038·9 (964·18 to 1117·97	0·92 (-2·58 to 4·27)
Pyoderma	295657·85 (284605·41 to 307035·41)	298212·12 (289308·63 to 307917·83)	670·69 (644·85 to 697·21)	675·06 (649·68 to 700·22)	0·65 (-1·89 to 3·14)	7587457·54 (7296009·76 to 7899124·68)	7498789·51 (7249431·42 to 7764934·67)	17257·31 (16584·45 to 17983·9	17337·46 (16641·52 to 18094·54	0·46 (-2·36 to 3·19)
Scabies	40863·78 (35225·69 to 47222·45)	40745·05 (35896·83 to 45715·03)	88·39 (76·76 to 101·87)	89·64 (78·29 to 103·22)	1·41 (-1·4 to 4·86)	122795·92 (106110·77 to 142218·28)	122193·8 (107472·38 to 136445·22)	267·02 (232·05 to 306·07	270·77 (235·19 to 310·7	1·4 (-1·47 to 4·9)
Fungal skin diseases	2122229·3 (1883610·74 to 2413010·86)	3701138·46 (3297331·43 to 4174523·18)	5591·7 (5010·09 to 6275·03)	5483 (4894·04 to 6170·61)	-1·94 (-2·93 to -1·22)	7005472·6 (6294012·01 to 7902031·8)	12342526·86 (10964786·49 to 13819524·96)	18423·09 (16639·39 to 20485·15	18164·85 (16379·97 to 20263·69	-1·4 (-2·05 to -0·87)
Viral skin diseases	1690260·18 (1620600·67 to 1757827·02)	1668992·25 (1608869·96 to 1730242·23)	3819·92 (3664·24 to 3967·23)	3813·74 (3657·29 to 3961·51)	-0·16 (-0·21 to -0·11)	720277·32 (690533·62 to 750874·55)	645892·22 (624124·93 to 669230·37)	1722·19 (1649·28 to 1797·48	1722·36 (1649 to 1797·61	0·01 (-0·04 to 0·06)

**Acne vulgaris**	2030079·07 (1788422·07 to 2291129·83)	1867281·48 (1695751·86 to 2041184·05)	3935·08 (3474·94 to 4432·17)	4783·55 (4230·83 to 5321·14)	21·56 (15·59 to 28·4)	1014774·87 (870310·37 to 1186602·69)	883301·77 (778872·21 to 999926·14)	2050·59 (1743·85 to 2421·5	2508·65 (2127·21 to 2945·88	22·34 (14·98 to 31·92)
**Alopecia areata**	134279·22 (129006·73 to 139538·81)	170177·61 (164108·15 to 176338·17)	287·33 (276·97 to 297·58)	282·71 (272·54 to 292·91)	-1·61 (-1·71 to -1·51)	238072·98 (228521·74 to 247612·5)	298085·17 (287509·99 to 308341·91)	507·45 (489·25 to 526·08	499·28 (481·11 to 517·94	-1·61 (-1·71 to -1·51)
**Pruritus**	280446·83 (246303·59 to 318471·93)	460685·52 (404031·59 to 529132·14)	669·48 (593·54 to 753·52)	714·64 (634·23 to 813·11)	6·75 (2·32 to 10·81)	219434·57 (193658·87 to 247144·18)	353948·53 (310849·42 to 400221·98)	524·42 (468·45 to 585·42	554·53 (494·88 to 622·56	5·74 (2·3 to 8·95)
**Urticaria**	330376·52 (289198·65 to 377829·24)	350725·58 (309937·5 to 392025·74)	760·06 (664·45 to 864·54)	752·2 (657·19 to 856·47)	-1·03 (-1·19 to -0·89)	579890·73 (508387·27 to 664364·24)	613810·39 (547341·64 to 682888·01)	1340·63 (1181·37 to 1522·99	1326·96 (1170·27 to 1507·1	-1·02 (-1·17 to -0·86)
**Decubitus ulcer**	3812·04 (3427·24 to 4210·34)	13314·32 (11868·73 to 14940·89)	13·47 (12·2 to 14·84)	16·93 (15·13 to 18·99)	25·75 (19·11 to 36)	13946·01 (12608·65 to 15433·85)	48750·39 (43676·26 to 55041·37)	49·68 (45·18 to 54·69	61·82 (55·62 to 69·17	24·45 (18·33 to 33·14)
**Other skin and subcutaneous diseases**	3715581·61 (3613992·58 to 3831261·42)	7329104·31 (7134706·57 to 7544757·27)	9514·66 (9263·46 to 9787·19)	10288·13 (10025·99 to 10573·61)	8·13 (6·37 to 9·84)	3863164·96 (3761507·62 to 3979317·24)	7595488·69 (7393868·56 to 7824635·88)	9890·14 (9633·24 to 10172·11	10696·96 (10437·46 to 10993·56	8·16 (6·44 to 9·88)
**Sense organ diseases**	5107613·49 (4871503·01 to 5329554·73)	11247741·16 (10819057·31 to 11663424·76)	14236·17 (13673·33 to 14780·05)	14239·23 (13690·22 to 14767·82)	0·02 (-1·48 to 2·06)	0 (0 to 0)	0 (0 to 0)	0 (0 to 0	0 (0 to 0	0 (0 to 0)
**Blindness and vision loss**	954842·25 (854116·39 to 1058929·85)	1754474·48 (1560812·32 to 1972198·83)	2693·98 (2430·45 to 2974·28)	2472·15 (2215·39 to 2738·39)	-8·23 (-11·42 to -4·75)	NA	NA	NA	NA	NA
**Glaucoma**	18870·2 (15484·91 to 22519·12)	49241·82 (41341·07 to 58854·8)	88·83 (73·57 to 104·97)	56·95 (47·98 to 68·41)	-35·88 (-40·76 to -30·36)	NA	NA	NA	NA	NA
**Cataract**	91339·72 (77978·6 to 106114·03)	297696·17 (248844·7 to 351240·61)	383·6 (327·48 to 444·47)	348·81 (292·37 to 409·24)	-9·07 (-14·73 to -2·81)	NA	NA	NA	NA	NA
**Age-related macular degeneration**	6672·16 (5255·39 to 8382·57)	18766·86 (15099·49 to 22618·74)	30·3 (24·13 to 37·4)	21·57 (17·44 to 25·97)	-28·81 (-35·32 to -22·25)	NA	NA	NA	NA	NA
**Refraction disorders**	578777·39 (505527·75 to 654585·24)	782430·63 (691422·44 to 871312·85)	1373·4 (1214·96 to 1534·87)	1329·65 (1175·23 to 1496·67)	-3·19 (-6·23 to -0·05)	NA	NA	NA	NA	NA
**Near vision loss**	167680·52 (111882·45 to 237437·12)	467037·01 (313793·69 to 663716·91)	579·53 (386·31 to 818·35)	530·85 (359·28 to 754·5)	-8·4 (-20·71 to 1·91)	NA	NA	NA	NA	NA
**Other vision loss**	112650·63 (97363·02 to 129930·5)	201306·65 (167844·77 to 240867·14)	332·61 (287·16 to 384·84)	263 (223·09 to 307·34)	-20·93 (-25·57 to -16·19)	NA	NA	NA	NA	NA

Age-related and other hearing loss	4243381·86 (4001563·08 to 4481862·26)	10549159·21 (9996785·42 to 11115258·18)	12613·69 (11948·09 to 13298·76)	12664·44 (12026·01 to 13316·06)	0·4 (-1·62 to 3·15)	NA	NA	NA	NA	NA
Other sense organ diseases	708249·66 (591901·89 to 856655·76)	1536351·52 (1256032·11 to 1907007·06)	1973·38 (1649·49 to 2388·21)	2048·94 (1719·51 to 2480·69)	3·83 (1·35 to 6·05)	0 (0 to 0)	0 (0 to 0)	0 (0 to 0	0 (0 to 0	0 (0 to 0)
Musculoskeletal disorders	8438783·43 (7900626·02 to 9025872·44)	17410454·78 (16514562·92 to 18365044·12)	21573·84 (20366·62 to 22819·98)	22540·31 (21314·27 to 23898·8)	4·48 (2·64 to 6·51)	1660807·27 (1496755·05 to 1851086·22)	2843205·72 (2588805·71 to 3132472·77)	3989·55 (3625·23 to 4388·18	3918·52 (3556·57 to 4294·92	-1·78 (-3·79 to 0·33)
Rheumatoid arthritis	70856·93 (63832·81 to 78890·41)	189457·85 (171890·97 to 209690·82)	184·95 (167·81 to 204·4)	229·73 (209·48 to 253·91)	24·21 (19·17 to 29·92)	4503·17 (4065·11 to 4999·91)	10055·35 (9082·6 to 11054·86)	10·46 (9·54 to 11·5	13·43 (12·25 to 14·69	28·41 (22·44 to 34·49)
Osteoarthritis	2984297·13 (2686531·72 to 3336252·32)	8433115·27 (7644912·95 to 9379717·74)	9433·19 (8558·46 to 10442·35)	9330·39 (8477·7 to 10352·75)	-1·09 (-2·98 to 0·91)	273982·69 (242234·94 to 310926·36)	636499·54 (565284·24 to 723340·69)	732·12 (651·73 to 824·64	734·4 (654·27 to 830·95	0·31 (-2·3 to 2·84)
Osteoarthritis hip	133592·89 (102234·74 to 169284·85)	429218·27 (328881·89 to 544547·98)	436·6 (333·75 to 547·14)	479·44 (366·85 to 605·31)	9·81 (4·23 to 15·56)	8352·31 (6243·59 to 10826·15)	21790·26 (15914·24 to 28761·15)	22·55 (16·63 to 29·18	24·67 (18·38 to 32·14	9·4 (3·67 to 15·23)
Osteoarthritis knee	1905987·45 (1645328·89 to 2197462·82)	5626789·31 (4844457·13 to 6460210·32)	6171·24 (5353·46 to 7067·76)	6211·13 (5360·4 to 7095·17)	0·65 (-3·28 to 5·01)	172392·88 (148971·31 to 196790·86)	415292·51 (358355·97 to 474450·38)	471·53 (409·73 to 535·53	474·85 (413·34 to 539·64	0·7 (-3·32 to 4·62)
Osteoarthritis hand	1419251·84 (1067896·41 to 1836832·43)	3863791·6 (2913744·88 to 5070049·26)	4552·75 (3451·69 to 5930·43)	4254·26 (3222·53 to 5529·72)	-6·56 (-6·95 to -6·17)	80058·32 (60396·85 to 104361·88)	166224·79 (124233·48 to 219366·78)	200·8 (152·26 to 260·34	196·59 (149·15 to 256·03	-2·09 (-2·66 to -1·57)
Osteoarthritis other	190593·45 (143228·5 to 246078·67)	595936·6 (450713·68 to 756180·61)	641·71 (491·67 to 815·8)	663·2 (504·09 to 839·98)	3·35 (-0·64 to 7·57)	13179·19 (10188·49 to 16454·12)	33191·98 (26277·47 to 40843·47)	37·24 (29·61 to 45·29	38·29 (30·56 to 46·98	2·83 (-1·15 to 6·8)
Low back pain	3887100·2 (3367766·78 to 4465936·17)	6291274·33 (5470612·66 to 7244066·77)	9341·54 (8199·35 to 10653·59)	8735 (7622·04 to 9958·81)	-6·49 (-10·09 to -2·6)	1194364·18 (1043433·17 to 1366994)	1846856·3 (1610991·29 to 2108698·76)	2788·54 (2445·89 to 3158·29	2704·08 (2371·06 to 3074·69	-3·03 (-5·89 to -0·17)
Neck pain	698510·34 (547488·59 to 900822·7)	1330805·2 (1035486·92 to 1716308·63)	1735·86 (1372·76 to 2219·94)	1719·45 (1361·87 to 2196·17)	-0·95 (-1·26 to -0·59)	151475·5 (117413·74 to 198529·73)	258217·02 (201520·01 to 332444·35)	358·31 (280·32 to 460·26	356·64 (279·43 to 458·59	-0·47 (-0·66 to -0·29)
Gout	209144·34 (162546·09 to 263023·16)	601148·04 (467700·5 to 768411·65)	615·07 (482·62 to 771·63)	698·03 (548·8 to 878·61)	13·49 (7·35 to 19·83)	36481·72 (28725·52 to 45745)	91577·51 (71910·27 to 115771·72)	100·12 (79·77 to 125·91	109·97 (87·23 to 137·69	9·84 (4·36 to 15·98)
Other musculoskeletal disorders	2292897·2 (1860289·85 to 2761899·01)	5296449·4 (4421643·99 to 6325037·6)	5253·03 (4249·12 to 6340·15)	7314·98 (6206·6 to 8648·15)	39·25 (28·98 to 51·61)	NA	NA	NA	NA	NA
Other non-communicable diseases	25980809·88 (24589770·18 to 27445689·31)	32818562·79 (31093282·56 to 34666377·39)	59193·31 (56246·36 to 62294·74)	57300·87 (54226·14 to 60466·73)	-3·2 (-5·1 to -1·21)	31188318·16 (27926991·45 to 34256947·49)	33123917·16 (30323502·88 to 36159202·48)	68743·57 (61842·53 to 75621·2	69295·91 (62149·75 to 76307·57	0·8 (-3·39 to 4·75)

**Congenital birth defects**	352881·39 (304727·88 to 413937·39)	279185·6 (243413·47 to 316934·69)	848·74 (732·08 to 992·57)	806·04 (695·72 to 934·75)	-5·03 (-11·93 to 2·49)	54577·92 (44406·06 to 66278·82)	27777·44 (22614·36 to 34037·73)	168·4 (137·01 to 204·5	141·04 (114·83 to 172·83	-16·25 (-22·91 to -8·76)
**Neural tube defects**	5340·03 (4224·29 to 6631·12)	6346·03 (5108·76 to 7642·08)	11·55 (9·17 to 14·32)	15·56 (12·61 to 18·87)	34·75 (18·43 to 54·02)	253·35 (212·31 to 301·17)	160·6 (135·93 to 187·16)	0·78 (0·66 to 0·93	0·82 (0·69 to 0·95	4·32 (-7·3 to 16·51)
**Congenital heart anomalies**	80100·31 (69221·04 to 93589·37)	74046·04 (64275·17 to 84554·5)	196·68 (168·59 to 231·48)	187·06 (162·09 to 217·99)	-4·89 (-11·14 to 1·45)	13421·31 (9923·59 to 18488·34)	6801·72 (5026·29 to 9425·43)	41·41 (30·62 to 57·05	34·54 (25·52 to 47·86	-16·6 (-26·29 to -4·62)
**Orofacial clefts**	20931·12 (16774·51 to 26030·21)	24662·67 (19151·7 to 30920·69)	50·21 (40·28 to 62·69)	50·45 (39·52 to 63·02)	0·48 (-16·63 to 18·62)	2221·41 (1391·85 to 3590·37)	811·87 (417·1 to 1429·2)	6·85 (4·29 to 11·08	4·12 (2·12 to 7·26	-39·86 (-63·32 to -10·63)
**Down syndrome**	24938·12 (19133·17 to 32614·09)	23374·74 (17773·73 to 29413·77)	52·56 (40·39 to 68·64)	62·97 (47·43 to 79·52)	19·8 (5·97 to 35·69)	756·7 (577·83 to 1002·88)	419·77 (319·61 to 536·65)	2·33 (1·78 to 3·09	2·13 (1·62 to 2·72	-8·71 (-20·84 to 4·81)
**Turner syndrome**	11480·87 (8434·36 to 14726·17)	8514·68 (6235·22 to 11151·38)	24·68 (18·19 to 31·59)	20·8 (15·16 to 27·42)	-15·72 (-26·28 to -3·76)	335 (237·04 to 444·91)	153·28 (109·13 to 203·95)	1·03 (0·73 to 1·37	0·78 (0·55 to 1·04	-24·7 (-34·3 to -12·93)
**Klinefelter syndrome**	262·25 (169·72 to 373·16)	235·47 (156·2 to 328·23)	0·59 (0·38 to 0·83)	0·65 (0·44 to 0·89)	9·75 (-4·69 to 27·87)	15·2 (9·87 to 21·86)	8·02 (5·37 to 11·14)	0·05 (0·03 to 0·07	0·04 (0·03 to 0·06	-13·21 (-25·54 to 0·31)
**Other chromosomal abnormalities**	41013·59 (33634·66 to 49901·83)	33455·52 (27175·34 to 40091·64)	91·79 (75·57 to 111·7)	97·24 (78·71 to 117·28)	5·93 (-3·77 to 16·01)	4893·5 (3993·91 to 6025·52)	2568·22 (2081·95 to 3103·73)	15·1 (12·32 to 18·59	13·04 (10·57 to 15·76	-13·63 (-21·18 to -5·69)
**Congenital musculoskeletal and limb anomalies**	184256·33 (137979·01 to 236714·63)	132261·24 (102066·3 to 164831·58)	437·66 (325·73 to 563·6)	379·4 (284·34 to 490·96)	-13·31 (-22·69 to -1·41)	23640·22 (14846·82 to 34308·23)	11239·66 (6892·53 to 16709·54)	72·94 (45·81 to 105·86	57·07 (35 to 84·84	-21·76 (-34·49 to -5·47)
**Urogenital congenital anomalies**	48502·27 (37784·61 to 60747·15)	36100·89 (28663·24 to 44498·4)	114·71 (88·51 to 144·5)	119·23 (94·02 to 149·15)	3·94 (-5·02 to 13·15)	5724·16 (3869·55 to 8156·33)	3622·72 (2443·9 to 5172·7)	17·66 (11·94 to 25·17	18·39 (12·41 to 26·26	4·15 (-7·91 to 17·75)
**Digestive congenital anomalies**	23118·51 (18223·89 to 28665·78)	28695·21 (23267·92 to 34602·31)	56·05 (44·19 to 69·09)	78·91 (64·13 to 96·22)	40·77 (23·41 to 63·82)	3317·06 (2401·68 to 4414·11)	1991·58 (1544·27 to 2546·52)	10·23 (7·41 to 13·62	10·11 (7·84 to 12·93	-1·19 (-18·88 to 19·44)
**Other congenital birth defects**	31947·02 (12866·48 to 64325·13)	27567·02 (8242·54 to 58539·37)	71·82 (28·94 to 144·55)	51·75 (15·48 to 109·89)	-27·94 (-48·33 to -14·05)	NA	NA	NA	NA	NA
**Urinary diseases and male infertility**	358280·77 (270281·18 to 484881·81)	760757·51 (591274·96 to 965195·18)	913·02 (708·88 to 1191·9)	1010·42 (789·3 to 1292·19)	10·67 (3 to 19·69)	3031159·83 (2704772·8 to 3375047·63)	4159043·51 (3682594·79 to 4621170·95)	6989·25 (6277·6 to 7704·77	7073·44 (6303·96 to 7779·49	1·2 (-5·14 to 7·94)
**Urinary tract infections and interstitial nephritis**	46768·8 (41384·74 to 52855·13)	57285·75 (50370·53 to 63914·62)	106·59 (94·79 to 118·61)	107·17 (94·76 to 119)	0·55 (-7·22 to 9·08)	2429486·75 (2147424·97 to 2743462·91)	2979225·26 (2615288·74 to 3325897·94)	5535·21 (4920·12 to 6160·95	5565·28 (4902·78 to 6173	0·54 (-7·43 to 8·95)

**Urolithiasis**	22004·06 (16961·67 to 27825·35)	42737·08 (32511·28 to 53947·01)	52·97 (40·93 to 66·82)	54·77 (42·66 to 68·28)	3·39 (-0·18 to 7·12)	583397·62 (450071·82 to 737520·41)	1119401·4 (848693·61 to 1416107·03)	1397·08 (1078·97 to 1754·82	1443·72 (1109·59 to 1807·66	3·34 (-0·28 to 7·05)
**Benign prostatic hyperplasia**	123828·76 (89253·36 to 169150·22)	467200·8 (339743·92 to 624932·64)	428·62 (309·44 to 578·05)	505·82 (369·08 to 675·23)	18·01 (6·3 to 31·9)	18275·46 (13196·31 to 24693·49)	60416·85 (43717·34 to 81639·56)	56·96 (41·34 to 75·77	64·44 (46·45 to 86·82	13·14 (1·37 to 25·72)
**Male infertility**	171121·4 (92648·87 to 284567·76)	205459·71 (106845·92 to 368165·84)	339·51 (180·59 to 585·93)	358·61 (192·6 to 613·79)	5·63 (-3·22 to 16·6)	NA	NA	NA	NA	NA
**Other urinary diseases**	NA	NA	NA	NA	NA	NA	NA	NA	NA	NA
**Gynecological diseases**	7234986·2 (6387374·78 to 8109723·38)	7971374·89 (7091438·51 to 8864392·38)	14768·75 (13109·87 to 16453·94)	13909·35 (12340·8 to 15537·98)	-5·82 (-8·08 to -3·36)	2766297·16 (2377632·57 to 3237738·98)	2776968·05 (2391098·04 to 3187650·84)	5535·41 (4789·35 to 6396·33	5154·26 (4458·7 to 5951·85	-6·89 (-8·88 to -4·77)
**Uterine fibroids**	813619·69 (614696·82 to 1077420·54)	1189124·66 (901216·55 to 1560667·56)	1724·28 (1306·84 to 2277·33)	1799·72 (1355·9 to 2381·77)	4·38 (-3·69 to 12·82)	40017·32 (29562·91 to 54586·14)	40267·78 (29588·79 to 54169·19)	74·4 (55·19 to 100·11	77·18 (56·57 to 104·46	3·74 (-5·26 to 13·1)
**Polycystic ovarian syndrome**	488973·69 (320403·84 to 673526·19)	799573·75 (537084·31 to 1126392·67)	940·14 (617·31 to 1300·86)	1492·47 (1005·69 to 2088·6)	58·75 (40·29 to 79·28)	20316·45 (13032·68 to 28869·9)	19236·57 (12856·41 to 27651·55)	38·8 (25·07 to 54·3	62·69 (41·6 to 88·53	61·56 (41·08 to 84·64)
**Female infertility**	87610·32 (9327·09 to 297770·55)	107069·84 (10330·02 to 366103·98)	184·35 (18·53 to 644·09)	173·51 (17·84 to 585·83)	-5·88 (-53·8 to 74·4)	NA	NA	NA	NA	NA
**Endometriosis**	193187·46 (132921·55 to 276768·18)	178370·58 (125812·29 to 240123·01)	377·55 (264·09 to 529·67)	310·75 (219·12 to 426·53)	-17·69 (-24·57 to -10·41)	30868·26 (21633·61 to 44638·07)	24850·47 (17959·47 to 34388·55)	58·6 (41·4 to 83·95	49·73 (35·3 to 70·1	-15·13 (-23·31 to -7·24)
**Genital prolapse**	242988·76 (179820·65 to 314083·17)	565637·65 (426187·81 to 736564·05)	829·65 (628·44 to 1073)	629·81 (479·42 to 813·92)	-24·09 (-30·04 to -17·71)	32145·45 (24010·12 to 42117·77)	67607·2 (50438·99 to 88419·09)	98·47 (73·29 to 129·9	75·93 (57·06 to 98·39	-22·89 (-28·46 to -17·39)
**Premenstrual syndrome**	5576929·01 (4509373·9 to 6756347·72)	5276743·09 (4236351·39 to 6414420·2)	10940·7 (8863·45 to 13188·55)	9872·73 (7954·88 to 11948·4)	-9·76 (-13·49 to -6·46)	1519114·45 (1210638·45 to 1828293·65)	1293865·07 (1025255·35 to 1565001)	2947·43 (2354·34 to 3528·55	2687·47 (2133·67 to 3215·22	-8·82 (-12·39 to -4·71)
**Other gynecological diseases**	1260373 (1007883·18 to 1623199·55)	1509008·01 (1217384·3 to 1838370·02)	2624·62 (2117·84 to 3277·56)	2484·52 (2006·51 to 3084·29)	-5·34 (-7·51 to -3·32)	1123835·24 (867818·52 to 1473138·34)	1331140·97 (1043083·29 to 1637418·45)	2317·71 (1833·27 to 2940·14	2201·26 (1733·27 to 2797·66	-5·02 (-6·11 to -4·02)
**Hemoglobinopathies and hemolytic anaemias**	3871954·13 (3495425·65 to 4270994·56)	4115679·58 (3666393·51 to 4571293·53)	8862·72 (7993·14 to 9786·74)	7572·25 (6764·16 to 8414·54)	-14·56 (-18·4 to -10·66)	51733·21 (45829·7 to 58153·69)	29653·28 (26305·17 to 33181·37)	159·62 (141·41 to 179·43	150·57 (133·57 to 168·48	-5·67 (-9·77 to -1·25)
**Thalassemias**	2108·25 (1487·54 to 3005·45)	1131·2 (831·12 to 1533·69)	5·46 (3·82 to 7·87)	4·67 (3·39 to 6·43)	-14·51 (-24·24 to -4·27)	308·04 (202·47 to 476·56)	155·55 (108·67 to 228·38)	0·95 (0·62 to 1·47	0·79 (0·55 to 1·16	-16·9 (-27·88 to -3·61)

**Thalassemias trait**	204088·43 (160747·14 to 251719·4)	440446·69 (336388·69 to 538907·83)	463·37 (364·54 to 571·2)	823·51 (628·6 to 1007·29)	77·72 (58·79 to 96·75)	3181·36 (2508·35 to 3929·25)	3395·28 (2591·18 to 4152·55)	9·82 (7·74 to 12·12	17·24 (13·16 to 21·09	75·63 (55·92 to 96·33)
**Sickle cell disorders**	49·82 (33·93 to 69·56)	32·65 (21·72 to 45·64)	0·12 (0·08 to 0·16)	0·11 (0·08 to 0·15)	-4·81 (-17·68 to 9·89)	6·79 (4·66 to 9·55)	3·93 (2·69 to 5·53)	0·02 (0·01 to 0·03	0·02 (0·01 to 0·03	-4·93 (-16·68 to 10·21)
**Sickle cell trait**	201730·68 (162004·32 to 246824·94)	246833·31 (197129·76 to 298345·09)	451·46 (362·23 to 552·41)	465 (371·98 to 561·95)	3 (-3·7 to 19·09)	3300·65 (2670·9 to 4050·4)	1922·43 (1538·81 to 2323·13)	10·18 (8·24 to 12·5	9·76 (7·81 to 11·8	-4·15 (-10·33 to 10·09)
**G6PD deficiency**	216099·61 (163592·77 to 277042·23)	213422·4 (160002·47 to 274615·09)	486·68 (368·31 to 624·03)	400·46 (300·28 to 515·5)	-17·72 (-23·5 to -11·7)	3467·59 (2627·89 to 4444·6)	1656·15 (1241·73 to 2131·84)	10·7 (8·11 to 13·71	8·41 (6·31 to 10·82	-21·4 (-26·89 to -15·68)
**G6PD trait**	2748039·75 (2405972·71 to 3099502·3)	2966430·95 (2579151·84 to 3363450·93)	6296·72 (5512·93 to 7102·05)	5484·64 (4768·6 to 6218·69)	-12·9 (-17·38 to -8·14)	41468·78 (36306·88 to 46772·46)	22519·95 (19579·88 to 25533·97)	127·95 (112·02 to 144·32	114·35 (99·42 to 129·65	-10·63 (-15·23 to -5·75)
**Other hemoglobinopathies and hemolytic anemias**	593112·94 (553379·17 to 631131·97)	333315·55 (281241·96 to 388435·64)	1378·59 (1297·04 to 1459·04)	551·63 (470·32 to 644·26)	-59·99 (-66·59 to -52·71)	NA	NA	NA	NA	NA
**Endocrine, metabolic, blood, and immune disorders**	3385092·69 (2941133·65 to 3924178·97)	5320304·09 (4497661·91 to 6331100·75)	7990·68 (6988·84 to 9177·02)	7627·78 (6554·24 to 8948·7)	-4·54 (-9·09 to 0·75)	0 (0 to 0)	0 (0 to 0)	0 (0 to 0	0 (0 to 0	0 (0 to 0)
**Oral disorders**	18521211·83 (16653426·3 to 20588356·77)	24199690·35 (21868667·17 to 26726575·63)	43207·74 (39149·5 to 47643·92)	41917·46 (38075·06 to 46092·45)	-2·99 (-6·51 to 0·56)	25284550·03 (22154768·45 to 28415044·66)	26130474·88 (23421246·03 to 29079172·8)	55890·89 (49098·67 to 62757·34	56776·6 (49907·68 to 63855·98	1·58 (-3·64 to 6·56)
**Caries of deciduous teeth**	3201504·3 (2541729·38 to 3847911·58)	1947803·93 (1543151·91 to 2372289·72)	8439·55 (6650·21 to 10120·49)	8279·79 (6525·11 to 10082·77)	-1·89 (-7·88 to 6·45)	6987931·12 (4732210·73 to 9041370·38)	4288457·38 (2945875·24 to 5488216·46)	17556·76 (12216·49 to 22566·98	17774·61 (12318·21 to 22719·31	1·24 (-5·24 to 7·57)
**Caries of permanent teeth**	12582366·01 (10594461·72 to 14848039·03)	14991453·62 (12687088 to 17528845·71)	27105·25 (22990·1 to 31730·31)	25187·4 (21464·57 to 29640·2)	-7·08 (-13·69 to -0·78)	17873824·42 (15613539·66 to 19897166·92)	20965618·34 (18636283·1 to 23830759·75)	37235·39 (32667·95 to 41437·11	37889·98 (33321·52 to 42566·24	1·76 (-5·78 to 8·49)
**Periodontal diseases**	3513713·92 (2490779·95 to 4830138·21)	8463953·67 (6318881·78 to 10969435·1)	9288·16 (6838·57 to 12471·97)	10172·7 (7512·4 to 13324·89)	9·52 (3·93 to 15·67)	346436·05 (257595·63 to 458520·48)	700211·4 (553551·12 to 856198·79)	857·04 (661·76 to 1088·37	908·81 (707·13 to 1137·74	6·04 (1·56 to 11·78)
**Edentulism**	898382·42 (696811·64 to 1131789·56)	2225426·66 (1717457·14 to 2789251·9)	3094·36 (2414·11 to 3810·17)	2580·55 (2001·2 to 3218·43)	-16·6 (-20·39 to -13·29)	76358·44 (59065·34 to 94296·56)	176187·75 (135585·15 to 224169·8)	241·71 (186·49 to 296·86	203·21 (158·86 to 252·33	-15·93 (-19·56 to -12·3)
**Other oral disorders**	823044·82 (786694·66 to 863967·21)	1097261·2 (1047442·04 to 1153022·78)	1834·38 (1757·69 to 1922·34)	1826·82 (1749·98 to 1914·01)	-0·41 (-0·52 to -0·31)	NA	NA	NA	NA	NA
**Sudden infant death syndrome**	NA	NA	NA	NA	NA	NA	NA	NA	NA	NA

**Injuries**	14356835·08 (13478926·71 to 15439809·53)	3778·42 (2739·38 to 4971·54)	8·18 (5·74 to 10·88)	8·52 (6·09 to 11·37)	-8·14 (-9·65 to -6·34)	7487500·99 (6935766·71 to 8077115·94)	7234295·11 (6677692·13 to 7829810·96)	15963·22 (14874·48 to 17149·77)	14727·29 (13351·9 to 16195·42)	-7·74 (-11·58 to -3·63)
**Transport injuries**	1343219·66 (1254276·33 to 1425191·35)	317320·64 (269741·68 to 369313·71)	497·45 (420·05 to 582·17)	450·49 (379·21 to 528·34)	-47·45 (-48·33 to -46·33)	581100·03 (510128·42 to 658623·85)	372101·49 (323203·81 to 422525·29)	1260·21 (1118·68 to 1423·33)	653·48 (562·12 to 753·32)	-48·15 (-53·7 to -41·62)
**Road injuries**	1232874·41 (1147429·51 to 1310709·68)	3833·82 (2330·17 to 5954·26)	3·26 (2·25 to 4·55)	5·43 (3·29 to 8·47)	-50·03 (-51·01 to -49·03)	552819·72 (482987·65 to 631491·52)	346335·91 (298494·72 to 397099·74)	1192·2 (1053·53 to 1354·93)	599·53 (510·39 to 694·56)	-49·71 (-55·4 to -42·79)
**Pedestrian road injuries**	280760·26 (239647·46 to 320512·94)	336830·33 (283742·11 to 404633·37)	769·76 (647 to 926·23)	442·4 (372·32 to 533)	-62·4 (-63·63 to -61·02)	98315·98 (80877·05 to 119524·98)	54004·47 (43664·65 to 65504·33)	222·92 (186·41 to 266·71)	83·46 (66·82 to 102·83)	-62·56 (-67·37 to -57·11)
**Cyclist road injuries**	305119·07 (256014·79 to 369729·97)	56120·58 (50230·41 to 62571·65)	30·8 (27·52 to 34·53)	77·17 (68·34 to 86·88)	-42·53 (-44·13 to -40·75)	179963·54 (148276·61 to 217531·01)	111705·19 (90304·85 to 136527·1)	385·17 (320·64 to 462·27	211·52 (167·62 to 260·98	-45·09 (-53 to -36·06)
**Motorcyclist road injuries**	273869·85 (240945·1 to 314123·84)	180614·28 (151747·38 to 221486·25)	280·39 (235·5 to 344·65)	255·58 (212·94 to 314·98)	-50·37 (-51·9 to -48·61)	78956·83 (64355·32 to 96591·48)	50999·83 (41944·68 to 62463·49)	169·98 (140·58 to 205·75)	86·64 (69·4 to 108·12)	-49·03 (-56·1 to -40·67)
**Motor vehicle road injuries**	348858·46 (303279·64 to 409226·42)	12·88 (8·19 to 19·83)	0 (0 to 0)	0·02 (0·02 to 0·04)	-47·05 (-48·47 to -45·5)	178774·39 (145801·72 to 216162·79)	116118·81 (93673·94 to 140496·76)	377·64 (313·45 to 450·38	193·77 (152·7 to 235·67	-48·69 (-55·62 to -40·64)
**Other road injuries**	24266·77 (20539·24 to 28596·64)	20922·38 (14257·53 to 29776·6)	20·8 (14·85 to 28·83)	28·82 (19·42 to 41·74)	-30·8 (-32·79 to -28·54)	16808·98 (12207·88 to 23293·91)	13507·61 (8951·63 to 18857·3)	36·49 (26·61 to 49·25	24·14 (15·86 to 34·72	-33·84 (-44·79 to -21·87)
**Other transport injuries**	110345·25 (92830·57 to 128899·21)	6637487·84 (5709729 to 7607064·05)	8983·09 (7780·04 to 10233·95)	9359·27 (8050·27 to 10704·96)	-19·62 (-22·52 to -16·34)	28280·31 (24212·04 to 33536·68)	25765·58 (21382·03 to 31698·88)	68·01 (58·82 to 79·44	53·95 (42·61 to 69·87	-20·67 (-29·58 to -9·68)
**Unintentional injuries**	11981962·5 (11119840·16 to 13020610·25)	6979594·68 (6106681·55 to 8114940·98)	9448·3 (8192·27 to 11050·97)	9588·43 (8306·43 to 11165·65)	-4·45 (-6·38 to -2·5)	6699855·12 (6154143·74 to 7288196·51)	6637215·6 (6093354·59 to 7212827·61)	14287·5 (13198·96 to 15457·31	13640·64 (12232·94 to 15101·13	-4·53 (-8·71 to -0·09)
**Falls**	3825293·3 (3272301·57 to 4525256·8)	1626471·46 (1385840·84 to 1918837·29)	2705·57 (2335·72 to 3097·88)	2293·58 (1953·37 to 2696·69)	1·48 (-1·05 to 4·59)	2239509·2 (1986886·89 to 2528516·71)	2473658·86 (2195109·49 to 2785217·36)	4764·4 (4257·49 to 5359·44	4794·58 (4112·57 to 5659·62	0·63 (-6·37 to 7·58)
**Drowning**	11940·17 (10520·37 to 13586·95)	393898·84 (340308·17 to 445733·52)	571·48 (488·55 to 652·41)	582·26 (493·51 to 667·98)	150·51 (138·82 to 163·01)	3704·21 (3087·46 to 4366·96)	11592·4 (9373·94 to 14094·36)	8·78 (7·45 to 10·13	21·23 (17·05 to 25·95	141·8 (113·43 to 174·31)
**Fire, heat, and hot substances**	1140383·17 (983883·7 to 1311197·12)	45845·53 (26302·93 to 68945)	78·03 (39·79 to 125·15)	78·36 (39·91 to 126·16)	-15·23 (-19·37 to -10·13)	139258·33 (112013·58 to 169992·92)	113008·32 (86738·03 to 139921·82)	290·65 (237·32 to 346·9	249·59 (187·97 to 312·37	-14·12 (-24·1 to -4·38)
**Poisonings**	61383·53 (51595·6 to 72790·5)	321969·01 (272489·13 to 370743·19)	425·54 (357·82 to 495·78)	464·43 (390·2 to 541·26)	-20·55 (-23·81 to -16·5)	64453·65 (52396·91 to 79112·35)	46082·36 (34839·64 to 59265·18)	138·43 (114·13 to 168·5	110 (81·97 to 143·61	-20·54 (-31·65 to -9·45)

**Poisoning by carbon monoxide**	14223·53 (11385·92 to 17885·77)	22390070·85 (20996231·91 to 23943586·07)	34495·62 (32539·18 to 36907·08)	31689·18 (29657·62 to 34047·71)	-33·11 (-37·18 to -28·64)	18114·52 (12291·23 to 25019·34)	10768·5 (7140·58 to 15613·55)	38·58 (26·72 to 52·72	29·32 (18·77 to 43·5	-24·02 (-37·54 to -9·61)
**Poisoning by other means**	47160 (38228·04 to 57859·85)	1068927·49 (959793·99 to 1191736·16)	1946·31 (1723·5 to 2191·86)	1848·79 (1633·33 to 2086·04)	-16·86 (-20·74 to -12·2)	46339·13 (36439·05 to 58430·8)	35313·86 (26579·39 to 46311·84)	99·85 (80·67 to 124·11	80·69 (58·87 to 106·75	-19·19 (-30·74 to -7·55)
**Exposure to mechanical forces**	3767639·35 (3248538·99 to 4342208·26)	378412·11 (325160·54 to 442009·08)	905·74 (786·62 to 1058·86)	479·61 (416·57 to 562·86)	4·19 (1·09 to 7·48)	2275879·56 (1866177·83 to 2745702·73)	2347111·12 (1915186·74 to 2800823·2)	4773·6 (3970·98 to 5667·13	4805·19 (3770·66 to 5947·81	0·66 (-8·83 to 9·74)
**Unintentional firearm injuries**	28108·5 (23593·18 to 34404·82)	258932·35 (226103·99 to 294644·85)	681 (600·04 to 774·58)	338·01 (295·45 to 383·96)	19·09 (17·22 to 20·84)	8601·39 (5692·11 to 12500·42)	11441·24 (7757·54 to 16626·72)	18·77 (12·58 to 26·85	22·94 (14·76 to 34·27	22·21 (9·1 to 36·51)
**Other exposure to mechanical forces**	3739530·85 (3222499·13 to 4311040·75)	243709·7 (200562·07 to 292411·86)	377·56 (310·63 to 455·94)	346·98 (284·94 to 419·44)	4·07 (0·94 to 7·37)	2267278·17 (1858724·65 to 2734653·88)	2335669·88 (1903784·6 to 2786917·81)	4754·83 (3957·15 to 5647·33	4782·25 (3753·97 to 5924·73	0·58 (-8·96 to 9·67)
**Adverse effects of medical treatment**	3255·48 (2249·24 to 4366·72)	6577583·58 (5645624·75 to 7547545·47)	8914·67 (7716·94 to 10171·95)	9277·79 (7974·84 to 10623·53)	4·14 (-2·09 to 9·79)	42580·1 (32788·92 to 53828·5)	49451·2 (38822·09 to 61016·67)	106·97 (83·37 to 134·91	111·39 (86·84 to 140·09	4·13 (-2·07 to 9·81)
**Animal contact**	204471·29 (171186·24 to 240614·81)	32539·06 (27750·2 to 38382·58)	61·81 (52·46 to 72·54)	42·77 (36·42 to 50·27)	-9·44 (-10·79 to -8·24)	393494·77 (308901·63 to 519611·93)	387510·56 (307322·86 to 494088·49)	852·82 (673·71 to 1116·99	775·39 (608·36 to 1034·96	-9·08 (-13·26 to -4·79)
**Venomous animal contact**	48981·16 (39049·26 to 62022)	172683·95 (145710·62 to 202533·13)	294·33 (252·12 to 337·59)	236·58 (197·05 to 279·85)	-13·66 (-15·4 to -11·86)	124449·73 (87680·66 to 172642·83)	121868·57 (88523·97 to 163784·2)	270·6 (194·33 to 369·79	240·31 (171·21 to 332·95	-11·19 (-15·44 to -6·65)
**Non-venomous animal contact**	155490·13 (126677·46 to 191010·68)	3335632·03 (2738481·53 to 4115002·13)	6211·4 (5190·36 to 7544·22)	4908·49 (4013·72 to 6156·75)	-8·1 (-9·73 to -6·74)	269045·03 (206932·71 to 357152·71)	265641·98 (208663·64 to 346090·43)	582·22 (452·57 to 768·5	535·08 (412·43 to 718·07	-8·1 (-12·41 to -3·81)
**Foreign body**	239775·34 (202430·25 to 276665·41)	227465·46 (197270·77 to 258976·54)	762 (659·5 to 862·15)	286·5 (248·18 to 324·21)	1·89 (-0·29 to 3·85)	371191·71 (298948·36 to 460799·61)	380155·1 (310221·43 to 468011·14)	808·57 (665·25 to 995·4	823·24 (676·51 to 1014·42	1·81 (-0·4 to 4·02)
**Pulmonary aspiration and foreign body in airway**	27740·44 (22312·08 to 35171·27)	16179·24 (13745·02 to 19158·87)	11·49 (9·66 to 13·8)	23·21 (19·65 to 27·51)	-41·88 (-45·75 to -38·11)	15680·47 (12488·02 to 20309·9)	8640·31 (6813·99 to 10850·82)	39·72 (31·45 to 51·88	23·37 (17·4 to 32·04	-41·18 (-49·72 to -33·26)
**Foreign body in eyes**	35181·87 (16973·06 to 58059·06)	289070·73 (240783·84 to 355942·19)	459·03 (375·51 to 565·85)	402·81 (332·67 to 496·31)	0·42 (-0·19 to 0·81)	209606·42 (151041·4 to 284941·51)	227721·49 (164439·33 to 305623·65)	439·02 (324·13 to 593·49	442·81 (326·96 to 598·61	0·86 (0·64 to 1·12)
**Foreign body in other body part**	176853·02 (146477·26 to 208630·61)	66893·21 (56816·11 to 78980·71)	136·25 (113·04 to 163·88)	93·59 (79·13 to 111·2)	9·14 (6·96 to 11·09)	145904·82 (108566·32 to 189038·67)	143793·29 (114014·15 to 178869·08)	329·83 (247·23 to 423·39	357·07 (268·75 to 461·9	8·26 (3·29 to 13·21)
**Environmental heat and cold exposure**	116204·4 (96274·64 to 144315·73)	13209·72 (10591·9 to 16691·23)	31·95 (25·65 to 40·05)	21·37 (16·96 to 27·53)	-8·85 (-10·51 to -7·43)	41088·42 (31578·64 to 55435·67)	36817·34 (29429·96 to 48425·96)	87·51 (68·85 to 115·73	80·9 (61·97 to 108·37	-7·55 (-14·17 to -1·73)

**Exposure to forces of nature**	9483·21 (6668·08 to 13272·13)	55545·41 (45351·77 to 68854·64)	108·6 (88·85 to 132·16)	90·29 (72·54 to 113·83)	38·6 (21·03 to 61·23)	7183·72 (5976·82 to 8698·4)	0 (0 to 0)	15·82 (13·33 to 18·95	0 (0 to 0	-100 (-100 to -100)
**Other unintentional injuries**	2602709·39 (2157818·72 to 3184642·06)	68755·13 (58083·22 to 81895·09)	140·55 (119·14 to 165·47)	111·67 (93 to 135·03)	-20·98 (-24·77 to -16·65)	1121511·45 (933396·44 to 1332841·39)	791828·36 (631354·42 to 977263·26)	2439·96 (2063·12 to 2877·51	1869·14 (1462·94 to 2365·49	-23·39 (-32·14 to -15·18)
**Self-harm and interpersonal violence**	1055824·57 (936210·61 to 1187132·86)	26084·3 (21476·43 to 32041·28)	67·91 (55·43 to 83·74)	39·47 (32·03 to 49·65)	3·77 (1·83 to 5·8)	206545·84 (171253·68 to 245984·14)	224978·03 (196247·87 to 255926·02)	415·51 (349·22 to 487·09	433·17 (365·3 to 504·27	4·25 (-2·29 to 11·64)
**Self-harm**	125576·76 (100759·19 to 148511·7)	1234179·31 (1148493·34 to 1311413·13)	3180·31 (2964·9 to 3373·52)	1589·29 (1475·72 to 1692·68)	58·59 (50·82 to 69·03)	44809·65 (40229·21 to 50027·76)	94328·35 (88425·89 to 101740·01)	89·23 (80·75 to 99·22	157·93 (147·2 to 170·31	77 (65·9 to 88·84)
**Self-harm by firearm**	1598·38 (1363·69 to 1882·13)	387305·68 (313723·28 to 452236·94)	308·18 (248·8 to 362·38)	488·73 (394·37 to 571·67)	72·79 (66·59 to 79·8)	690·99 (441·01 to 1025·11)	2361·2 (1537·81 to 3495·59)	1·69 (1·09 to 2·48	2·9 (1·91 to 4·19	71·97 (39·11 to 109·2)
**Self-harm by other specified means**	123978·37 (99099·6 to 146838·71)	1460079·87 (1328041·77 to 1598929·98)	2257·75 (2025·76 to 2513·96)	2342·98 (2103·79 to 2602·57)	58·37 (50·46 to 68·98)	44118·65 (39583·23 to 49197·69)	91967·16 (86243·99 to 99275·07)	87·54 (78·98 to 97·04	155·03 (144·31 to 167·4	77·09 (65·89 to 89·15)
**Interpersonal violence**	928832·21 (809938·99 to 1061147·21)	6804·5 (5838·48 to 8068·18)	4·56 (3·9 to 5·37)	7·89 (6·78 to 9·35)	-5·01 (-6·16 to -3·97)	159269·45 (125029·92 to 197696·11)	130649·67 (102654·4 to 158628·4)	320·14 (254·81 to 390·41	275·25 (208·81 to 346·01	-14·02 (-22·39 to -4·35)
**Physical violence by firearm**	5010·74 (4175·69 to 6065·06)	380501·18 (307224·42 to 445915·44)	303·62 (244·37 to 358·16)	480·85 (386·37 to 564·2)	101·94 (92·15 to 110·1)	1338·19 (836·8 to 2205·58)	2767·3 (1912·04 to 3971·94)	2·95 (1·89 to 4·64	6·3 (4·11 to 9·8	113·17 (88·91 to 142·97)
**Physical violence by sharp object**	57596·11 (47415·29 to 69666·25)	696784·32 (601967·14 to 812780·33)	1339·53 (1132·53 to 1574·06)	1329·17 (1123·53 to 1564·37)	-31·31 (-33·89 to -28·96)	24629·27 (17105·29 to 33289·24)	15777·63 (10878·62 to 21745·92)	50·58 (36·36 to 67·38	34·29 (23·08 to 48·8	-32·2 (-43·13 to -21·27)
**Sexual violence**	671551·43 (562801·27 to 798791·3)	1406863·26 (1321217·09 to 1491490·41)	3474·63 (3259·83 to 3684·74)	1825·87 (1710·98 to 1935·59)	-0·77 (-0·91 to -0·63)	NA	NA	NA	NA	NA
**Physical violence by other means**	194673·93 (158227·44 to 242063·05)	59904·27 (50222·68 to 70796·72)	68·42 (57·93 to 82·36)	81·48 (68·95 to 97·66)	-12·25 (-14·88 to -9·64)	133301·99 (103537·89 to 167137·54)	112104·74 (87494 to 137240·61)	266·61 (210·12 to 327·98	234·66 (174·13 to 296·58	-11·98 (-20·32 to -2·06)
**Conflict and terrorism**	1415·6 (967·9 to 1995·45)	19585334·8 (18190694·53 to 21212829·8)	28891·13 (26913·44 to 31272·51)	27605·69 (25556·87 to 29979·6)	66·62 (36·54 to 97·31)	2466·75 (2102·95 to 2912·26)	0 (0 to 0)	6·14 (5·19 to 7·26	0 (0 to 0	-100 (-100 to -100)
**Executions and police conflict**	0 (0 to 0)	73610·94 (58973·48 to 91543·48)	119·89 (96·54 to 150·73)	103·51 (82·89 to 129·68)	0 (0 to 0)	0 (0 to 0)	0 (0 to 0)	0 (0 to 0	0 (0 to 0	0 (0 to 0)

Appendix table 7. Number and age-standardised rate of deaths and DALYs, and percentage change from 1990 to 2019 by for both sex causes. (DALY= Disability-adjusted life years)

Causes	Deaths (95% UI)					DALYs (95% UI)				
	Counts 1990	Counts 2019	Age-standardised estimates 1990	Age-standardised estimates 2019	Percentage change of age-standardised rates between 1990 to 2019 (%)	Counts 1990	Counts 2019	Age-standardised estimates 1990	Age-standardised estimates 2019	Percentage change of age-standardised rates between 1990 to 2019 (%)
All causes	237240·64 (235686·56 to 238816·95)	318630·55 (307760·77 to 330081·14)	928·57 (923·77 to 933·37)	391·13 (378·26 to 404·58)	-57·88 (-59·27 to -56·34)	11657874·37 (10560783·57 to 12914811·47)	12401335·16 (10776856·94 to 14299277·06)	32256·41 (29591·74 to 35278·38)	17191·57 (14738·48 to 19958·17)	-46·7 (-50·28 to -43·19)
Communicable, maternal, neonatal, and nutritional diseases	17748·85 (16711·54 to 19228·03)	21483·29 (14852·54 to 24299·51)	69·53 (64·39 to 77·6)	29·49 (21·2 to 33·26)	-57·6 (-72·52 to -52·07)	1202890·01 (1052701·86 to 1385118·98)	649590·23 (548226·84 to 754454·32)	3292·57 (2928·07 to 3730·62)	1217·08 (1039·7 to 1426·19)	-63·04 (-68·25 to -59·15)
HIV/AIDS and sexually transmitted infections	124·33 (81·99 to 203·04)	199·69 (168·4 to 248·26)	0·36 (0·23 to 0·6)	0·41 (0·27 to 0·65)	14·38 (-8·23 to 42·28)	14729·1 (9762·31 to 22565·94)	19449·61 (13899·11 to 26862·27)	36·93 (23·35 to 59·02)	41·46 (26·65 to 63·85)	12·25 (-8·42 to 41·91)
HIV/AIDS	51·91 (49·35 to 54·58)	138·19 (128·53 to 148·87)	0·13 (0·12 to 0·13)	0·18 (0·17 to 0·19)	42·01 (29·97 to 54·64)	2825·04 (2351·44 to 3159·15)	8886·09 (6634·78 to 12553·85)	6·55 (5·54 to 7·24)	12·56 (9·66 to 17·03)	91·84 (49·41 to 171·24)
HIV/AIDS - drug-susceptible tuberculosis	23 (21·42 to 24·28)	59·3 (50·77 to 65)	0·06 (0·05 to 0·06)	0·08 (0·07 to 0·08)	37·5 (16·83 to 53·69)	1296·74 (1042·35 to 1450·28)	2355·98 (2029·91 to 2579·67)	3 (2·46 to 3·33)	3·48 (3 to 3·8)	16·23 (-3·54 to 46·82)
HIV/AIDS– multidrug-resistant tuberculosis without extensive drug resistance	0·35 (0·05 to 1·34)	2·09 (0·25 to 8·05)	0 (0 to 0)	0 (0 to 0·01)	214·65 (-66·62 to 2553·2)	17·85 (2·72 to 70·47)	79·93 (9·55 to 312·11)	0·04 (0·01 to 0·16)	0·12 (0·01 to 0·46)	184·36 (-70·19 to 2294·72)
HIV/AIDS - extensively drug-resistant tuberculosis	0 (0 to 0)	0·55 (0·07 to 2·14)	0 (0 to 0)	0 (0 to 0)	NA	0 (0 to 0)	20·74 (2·48 to 79·25)	0 (0 to 0)	0·03 (0 to 0·12)	NA
HIV/AIDS resulting in other diseases	28·55 (27·14 to 30·02)	76·25 (70·69 to 82·89)	0·07 (0·07 to 0·07)	0·1 (0·09 to 0·11)	42·47 (29·97 to 55·84)	1510·46 (1293·29 to 1723·09)	6429·45 (4211·39 to 10130·57)	3·51 (3·04 to 3·95)	8·93 (6·05 to 13·36)	154·42 (80·6 to 285·95)
Sexually transmitted infections excluding HIV	72·42 (29·99 to 150·86)	61·5 (32·49 to 109·01)	0·23 (0·1 to 0·47)	0·23 (0·09 to 0·48)	-0·76 (-32·72 to 41·2)	11904·06 (6842·72 to 19795·52)	10563·52 (6303·48 to 17097·68)	30·39 (16·58 to 53·16)	28·9 (14·71 to 50·5)	-4·89 (-25·89 to 25·7)
Syphilis	62·43 (20·17 to 139·95)	42·9 (15·03 to 91·3)	0·19 (0·06 to 0·43)	0·21 (0·07 to 0·45)	8·33 (-28·51 to 63·54)	5537·24 (1795·24 to 12401·42)	3994·05 (1530·49 to 8238·01)	16·75 (5·29 to 37·81)	18·61 (6·15 to 40·05)	11·12 (-25·16 to 68·55)
Chlamydial infection	1·66 (1·43 to 2·02)	3·57 (2·37 to 4·57)	0·01 (0·01 to 0·01)	0 (0 to 0·01)	-40·44 (-64·31 to -21·19)	824·8 (494·59 to 1298·38)	778·75 (477·08 to 1221·1)	1·66 (1·03 to 2·61)	1·37 (0·83 to 2·15)	-17·61 (-31·84 to 0·61)

Gonococcal infection	5·72 (4·98 to 6·86)	11·09 (7·47 to 14·03)	0·02 (0·02 to 0·03)	0·01 (0·01 to 0·02)	-42·01 (-63·95 to -24·36)	521·46 (374·74 to 729·38)	442·24 (332·16 to 592·93)	1·18 (0·89 to 1·6)	0·74 (0·55 to 1·01)	-37·03 (-46·93 to -28·16)
Trichomoniasis	NA	NA	NA	NA	NA	1085·66 (416·99 to 2268·26)	1444·13 (568·9 to 3022·07)	2·32 (0·89 to 4·84)	2·17 (0·84 to 4·51)	-6·39 (-16·11 to 4·51)
Genital herpes	NA	NA	NA	NA	NA	2033·25 (712·96 to 4919·33)	2081·18 (660·3 to 5107·33)	4·64 (1·6 to 11·25)	2·87 (0·93 to 7·07)	-38·12 (-47·4 to -27·67)
Other sexually transmitted infections	2·61 (2·31 to 3·06)	3·94 (2·73 to 4·89)	0·01 (0·01 to 0·01)	0·01 (0 to 0·01)	-44·07 (-64·13 to -29·03)	1901·65 (1208·15 to 2878·3)	1823·17 (1134·72 to 2796·3)	3·84 (2·46 to 5·75)	3·13 (1·96 to 4·76)	-18·36 (-29·61 to -6·07)
Respiratory infections and tuberculosis	12234·26 (11639·27 to 13112·77)	18810·74 (12258·4 to 21461·75)	50·11 (46·54 to 55·68)	23·68 (15·52 to 27·05)	-52·74 (-72·65 to -46·16)	464349·07 (432108·62 to 502312·43)	313470·28 (237676·39 to 356224·35)	1342·79 (1257·32 to 1452·27)	439·11 (346·36 to 515·45)	-67·3 (-75·19 to -63·26)
Tuberculosis	7521·56 (7146·12 to 7929·74)	3154·24 (2733·44 to 3576·7)	26·78 (25·09 to 28·78)	3·83 (3·3 to 4·36)	-85·69 (-87·45 to -83·68)	257887·94 (244358·55 to 270950·63)	58306·51 (52526·48 to 64984·11)	714·22 (677·74 to 752·83)	71·97 (64·95 to 79·85)	-89·92 (-90·87 to -88·72)
Latent tuberculosis infection	NA	NA	NA	NA	NA	0 (0 to 0)	0 (0 to 0)	0 (0 to 0)	0 (0 to 0)	NA
Drug-susceptible tuberculosis	7427·95 (6976·53 to 7883·43)	3043·45 (2545·53 to 3497·33)	26·45 (24·62 to 28·64)	3·7 (3·07 to 4·25)	-86·02 (-88·23 to -83·87)	254777·19 (239617·08 to 269517·08)	56365·07 (48617·3 to 63160·06)	705·57 (662·12 to 745·9)	69·59 (60·34 to 77·65)	-90·14 (-91·45 to -88·87)
Multidrug-resistant tuberculosis without extensive drug resistance	93·61 (12·92 to 359·71)	87·64 (9·64 to 342·05)	0·33 (0·05 to 1·28)	0·11 (0·01 to 0·42)	-68·08 (-96·68 to 178·84)	3110·75 (443·41 to 12072·22)	1545·51 (181·1 to 6148·42)	8·64 (1·22 to 33·38)	1·9 (0·22 to 7·59)	-78·04 (-97·78 to 84·56)
Extensively drug-resistant tuberculosis	0 (0 to 0)	23·15 (2·67 to 93·77)	0 (0 to 0)	0·03 (0 to 0·11)	NA	0 (0 to 0)	395·93 (47·23 to 1622·07)	0 (0 to 0)	0·48 (0·06 to 1·99)	NA
Lower respiratory infections	4657·01 (4312·15 to 5273·98)	15634·6 (8905·55 to 18030·91)	23·02 (20·64 to 27·67)	19·82 (11·39 to 22·91)	-13·93 (-58·04 to 0·07)	150555·81 (139405·67 to 164376·01)	200483·28 (118361·09 to 226964·86)	501·25 (464·35 to 550·42)	251·68 (156 to 283·81)	-49·79 (-71·28 to -42·35)
Upper respiratory infections	54·65 (9·1 to 101·84)	21·39 (11·23 to 35·43)	0·3 (0·04 to 0·62)	0·03 (0·02 to 0·05)	-90·01 (-95·58 to -13·45)	45732·26 (28107·6 to 71453·62)	46800·87 (28333·51 to 73375·64)	104·52 (64·44 to 160·7)	98·84 (59·48 to 157·45)	-5·43 (-13·64 to 2·34)
Otitis media	1·04 (0·69 to 1·34)	0·51 (0·28 to 0·88)	0 (0 to 0)	0 (0 to 0)	-82·23 (-89·96 to -68·55)	10173·06 (5848·66 to 16779·27)	7879·63 (4420·44 to 13158·79)	22·81 (13·13 to 38·18)	16·61 (9·3 to 27·93)	-27·16 (-42·67 to -17·04)
Enteric infections	693·05 (389·26 to 1081·5)	999·79 (528·64 to 1835·7)	3·44 (1·83 to 5·8)	1·3 (0·7 to 2·4)	-62·24 (-72·74 to -41·45)	37690·54 (28984·16 to 48179·62)	42403·96 (31062·22 to 57505·84)	113·3 (84·39 to 150·12)	58·12 (43·52 to 78·12)	-48·71 (-58·92 to -36·03)
Diarrheal diseases	681·04 (378·58 to 1067·12)	990·9 (517 to 1825·44)	3·41 (1·79 to 5·76)	1·28 (0·68 to 2·38)	-62·32 (-72·86 to -41·35)	36981·88 (28324·28 to 47454·13)	42062·86 (30601·94 to 57020·64)	111·58 (82·74 to 148·56)	57·34 (42·61 to 77·23)	-48·61 (-58·94 to -35·85)
Typhoid and paratyphoid	0·03 (0·01 to 0·09)	0·03 (0·01 to 0·08)	0 (0 to 0)	0 (0 to 0)	-10·04 (-28·26 to 6·47)	2·12 (0·54 to 5·97)	1·53 (0·41 to 4·03)	0·01 (0 to 0·01)	0 (0 to 0·01)	-10·49 (-31·56 to 9·69)

**Typhoid fever**	0·01 (0 to 0·03)	0·01 (0 to 0·03)	0 (0 to 0)	0 (0 to 0)	-2·57 (-30·45 to 30·76)	0·68 (0·15 to 1·99)	0·54 (0·14 to 1·42)	0 (0 to 0)	0 (0 to 0)	-3·4 (-34·45 to 38·46)
**Paratyphoid fever**	0·02 (0 to 0·06)	0·02 (0·01 to 0·06)	0 (0 to 0)	0 (0 to 0)	-13·44 (-31·3 to 4·26)	1·44 (0·33 to 4·21)	0·99 (0·25 to 2·94)	0 (0 to 0·01)	0 (0 to 0·01)	-13·77 (-34·99 to 8·34)
**Invasive non-typhoidal Salmonella (iNTS)**	8·23 (3·93 to 15·26)	7·4 (3·01 to 14·62)	0·02 (0·01 to 0·04)	0·01 (0·01 to 0·02)	-41·38 (-49·64 to -33·8)	462·97 (215·1 to 862·15)	284·46 (128·01 to 532·47)	1·1 (0·52 to 2·01)	0·65 (0·28 to 1·26)	-40·98 (-50·34 to -31·71)
**Other intestinal infectious diseases**	3·75 (2·1 to 5·46)	1·46 (0·78 to 2·38)	0·01 (0·01 to 0·02)	0 (0 to 0)	-78·11 (-89·06 to -58·67)	243·57 (126·64 to 381·95)	55·1 (38·3 to 75·86)	0·61 (0·3 to 0·97)	0·12 (0·07 to 0·18)	-80·28 (-90·23 to -56·51)
**Neglected tropical diseases and malaria**	30·95 (26·75 to 40·86)	76·9 (14·77 to 112·19)	0·1 (0·08 to 0·13)	0·09 (0·02 to 0·13)	-2·9 (-78·02 to 46·62)	34130·51 (23723·87 to 46054·37)	33899·38 (22833·25 to 47452·75)	83·33 (58·23 to 111·96)	49·28 (33·77 to 68·63)	-40·86 (-52·35 to -27·34)
**Malaria**	13·37 (11·78 to 15·3)	1·7 (1·42 to 2·01)	0·04 (0·03 to 0·04)	0 (0 to 0)	-93·53 (-94·31 to -92·59)	2126·33 (1672·82 to 2690·67)	92·83 (66·48 to 140·32)	4·59 (3·61 to 5·79)	0·18 (0·12 to 0·28)	-96·18 (-97·45 to -94·07)
**Chagas disease**	0 (0 to 0)	0 (0 to 0)	0 (0 to 0)	0 (0 to 0)	NA	0 (0 to 0)	0 (0 to 0)	0 (0 to 0)	0 (0 to 0)	NA
**Leishmaniasis**	0 (0 to 0)	0 (0 to 0)	0 (0 to 0)	0 (0 to 0)	NA	0 (0 to 0)	0 (0 to 0)	0 (0 to 0)	0 (0 to 0)	NA
**Visceral leishmaniasis**	0 (0 to 0)	0 (0 to 0)	0 (0 to 0)	0 (0 to 0)	NA	0 (0 to 0)	0 (0 to 0)	0 (0 to 0)	0 (0 to 0)	NA
**Cutaneous and mucocutaneous leishmaniasis**	NA	NA	NA	NA	NA	0 (0 to 0)	0 (0 to 0)	0 (0 to 0)	0 (0 to 0)	NA
**African trypanosomiasis**	0 (0 to 0)	0 (0 to 0)	0 (0 to 0)	0 (0 to 0)	NA	0 (0 to 0)	0 (0 to 0)	0 (0 to 0)	0 (0 to 0)	NA
**Schistosomiasis**	0 (0 to 0)	0 (0 to 0)	0 (0 to 0)	0 (0 to 0)	NA	0 (0 to 0)	0 (0 to 0)	0 (0 to 0)	0 (0 to 0)	NA
**Cysticercosis**	0·85 (0·47 to 1·33)	0·33 (0·17 to 0·52)	0 (0 to 0)	0 (0 to 0)	-81·05 (-90·56 to -61·7)	5971·01 (2874·87 to 10325·07)	8601·84 (3448·68 to 17409·18)	17·45 (8·76 to 30·44)	10·08 (4·12 to 20·73)	-42·27 (-76·07 to 24·71)
**Cystic echinococcosis**	2·45 (1·37 to 3·62)	0·57 (0·28 to 0·95)	0·01 (0 to 0·01)	0 (0 to 0)	-90·94 (-95·85 to -81·12)	98·69 (57·71 to 142·85)	18·98 (12·64 to 26·32)	0·25 (0·15 to 0·37)	0·03 (0·02 to 0·04)	-89·05 (-93·4 to -80·1)
**Lymphatic filariasis**	NA	NA	NA	NA	NA	0 (0 to 0)	0 (0 to 0)	0 (0 to 0)	0 (0 to 0)	NA
**Onchocerciasis**	NA	NA	NA	NA	NA	0 (0 to 0)	0 (0 to 0)	0 (0 to 0)	0 (0 to 0)	NA

**Trachoma**	NA	NA	NA	NA	NA	0 (0 to 0)	0 (0 to 0)	0 (0 to 0)	0 (0 to 0)	NA
**Dengue**	0·31 (0·01 to 0·55)	0·07 (0·02 to 0·12)	0 (0 to 0)	0 (0 to 0)	-94·19 (-97·11 to 12·85)	7·94 (0·69 to 13·58)	1·71 (0·53 to 2·98)	0·03 (0 to 0·05)	0 (0 to 0)	-91·35 (-95·79 to 4·78)
**Yellow fever**	0 (0 to 0)	0 (0 to 0)	0 (0 to 0)	0 (0 to 0)	NA	0 (0 to 0)	0 (0 to 0)	0 (0 to 0)	0 (0 to 0)	NA
**Rabies**	1·48 (0·03 to 2·19)	0·14 (0·03 to 0·24)	0 (0 to 0·01)	0 (0 to 0)	-96·21 (-97·91 to -46·16)	50·54 (1·24 to 75·92)	3·48 (0·59 to 5·99)	0·13 (0 to 0·2)	0 (0 to 0·01)	-96·8 (-98·19 to -61·65)
**Intestinal nematode infections**	1·4 (0·65 to 2·34)	0·27 (0·11 to 0·48)	0 (0 to 0·01)	0 (0 to 0)	-90·26 (-96 to -76·03)	1608·16 (934·26 to 2656·17)	685·72 (362·76 to 1193·22)	3·05 (1·81 to 4·99)	1·33 (0·7 to 2·28)	-56·45 (-75·3 to -24·61)
**Ascariasis**	1·4 (0·65 to 2·34)	0·27 (0·11 to 0·48)	0 (0 to 0·01)	0 (0 to 0)	-90·26 (-96 to -76·03)	83·94 (29·7 to 162·45)	6·58 (3·28 to 10·39)	0·22 (0·08 to 0·41)	0·01 (0·01 to 0·03)	-93·18 (-97·69 to -76·83)
**Trichuriasis**	NA	NA	NA	NA	NA	0 (0 to 0)	0 (0 to 0)	0 (0 to 0)	0 (0 to 0)	26496·63 (14555·5 to 50190·67)
**Hookworm disease**	NA	NA	NA	NA	NA	1524·22 (847·81 to 2542·75)	679·13 (357·15 to 1184·49)	2·84 (1·57 to 4·72)	1·31 (0·69 to 2·28)	-53·64 (-73·97 to -17·51)
**Food-borne trematodiases**	NA	NA	NA	NA	NA	12424·16 (8330·23 to 17462·58)	19453·03 (12916·88 to 27948·86)	30·83 (20·8 to 43·25)	26·99 (17·99 to 38·54)	-12·47 (-23·37 to -1·3)
**Leprosy**	NA	NA	NA	NA	NA	12·39 (7·51 to 18·98)	16·07 (9·88 to 24·03)	0·03 (0·02 to 0·05)	0·02 (0·01 to 0·03)	-36·89 (-42·54 to -29·27)
**Ebola virus disease**	0 (0 to 0)	0 (0 to 0)	0 (0 to 0)	0 (0 to 0)	NA	0 (0 to 0)	0 (0 to 0)	0 (0 to 0)	0 (0 to 0)	NA
**Zika virus disease**	0 (0 to 0)	0 (0 to 0)	0 (0 to 0)	0 (0 to 0)	NA	0 (0 to 0)	0 (0 to 0)	0 (0 to 0)	0 (0 to 0)	NA
**Guinea worm disease**	NA	NA	NA	NA	NA	0 (0 to 0)	0 (0 to 0)	0 (0 to 0)	0 (0 to 0)	NA
**Other neglected tropical diseases**	11·09 (7·68 to 22·09)	73·83 (11·75 to 109·38)	0·04 (0·03 to 0·07)	0·09 (0·02 to 0·13)	121·82 (-62·91 to 270·81)	11831·29 (7765·07 to 17285·99)	5025·73 (2982 to 7410·55)	26·95 (17·8 to 39·31)	10·66 (6·57 to 16·45)	-60·46 (-70·08 to -48·04)
**Other infectious diseases**	1342·04 (975·06 to 2055·37)	499·28 (359·57 to 574·25)	4·08 (3·01 to 6·18)	0·79 (0·57 to 0·99)	-80·72 (-88·57 to -71·75)	106671·56 (73989·68 to 171749·04)	30525·99 (24475·11 to 37219·15)	281·13 (188·55 to 467·45)	62·61 (49·17 to 80·65)	-77·73 (-87·15 to -65·84)
**Meningitis**	580·93 (531·93 to 629·6)	77·16 (66·98 to 87·63)	1·82 (1·67 to 1·97)	0·11 (0·1 to 0·13)	-93·83 (-94·65 to -92·79)	37503·79 (33279·14 to 41838·33)	5234·13 (4189·62 to 6461·45)	96·46 (85·22 to 108·59)	9·76 (7·89 to 11·99)	-89·88 (-91·86 to -87·4)

**Encephalitis**	70·69 (56·09 to 124·8)	186·76 (120·32 to 220·89)	0·2 (0·16 to 0·35)	0·29 (0·19 to 0·34)	44·9 (-33·19 to 103·79)	4974·53 (3984·17 to 7713·91)	7061·62 (5207·39 to 8129·23)	12·15 (9·75 to 18·96)	14·57 (10·56 to 17·32)	19·89 (-32·73 to 62·66)
**Diphtheria**	3·77 (2·2 to 5·85)	0·26 (0·15 to 0·42)	0·01 (0·01 to 0·01)	0 (0 to 0)	-94·37 (-97·04 to -88·75)	253·89 (139·8 to 418·04)	13·39 (8·08 to 20·85)	0·62 (0·34 to 1·04)	0·03 (0·02 to 0·05)	-94·75 (-97·4 to -89·21)
**Whooping cough**	398·39 (40·99 to 1137·76)	20·8 (2·33 to 60)	1·15 (0·12 to 3·3)	0·09 (0·01 to 0·27)	-91·88 (-99·23 to -12·95)	35060·15 (4260·63 to 98542·55)	1914·06 (340·45 to 5290·68)	101·97 (12·42 to 286·37)	8·73 (1·55 to 24·06)	-91·43 (-98·74 to -21·4)
**Tetanus**	40·68 (28·3 to 53·67)	7·86 (6·39 to 9·8)	0·14 (0·1 to 0·19)	0·01 (0·01 to 0·02)	-89·98 (-92·44 to -84·99)	1955·31 (1480·92 to 2636·56)	304·87 (270·75 to 349·44)	5·64 (4·28 to 7·52)	0·71 (0·63 to 0·82)	-87·39 (-90·77 to -82·65)
**Measles**	3·96 (2·75 to 5·59)	0·12 (0·07 to 0·19)	0·01 (0·01 to 0·02)	0 (0 to 0)	-95·31 (-96·91 to -93·43)	344·7 (241·89 to 483·92)	10·88 (6·48 to 17·26)	0·98 (0·68 to 1·37)	0·05 (0·03 to 0·08)	-95·04 (-96·66 to -93·18)
**Varicella and herpes zoster**	34·04 (24·72 to 46·08)	15·17 (9·7 to 22·87)	0·16 (0·1 to 0·23)	0·02 (0·02 to 0·03)	-84·73 (-91·13 to -72·72)	3064·4 (2154·63 to 4259·67)	3299·24 (2002·49 to 5030·4)	8·54 (6·06 to 11·79)	4·97 (3·17 to 7·38)	-41·85 (-59·14 to -19·71)
**Acute hepatitis**	126·97 (115·92 to 138·69)	27·5 (23·8 to 32·36)	0·34 (0·31 to 0·37)	0·04 (0·03 to 0·04)	-88·95 (-90·75 to -86·18)	8819·77 (7627·25 to 10248·78)	3636·05 (2629·64 to 4915·6)	20·53 (17·77 to 23·73)	6·14 (4·39 to 8·25)	-70·1 (-76·37 to -63·06)
**Acute hepatitis A**	23·7 (15·39 to 36·4)	4·65 (1·3 to 6·62)	0·06 (0·04 to 0·09)	0·01 (0 to 0·01)	-85·4 (-96·55 to -73·88)	2884·48 (2109·26 to 3736·74)	1501·51 (989·28 to 2140·78)	6·23 (4·52 to 8·28)	3·23 (2·15 to 4·6)	-48·11 (-66·79 to -31·44)
**Acute hepatitis B**	77·69 (64·36 to 92·78)	19·86 (16·64 to 23·95)	0·21 (0·17 to 0·25)	0·03 (0·02 to 0·03)	-88·2 (-90·76 to -83·65)	4516·12 (3695·14 to 5587·1)	1922·69 (1386·15 to 2622·24)	10·83 (8·82 to 13·28)	2·52 (1·82 to 3·42)	-76·71 (-82·43 to -69·54)
**Acute hepatitis C**	23·7 (12·17 to 35·46)	2·88 (1·86 to 4·67)	0·07 (0·04 to 0·1)	0 (0 to 0·01)	-94 (-96·6 to -88·91)	1185·51 (546·77 to 1783·39)	116·81 (81·27 to 170·96)	2·86 (1·38 to 4·18)	0·19 (0·13 to 0·27)	-93·48 (-96·14 to -86·54)
**Acute hepatitis E**	1·88 (0·31 to 4·15)	0·11 (0·06 to 0·18)	0·01 (0 to 0·01)	0 (0 to 0)	-94·04 (-97·74 to -76·61)	233·66 (92·72 to 438·25)	95·04 (60·96 to 144·43)	0·61 (0·21 to 1·2)	0·2 (0·13 to 0·29)	-67·92 (-84·86 to -21·89)
**Other unspecified infectious diseases**	82·6 (71·61 to 121·71)	163·66 (78 to 198·99)	0·25 (0·22 to 0·37)	0·22 (0·1 to 0·27)	-13·4 (-67·8 to 15·23)	14695·02 (10656·28 to 19908·45)	9051·75 (6266·77 to 11873·29)	34·24 (25·29 to 45·82)	17·65 (12·22 to 23·79)	-48·44 (-65 to -36·97)
**Maternal and neonatal disorders**	2879·02 (2414·87 to 3573·27)	579·05 (488·08 to 677·23)	8·66 (7·23 to 10·76)	2·79 (2·33 to 3·28)	-67·8 (-75·24 to -59·02)	313324·66 (268272·32 to 375193·35)	122067·65 (103044·99 to 142439·83)	895·65 (762·01 to 1084·77)	396 (342·94 to 456·02)	-55·79 (-64·56 to -46·32)
**Maternal disorders**	146·24 (131·22 to 164·8)	38·28 (31·59 to 45·69)	0·27 (0·24 to 0·3)	0·07 (0·06 to 0·09)	-72·92 (-78·44 to -66·36)	9874·14 (8807·31 to 11109·62)	2830·21 (2356·7 to 3333·46)	17·82 (15·94 to 20·01)	5·49 (4·57 to 6·48)	-69·18 (-74·59 to -62·61)
**Maternal hemorrhage**	34·84 (27·9 to 43·03)	6·47 (4·63 to 8·74)	0·06 (0·05 to 0·08)	0·01 (0·01 to 0·02)	-80·42 (-86·66 to -70·88)	2663·76 (2151·25 to 3252·2)	677·38 (496·1 to 916·82)	4·76 (3·85 to 5·77)	1·32 (0·96 to 1·79)	-72·36 (-79·46 to -62·43)
**Maternal sepsis and other maternal infections**	39·39 (32·71 to 47·34)	0·45 (0·32 to 0·59)	0·07 (0·06 to 0·09)	0 (0 to 0)	-98·9 (-99·23 to -98·48)	2429·34 (2009·64 to 2949·44)	95·72 (48·8 to 167·11)	4·5 (3·75 to 5·4)	0·19 (0·09 to 0·33)	-95·79 (-97·87 to -92·87)

**Maternal hypertensive disorders**	40·07 (34·13 to 47·51)	3·11 (2·34 to 3·99)	0·07 (0·06 to 0·08)	0·01 (0 to 0·01)	-91·37 (-93·81 to -88·23)	2496·35 (2130·96 to 2968·18)	229·97 (176·94 to 297·21)	4·42 (3·78 to 5·25)	0·46 (0·35 to 0·59)	-89·67 (-92·4 to -86·06)
**Maternal obstructed labor and uterine rupture**	1·43 (1 to 2·03)	0·44 (0·29 to 0·63)	0 (0 to 0)	0 (0 to 0)	-66·23 (-79·75 to -45·52)	280·15 (149·02 to 476·37)	147·56 (83·52 to 238·88)	0·49 (0·26 to 0·83)	0·29 (0·16 to 0·47)	-41·02 (-63·24 to -4·36)
**Maternal abortion and miscarriage**	13·05 (10·05 to 16·83)	0·38 (0·28 to 0·53)	0·02 (0·02 to 0·03)	0 (0 to 0)	-97·24 (-98·15 to -95·85)	854·61 (658·03 to 1094·55)	59·98 (39·63 to 88·19)	1·57 (1·22 to 1·99)	0·12 (0·08 to 0·17)	-92·54 (-95·19 to -88·72)
**Ectopic pregnancy**	1·95 (1·54 to 2·43)	0·52 (0·37 to 0·7)	0 (0 to 0)	0 (0 to 0)	-70·89 (-80·3 to -56·6)	136·26 (109·51 to 169·08)	40·09 (30·93 to 51·02)	0·24 (0·2 to 0·3)	0·08 (0·06 to 0·1)	-67·31 (-76·07 to -54·69)
**Indirect maternal deaths**	2·3 (1·79 to 2·92)	2·9 (2·09 to 3·92)	0 (0 to 0·01)	0·01 (0 to 0·01)	41·73 (-5·32 to 114·98)	138·92 (108·45 to 177·33)	165·57 (119·83 to 223·4)	0·25 (0·19 to 0·31)	0·34 (0·24 to 0·46)	36·14 (-9·62 to 107·07)
**Late maternal deaths**	0·89 (0·74 to 1·11)	0·81 (0·57 to 1·13)	0 (0 to 0)	0 (0 to 0)	-6·97 (-33·08 to 24·8)	52·28 (43·12 to 65·21)	44·39 (31·02 to 62·2)	0·1 (0·08 to 0·12)	0·09 (0·06 to 0·12)	-10·36 (-36·42 to 20·62)
**Maternal deaths aggravated by HIV/AIDS**	0 (0 to 0)	0 (0 to 0·01)	0 (0 to 0)	0 (0 to 0)	NA	0·11 (0 to 0·23)	0·21 (0·11 to 0·36)	0 (0 to 0)	0 (0 to 0)	NA
**Other maternal disorders**	12·32 (9·45 to 15·88)	23·19 (18·94 to 27·99)	0·02 (0·02 to 0·03)	0·04 (0·04 to 0·05)	92·33 (39·02 to 164·66)	822·36 (644·48 to 1036·49)	1369·34 (1132·54 to 1639·35)	1·48 (1·16 to 1·85)	2·62 (2·15 to 3·16)	76·83 (30·98 to 134·28)
**Neonatal disorders**	2732·78 (2267·28 to 3412·77)	540·78 (449·05 to 639·22)	8·39 (6·96 to 10·48)	2·71 (2·25 to 3·2)	-67·64 (-75·3 to -58·65)	303450·52 (258878·69 to 364956·74)	119237·44 (100505·79 to 139594·16)	877·83 (744·42 to 1067·16)	390·51 (337·3 to 450·17)	-55·51 (-64·5 to -45·79)
**Neonatal preterm birth**	1655·02 (1352·5 to 2079·98)	285·7 (235·64 to 340·88)	5·08 (4·15 to 6·4)	1·44 (1·18 to 1·71)	-71·76 (-79·36 to -63·67)	186888·33 (159028·43 to 226319·25)	66432·45 (55428·57 to 79220·56)	537·73 (455·15 to 655·52)	212·77 (181·82 to 249·06)	-60·43 (-69·34 to -51·11)
**Neonatal encephalopathy due to birth asphyxia and trauma**	417·71 (314·05 to 537·87)	65·71 (51·2 to 82·22)	1·28 (0·96 to 1·65)	0·33 (0·26 to 0·41)	-74·3 (-81·45 to -63·39)	53478·4 (41294·69 to 68555·86)	31098·84 (24238·47 to 39204·94)	149·25 (115·61 to 189·14)	80·29 (64·56 to 96·93)	-46·2 (-58·94 to -27·88)
**Neonatal sepsis and other neonatal infections**	282·51 (197·8 to 400·3)	76·93 (60·27 to 97·09)	0·87 (0·61 to 1·23)	0·39 (0·3 to 0·49)	-55·47 (-70·38 to -30·44)	26707·27 (18868·94 to 37378·3)	9457·73 (7548·06 to 11998·65)	80·31 (56·81 to 112·87)	39·32 (31·76 to 48·64)	-51·03 (-66·71 to -26·41)
**Hemolytic disease and other neonatal jaundice**	19·13 (12·2 to 28·92)	2·12 (1·6 to 2·86)	0·06 (0·04 to 0·09)	0·01 (0·01 to 0·01)	-82·22 (-89·35 to -69·6)	2641·72 (1965·22 to 3559·18)	1100·31 (854·55 to 1363·37)	7·27 (5·25 to 10·01)	2·74 (2·19 to 3·33)	-62·3 (-73·48 to -47·17)
**Other neonatal disorders**	358·41 (247·67 to 503·95)	110·31 (81·95 to 142·62)	1·1 (0·76 to 1·55)	0·55 (0·41 to 0·72)	-49·62 (-68·62 to -18·98)	33734·81 (23887·59 to 46425·74)	11148·12 (8620·28 to 14090·96)	103·27 (73·04 to 142·18)	55·38 (42·73 to 70·18)	-46·37 (-65·5 to -16·95)
**Nutritional deficiencies**	445·2 (405·27 to 484·76)	317·83 (258·82 to 371·57)	2·79 (2·42 to 3·16)	0·43 (0·34 to 0·51)	-84·64 (-87·12 to -81·94)	231994·56 (155194·42 to 338797·13)	87773·37 (57498·93 to 132055·12)	539·45 (371·03 to 780·42)	170·51 (109·32 to 262·67)	-68·39 (-74·08 to -61·63)
**Protein-energy malnutrition**	282·72 (250·88 to 314·26)	122·09 (95·16 to 151·92)	1·84 (1·56 to 2·09)	0·17 (0·13 to 0·21)	-90·91 (-92·83 to -88·49)	11856·99 (9366·87 to 14981·39)	8355·94 (5742·12 to 11728·75)	38·84 (32·48 to 46·43)	12·84 (8·48 to 18·49)	-66·94 (-75·21 to -56·5)

**Iodine deficiency**	NA	NA	NA	NA	NA	1698·9 (776·08 to 3295·99)	1552·75 (702·57 to 3023·69)	3·67 (1·69 to 6·94)	2·53 (1·14 to 4·96)	-30·93 (-37·95 to -23·19)
**Vitamin A deficiency**	NA	NA	NA	NA	NA	724·83 (382·74 to 1186·98)	77·17 (35·84 to 139·77)	1·86 (0·99 to 3·04)	0·33 (0·16 to 0·6)	-82·19 (-91·32 to -67·82)
**Dietary iron deficiency**	NA	NA	NA	NA	NA	210215·41 (136906·44 to 309356·4)	72418·92 (44958·8 to 111994·89)	472·09 (310·16 to 701·07)	147·1 (90·55 to 236·17)	-68·84 (-75·07 to -61·3)
**Other nutritional deficiencies**	162·48 (137·01 to 196·72)	195·74 (155·11 to 232·03)	0·95 (0·77 to 1·21)	0·26 (0·21 to 0·31)	-72·56 (-79·21 to -65·1)	7498·44 (5860·36 to 9432·48)	5368·6 (4184·75 to 6744·09)	22·98 (18·76 to 28·15)	7·71 (5·88 to 9·97)	-66·46 (-72·37 to -59·45)
**Non-communicable diseases**	185875·6 (180500·25 to 187651·4)	265287·84 (254753·7 to 277949·03)	774·69 (754·46 to 782·58)	319·02 (306·54 to 334·36)	-58·82 (-60·51 to -55·9)	8276562·92 (7416287·03 to 9247593·51)	10101126·11 (8677683·04 to 11675850·37)	24002·98 (21867·8 to 26356·1)	13534·56 (11432·62 to 15827·79)	-43·61 (-47·94 to -39·35)
**Neoplasms**	48341·6 (46611·19 to 51148·3)	104176·1 (94906·84 to 111188·61)	161·69 (154·31 to 175·88)	119·63 (108·57 to 127·75)	-26·01 (-33·06 to -20·74)	1493726·9 (1442330·76 to 1537167·81)	2267782·05 (2108839·56 to 2418417·47)	4264·6 (4118·27 to 4453·66)	2647·66 (2464·65 to 2824)	-37·92 (-42·67 to -33·43)
**Lip and oral cavity cancer**	271·89 (256·7 to 289·88)	796·96 (709·95 to 887·71)	0·95 (0·89 to 1·03)	0·92 (0·81 to 1·02)	-3·78 (-15·21 to 9·42)	7954·64 (7496·34 to 8518·46)	17894·43 (16077·61 to 20024·16)	23·42 (22·12 to 25·03)	20·7 (18·68 to 23·08)	-11·62 (-21·88 to -0·05)
**Nasopharynx cancer**	135·14 (113·76 to 158·46)	199·92 (174 to 226·55)	0·4 (0·34 to 0·47)	0·23 (0·2 to 0·26)	-42·35 (-54·25 to -27·88)	4712·16 (3926·79 to 5535·9)	6114·51 (5289·01 to 7017·06)	12·42 (10·45 to 14·49)	7·34 (6·4 to 8·41)	-40·85 (-53·29 to -24·74)
**Other pharynx cancer**	112·68 (103·13 to 123·02)	496·62 (416·36 to 580·89)	0·37 (0·34 to 0·4)	0·55 (0·46 to 0·64)	47·81 (22·3 to 76·03)	3255·1 (2977·43 to 3531·08)	11898·8 (9965·05 to 13995·22)	9·56 (8·74 to 10·42)	13·05 (10·92 to 15·33)	36·48 (11·58 to 63·43)
**Esophageal cancer**	1643·21 (1215·6 to 1765·12)	2192·61 (1891·03 to 3172·85)	5·49 (4·28 to 5·87)	2·43 (2·09 to 3·52)	-55·79 (-62·79 to -16·26)	46230·18 (31654·34 to 50164·66)	47390·29 (40427·91 to 70394·26)	138·16 (99·48 to 149)	51·74 (44·19 to 76·96)	-62·55 (-69·22 to -17·78)
**Stomach cancer**	15885·12 (15178·95 to 16573·14)	12249·57 (10961·69 to 13503·11)	52·35 (49·97 to 55·05)	14·09 (12·57 to 15·6)	-73·07 (-75·69 to -69·97)	475632·46 (454611·9 to 496240·29)	259057·22 (232822·58 to 285793·32)	1360·58 (1300·37 to 1416·56)	298·7 (270·17 to 328·42)	-78·05 (-80·24 to -75·45)
**Colon and rectum cancer**	3203·84 (3050·76 to 3377·21)	12004·11 (10649·1 to 13219·62)	11·81 (11·16 to 12·63)	13·87 (12·22 to 15·3)	17·48 (5·43 to 30·33)	89317·03 (85257·55 to 93793·69)	246589·57 (222651·43 to 270173·56)	272·25 (258·97 to 286·49)	280·81 (253·86 to 307·51)	3·14 (-7·15 to 13·86)
**Liver cancer**	3412·87 (2831·28 to 4174·37)	14477·37 (12937·32 to 16038·64)	11·04 (9·25 to 13·4)	16·2 (14·47 to 17·94)	46·72 (15·8 to 85·78)	105414·8 (86558·71 to 129054·82)	348014·04 (309267·96 to 389220·2)	297·39 (245·74 to 363·22)	390·81 (348·58 to 435·93)	31·42 (2·75 to 67·88)
**Liver cancer due to hepatitis B**	2220·27 (1770·57 to 2766·48)	7876·21 (6388·91 to 9395·61)	6·65 (5·29 to 8·21)	8·76 (7·19 to 10·42)	31·83 (2·01 to 69·54)	73451·82 (58538·85 to 92186·77)	210261·9 (172834·32 to 249480·14)	197·86 (157·98 to 246·78)	237·05 (196·11 to 279·13)	19·81 (-7·88 to 56·57)
**Liver cancer due to hepatitis C**	487·47 (325·83 to 692·81)	2707·17 (1879·05 to 3573·81)	1·98 (1·35 to 2·75)	3·08 (2·18 to 4·04)	55·71 (19·18 to 101·83)	11715·65 (7555·17 to 16934·62)	50729·3 (34099·26 to 68971·95)	39·3 (26·15 to 55·55)	56 (38·15 to 75·45)	42·51 (8·64 to 84·62)
**Liver cancer due to alcohol use**	439·23 (281·05 to 647·6)	2598·77 (1790·64 to 3640·88)	1·47 (0·95 to 2·18)	2·88 (2 to 4·03)	95·8 (50·05 to 153·95)	12390·78 (7826·73 to 18905·05)	59640·77 (39873·7 to 83711·08)	36·87 (23·55 to 54·84)	65·8 (44·69 to 91·92)	78·48 (36·12 to 133·19)

**Liver cancer due to NASH**	149·09 (98·28 to 214·15)	837·29 (564·98 to 1221·55)	0·56 (0·38 to 0·81)	0·94 (0·64 to 1·37)	67·63 (26·47 to 121·55)	3844·03 (2530·72 to 5543·17)	16526·63 (11292·19 to 24140·6)	12·17 (8·06 to 17·43)	18·39 (12·66 to 26·78)	51·13 (13·9 to 99·33)
**Liver cancer due to other causes**	116·81 (78·47 to 166·09)	457·92 (298·9 to 654)	0·38 (0·25 to 0·55)	0·53 (0·36 to 0·74)	39·5 (5·08 to 83·44)	4012·52 (2820·34 to 5635·12)	10855·45 (7190·52 to 15550·88)	11·19 (7·85 to 15·54)	13·56 (9·56 to 18·57)	21·21 (-8·13 to 57·29)
**Gallbladder and biliary tract cancer**	2664·67 (2194·88 to 3319·82)	5617·51 (4253·36 to 6662·61)	10·09 (8·37 to 13·24)	6·44 (4·91 to 7·67)	-36·16 (-57·83 to -16·2)	67189·43 (54761·15 to 78810·35)	101276·85 (78755·75 to 120276·48)	216·12 (177·87 to 264·04)	113·23 (88·36 to 134·05)	-47·61 (-61·79 to -24·05)
**Pancreatic cancer**	2197·52 (2097·84 to 2321·28)	7303·11 (6519·3 to 8149·8)	7·74 (7·33 to 8·19)	8·25 (7·34 to 9·2)	6·52 (-4·29 to 19·16)	58830·73 (56165·58 to 61921·42)	147639·98 (134529·33 to 162746·35)	181·07 (173·04 to 190·71)	164·37 (149·43 to 181·18)	-9·23 (-17·91 to 0·56)
**Larynx cancer**	702·66 (655·17 to 744·73)	518·21 (450·27 to 606·24)	2·43 (2·24 to 2·57)	0·58 (0·51 to 0·68)	-76·03 (-79·31 to -70·36)	19580·85 (17899·08 to 20983·88)	11661·45 (10104·47 to 13768·13)	60·04 (55·14 to 63·95)	12·94 (11·27 to 15·19)	-78·45 (-81·65 to -70·77)
**Tracheal, bronchus, and lung cancer**	**7050·22 (6752·38 to 7450·47)**	23299·61 (20864·16 to 25565·36)	23·66 (22·63 to 25·31)	26·3 (23·51 to 28·95)	11·15 (-1·11 to 23·89)	196792·68 (188429·92 to 205918·94)	448176·62 (407150·4 to 489987·33)	589·84 (564·52 to 620·52)	498·37 (452·28 to 545·03)	-15·51 (-23·74 to -6·42)
**Malignant skin melanoma**	74·07 (57·45 to 140·52)	245·95 (109·56 to 314·15)	0·25 (0·2 to 0·46)	0·29 (0·13 to 0·37)	15·41 (-62·48 to 78·09)	2507·3 (1921·96 to 4802·1)	6410·25 (2857·98 to 8228·54)	6·79 (5·26 to 12·78)	7·92 (3·75 to 10·12)	16·78 (-61·54 to 81·55)
**Non-melanoma skin cancer**	248·07 (229·28 to 264·43)	444·99 (374·48 to 509·13)	1·28 (1·13 to 1·41)	0·58 (0·47 to 0·67)	-54·81 (-61·03 to -47·26)	5607·45 (5261·45 to 5940·01)	6365·17 (5664·75 to 7084·3)	20·28 (18·8 to 21·64)	7·73 (6·84 to 8·6)	-61·87 (-65·95 to -57·2)
**Non-melanoma skin cancer (squamous-cell carcinoma)**	248·07 (229·28 to 264·43)	444·99 (374·48 to 509·13)	1·28 (1·13 to 1·41)	0·58 (0·47 to 0·67)	-54·81 (-61·03 to -47·26)	5606·86 (5261 to 5939·37)	6362·46 (5663·3 to 7081·57)	20·28 (18·8 to 21·64)	7·73 (6·84 to 8·6)	-61·89 (-65·96 to -57·21)
**Non-melanoma skin cancer (basal-cell carcinoma)**	NA	NA	NA	NA	NA	0·59 (0·27 to 1·09)	2·71 (1·24 to 5·03)	0 (0 to 0)	0 (0 to 0·01)	55·31 (42·84 to 68·64)
**Breast cancer**	1428·61 (1352·81 to 1511·88)	4090·18 (3603·11 to 4577·51)	4·32 (4·09 to 4·58)	4·69 (4·12 to 5·24)	8·46 (-6·62 to 22·79)	51577·61 (48815·44 to 54681·65)	122959·61 (108642·96 to 138699·9)	136·52 (129·1 to 144·82)	143·56 (126·99 to 161·59)	5·16 (-9·85 to 19·94)
**Cervical cancer**	1140·21 (998·06 to 1479·71)	1269·67 (1009·87 to 1586·86)	3·38 (3·04 to 4·84)	1·5 (1·19 to 1·86)	-55·72 (-68·41 to -45·21)	41124·16 (34271·83 to 48091·63)	32673·01 (26790·44 to 41287·02)	106·47 (93·42 to 132·16)	39·78 (32·54 to 49·93)	-62·63 (-69·2 to -52·63)
**Uterine cancer**	474·98 (230·75 to 547·92)	372·58 (289·01 to 463·24)	1·56 (0·81 to 1·8)	0·42 (0·33 to 0·52)	-73·17 (-78·45 to -39·24)	14558·88 (6374·81 to 17012)	9880·21 (6912·95 to 12120·54)	41·91 (19·49 to 48·64)	11·13 (7·83 to 13·63)	-73·43 (-78·75 to -35·07)
**Ovarian cancer**	341·09 (290·82 to 599·54)	1370·18 (822·72 to 1607·55)	1·08 (0·9 to 1·96)	1·57 (0·96 to 1·84)	45·23 (-57·62 to 92·8)	11650·89 (10139·13 to 19198·25)	35587·49 (20987·66 to 41442·11)	31·16 (26·79 to 53·54)	41·62 (25·24 to 48·42)	33·57 (-59·61 to 73·93)
**Prostate cancer**	628·15 (552·06 to 837·39)	2711·75 (2088·8 to 3342·6)	2·74 (2·4 to 3·76)	3·14 (2·41 to 3·83)	14·32 (-30·72 to 37·32)	12993·09 (11308·11 to 17116·47)	48810·12 (38724·09 to 62262·13)	49·26 (43·22 to 65·79)	54·6 (43·28 to 69·17)	10·84 (-31·45 to 29·16)
**Testicular cancer**	15·55 (12·5 to 19·33)	17·65 (13·25 to 23·63)	0·04 (0·03 to 0·05)	0·03 (0·02 to 0·04)	-27·79 (-49·75 to 0·89)	855·65 (677·13 to 1068·82)	865·17 (636·13 to 1130·79)	1·86 (1·5 to 2·29)	1·74 (1·19 to 2·38)	-6·05 (-39·07 to 35·56)

**Kidney cancer**	288·99 (269·76 to 307·94)	1569·01 (1382·67 to 1767·59)	0·99 (0·92 to 1·06)	1·8 (1·59 to 2·02)	81·68 (57·87 to 107·53)	8978·12 (8368·37 to 9568·4)	34638·93 (30648·67 to 39068·81)	26·37 (24·64 to 28·1)	40·85 (36·3 to 45·81)	54·93 (34·49 to 78·96)
**Bladder cancer**	662·34 (619·21 to 728)	1973·69 (1724·32 to 2225·53)	2·87 (2·65 to 3·19)	2·31 (2·01 to 2·61)	-19·51 (-29·03 to -8·09)	14685·14 (13804·09 to 15811·82)	34458 (30426·61 to 38806·15)	53·01 (49·62 to 58·04)	38·91 (34·32 to 43·73)	-26·59 (-35·23 to -16·18)
**Brain and central nervous system cancer**	974·22 (689·2 to 1273·72)	1479·33 (923·78 to 1750·5)	2·74 (1·97 to 3·46)	1·91 (1·21 to 2·25)	-30·23 (-64·48 to -4·92)	43710·22 (31486·48 to 61526·1)	46150·05 (29767·64 to 55389·17)	108·12 (78·56 to 150·66)	71·43 (47·79 to 86·64)	-33·94 (-67·73 to -4·36)
**Thyroid cancer**	140·07 (114·76 to 312·55)	580·35 (425·81 to 678·95)	0·55 (0·44 to 1·24)	0·68 (0·5 to 0·79)	24·49 (-58·66 to 66·34)	3985·9 (3296·46 to 8461·08)	13779·34 (9787·24 to 16466·16)	12·35 (10·19 to 26·87)	16·56 (11·45 to 19·76)	34·13 (-56·91 to 81·17)
**Mesothelioma**	43·85 (35·54 to 59·41)	126·05 (104·19 to 150·43)	0·15 (0·12 to 0·21)	0·14 (0·12 to 0·17)	-1·56 (-33·98 to 30·02)	1336·38 (1075·18 to 1841·91)	2949·45 (2425·68 to 3534·14)	3·8 (3·07 to 5·12)	3·4 (2·77 to 4·03)	-10·39 (-42·66 to 20·56)
**Hodgkin lymphoma**	35·01 (26·34 to 57·99)	59·31 (33·15 to 75·48)	0·1 (0·07 to 0·16)	0·07 (0·04 to 0·09)	-24·67 (-65·88 to 21·06)	1507·21 (1125·3 to 2496·41)	2793·24 (1517·87 to 3833·02)	3·66 (2·75 to 6·02)	3·76 (2·11 to 5·09)	2·8 (-55·18 to 72·43)
**Non-Hodgkin lymphoma**	**729·37 (692·42 to 766·66)**	2325·8 (2087·6 to 2590·9)	2·16 (2·05 to 2·27)	2·74 (2·46 to 3·04)	26·91 (14·1 to 41·64)	29005·23 (27310·16 to 30558·99)	55227·6 (49921·77 to 61000·16)	72·35 (68·61 to 76·25)	69·62 (63·09 to 76·73)	-3·77 (-14·16 to 7·01)
**Multiple myeloma**	309·15 (229·25 to 537·53)	1105·84 (627·21 to 1312·7)	1·1 (0·8 to 1·98)	1·23 (0·7 to 1·46)	12·04 (-59·84 to 73·6)	8151·11 (6135·31 to 13826·17)	24081·85 (13543·38 to 28478)	25·61 (19·09 to 44·05)	26·64 (15·05 to 31·5)	4·03 (-62·29 to 57·66)
**Leukemia**	1750·29 (1479·28 to 2016·37)	2049·08 (1514·31 to 2392·54)	4·6 (4·04 to 5·62)	2·69 (2 to 3·15)	-41·55 (-61·56 to -26·34)	91864·31 (73342·73 to 101836·32)	60045·45 (44776·48 to 71351·7)	213·68 (174·01 to 240·47)	96·63 (72·15 to 116·25)	-54·78 (-67·08 to -37·02)
**Acute lymphoid leukemia**	253·08 (136·99 to 361·06)	224·05 (134·7 to 271·86)	0·58 (0·32 to 0·83)	0·38 (0·25 to 0·48)	-33·7 (-65·94 to 36·29)	17032·24 (8633·5 to 23617·86)	11250·35 (7242·67 to 13980·01)	38·19 (19·12 to 52·54)	23·66 (16·24 to 30·62)	-38·04 (-65·97 to 47·1)
**Chronic lymphoid leukemia**	17·04 (12·96 to 28·76)	59·55 (39·09 to 74·23)	0·06 (0·04 to 0·1)	0·07 (0·05 to 0·08)	17·37 (-51·02 to 72·8)	526·76 (409·27 to 875·49)	1507·16 (1018·75 to 1866·64)	1·49 (1·14 to 2·48)	1·82 (1·26 to 2·23)	22·37 (-46·27 to 73·55)
**Acute myeloid leukemia**	363·47 (281·43 to 460·93)	914·16 (604·23 to 1085·61)	0·9 (0·72 to 1·19)	1·17 (0·78 to 1·39)	29·71 (-26·54 to 68·84)	19626·44 (12374·24 to 25551·36)	26953·59 (17448·06 to 32297·81)	44·64 (30·02 to 58·05)	41·11 (26·61 to 49·69)	-7·9 (-41·29 to 26·51)
**Chronic myeloid leukemia**	171·17 (132·12 to 285·24)	149·25 (97·56 to 185·62)	0·43 (0·34 to 0·72)	0·18 (0·12 to 0·23)	-57·85 (-81·47 to -40·98)	7884·23 (6047·81 to 13192·55)	4376·86 (2988·94 to 5486·77)	17·57 (13·51 to 29·88)	6·07 (4·18 to 7·59)	-65·48 (-83·8 to -51·44)
**Other leukemia**	945·53 (622·61 to 1155·67)	702·07 (519·98 to 907·15)	2·63 (1·91 to 3·3)	0·88 (0·66 to 1·15)	-66·35 (-76·12 to -43·04)	46794·65 (26454·13 to 57623·98)	15957·48 (12322·12 to 21836·75)	111·8 (67·19 to 140·83)	23·97 (18·28 to 33·22)	-78·56 (-85·55 to -53·62)
**Other malignant neoplasms**	1674 (1353·82 to 1801·77)	2489·73 (2194·62 to 2802·59)	5·07 (4·22 to 5·44)	3·13 (2·78 to 3·51)	-38·19 (-46·54 to -18·91)	69939·4 (56147·56 to 75754·22)	65158·47 (57977·27 to 74095·87)	176·41 (143·36 to 190·82)	96·09 (86·07 to 109·81)	-45·53 (-52·84 to -25·01)
**Other neoplasms**	**103·74 (84·51 to 151·3)**	739·37 (483·91 to 896·8)	0·38 (0·3 to 0·57)	0·86 (0·56 to 1·04)	127·25 (26·93 to 216·83)	4778·82 (3822·68 to 6170·19)	19234·87 (13220·27 to 23505·02)	14·14 (11·31 to 18·48)	23·6 (16·38 to 28·87)	66·89 (10·35 to 115·76)

**Myelodysplastic, myeloproliferative, and other haematopoietic neoplasms**	103·74 (84·51 to 151·3)	739·37 (483·91 to 896·8)	0·38 (0·3 to 0·57)	0·86 (0·56 to 1·04)	127·25 (26·93 to 216·83)	4778·82 (3822·68 to 6170·19)	19234·87 (13220·27 to 23505·02)	14·14 (11·31 to 18·48)	23·6 (16·38 to 28·87)	66·89 (10·35 to 115·76)
**Benign and in situ intestinal neoplasms**	NA	NA	NA	NA	NA	0 (0 to 0)	0 (0 to 0)	0 (0 to 0)	0 (0 to 0)	NA
**Benign and in situ cervical and uterine neoplasms**	NA	NA	NA	NA	NA	0 (0 to 0)	0 (0 to 0)	0 (0 to 0)	0 (0 to 0)	NA
**Other benign and in situ neoplasms**	NA	NA	NA	NA	NA	0 (0 to 0)	0 (0 to 0)	0 (0 to 0)	0 (0 to 0)	NA
**Cardiovascular diseases**	85939·38 (77075·95 to 89572·43)	77480·53 (66331·6 to 89708·48)	397·21 (352·55 to 415·94)	95·41 (80·6 to 110·54)	-75·98 (-78·78 to -69·81)	2111130·22 (1891861·73 to 2196708·16)	1409203·41 (1260972·08 to 1609305·45)	7394·08 (6687·51 to 7707·58)	1666·57 (1490·71 to 1897·99)	-77·46 (-79·52 to -71·33)
**Rheumatic heart disease**	313·05 (285·6 to 346·8)	314·93 (257·73 to 378·91)	1·16 (1·06 to 1·3)	0·38 (0·31 to 0·46)	-67·05 (-72·81 to -59·38)	10404·38 (9515·02 to 11457·83)	6653·18 (5667·97 to 7715·16)	29·36 (26·9 to 32·2)	8·2 (6·99 to 9·5)	-72·08 (-75·97 to -67·1)
**Ischemic heart disease**	31168·69 (29040·77 to 32640·67)	28280·7 (23997·74 to 32634·65)	147·6 (132·83 to 156·44)	35·02 (29·52 to 40·61)	-76·27 (-79·21 to -72·04)	728966·43 (673716·55 to 761373·24)	440565·04 (391043·08 to 495969·49)	2535·53 (2376·06 to 2651·77)	517·85 (457·8 to 585·42)	-79·58 (-81·77 to -75·62)
**Stroke**	47727·51 (40868·88 to 50260·42)	36508·93 (31325·6 to 44084·95)	215·98 (185·98 to 229·48)	44·26 (37·51 to 53·5)	-79·51 (-82·17 to -72·22)	1185260·31 (1053397·12 to 1245689·09)	735530·72 (652395·67 to 868110·12)	4192·39 (3654·04 to 4415·06)	860·42 (761·94 to 1011·3)	-79·48 (-81·37 to -72·28)
**Ischemic stroke**	19925·63 (15856·68 to 22258·92)	22578·62 (18897·91 to 27428·88)	104·45 (83·78 to 115·6)	27·83 (23·14 to 33·5)	-73·35 (-77·97 to -62·01)	434660·98 (357771·65 to 483902·11)	413614·7 (360533·95 to 480454·38)	1792·23 (1482·1 to 1980·87)	482·92 (417·58 to 556·91)	-73·05 (-76·57 to -62·92)
**Intracerebral hemorrhage**	23785·43 (21186·04 to 25670·09)	10759·81 (9331·91 to 13438·63)	97·42 (85·58 to 106·21)	12·68 (10·97 to 15·64)	-86·99 (-88·82 to -81·8)	613997·54 (547584·91 to 664854·73)	223518·73 (198961·92 to 283064·13)	2009·28 (1795·19 to 2162·2)	259·68 (232·46 to 330·72)	-87·08 (-88·61 to -81·65)
**Subarachnoid hemorrhage**	4016·45 (2954·51 to 4963·7)	3170·51 (2756·9 to 3797·4)	14·1 (10·15 to 17·62)	3·75 (3·25 to 4·47)	-73·42 (-79·24 to -60·99)	136601·79 (105756·43 to 164850·11)	98397·28 (86957·43 to 115528·76)	390·87 (301·1 to 473·88)	117·81 (104·18 to 137·34)	-69·86 (-75·31 to -58·77)
**Hypertensive heart disease**	**3389·12 (2617·43 to 3743·19)**	5086·33 (3947·37 to 6472·04)	17·62 (14·34 to 19·71)	6·73 (5·16 to 8·37)	-61·8 (-70 to -50·12)	73426·38 (52809·35 to 79977·57)	62698·16 (51238·83 to 93096·13)	277·64 (214·46 to 304·26)	77·44 (62·77 to 111·45)	-72·11 (-77·47 to -51·97)
**Non-rheumatic valvular heart disease**	200·09 (160·54 to 235·07)	836·05 (689·04 to 972·33)	0·89 (0·71 to 1·04)	1·03 (0·85 to 1·21)	16·63 (-9·33 to 53·38)	6276·86 (4987·89 to 7725·27)	17196·67 (13957·85 to 21368·55)	21·46 (17·17 to 26·31)	20·31 (16·59 to 25·1)	-5·38 (-21·86 to 16·53)
**Non-rheumatic calcific aortic valve heart disease**	113·29 (83·63 to 144·27)	664·47 (536·44 to 786·9)	0·52 (0·39 to 0·65)	0·83 (0·67 to 0·98)	58·37 (21·22 to 117·94)	2876·72 (2151·43 to 3615·79)	11145·48 (9362·57 to 13336·84)	10·13 (7·69 to 12·6)	13·25 (11·11 to 15·77)	30·82 (2·93 to 74·69)
**Non-rheumatic degenerative mitral valve heart disease**	76·92 (61·34 to 96·93)	160·25 (111·86 to 203·86)	0·33 (0·26 to 0·42)	0·19 (0·13 to 0·24)	-41·56 (-58·95 to -14·02)	3056·63 (2354·43 to 3997·1)	5824·6 (4145·75 to 8281·32)	10·44 (8·05 to 13·83)	6·75 (4·85 to 9·54)	-35·29 (-48·45 to -20·58)
**Other non-rheumatic valve heart diseases**	**9·88 (6·65 to 13·27)**	11·33 (5·33 to 17·38)	0·04 (0·02 to 0·05)	0·01 (0·01 to 0·02)	-60·81 (-71·81 to -42·91)	343·51 (252·24 to 478·44)	226·59 (141·78 to 302·55)	0·9 (0·64 to 1·18)	0·3 (0·2 to 0·39)	-66·23 (-75·57 to -51·53)

**Cardiomyopathy and myocarditis**	641·51 (470·31 to 934·02)	1129·98 (745·31 to 1347·42)	2·37 (1·61 to 3·1)	1·43 (0·97 to 1·69)	-39·77 (-62·11 to -14·6)	25798·67 (19700·11 to 39953·46)	26537·63 (19499·34 to 31058·72)	71·68 (53·22 to 104·95)	38·3 (29·11 to 44·98)	-46·56 (-68·49 to -25·87)
**Myocarditis**	106·97 (61·85 to 153·62)	74·87 (43·38 to 98·84)	0·38 (0·16 to 0·55)	0·12 (0·07 to 0·15)	-68·49 (-78·87 to -29·31)	5737·65 (3653·59 to 8966·71)	2594·45 (1978·36 to 3476·25)	14·78 (8·28 to 21·11)	5·36 (3·93 to 6·85)	-63·77 (-79·14 to -28·29)
**Alcoholic cardiomyopathy**	29·22 (17·62 to 47·53)	23·08 (14·85 to 31·17)	0·09 (0·06 to 0·15)	0·03 (0·02 to 0·04)	-69·71 (-83·25 to -44·14)	1074·84 (669·01 to 1748·13)	707·44 (499·72 to 920·58)	2·76 (1·7 to 4·48)	0·95 (0·69 to 1·24)	-65·58 (-80·2 to -41·3)
**Other cardiomyopathy**	505·31 (372·49 to 734·07)	1032·03 (682·84 to 1230·96)	1·9 (1·36 to 2·64)	1·28 (0·87 to 1·52)	-32·56 (-58·47 to -3·66)	18986·18 (14032·72 to 29618·75)	23235·73 (16807·7 to 27092·4)	54·13 (40·32 to 80·49)	32 (23·81 to 37·55)	-40·89 (-64·56 to -18·1)
**Atrial fibrillation and flutter**	466·38 (402·63 to 540·92)	2022·03 (1640·38 to 2301·69)	2·93 (2·47 to 3·42)	2·57 (2·05 to 2·94)	-12·38 (-27·51 to 5)	16137·52 (12663·87 to 20214·27)	49455·1 (39534·73 to 61931·65)	65·94 (53·47 to 80·34)	56·82 (45·46 to 70·8)	-13·84 (-23·44 to -4·1)
**Aortic aneurysm**	449·1 (370·52 to 549·98)	1461·09 (1251·44 to 1691·33)	1·92 (1·6 to 2·37)	1·72 (1·47 to 2)	-10·4 (-27·79 to 11·74)	10417·19 (8446·01 to 12647·47)	24014·19 (20926·96 to 27308·06)	35·4 (29·24 to 43·31)	27·85 (24·31 to 31·63)	-21·32 (-37·67 to -0·97)
**Peripheral artery disease**	74·74 (60·35 to 95·01)	205·11 (163·33 to 248·78)	0·38 (0·31 to 0·48)	0·25 (0·2 to 0·31)	-33·83 (-53·06 to -13·27)	3439·54 (2324·2 to 5167·9)	6609·63 (4468·65 to 10076·94)	14·31 (9·75 to 21·34)	7·53 (5·14 to 11·39)	-47·37 (-54·71 to -40·14)
**Endocarditis**	641·64 (279·45 to 864·84)	587·17 (447·61 to 908·33)	2·77 (1·15 to 3·8)	0·73 (0·55 to 1·12)	-73·69 (-83·71 to -22·83)	18175·04 (8544·6 to 23819·67)	10546·43 (8193·06 to 17061·33)	56·69 (25·56 to 75·45)	13·39 (10·35 to 21·74)	-76·39 (-85·39 to -28·98)
**Other cardiovascular and circulatory diseases**	867·56 (691·57 to 968·13)	1048·22 (879·67 to 1328·57)	3·58 (2·96 to 3·97)	1·27 (1·06 to 1·63)	-64·51 (-70·41 to -54·91)	32827·91 (25790·94 to 38423·78)	29396·67 (24523·5 to 36483·25)	93·67 (75·42 to 108·64)	38·47 (31·7 to 48·64)	-58·93 (-64·82 to -48·41)
**Chronic respiratory diseases**	11476·8 (8397·69 to 12517·66)	15506·39 (12625·84 to 17472·24)	62·49 (41·58 to 69·62)	19·16 (15·35 to 21·58)	-69·35 (-73·53 to -55·26)	323117·32 (273813 to 361754·53)	421654·58 (367032·54 to 477126·53)	1203·64 (961·4 to 1332·47)	559·69 (476·76 to 651·98)	-53·5 (-58·54 to -41·77)
**Chronic obstructive pulmonary disease**	4572·22 (3543·17 to 5215·14)	10691·51 (8784·55 to 12326·26)	24·22 (17·02 to 28·29)	13·22 (10·76 to 15·14)	-45·4 (-56·06 to -20·99)	122623·97 (106553·55 to 135351·9)	263783·73 (231185·92 to 295758·05)	479·94 (402·59 to 535·85)	313·85 (275·17 to 351·29)	-34·61 (-42·28 to -19·12)
**Pneumoconiosis**	391·25 (301·11 to 481·92)	426·42 (344·97 to 532·5)	1·29 (0·99 to 1·58)	0·48 (0·39 to 0·59)	-63·26 (-72·62 to -47·39)	10795·66 (8311·33 to 13326·22)	8478·92 (6922·71 to 10565·81)	33·16 (25·78 to 40·57)	9·37 (7·68 to 11·63)	-71·74 (-78·89 to -59·66)
**Silicosis**	53·58 (17·78 to 93·54)	22·76 (8·97 to 109·33)	0·17 (0·06 to 0·3)	0·03 (0·01 to 0·12)	-85·54 (-95·23 to -35·09)	1551·07 (483·76 to 2637·67)	703·36 (384·33 to 2284·77)	4·69 (1·65 to 8·21)	0·78 (0·43 to 2·52)	-83·39 (-92·52 to -37·16)
**Asbestosis**	1·27 (0·56 to 2·29)	7·41 (4·65 to 12·57)	0 (0 to 0·01)	0·01 (0·01 to 0·01)	68·65 (-11·39 to 346·24)	34·1 (18·15 to 61·88)	147·53 (98·68 to 237·68)	0·11 (0·06 to 0·2)	0·17 (0·11 to 0·26)	47·3 (-16·49 to 216·44)
**Coal workers pneumoconiosis**	156·12 (94·4 to 217·28)	333·88 (119·35 to 423·47)	0·51 (0·31 to 0·71)	0·37 (0·14 to 0·47)	-27·11 (-67·54 to 15·6)	4204·09 (2570·95 to 5852·4)	5937·89 (2313·45 to 7498·43)	12·81 (7·74 to 17·86)	6·56 (2·57 to 8·27)	-48·81 (-74·84 to -18·44)
**Other pneumoconiosis**	180·29 (94·85 to 255·3)	62·37 (33·03 to 251·51)	0·6 (0·33 to 0·86)	0·07 (0·04 to 0·28)	-88·51 (-94·61 to -24·36)	5006·4 (2611·31 to 7035·57)	1690·14 (1080·31 to 4918·96)	15·54 (8·4 to 21·9)	1·87 (1·19 to 5·43)	-87·98 (-93·34 to -42·38)

**Asthma**	6133 (3869·57 to 7153·01)	2673·7 (2001·91 to 3282·02)	35·6 (20·81 to 42·09)	3·48 (2·56 to 4·31)	-90·23 (-92·41 to -83·76)	173434·98 (132844·6 to 210583·71)	103986·57 (76945·64 to 139186·65)	645·6 (465·16 to 764·32)	178·52 (126·09 to 251·8)	-72·35 (-79·18 to -60·84)
**Interstitial lung disease and pulmonary sarcoidosis**	283·16 (226·08 to 395·24)	1626·54 (779·03 to 2213·01)	1·11 (0·86 to 1·52)	1·87 (0·9 to 2·53)	68·02 (-19·98 to 156·31)	9210·89 (7458·5 to 13145·89)	37186·07 (23326·4 to 47190·35)	28·95 (23·68 to 39·79)	43·07 (27·9 to 54·21)	48·76 (-14·88 to 104·23)
**Other chronic respiratory diseases**	97·17 (46·65 to 143·86)	88·23 (66·62 to 132·07)	0·26 (0·13 to 0·39)	0·11 (0·08 to 0·16)	-57·78 (-76·14 to -3·15)	7051·82 (4634·57 to 9283·02)	8219·29 (6737·85 to 10021·93)	15·99 (10·4 to 21·16)	14·88 (11·93 to 18·28)	-6·96 (-34·88 to 44·62)
**Digestive diseases**	21168 (20224·33 to 21946·52)	17385·28 (15281·89 to 19344·55)	72·6 (68·84 to 76·4)	20·86 (18·23 to 23·26)	-71·27 (-74·49 to -67·74)	778657·4 (733374·15 to 823404·54)	512621·51 (453081·68 to 582024·13)	2134·01 (2022·88 to 2252·96)	645·95 (565·45 to 738·19)	-69·73 (-72·82 to -66·49)
**Cirrhosis and other chronic liver diseases**	15888·22 (14270·68 to 16507·81)	10355·65 (9156·33 to 11891·08)	47·55 (43·41 to 49·42)	11·94 (10·56 to 13·82)	-74·89 (-77·89 to -68·73)	556869·47 (493079·57 to 581931·79)	279336·99 (249805·49 to 312891·27)	1457·75 (1306·98 to 1519·08)	326·62 (292·31 to 365·16)	-77·59 (-80·18 to -73·18)
**Cirrhosis and other chronic liver diseases due to hepatitis B**	8033·68 (6777·45 to 9285·29)	3692·96 (2897·28 to 4731·08)	23·93 (20·06 to 27·71)	4·3 (3·38 to 5·42)	-82·03 (-84·64 to -77·33)	285003·49 (240041·77 to 330773·91)	101159·63 (79525·03 to 126850·36)	737·91 (623·33 to 852·91)	119·53 (94·25 to 147·89)	-83·8 (-86 to -80·45)
**Cirrhosis and other chronic liver diseases due to hepatitis C**	1231·62 (901·87 to 1661·1)	978·68 (688·9 to 1341·32)	3·71 (2·75 to 5·04)	1·15 (0·83 to 1·56)	-69·08 (-73·83 to -61·34)	43832·4 (31855·08 to 59305·56)	26267·46 (18176·82 to 37040·02)	113·57 (83·31 to 152·58)	31·16 (21·8 to 43·01)	-72·57 (-76·47 to -66·92)
**Cirrhosis and other chronic liver diseases due to alcohol use**	5778·36 (4516·54 to 7050·48)	4825·56 (3812·59 to 5938·66)	17·1 (13·55 to 20·9)	5·46 (4·34 to 6·71)	-68·04 (-72·11 to -60·02)	196776·68 (151961·6 to 240671·96)	132477·94 (104540·45 to 161197·54)	523·5 (408·02 to 635·76)	151·08 (119·44 to 182·81)	-71·14 (-74·81 to -65·17)
**Cirrhosis and other chronic liver diseases due to NAFLD**	301·72 (221·33 to 407·95)	346·39 (231·06 to 491·77)	1·01 (0·74 to 1·35)	0·4 (0·27 to 0·58)	-60·04 (-67·4 to -48·72)	9638·87 (6910·45 to 12990·86)	8140·75 (5343·86 to 12141·31)	26·72 (19·55 to 35·83)	9·45 (6·36 to 13·62)	-64·62 (-71·25 to -55·44)
**Cirrhosis and other chronic liver diseases due to other causes**	542·84 (415·86 to 703·15)	512·06 (352·85 to 719·96)	1·8 (1·33 to 2·39)	0·62 (0·44 to 0·87)	-65·4 (-71·82 to -53·15)	21618·03 (17436·62 to 26867·14)	11291·2 (8401·45 to 14741·84)	56·05 (44·77 to 69·45)	15·4 (12·01 to 19·47)	-72·53 (-77·24 to -65·76)
**Upper digestive system diseases**	2235·9 (2040·93 to 2749·3)	1081·43 (881·59 to 1294·18)	10·79 (9·68 to 12·97)	1·38 (1·11 to 1·68)	-87·18 (-89·52 to -84·46)	79349·56 (65685·23 to 102322)	60460·97 (40064·11 to 93950·75)	249·51 (214·65 to 308·28)	81·86 (53·46 to 127·8)	-67·19 (-76·45 to -56·17)
**Peptic ulcer disease**	1775·08 (1569·07 to 2424·93)	977·48 (800·27 to 1163·01)	8·07 (6·95 to 11·4)	1·24 (1 to 1·49)	-84·63 (-88·97 to -81·37)	43867·43 (39236·8 to 61846·56)	16623·43 (13887·21 to 19387·27)	149·21 (132·59 to 204·47)	20·7 (17·17 to 24·21)	-86·13 (-90·78 to -83·24)
**Gastritis and duodenitis**	460·82 (246 to 591·96)	103·94 (72·8 to 154·25)	2·73 (1·2 to 3·51)	0·14 (0·1 to 0·22)	-94·72 (-96·77 to -75·89)	13254·76 (8977·64 to 16509·6)	5458·45 (3735·82 to 7768·37)	47·89 (27·36 to 58·67)	8·66 (5·83 to 12·44)	-81·93 (-87·66 to -67·39)
**Gastroesophageal reflux disease**	NA	NA	NA	NA	NA	22227·37 (11306·14 to 39775·7)	38379·09 (19211·07 to 69923·32)	52·4 (26·58 to 94·47)	52·51 (26·67 to 94·45)	0·2 (-1·64 to 1·9)
**Appendicitis**	206·57 (181·34 to 260·74)	137·55 (85·55 to 176·73)	0·79 (0·67 to 0·96)	0·18 (0·11 to 0·23)	-77·29 (-87·27 to -69·42)	10488·49 (8878·73 to 12963·35)	4373·71 (3243·43 to 5689·64)	26·14 (22·5 to 32·18)	8·39 (5·92 to 11·49)	-67·9 (-78·73 to -57·29)
**Paralytic ileus and intestinal obstruction**	391·33 (316·94 to 684·7)	1593·79 (882·47 to 1985·92)	1·81 (1·43 to 3·09)	2·03 (1·13 to 2·55)	12·11 (-57·23 to 63·23)	12286·12 (10073·47 to 20304·19)	23925·29 (14209·48 to 29139·92)	40·46 (33·16 to 65·58)	32·07 (19·84 to 38·63)	-20·73 (-66·49 to 9·99)

**Inguinal, femoral, and abdominal hernia**	211·62<br>(131·95 to 246·82)	138·43<br>(98·37 to 251·79)	1·08<br>(0·63 to 1·31)	0·18<br>(0·13 to 0·32)	-83·54<br>(-88·84 to -59·32)	22042·25<br>(16235·6 to 29537·47)	19829·5<br>(13913·08 to 27655·3)	60·69<br>(45·38 to 79·97)	36·45<br>(24·91 to 51·1)	-39·94<br>(-49·95 to -28·66)
**Inflammatory bowel disease**	313·12<br>(143·27 to 399·24)	233·74<br>(182·56 to 313·27)	1·76<br>(0·56 to 2·34)	0·3<br>(0·23 to 0·39)	-83·15<br>(-88·16 to -49·32)	9497·96<br>(6903·37 to 11453·94)	10451·4<br>(7828·58 to 13342·91)	31·72<br>(18·3 to 38·85)	15<br>(11·13 to 19·33)	-52·71<br>(-66·41 to -17·79)
**Vascular intestinal disorders**	134·88<br>(104·03 to 184·33)	631·58<br>(480·5 to 754·15)	0·61<br>(0·47 to 0·82)	0·76<br>(0·58 to 0·91)	25·4<br>(-22·3 to 75·5)	3316·11<br>(2549·16 to 4666·14)	10298·28<br>(7890·75 to 12097·4)	11·49<br>(8·95 to 15·78)	12·22<br>(9·43 to 14·41)	6·37<br>(-35·97 to 48·61)
**Gallbladder and biliary diseases**	1057·68<br>(901·61 to 1330·08)	2034·1<br>(1466·34 to 2436·49)	5·27<br>(4·52 to 7·07)	2·62<br>(1·88 to 3·18)	-50·29<br>(-68·3 to -37·56)	59363·81<br>(44799·59 to 78307·82)	78129·04<br>(57252·11 to 105631·5)	182·84<br>(143·39 to 231·99)	99·81<br>(73 to 135·74)	-45·41<br>(-54·03 to -37·76)
**Pancreatitis**	502·8<br>(347·56 to 734·23)	562·14<br>(414·78 to 693·84)	1·73<br>(1·2 to 2·29)	0·69<br>(0·51 to 0·84)	-60·29<br>(-75·57 to -32·05)	17628·97<br>(11957·68 to 27207·73)	14067·87<br>(10802·17 to 18126·85)	47·3<br>(33 to 68·87)	17·68<br>(13·77 to 22·69)	-62·62<br>(-79·88 to -33·56)
**Other digestive diseases**	225·87<br>(195·26 to 306·76)	616·89<br>(459·81 to 755·72)	1·21<br>(1·01 to 1·49)	0·78<br>(0·58 to 0·96)	-35·43<br>(-57·7 to -15·37)	7814·66<br>(6573·91 to 10634·71)	11748·45<br>(9219·83 to 14080·73)	26·11<br>(22·47 to 33·63)	15·85<br>(12·71 to 19)	-39·28<br>(-60·1 to -26·65)
**Neurological disorders**	5082·34<br>(2486·19 to 10736·67)	23622·93<br>(10130·01 to 51640·35)	31·94<br>(12·44 to 73·79)	30·2<br>(12·54 to 66·09)	-5·45<br>(-18·35 to 7·98)	423218·86<br>(210156·01 to 726838·86)	726908·02<br>(424600·22 to 1176642·37)	1177·48<br>(665·99 to 1940·32)	1052·53<br>(594·57 to 1718·93)	-10·61<br>(-21·45 to 0·46)
**Alzheimer's disease and other dementias**	3364·03<br>(804·76 to 9038·49)	18450·93<br>(4756·66 to 47101·55)	25·72<br>(6·11 to 68·11)	23·94<br>(6·16 to 60·27)	-6·92<br>(-16·48 to 6·86)	62632·75<br>(27705·22 to 138968·99)	286944·39<br>(131406·37 to 603047·17)	368·87<br>(162·4 to 805·71)	346·02<br>(157·4 to 724·35)	-6·2<br>(-14·35 to 2·12)
**Parkinson's disease**	696·86<br>(619·82 to 1183·72)	3730·07<br>(3020·93 to 4127·61)	3·6<br>(3·15 to 6·27)	4·34<br>(3·54 to 4·81)	20·68<br>(-40·28 to 41·03)	13826·5<br>(12334·59 to 21447·66)	61712·53<br>(50254·67 to 68374·7)	59·04<br>(52·33 to 94·43)	69·49<br>(56·77 to 76·91)	17·69<br>(-37·33 to 34·77)
**Idiopathic epilepsy**	597·55<br>(543·24 to 841·76)	504·27<br>(389·96 to 575·73)	1·43<br>(1·31 to 2·03)	0·75<br>(0·6 to 0·86)	-47·94<br>(-69 to -36·62)	71035·61<br>(40987·65 to 113075·87)	47763·58<br>(23686·57 to 86697·22)	157·73<br>(89·45 to 253·85)	91·38<br>(44·51 to 167·57)	-42·07<br>(-73·1 to 22·8)
**Multiple sclerosis**	43·91<br>(37·09 to 65·39)	74·85<br>(60·49 to 109·53)	0·12<br>(0·1 to 0·18)	0·09<br>(0·07 to 0·13)	-24·99<br>(-55·39 to -0·74)	2662·51<br>(2183·05 to 3587·78)	4226·35<br>(3403·43 to 5398·57)	6·47<br>(5·34 to 8·67)	5·46<br>(4·38 to 6·93)	-15·69<br>(-39·92 to 1·05)
**Motor neuron disease**	136·64<br>(118·21 to 159·74)	78·5<br>(67·9 to 90·66)	0·41<br>(0·35 to 0·48)	0·09<br>(0·08 to 0·1)	-78·53<br>(-82·81 to -73·74)	8291·93<br>(6729 to 10276·89)	2842·12<br>(2481·9 to 3229·91)	23·89<br>(19·1 to 29·91)	3·53<br>(3·08 to 3·97)	-85·22<br>(-88·63 to -81·2)
**Headache disorders**	NA	NA	NA	NA	NA	245228·05<br>(52814·39 to 528157·36)	292109·2<br>(80775·33 to 602652·7)	515·11<br>(118·82 to 1106·26)	483·65<br>(117·89 to 1028·07)	-6·11<br>(-12·56 to 2·7)
**Migraine**	NA	NA	NA	NA	NA	217755·91<br>(34070·08 to 501098·76)	251195·75<br>(54577·36 to 561351·44)	454·78<br>(77·09 to 1031·19)	420·67<br>(80·12 to 942·23)	-7·5<br>(-13·79 to 1·36)
**Tension-type headache**	NA	NA	NA	NA	NA	27472·14<br>(7262·52 to 100475·95)	40913·45<br>(11577·83 to 136214·86)	60·33<br>(16·51 to 213·97)	62·98<br>(16·51 to 228·37)	4·39<br>(-9·83 to 16·18)
**Other neurological disorders**	243·35<br>(210·19 to 282·94)	784·31<br>(705·37 to 871·53)	0·67<br>(0·58 to 0·77)	1<br>(0·9 to 1·1)	50·45<br>(24·65 to 79·42)	19541·51<br>(13712·72 to 27248·31)	31309·85<br>(26035·77 to 38978·22)	46·35<br>(33·28 to 63·54)	53·01<br>(40·89 to 71·13)	14·36<br>(-21·32 to 71·1)

**Mental disorders**	1·05 (0·6 to 1·76)	0·86 (0·6 to 1·2)	0 (0 to 0)	0 (0 to 0)	-14·79 (-56·97 to 58·19)	600087·5 (439697·28 to 793373·32)	794605·85 (582721·13 to 1039026·48)	1291·43 (952·2 to 1703·11)	1334·59 (975·74 to 1758·55)	3·34 (0·29 to 6·69)
**Schizophrenia**	NA	NA	NA	NA	NA	86547·3 (59487·88 to 117170·95)	134587·19 (93721·39 to 176673·4)	187·93 (132·25 to 252·13)	194·88 (134·03 to 259·99)	3·7 (-4·43 to 13·39)
**Depressive disorders**	NA	NA	NA	NA	NA	151184·35 (102870·4 to 208817·72)	227039·43 (158456·33 to 308654·02)	329·09 (227·69 to 453·28)	357·64 (248·35 to 491·66)	8·67 (1·62 to 15·56)
**Major depressive disorder**	NA	NA	NA	NA	NA	119944·26 (80451·01 to 168315·68)	183103·41 (127196·25 to 250324·48)	261·33 (177·67 to 362·34)	289·88 (198·35 to 400·17)	10·93 (2·26 to 19·91)
**Dysthymia**	NA	NA	NA	NA	NA	31240·09 (18902·03 to 48546·36)	43936·02 (27741·08 to 66979·25)	67·76 (41·82 to 104·81)	67·75 (41·18 to 104·61)	-0·01 (-5·06 to 5·17)
**Bipolar disorder**	NA	NA	NA	NA	NA	49682·91 (28754·16 to 78551·1)	69048·24 (41586·26 to 108121·8)	105·86 (63·16 to 168·72)	109·01 (64·81 to 170·97)	2·97 (-4·91 to 12·1)
**Anxiety disorders**	NA	NA	NA	NA	NA	144988·32 (96025·38 to 206636·09)	177414·5 (118498·36 to 251197·77)	310·72 (206·99 to 440·65)	310·93 (205·17 to 439·93)	0·07 (-6·88 to 7·87)
**Eating disorders**	1·05 (0·6 to 1·76)	0·86 (0·6 to 1·2)	0 (0 to 0)	0 (0 to 0)	-14·79 (-56·97 to 58·19)	27309·22 (16991·74 to 40909·87)	35617·86 (22436·76 to 52753·92)	50·7 (31·62 to 75·7)	75·32 (47·19 to 111·8)	48·56 (34·37 to 64·58)
**Anorexia nervosa**	0·97 (0·55 to 1·65)	0·74 (0·51 to 1·03)	0 (0 to 0)	0 (0 to 0)	-20·12 (-61·43 to 49·56)	8962·8 (5373·72 to 14073·48)	10563·72 (6419·12 to 16609·17)	16·65 (9·95 to 25·9)	23·49 (14·12 to 37·59)	41·12 (17·06 to 69·07)
**Bulimia nervosa**	0·07 (0·03 to 0·16)	0·12 (0·05 to 0·22)	0 (0 to 0)	0 (0 to 0)	54·33 (-45·05 to 285·91)	18346·42 (10439·57 to 28931·55)	25054·14 (14448·74 to 38756·5)	34·05 (19·41 to 53·55)	51·83 (30·21 to 79·78)	52·19 (34·34 to 71·39)
**Autism spectrum disorders**	NA	NA	NA	NA	NA	37424·39 (24234·62 to 54071·88)	41017·25 (26331·87 to 59285·83)	82·55 (53·6 to 119·57)	84·14 (53·82 to 121·94)	1·93 (-3·01 to 7·55)
**Attention-deficit/hyperactivity disorder**	NA	NA	NA	NA	NA	8595·71 (4748·07 to 14802·84)	6359·01 (3561·72 to 10762·86)	17·66 (9·83 to 30·27)	16·77 (9·35 to 28·79)	-5·02 (-20·99 to 11·66)
**Conduct disorder**	NA	NA	NA	NA	NA	30729·94 (17075·29 to 48916·1)	16943·03 (9174·14 to 27190·54)	64·39 (35·47 to 102·82)	61·34 (33·04 to 98·35)	-4·73 (-14·81 to 6·43)
**Idiopathic developmental intellectual disability**	NA	NA	NA	NA	NA	11104·65 (4204·85 to 19334·95)	2229·63 (453·57 to 5006·75)	24·31 (9·3 to 42·33)	4·09 (0·57 to 9·71)	-83·18 (-93·87 to -75·26)
**Other mental disorders**	NA	NA	NA	NA	NA	52520·72 (32890·81 to 79591·15)	84349·7 (53187·63 to 126636·84)	118·23 (74·34 to 177·74)	120·48 (75·81 to 181·46)	1·9 (-3·08 to 7·16)
**Substance use disorders**	1153·03 (995·03 to 1321·19)	1115·79 (921·25 to 1346·09)	2·91 (2·58 to 3·37)	1·32 (1·12 to 1·61)	-54·63 (-67·02 to -24·68)	239100·68 (178046·42 to 310747·28)	249910·58 (185046·19 to 332655·08)	508·34 (382·51 to 654·1)	384·42 (280·31 to 512·32)	-24·38 (-33·36 to -12·96)

**Alcohol use disorders**	**1071·46 (912·9 to 1238·78)**	996·57 (803·22 to 1235·97)	2·69 (2·35 to 3·14)	1·16 (0·96 to 1·44)	-56·93 (-69·69 to -24·69)	188704·27 (137681·69 to 248973·67)	189798·06 (136786·33 to 256521)	410·79 (302·45 to 535·99)	273·67 (193·91 to 373·91)	-33·38 (-43·46 to -20·12)
**Drug use disorders**	81·57 (74·66 to 88·82)	119·22 (106·84 to 132·02)	0·22 (0·2 to 0·24)	0·16 (0·15 to 0·18)	-26·54 (-35·77 to -15·7)	50396·41 (34862·09 to 69429·62)	60112·52 (42723·3 to 80856·53)	97·55 (68·06 to 132·63)	110·75 (77·33 to 149·11)	13·53 (0·73 to 27·22)
**Opioid use disorders**	60·41 (54·46 to 66·22)	68·83 (61·07 to 77·16)	0·16 (0·15 to 0·18)	0·1 (0·09 to 0·11)	-39·46 (-47·52 to -29·49)	11407·72 (8018·35 to 16062·09)	20993·2 (14184·04 to 29143·01)	23·53 (16·9 to 32·25)	36·13 (23·76 to 52·08)	53·54 (26·6 to 83·62)
**Cocaine use disorders**	7·67 (6·26 to 10·14)	9·77 (8·02 to 11·89)	0·02 (0·02 to 0·03)	0·01 (0·01 to 0·01)	-37·24 (-57·09 to -14·8)	10147·61 (5752·82 to 15914·65)	10877·22 (6448·43 to 16853·61)	19·94 (11·57 to 31·01)	19·78 (11·49 to 31·2)	-0·78 (-18·17 to 19·6)
**Amphetamine use disorders**	3·91 (3·22 to 4·87)	4·82 (4·03 to 5·85)	0·01 (0·01 to 0·01)	0·01 (0·01 to 0·01)	-36·26 (-53·58 to -14·9)	8375·08 (4456·18 to 14032·36)	7520·81 (4091·73 to 12571·16)	15·27 (8·32 to 25·49)	15·12 (8·11 to 25·54)	-1·01 (-22·12 to 22·48)
**Cannabis use disorders**	NA	NA	NA	NA	NA	7198·45 (3853·2 to 12250·28)	6373·94 (3527·62 to 10363·27)	13·33 (7·26 to 22·7)	13·43 (7·18 to 22·23)	0·71 (-11·24 to 15·32)
**Other drug use disorders**	9·59 (7·86 to 11·15)	35·81 (31·26 to 40·9)	0·03 (0·03 to 0·03)	0·05 (0·04 to 0·05)	53·25 (25·12 to 95·74)	13267·54 (8157·67 to 19953·85)	14347·35 (9173·32 to 20837·66)	25·47 (15·99 to 37·92)	26·29 (16·27 to 38·58)	3·21 (-19·91 to 33·71)
**Diabetes and kidney diseases**	8251·58 (7871·87 to 9501·87)	20855·39 (18321·07 to 23057·54)	30·42 (28·57 to 37·57)	24·6 (21·39 to 27·26)	-19·14 (-37·22 to -9·49)	347964·43 (309237·75 to 393748·33)	720185·16 (590979·4 to 860975·11)	1042·54 (926·89 to 1186·17)	835·27 (687·74 to 999·11)	-19·88 (-28·72 to -12·77)
**Diabetes mellitus**	5119·42 (4844·8 to 6206·93)	12861·93 (11228·51 to 14493·12)	18·3 (17·14 to 24·87)	15·02 (13 to 16·98)	-17·92 (-43·96 to -6·01)	235528·53 (202634·92 to 275455·15)	548993·69 (429289·36 to 683320·29)	709·26 (610·83 to 840·39)	631·95 (493·45 to 786·57)	-10·9 (-22·24 to -2·53)
**Diabetes mellitus type 1**	458·5 (361·71 to 515·02)	249·58 (212·24 to 319·36)	1·3 (0·99 to 1·48)	0·32 (0·27 to 0·4)	-75·51 (-80·24 to -63·31)	28103·86 (23259·33 to 33836·02)	23649·25 (17495·23 to 31617·08)	66·54 (54·8 to 80·15)	35·38 (26·04 to 47·52)	-46·82 (-57·48 to -33·93)
**Diabetes mellitus type 2**	4660·91 (4404·49 to 5759·56)	12612·35 (10966·47 to 14249·55)	17 (15·87 to 23·74)	14·7 (12·7 to 16·64)	-13·51 (-42·64 to -1·41)	207424·66 (176501·08 to 243950·87)	525344·44 (410939·74 to 652314·33)	642·72 (548·81 to 768·98)	596·57 (466·52 to 740·62)	-7·18 (-19·05 to 1·87)
**Chronic kidney disease**	3122·29 (2974·75 to 3328·5)	7979·04 (6930·95 to 8863·81)	12·08 (11·28 to 12·97)	9·56 (8·27 to 10·64)	-20·85 (-30·1 to -12·15)	112151·76 (105193·68 to 120648·22)	170934·37 (154033·43 to 188914·6)	332·38 (311·39 to 358·81)	202·96 (182·77 to 224·5)	-38·94 (-43·8 to -33·67)
**Chronic kidney disease due to diabetes mellitus type 1**	337·53 (233·98 to 452·13)	398·7 (261·84 to 586·09)	0·9 (0·61 to 1·24)	0·46 (0·31 to 0·66)	-48·65 (-56·29 to -38·59)	14206·45 (10241·1 to 18617·66)	13546·76 (8907·95 to 18648·58)	34·29 (24·06 to 45·4)	16·58 (11·51 to 22·3)	-51·64 (-57·48 to -44·82)
**Chronic kidney disease due to diabetes mellitus type 2**	1209·13 (1038·69 to 1377·05)	3833·41 (3194·01 to 4476·31)	5 (4·28 to 5·75)	4·5 (3·73 to 5·26)	-9·96 (-22·22 to 2·25)	32847·46 (27693·16 to 37322·64)	74259·31 (64329·5 to 84422·72)	109·79 (94·08 to 123·8)	83·44 (72·36 to 94·89)	-24 (-32·15 to -15·18)
**Chronic kidney disease due to hypertension**	744·68 (627·32 to 901·53)	2592·64 (2101·75 to 3160·38)	3·47 (2·88 to 4·15)	3·16 (2·54 to 3·86)	-8·98 (-24·73 to 6·65)	20383·36 (17385·26 to 24759·76)	44897·98 (38039·26 to 52689·88)	70·25 (60·94 to 83·04)	52·35 (44·5 to 61·24)	-25·48 (-36·49 to -14·08)
**Chronic kidney disease due to glomerulonephritis**	516·96 (459·97 to 582·1)	367·77 (274·43 to 498·37)	1·44 (1·24 to 1·67)	0·44 (0·34 to 0·6)	-69·15 (-75·58 to -60·2)	25014·18 (22637·46 to 27570·3)	11190·6 (9172·05 to 13597·23)	58·6 (52·74 to 65·08)	15·16 (12·4 to 18·35)	-74·13 (-77·81 to -69·94)

Chronic kidney disease due to other and unspecified causes	313·99 (258·58 to 379·16)	786·52 (537·22 to 1078·85)	1·28 (0·96 to 1·65)	1 (0·68 to 1·38)	-21·76 (-35·47 to -7·03)	19700·31 (16700·99 to 23369·99)	27039·73 (21812·66 to 33619·31)	59·45 (49·67 to 71·18)	35·43 (28·86 to 43·6)	-40·41 (-45·98 to -33·96)
Acute glomerulonephritis	9·87 (7·15 to 12·82)	14·42 (6·94 to 19·4)	0·04 (0·03 to 0·06)	0·02 (0·01 to 0·02)	-59·86 (-80·04 to -37·59)	284·14 (210·32 to 358·04)	257·1 (129·5 to 320·73)	0·9 (0·67 to 1·14)	0·36 (0·21 to 0·44)	-60·28 (-76·88 to -43·46)
Skin and subcutaneous diseases	**216·87 (167·96 to 264·7)**	654·96 (488·58 to 757·27)	1·19 (0·9 to 1·48)	0·83 (0·61 to 0·96)	-30·4 (-50·65 to -5·58)	255588·16 (166651·87 to 372273·93)	285995·98 (187872·88 to 422346·72)	590·53 (384·93 to 862·76)	605·78 (393·27 to 893·47)	2·58 (-0·15 to 5·45)
Dermatitis	NA	NA	NA	NA	NA	79966·62 (43533·83 to 131458·92)	73599·61 (41336·54 to 120475·91)	189·28 (103·02 to 311·73)	190·99 (105·72 to 317·69)	0·9 (-4 to 6·43)
Atopic dermatitis	NA	NA	NA	NA	NA	74653·18 (39602 to 124552·31)	65736·01 (35567·77 to 111405·98)	177·09 (93·71 to 295·72)	178·79 (96·7 to 302·53)	0·96 (-4·06 to 6·62)
Contact dermatitis	NA	NA	NA	NA	NA	3567·82 (2225·04 to 5407·59)	5310·58 (3250·58 to 8065·91)	8·13 (5·09 to 12·24)	8·11 (5·03 to 12·14)	-0·24 (-11·44 to 12·51)
Seborrhoeic dermatitis	NA	NA	NA	NA	NA	1745·61 (1000·85 to 2770·66)	2553·01 (1444·31 to 4048·55)	4·07 (2·34 to 6·44)	4·09 (2·32 to 6·51)	0·48 (-7·89 to 9·38)
Psoriasis	NA	NA	NA	NA	NA	11349·09 (7969·66 to 15272·84)	14825·28 (10333·07 to 19862·45)	26·06 (18·42 to 34·99)	22·74 (15·84 to 30·77)	-12·75 (-22·77 to -1·29)
Bacterial skin diseases	49·84 (33·5 to 73·45)	159·98 (111·73 to 208·07)	0·22 (0·13 to 0·33)	0·19 (0·14 to 0·25)	-11·05 (-42·4 to 35·46)	4061·33 (2739·69 to 6224·25)	5812·89 (4312·65 to 8045·99)	10·58 (7·39 to 15·92)	9·89 (6·79 to 14·77)	-6·55 (-22·99 to 10·26)
Cellulitis	24·33 (13·17 to 37·13)	74·69 (38·05 to 99·57)	0·11 (0·05 to 0·17)	0·09 (0·05 to 0·12)	-14·81 (-47·47 to 41·32)	1671·78 (1194·49 to 2245·8)	2624·01 (1875·55 to 3362·67)	4·49 (3·24 to 6·06)	4·07 (2·92 to 5·35)	-9·25 (-30·03 to 16·77)
Pyoderma	25·5 (15·2 to 42·41)	85·28 (58·14 to 131·1)	0·11 (0·06 to 0·19)	0·1 (0·07 to 0·16)	-7·54 (-43·37 to 65·16)	2389·55 (1350·58 to 4349·67)	3188·88 (2042·71 to 5159·23)	6·09 (3·58 to 10·62)	5·81 (3·4 to 10·26)	-4·56 (-23·16 to 13·97)
Scabies	NA	NA	NA	NA	NA	1060·29 (572·29 to 1729·42)	1050·07 (587·46 to 1674·11)	2·29 (1·25 to 3·7)	2·33 (1·29 to 3·72)	1·85 (-10·99 to 16·1)
Fungal skin diseases	NA	NA	NA	NA	NA	11906·06 (4788·66 to 25485·37)	20387·62 (8288·24 to 44434·55)	30·96 (12·52 to 67·2)	30·48 (12·24 to 65·9)	-1·56 (-3·22 to -0·05)
Viral skin diseases	NA	NA	NA	NA	NA	52174·02 (33571·28 to 78074·85)	50950·67 (32920·15 to 74988·72)	117·64 (75·73 to 176·13)	117·8 (75·42 to 176·2)	0·13 (-2·53 to 2·82)
Acne vulgaris	NA	NA	NA	NA	NA	43487·72 (25912·8 to 69335·16)	39930·36 (23751·87 to 62766·92)	84·28 (50·18 to 134·28)	102·67 (60·79 to 162·86)	21·82 (13·58 to 30·91)
Alopecia areata	NA	NA	NA	NA	NA	4416·18 (2774·47 to 6569·25)	5551·74 (3585·22 to 8193·82)	9·41 (5·89 to 14·06)	9·28 (5·92 to 13·83)	-1·34 (-8·79 to 7·16)

**Pruritus**	NA	NA	NA	NA	NA	2992·98 (1408·84 to 5492·77)	4861·16 (2302·78 to 8714·32)	7·1 (3·37 to 12·81)	7·6 (3·56 to 13·49)	7·16 (1·36 to 13·14)
**Urticaria**	NA	NA	NA	NA	NA	19898·38 (13023·53 to 28937·45)	20866·7 (13567·55 to 29611·25)	45·68 (30·06 to 66·66)	45·39 (29·23 to 65·77)	-0·62 (-6·99 to 5·79)
**Decubitus ulcer**	156·76 (119·3 to 199·73)	433·24 (312·51 to 523·16)	0·93 (0·69 to 1·17)	0·56 (0·4 to 0·67)	-39·89 (-57·74 to -17·13)	3503·57 (2818·9 to 4332·09)	7181·11 (5795·49 to 8438·57)	14·36 (11·45 to 17·68)	8·86 (7·16 to 10·39)	-38·33 (-53·8 to -19·89)
**Other skin and subcutaneous diseases**	10·28 (6·85 to 14·65)	61·74 (36·02 to 85·18)	0·04 (0·02 to 0·06)	0·07 (0·04 to 0·1)	80·65 (2·9 to 175·94)	20771·92 (10161·87 to 37756·58)	40978·77 (19977·82 to 73225·05)	52·89 (26·11 to 95·66)	57·75 (28·04 to 104·14)	9·2 (6·33 to 11·91)
**Sense organ diseases**	NA	NA	NA	NA	NA	181740·56 (124735·18 to 255622·12)	398119·24 (271308·36 to 566719·49)	553·6 (386·76 to 774·12)	500·81 (341·93 to 705·71)	-9·54 (-13·32 to -5·95)
**Blindness and vision loss**	NA	NA	NA	NA	NA	44227·67 (30718·07 to 61529·31)	75097·55 (51936·84 to 104345·73)	129·08 (90·8 to 176·5)	104·36 (71·75 to 145·46)	-19·15 (-24·17 to -14·21)
**Glaucoma**	NA	NA	NA	NA	NA	2386·25 (1569·09 to 3464·73)	5197·8 (3510·65 to 7245·18)	10·94 (7·37 to 15·72)	6 (4·08 to 8·33)	-45·21 (-50·95 to -38·19)
**Cataract**	NA	NA	NA	NA	NA	6733·57 (4666·68 to 9355·65)	17409·29 (12073·35 to 24180·84)	26·1 (17·94 to 35·64)	20·34 (14·11 to 28·27)	-22·06 (-29·03 to -14·61)
**Age-related macular degeneration**	NA	NA	NA	NA	NA	769·37 (479·95 to 1126·17)	1815·85 (1193·33 to 2609·23)	3·4 (2·22 to 4·92)	2·08 (1·38 to 2·99)	-38·72 (-47·43 to -27·57)
**Refraction disorders**	NA	NA	NA	NA	NA	22102·18 (14400·39 to 32459·67)	31347·76 (20645·98 to 45076·78)	53·89 (35·33 to 78·06)	51·15 (33·36 to 75·17)	-5·09 (-9·56 to -0·42)
**Near vision loss**	NA	NA	NA	NA	NA	1671·39 (749·02 to 3475·27)	4638·61 (2009·83 to 9321·65)	5·71 (2·56 to 11·51)	5·27 (2·3 to 10·59)	-7·61 (-20·66 to 2·92)
**Other vision loss**	NA	NA	NA	NA	NA	10564·91 (7144·22 to 14761·9)	14688·24 (10085·63 to 20383·09)	29·03 (20·07 to 39·88)	19·51 (13·35 to 27·01)	-32·79 (-39·67 to -25·28)
**Age-related and other hearing loss**	NA	NA	NA	NA	NA	120775·52 (81641·52 to 172714·31)	285944·23 (191872·23 to 415199·51)	377·95 (259·86 to 537·11)	347·74 (234·73 to 503·09)	-7·99 (-12·59 to -3·75)
**Other sense organ diseases**	NA	NA	NA	NA	NA	16737·37 (9890·56 to 25746·75)	37077·47 (21890·76 to 58037·92)	46·57 (27·78 to 71·49)	48·71 (28·78 to 76·08)	4·58 (-0·13 to 9·96)
**Musculoskeletal disorders**	449·12 (387·88 to 573·18)	1255·66 (792·82 to 1518·78)	2·11 (1·46 to 2·37)	1·56 (0·97 to 1·9)	-25·94 (-38·01 to -14·4)	855635·73 (603128·35 to 1143300·47)	1700681·01 (1198840·65 to 2294152·32)	2132·14 (1506·43 to 2845·08)	2246·44 (1585·66 to 3014·79)	5·36 (2·31 to 8·73)
**Rheumatoid arthritis**	134·19 (104·52 to 169·87)	220·66 (155·79 to 332·36)	0·58 (0·41 to 0·73)	0·25 (0·17 to 0·38)	-56·57 (-69·33 to -38·6)	12713·13 (9536·07 to 16373·89)	29201·43 (21353·61 to 38703·71)	35·39 (27·18 to 44·91)	35·24 (25·66 to 46·69)	-0·41 (-13·43 to 11·46)

**Osteoarthritis**	NA	NA	NA	NA	NA	116173·35 (58609·89 to 235425·29)	334791·59 (169397·27 to 661648·87)	372·14 (189·15 to 746·52)	369·39 (186·93 to 729·56)	-0·74 (-3·23 to 2·03)
**Osteoarthritis hip**	NA	NA	NA	NA	NA	4263·49 (1961·28 to 8752·12)	13659·86 (6492·02 to 28490·65)	13·78 (6·39 to 28·08)	15·26 (7·25 to 31·7)	10·75 (1·91 to 19·56)
**Osteoarthritis knee**	NA	NA	NA	NA	NA	60711·95 (29724·79 to 123248·9)	179409·43 (89536·86 to 364811·07)	194·76 (96·21 to 394·45)	197·96 (98·58 to 402·76)	1·64 (-2·79 to 6·42)
**Osteoarthritis hand**	NA	NA	NA	NA	NA	45151·87 (22928·88 to 93461·21)	122864·61 (61272·73 to 256518·05)	143·48 (72·48 to 293·95)	135·2 (68·07 to 281·23)	-5·77 (-7·85 to -3·79)
**Osteoarthritis other**	NA	NA	NA	NA	NA	6046·04 (2922·37 to 12628·32)	18857·69 (9321·12 to 39159·95)	20·12 (9·94 to 41·83)	20·97 (10·38 to 43·47)	4·21 (-2·45 to 10·73)
**Low back pain**	NA	NA	NA	NA	NA	442336·83 (306566·74 to 601507·13)	714324·8 (501072·42 to 966622·63)	1054·29 (738·6 to 1428·24)	993·31 (693·92 to 1338·83)	-5·78 (-9·66 to -1·62)
**Neck pain**	NA	NA	NA	NA	NA	69942·12 (46032·26 to 100424·73)	132335·85 (86384·5 to 194651·27)	172·29 (113·62 to 247·81)	171·97 (112·56 to 247·48)	-0·19 (-3·33 to 3·02)
**Gout**	NA	NA	NA	NA	NA	6630·34 (4101·14 to 9687·02)	18854·71 (11572·42 to 27691·75)	19·19 (11·9 to 27·38)	21·96 (13·66 to 31·95)	14·44 (0·78 to 29·36)
**Other musculoskeletal disorders**	314·93 (267·1 to 401·7)	1035 (623·05 to 1256·03)	1·53 (1·01 to 1·78)	1·31 (0·78 to 1·59)	-14·4 (-28·57 to -0·57)	207839·97 (139158·15 to 291866·09)	471172·63 (322489·75 to 651743·73)	478·84 (325·25 to 664·71)	654·57 (452·64 to 901·73)	36·7 (26·67 to 48·24)
**Other non-communicable diseases**	3795·84 (3112·37 to 4267·67)	3233·96 (2200·31 to 3722·79)	12·12 (10·37 to 13·59)	5·46 (4·1 to 6·21)	-54·97 (-65·88 to -47·62)	666595·15 (534873·45 to 834072·36)	613458·72 (442732·56 to 843545·06)	1710·6 (1396·71 to 2096·34)	1054·84 (790·79 to 1391·06)	-38·34 (-46·56 to -28·16)
**Congenital birth defects**	2610·17 (1772·68 to 3047·42)	418·75 (360·96 to 528·92)	7·57 (5·12 to 8·88)	1·64 (1·38 to 2·11)	-78·3 (-82·97 to -59·15)	270986·95 (197478·9 to 312692·64)	70632·04 (56952·4 to 86411·87)	764·93 (548·2 to 882·11)	242·81 (197·94 to 296·71)	-68·26 (-74·54 to -49·37)
**Neural tube defects**	34·25 (20·55 to 49·6)	33·22 (24·05 to 44·67)	0·1 (0·06 to 0·15)	0·14 (0·1 to 0·2)	44·45 (-13·98 to 177·81)	4425·71 (3147·12 to 5901·99)	4503·47 (3481·46 to 5739·1)	11·76 (8·09 to 16·04)	16·75 (12·68 to 21·72)	42·36 (-3·96 to 126·57)
**Congenital heart anomalies**	1769·28 (907·66 to 2135·56)	185·58 (153·34 to 229·45)	5·13 (2·57 to 6·23)	0·69 (0·54 to 0·86)	-86·65 (-90·53 to -70·6)	154416·89 (77789·77 to 186122·43)	16963·87 (13682·56 to 21175·18)	450·25 (222·28 to 546·85)	64·61 (51·64 to 80·57)	-85·65 (-89·76 to -68·53)
**Orofacial clefts**	14·01 (5·96 to 26·84)	0·27 (0·18 to 0·39)	0·04 (0·02 to 0·08)	0 (0 to 0)	-97·05 (-98·54 to -92·38)	2547·63 (1611·98 to 3823·21)	1547·81 (924·51 to 2296·26)	6·93 (4·33 to 10·68)	3·25 (1·98 to 4·84)	-53·03 (-71·73 to -26·25)
**Down syndrome**	79·83 (48·47 to 106·96)	26·84 (20·5 to 35·15)	0·22 (0·13 to 0·3)	0·09 (0·07 to 0·12)	-58·16 (-74·21 to -16·17)	8701·69 (5903·28 to 11423·46)	3910·7 (2966·86 to 5089·88)	22·91 (15·21 to 30·33)	12·75 (9·7 to 16·83)	-44·34 (-60·98 to -6·91)
**Turner syndrome**	NA	NA	NA	NA	NA	203·5 (85·53 to 365·55)	152·97 (68·52 to 266·81)	0·42 (0·18 to 0·77)	0·36 (0·15 to 0·64)	-15·49 (-45·19 to 32·21)

**Klinefelter syndrome**	NA	NA	NA	NA	NA	1·39<br>(0·58 to 2·78)	1·47<br>(0·61 to 3)	0<br>(0 to 0·01)	0<br>(0 to 0·01)	16·49<br>(0·56 to 37·4)
**Other chromosomal abnormalities**	57·22<br>(37·78 to 148·2)	48·83<br>(31·24 to 99·34)	0·17<br>(0·11 to 0·44)	0·21<br>(0·13 to 0·46)	26·05<br>(-40·23 to 156·64)	8524·76<br>(6149·09 to 16630·55)	6910·39<br>(4965·89 to 11363·14)	22·5<br>(16·01 to 46·65)	26·42<br>(18·56 to 48·22)	17·39<br>(-26·92 to 101·15)
**Congenital musculoskeletal and limb anomalies**	66·75<br>(33·25 to 93·72)	18·66<br>(11·22 to 25·41)	0·19<br>(0·1 to 0·28)	0·08<br>(0·05 to 0·11)	-58·42<br>(-79·5 to -20·46)	33834·59<br>(22514·65 to 48265·05)	21505·29<br>(14330·73 to 30877·41)	83·55<br>(55·87 to 118·19)	64·69<br>(42·22 to 94·16)	-22·57<br>(-34·64 to -9·32)
**Urogenital congenital anomalies**	25·14<br>(17·23 to 38·07)	8·74<br>(7·12 to 11·02)	0·07<br>(0·05 to 0·11)	0·03<br>(0·02 to 0·04)	-61·1<br>(-77·34 to -32·44)	3922·26<br>(2815·26 to 5599·47)	1960·75<br>(1340·07 to 2814·95)	10·41<br>(7·4 to 14·84)	6·7<br>(4·53 to 9·6)	-35·66<br>(-55·33 to -13)
**Digestive congenital anomalies**	167·59<br>(75·28 to 233·94)	24·97<br>(17·18 to 33·45)	0·5<br>(0·22 to 0·7)	0·11<br>(0·07 to 0·15)	-78·64<br>(-88·1 to -47·18)	15673·44<br>(7607·24 to 21741·74)	3411·46<br>(2552·87 to 4410·97)	46·21<br>(22·14 to 64·33)	13·07<br>(9·53 to 16·79)	-71·71<br>(-82·85 to -36·9)
**Other congenital birth defects**	396·1<br>(209·57 to 584·74)	71·64<br>(55·42 to 95·74)	1·15<br>(0·61 to 1·69)	0·3<br>(0·23 to 0·41)	-74·05<br>(-84·27 to -40·9)	38735·1<br>(21940·77 to 55039·41)	9763·85<br>(7249·02 to 14384·69)	109·99<br>(61·38 to 158·26)	34·21<br>(26·45 to 45·62)	-68·89<br>(-80·21 to -36·87)
**Urinary diseases and male infertility**	290·84<br>(247·38 to 523·28)	1795·44<br>(910·54 to 2203·97)	1·55<br>(1·26 to 2·91)	2·31<br>(1·16 to 2·84)	49·32<br>(-58·21 to 104·25)	14359·64<br>(11331·77 to 18680·51)	38354·29<br>(26824·01 to 47244·01)	44·5<br>(35·72 to 63·2)	48·53<br>(33·71 to 59·65)	9·05<br>(-39·36 to 27·76)
**Urinary tract infections and interstitial nephritis**	182·49<br>(134·4 to 345·49)	1110·38<br>(608·92 to 1619·09)	1<br>(0·75 to 2·07)	1·43<br>(0·78 to 2·09)	42<br>(-58·49 to 146·02)	5629<br>(4370·61 to 7996·94)	15084·72<br>(9394·8 to 20479·24)	18·73<br>(14·51 to 30·65)	19·8<br>(12·68 to 26·49)	5·75<br>(-55·34 to 62·31)
**Urolithiasis**	18·34<br>(12·02 to 30·31)	63·51<br>(32·58 to 106·59)	0·09<br>(0·06 to 0·15)	0·08<br>(0·04 to 0·13)	-12·72<br>(-66·18 to 62·38)	2051·8<br>(1394·51 to 2839·29)	3935·61<br>(2669·57 to 5431·41)	5·37<br>(3·7 to 7·39)	4·99<br>(3·41 to 7·02)	-7·02<br>(-27·12 to 15·18)
**Benign prostatic hyperplasia**	NA	NA	NA	NA	NA	2486·32<br>(1411·87 to 3894·11)	9403·2<br>(5378·55 to 14797·98)	8·56<br>(4·91 to 13·3)	10·17<br>(5·84 to 15·91)	18·77<br>(5·73 to 32·67)
**Male infertility**	NA	NA	NA	NA	NA	996·7<br>(366·43 to 2356·73)	1163·82<br>(420·8 to 2682·06)	1·96<br>(0·72 to 4·6)	2·06<br>(0·75 to 4·73)	5·18<br>(-5·48 to 17·91)
**Other urinary diseases**	90·02<br>(70·29 to 165·8)	621·55<br>(236·97 to 920·41)	0·46<br>(0·35 to 0·85)	0·81<br>(0·3 to 1·21)	77·66<br>(-58·18 to 207·63)	3195·81<br>(2587·16 to 4766·59)	8766·95<br>(4525·06 to 11894·75)	9·88<br>(8·02 to 15·77)	11·5<br>(6·08 to 15·5)	16·4<br>(-55·66 to 78·82)
**Gynecological diseases**	14·33<br>(5·98 to 18·93)	13·47<br>(8·01 to 29·36)	0·06<br>(0·02 to 0·07)	0·02<br>(0·01 to 0·04)	-68·77<br>(-78·88 to -25·69)	113918·09<br>(76829·3 to 162063·54)	122687·96<br>(83904·92 to 171786·45)	228·82<br>(155·49 to 323·08)	210·96<br>(143·05 to 301·21)	-7·8<br>(-11·79 to -3·84)
**Uterine fibroids**	6·99<br>(3·18 to 10·62)	4·4<br>(2·67 to 9·47)	0·03<br>(0·01 to 0·04)	0·01<br>(0 to 0·01)	-79<br>(-87·1 to -53·88)	4864·95<br>(2425·71 to 8986·98)	6677·11<br>(3198·42 to 12651·32)	10·38<br>(5·25 to 19·12)	10·15<br>(4·83 to 19·74)	-2·22<br>(-12·49 to 6·47)
**Polycystic ovarian syndrome**	NA	NA	NA	NA	NA	4280·25<br>(1772·46 to 9001·31)	6937·74<br>(2905·16 to 14689·35)	8·2<br>(3·41 to 17·33)	13·01<br>(5·47 to 27·44)	58·66<br>(40·43 to 80·6)
**Female infertility**	NA	NA	NA	NA	NA	458·7<br>(39·06 to 1592·22)	551·09<br>(41·3 to 2000·37)	0·96<br>(0·08 to 3·43)	0·9<br>(0·07 to 3·23)	-6·26<br>(-52·55 to 74·27)

**Endometriosis**	0·63 (0·1 to 1·61)	0·08 (0·02 to 0·24)	0 (0 to 0)	0 (0 to 0)	-90·1 (-97·71 to -44·09)	17928·31 (10304·28 to 28963·03)	16554·24 (9538·2 to 26107·24)	34·99 (20·34 to 55·32)	28·94 (16·97 to 46·2)	-17·28 (-27·48 to -6·94)
**Genital prolapse**	4·91 (1·51 to 6·9)	4·13 (2·44 to 9·6)	0·02 (0·01 to 0·03)	0·01 (0 to 0·01)	-72·52 (-83·57 to -27·15)	922·04 (514·81 to 1603·16)	1837·2 (903·15 to 3423·87)	2·96 (1·62 to 5·26)	2·09 (1·04 to 3·82)	-29·58 (-40·22 to -18·02)
**Premenstrual syndrome**	NA	NA	NA	NA	NA	46747·65 (28298·31 to 74168·39)	44106·27 (26475·27 to 69450·02)	91·6 (54·96 to 144·83)	82·92 (49·79 to 130·55)	-9·47 (-14·58 to -4·3)
**Other gynecological diseases**	1·8 (0·62 to 2·72)	4·85 (2·44 to 10·7)	0·01 (0 to 0·01)	0·01 (0 to 0·01)	-21·58 (-56·44 to 118·15)	38716·2 (24165·7 to 58588·73)	46024·31 (28580·36 to 68332·53)	79·73 (49·63 to 118·56)	72·95 (45·15 to 109·44)	-8·51 (-14·79 to -2·85)
**Hemoglobinopathies and hemolytic anaemias**	224·18 (211·15 to 239·6)	304·84 (264·14 to 340·33)	0·69 (0·64 to 0·74)	0·4 (0·35 to 0·44)	-42·44 (-49·54 to -35·63)	27779·95 (21699·32 to 35828·79)	13862·15 (10863·33 to 17814·6)	64 (50·44 to 81·85)	23·75 (18·36 to 31·6)	-62·89 (-67·36 to -57·97)
**Thalassemias**	54·07 (46·02 to 62·76)	12·33 (9·9 to 14·93)	0·12 (0·1 to 0·14)	0·04 (0·03 to 0·05)	-66·36 (-73·68 to -57·46)	4181·46 (3573·22 to 4835·29)	930·26 (747·75 to 1118·44)	9·34 (7·94 to 10·82)	3·16 (2·54 to 3·82)	-66·2 (-73·65 to -57·21)
**Thalassemias trait**	NA	NA	NA	NA	NA	2048·81 (1228·02 to 3184·62)	1541·55 (884·7 to 2453·07)	4·03 (2·4 to 6·25)	3 (1·75 to 4·81)	-25·58 (-41·13 to -5·21)
**Sickle cell disorders**	NA	NA	NA	NA	NA	104·75 (67·27 to 151·45)	48·75 (30·57 to 69·82)	0·23 (0·15 to 0·34)	0·12 (0·07 to 0·17)	-49·13 (-61·66 to -32·87)
**Sickle cell trait**	1·53 (0·99 to 2·21)	0·83 (0·51 to 1·19)	0 (0 to 0)	0 (0 to 0)	-46·13 (-59·03 to -30·2)	936·55 (535·85 to 1491·12)	494·64 (266·73 to 796)	1·87 (1·08 to 2·97)	0·97 (0·52 to 1·56)	-48·24 (-60·61 to -28·85)
**G6PD deficiency**	11·34 (7·36 to 15·75)	10·33 (6·88 to 14·28)	0·02 (0·02 to 0·03)	0·01 (0·01 to 0·02)	-38 (-47·13 to -26·18)	939·42 (632·86 to 1306·64)	521·89 (361·19 to 723·24)	1·85 (1·26 to 2·56)	0·89 (0·61 to 1·25)	-52·09 (-63·41 to -37·95)
**G6PD trait**	NA	NA	NA	NA	NA	116·68 (59·59 to 228·43)	46·75 (21·79 to 85·92)	0·23 (0·12 to 0·42)	0·09 (0·04 to 0·17)	-59·65 (-81·11 to -22·61)
**Other hemoglobinopathies and hemolytic anemias**	157·24 (145·5 to 170·05)	281·36 (241·83 to 315·46)	0·54 (0·5 to 0·59)	0·34 (0·29 to 0·38)	-37·42 (-45·42 to -29·18)	19452·28 (14570·77 to 26137·86)	10278·31 (8068·46 to 13185·05)	46·44 (35·27 to 61·79)	15·52 (11·82 to 20·87)	-66·57 (-71·1 to -61·42)
**Endocrine, metabolic, blood, and immune disorders**	488·41 (400·47 to 634·2)	665·78 (453·5 to 817·6)	1·74 (1·36 to 2·1)	0·91 (0·63 to 1·14)	-47·71 (-61·66 to -33·11)	139198·07 (99894·94 to 188363·71)	201359·31 (137332·17 to 279194·45)	335·23 (241·69 to 445·54)	294·53 (202·37 to 406·41)	-12·14 (-20·23 to -4·74)
**Oral disorders**	NA	NA	NA	NA	NA	85504·95 (49439·21 to 138763·49)	163407·11 (94062·08 to 267144·96)	227·86 (134·28 to 367·8)	218·64 (125·63 to 352·33)	-4·05 (-7·76 to -0·73)
**Caries of deciduous teeth**	NA	NA	NA	NA	NA	1232·62 (539·22 to 2541·91)	748·67 (329·73 to 1561·78)	3·25 (1·41 to 6·73)	3·18 (1·39 to 6·66)	-2·06 (-11·12 to 8·84)
**Caries of permanent teeth**	NA	NA	NA	NA	NA	12466·26 (5555·56 to 24175·18)	14749·24 (6631·22 to 29317·57)	26·74 (12·01 to 52·27)	24·94 (11·29 to 49·42)	-6·74 (-13·47 to -0·1)

Periodontal diseases	NA	NA	NA	NA	NA	22993·75 (8674·65 to 54730·82)	55147·9 (21844·29 to 126643·82)	60·37 (23·27 to 141·14)	66·46 (26·01 to 156·95)	10·1 (4·5 to 16·54)
Edentulism	NA	NA	NA	NA	NA	24844·04 (15819·75 to 37349·55)	61086·37 (38862·5 to 90611·25)	84·31 (53·6 to 124·29)	70·89 (44·7 to 105·63)	-15·92 (-20·42 to -11·86)
Other oral disorders	NA	NA	NA	NA	NA	23968·28 (14846 to 35507·53)	31674·93 (19552·01 to 46607·2)	53·19 (33·13 to 78·79)	53·17 (32·99 to 78·59)	-0·05 (-3·15 to 3·23)
Sudden infant death syndrome	167·9 (49·27 to 339·15)	35·68 (12·55 to 65·16)	0·51 (0·15 to 1·03)	0·18 (0·06 to 0·32)	-65·5 (-84·92 to -18·98)	14847·49 (4357·51 to 29986·23)	3155·87 (1109·6 to 5762·44)	45·26 (13·28 to 91·38)	15·62 (5·49 to 28·5)	-65·49 (-84·92 to -18·99)
Injuries	33616·18 (32369·81 to 38230·26)	31859·41 (26038·74 to 34652·78)	84·35 (80·65 to 98·69)	42·62 (35·86 to 46·1)	-49·48 (-62·13 to -44·25)	2178421·44 (2013837·15 to 2379367·79)	1650618·81 (1404181·32 to 1937727·89)	4960·86 (4559·44 to 5447·42)	2439·93 (2101·91 to 2844·22)	-50·82 (-57·79 to -46·63)
Transport injuries	16121·36 (14824·8 to 17104·52)	7553·3 (6716·07 to 9432·18)	39·61 (36·48 to 42·41)	10·14 (8·98 to 13·02)	-74·41 (-77·37 to -67·78)	894074·16 (831067·92 to 947954·87)	305239·51 (268887·19 to 385134·66)	2002·96 (1855·78 to 2132·49)	464·65 (408·85 to 604·33)	-76·8 (-79·53 to -70·4)
Road injuries	15003·64 (13792·62 to 15926·85)	6882·36 (6086·06 to 8579·83)	36·94 (33·99 to 39·59)	9·3 (8·21 to 11·9)	-74·82 (-77·77 to -68·14)	831547·13 (773021·23 to 883232·38)	276741·88 (243751·47 to 350526·34)	1864·79 (1725·75 to 1984·37)	424·9 (372·93 to 555·04)	-77·21 (-79·92 to -70·74)
Pedestrian road injuries	6243·33 (4697·39 to 7385·87)	3376·93 (2904·02 to 4146·72)	17·43 (13·75 to 20·39)	4·28 (3·68 to 5·24)	-75·42 (-80·1 to -64·5)	306474·01 (226445·47 to 362810·46)	94245·37 (82470·19 to 118575·71)	747·83 (566·14 to 875·57)	138·07 (120·07 to 174·23)	-81·54 (-85·31 to -70·77)
Cyclist road injuries	925·84 (754·46 to 1253·31)	421·51 (303·17 to 517·73)	2·26 (1·84 to 3·08)	0·53 (0·38 to 0·65)	-76·66 (-87·12 to -68·99)	62035·44 (51791·14 to 78367·31)	28211·62 (22253·06 to 35053·71)	143·37 (120·12 to 180·71)	38·97 (30·62 to 48·28)	-72·82 (-81·36 to -66·75)
Motorcyclist road injuries	3286·94 (2603·92 to 4340·04)	1037·35 (685·86 to 1814·79)	6·9 (5·4 to 9·18)	1·54 (0·98 to 2·79)	-77·65 (-88·21 to -60·25)	200551·88 (163670·77 to 257831·09)	52392·79 (36965·3 to 90738·38)	409·72 (334·56 to 527·98)	86·93 (57·48 to 154·79)	-78·78 (-87·93 to -62·14)
Motor vehicle road injuries	4367·81 (3212·85 to 5219·02)	2028·11 (1700·3 to 2766·41)	9·9 (7 to 11·82)	2·92 (2·45 to 4·32)	-70·5 (-78·71 to -49·56)	252375·95 (198608·7 to 298606·49)	99783·33 (84178·32 to 138103·33)	541·33 (414·78 to 639·92)	157·84 (131·85 to 233·12)	-70·84 (-78·21 to -51·34)
Other road injuries	179·72 (128·79 to 257·75)	18·46 (12·51 to 23·06)	0·44 (0·32 to 0·62)	0·03 (0·02 to 0·03)	-93·99 (-97·02 to -91·25)	10109·85 (7514·8 to 14128·16)	2108·77 (1574·77 to 2744·4)	22·54 (16·84 to 31·36)	3·08 (2·33 to 3·93)	-86·32 (-91·4 to -80·77)
Other transport injuries	1117·73 (1011·29 to 1217·35)	670·94 (550·92 to 836·79)	2·67 (2·41 to 2·93)	0·83 (0·7 to 1·06)	-68·84 (-73·93 to -61·17)	62527·03 (56760·35 to 68293·44)	28497·63 (24447·55 to 35171·71)	138·17 (125·55 to 150·23)	39·75 (34·14 to 50·14)	-71·23 (-74·9 to -64·21)
Unintentional injuries	11620·19 (10473·48 to 12289·53)	8980·22 (7360·76 to 10145·53)	31·08 (29·37 to 34·29)	11·85 (9·87 to 13·41)	-61·87 (-69·21 to -55·61)	965780·05 (828456·84 to 1129617·77)	775114·89 (588819·01 to 1015312·25)	2289·96 (1977·75 to 2675·23)	1113·85 (853·62 to 1458·82)	-51·36 (-58·54 to -44·62)
Falls	3192·26 (2335·2 to 3863·13)	5259·74 (3921·95 to 6269·45)	9·48 (7·63 to 13·05)	6·72 (5·05 to 8·14)	-29·15 (-58·36 to -1·32)	299182·46 (233466·23 to 370096·38)	389491·21 (296598·25 to 508310·63)	732·46 (581·64 to 898·21)	528·63 (403·74 to 688·36)	-27·83 (-37·19 to -11·13)
Drowning	2466·77 (1866·58 to 2666·05)	691·1 (568·56 to 786·55)	5·71 (4·55 to 6·15)	1·03 (0·86 to 1·18)	-81·91 (-84·77 to -77·31)	154042·5 (111728·89 to 168371·16)	27189·01 (22616·55 to 30975·64)	339·64 (255·56 to 371·19)	49·83 (41·46 to 57·98)	-85·33 (-87·76 to -80·5)

**Fire, heat, and hot substances**	866·09<br>(660·88 to 1045·7)	557·13<br>(426·31 to 839·95)	2·39<br>(1·91 to 3·04)	0·76<br>(0·58 to 1·14)	-68·43<br>(-75·11 to -45·66)	74506·09<br>(57902·32 to 90112·49)	47108·31<br>(33182·49 to 68328·06)	171·93<br>(136·14 to 211·08)	70·83<br>(50·26 to 101·86)	-58·8<br>(-67·36 to -43·94)
**Poisonings**	914·11<br>(619·81 to 1019·27)	211·56<br>(182·83 to 303·58)	2·37<br>(1·55 to 2·64)	0·28<br>(0·24 to 0·42)	-88·28<br>(-90·43 to -72·87)	52657·88<br>(38566·98 to 58784·48)	13327·8<br>(10334·34 to 18960·84)	117·96<br>(84·46 to 131·39)	21·77<br>(16·51 to 31·63)	-81·54<br>(-86 to -64·54)
**Poisoning by carbon monoxide**	579·68<br>(319·67 to 713·12)	122·05<br>(97·87 to 194·98)	1·4<br>(0·73 to 1·74)	0·17<br>(0·14 to 0·29)	-87·97<br>(-91·69 to -69·04)	33244·54<br>(19591·5 to 40808·27)	5426·85<br>(4358·97 to 8820·65)	72·05<br>(40·95 to 88·42)	8·93<br>(7·15 to 15·06)	-87·6<br>(-91·19 to -69·59)
**Poisoning by other means**	334·43<br>(216·16 to 595·64)	89·51<br>(52·43 to 113·58)	0·97<br>(0·61 to 1·65)	0·11<br>(0·06 to 0·15)	-88·73<br>(-95·71 to -77·96)	19413·35<br>(14124·3 to 32692·92)	7900·95<br>(5529·8 to 10717·15)	45·91<br>(33·25 to 75·98)	12·84<br>(8·78 to 17·84)	-72·03<br>(-85·41 to -55·48)
**Exposure to mechanical forces**	828·17<br>(693·17 to 953·24)	533·06<br>(382·97 to 622·05)	1·96<br>(1·64 to 2·24)	0·71<br>(0·54 to 0·81)	-63·96<br>(-72·72 to -58·08)	119845·77<br>(93118·86 to 156243·61)	146231·23<br>(102626·63 to 207998·79)	277·36<br>(213·64 to 362·12)	207·59<br>(145·53 to 295·91)	-25·16<br>(-33·72 to -17·28)
**Unintentional firearm injuries**	38·23<br>(15·92 to 62·93)	8·01<br>(5·98 to 12·6)	0·09<br>(0·04 to 0·14)	0·01<br>(0·01 to 0·02)	-83·61<br>(-90·81 to -55·87)	3694·21<br>(2374·49 to 5162·01)	3131·99<br>(2268·16 to 4162·32)	8·05<br>(5·39 to 11·09)	4·65<br>(3·41 to 6·1)	-42·16<br>(-59·67 to -10·02)
**Other exposure to mechanical forces**	**789·94**<br>**(660·4 to 914·38)**	525·05<br>(375·04 to 613·72)	1·87<br>(1·57 to 2·15)	0·69<br>(0·52 to 0·79)	-63·04<br>(-72·21 to -56·84)	116151·55<br>(89499·4 to 151273·95)	143099·24<br>(100482·02 to 203980·64)	269·31<br>(207·14 to 351·28)	202·93<br>(142·16 to 289·73)	-24·65<br>(-33·33 to -16·69)
**Adverse effects of medical treatment**	150·98<br>(126·82 to 219·95)	215·53<br>(142·12 to 258·45)	0·52<br>(0·43 to 0·78)	0·28<br>(0·18 to 0·34)	-46·13<br>(-71·57 to -25·95)	6602·41<br>(5542·37 to 9254·25)	5143·32<br>(3534·05 to 6048·79)	17·85<br>(15 to 25·15)	8·48<br>(5·66 to 10·28)	-52·5<br>(-73·52 to -36·59)
**Animal contact**	52·85<br>(47·45 to 63·03)	59·28<br>(40·54 to 69·32)	0·16<br>(0·14 to 0·19)	0·07<br>(0·05 to 0·09)	-52·91<br>(-70·09 to -43·13)	6274·16<br>(4889·13 to 8196·94)	6958·8<br>(5099·56 to 9612·68)	15·28<br>(11·99 to 19·8)	10·48<br>(7·73 to 14·55)	-31·42<br>(-39·75 to -25·51)
**Venomous animal contact**	34·71<br>(29·68 to 40·57)	45·37<br>(30·83 to 53·68)	0·1<br>(0·09 to 0·12)	0·05<br>(0·04 to 0·06)	-45·76<br>(-65·64 to -32·59)	3079·94<br>(2464·64 to 3855·83)	3344·92<br>(2599·14 to 4208·61)	7·38<br>(5·94 to 9·15)	5·02<br>(3·83 to 6·45)	-31·99<br>(-40·77 to -25·01)
**Non-venomous animal contact**	18·14<br>(15·19 to 23·42)	13·91<br>(9·75 to 16·83)	0·06<br>(0·05 to 0·07)	0·02<br>(0·01 to 0·02)	-65·59<br>(-78·9 to -55·45)	3194·22<br>(2292·82 to 4524·97)	3613·88<br>(2356 to 5547·45)	7·9<br>(5·75 to 11·12)	5·46<br>(3·62 to 8·27)	-30·89<br>(-41·44 to -23·3)
**Foreign body**	1529·22<br>(1215·63 to 1680·57)	971·01<br>(839·79 to 1141·6)	4·55<br>(3·86 to 4·99)	1·38<br>(1·2 to 1·62)	-69·58<br>(-74·48 to -56·65)	94943·02<br>(72879·19 to 108087·07)	33191·4<br>(28650·15 to 39231·92)	256·81<br>(201·92 to 296·19)	59·65<br>(51·47 to 70·04)	-76·77<br>(-81·03 to -65·52)
**Pulmonary aspiration and foreign body in airway**	1511·61<br>(1206·36 to 1662·1)	960·41<br>(830·57 to 1126·73)	4·5<br>(3·84 to 4·95)	1·36<br>(1·18 to 1·6)	-69·66<br>(-74·47 to -57·09)	86538·76<br>(66091·63 to 99677·9)	21373·15<br>(18851·59 to 25183·47)	236·98<br>(183·54 to 276·74)	41·63<br>(35·76 to 49·97)	-82·43<br>(-85·73 to -72·72)
**Foreign body in eyes**	**17·61**<br>**(9·98 to 25·78)**	10·6<br>(7·89 to 17·19)	0·05<br>(0·03 to 0·07)	0·02<br>(0·01 to 0·03)	-62·43<br>(-76·88 to -11·86)	2085·93<br>(933·85 to 3774·63)	2759·49<br>(1447·31 to 4655·01)	4·64<br>(2·19 to 8·2)	4·65<br>(2·2 to 8·23)	0·33<br>(-1·05 to 1·45)
**Foreign body in other body part**	NA	NA	NA	NA	NA	6318·33<br>(4651·57 to 8342·8)	9058·75<br>(6447·51 to 12472·95)	15·2<br>(11·22 to 20·1)	13·37<br>(9·59 to 18·25)	-12·04<br>(-22·6 to -3·29)
**Environmental heat and cold exposure**	331·57<br>(278·07 to 570·47)	301·29<br>(142·57 to 373·51)	0·97<br>(0·81 to 1·68)	0·37<br>(0·18 to 0·46)	-61·84<br>(-80·31 to -47·4)	17613·87<br>(14690·58 to 27263·13)	13386·55<br>(9316·08 to 16527·69)	42·7<br>(35·63 to 66·84)	18·28<br>(13·17 to 22·88)	-57·18<br>(-71·72 to -47·82)

**Exposure to forces of nature**	154 (139·83 to 169·44)	0 (0 to 0)	0·37 (0·33 to 0·41)	0 (0 to 0)	-100 (-100 to -100)	9027·58 (8188·09 to 9939·46)	590·14 (425·64 to 815·42)	19·86 (18·02 to 21·86)	0·8 (0·56 to 1·1)	-95·99 (-97·05 to -94·58)
**Other unintentional injuries**	1134·18 (574·39 to 1278·98)	180·52 (149·99 to 296·11)	2·61 (1·43 to 2·91)	0·25 (0·21 to 0·43)	-90·24 (-92·44 to -71·35)	131084·31 (87967·57 to 167869·42)	92497·11 (60341·36 to 134625·1)	298·11 (206·08 to 385·52)	137·51 (90·05 to 200·71)	-53·87 (-63·26 to -38·17)
**Self-harm and interpersonal violence**	5874·62 (5105·68 to 11070·73)	15325·89 (9714·42 to 17219·52)	13·66 (11·85 to 26·07)	20·63 (13·43 to 23·02)	51·05 (-40·34 to 89·14)	318567·22 (279635·98 to 554571·59)	570264·41 (383194·69 to 633714·38)	667·94 (584·61 to 1185·28)	861·43 (596·77 to 950·28)	28·97 (-43·64 to 57·75)
**Self-harm**	5031·87 (4269·2 to 10223·3)	14575·18 (8993·67 to 16436·88)	11·77 (9·97 to 23·97)	19·54 (12·39 to 21·88)	66·09 (-39·86 to 113·01)	256246·58 (219027·94 to 496694·5)	522651·08 (338100·09 to 584676·65)	534·78 (456·44 to 1054·5)	781·01 (515·37 to 868·5)	46·04 (-43·48 to 85·06)
**Self-harm by firearm**	35·02 (20·97 to 93·98)	22·51 (10·71 to 32·14)	0·07 (0·05 to 0·2)	0·03 (0·02 to 0·05)	-55·28 (-85·41 to -14·91)	1986·77 (1193·71 to 4958·16)	1187·85 (738·72 to 1612·94)	3·97 (2·44 to 10·07)	1·88 (1·11 to 2·62)	-52·71 (-82·63 to -8·37)
**Self-harm by other specified means**	4996·85 (4239·5 to 10146·02)	14552·67 (8982·64 to 16411·59)	11·69 (9·9 to 23·81)	19·51 (12·38 to 21·84)	66·87 (-39·41 to 113·98)	254259·81 (217257·78 to 492584·47)	521463·23 (336972·15 to 583421·55)	530·81 (452·69 to 1045·38)	779·13 (514·35 to 866·57)	46·78 (-43·25 to 86·08)
**Interpersonal violence**	808·3 (732·73 to 874·27)	745·58 (657·9 to 841·37)	1·8 (1·65 to 1·94)	1·08 (0·96 to 1·21)	-40·02 (-48·2 to -30·96)	60864·45 (54523·94 to 67149·76)	47248·88 (40856·01 to 53822·14)	129·59 (116·44 to 143·1)	79·7 (68·97 to 91·04)	-38·5 (-44·88 to -32·1)
**Physical violence by firearm**	20·97 (14·48 to 26·99)	13·1 (10·34 to 15·84)	0·04 (0·03 to 0·06)	0·02 (0·02 to 0·03)	-52·5 (-64·95 to -34·92)	1517·52 (1138·85 to 1911·36)	1512·99 (1183·7 to 1877·84)	3·1 (2·38 to 3·81)	2·47 (1·93 to 3·01)	-20·38 (-37·48 to 1·78)
**Physical violence by sharp object**	375·61 (258·18 to 437·86)	165·17 (138·03 to 210·75)	0·79 (0·55 to 0·92)	0·24 (0·2 to 0·3)	-69·99 (-76·76 to -56·3)	22601·56 (16008·88 to 26233·47)	8789·68 (7377·67 to 10662·01)	45·94 (32·87 to 53·24)	13·82 (11·52 to 16·81)	-69·91 (-75·9 to -57·64)
**Sexual violence**	NA	NA	NA	NA	NA	5821·8 (3785·31 to 8252·24)	5659·18 (3757·68 to 8036·76)	11·45 (7·47 to 16·2)	11·32 (7·38 to 16·01)	-1·13 (-1·29 to -0·99)
**Physical violence by other means**	411·72 (358·7 to 484·48)	567·31 (483·06 to 648·05)	0·97 (0·85 to 1·13)	0·82 (0·7 to 0·93)	-14·86 (-32·94 to 2·21)	30923·57 (26645·87 to 36154·69)	31287·03 (26372·4 to 36446·91)	69·1 (59·78 to 80·4)	52·09 (43·72 to 60·43)	-24·61 (-37·93 to -13·04)
**Conflict and terrorism**	29 (26·33 to 31·91)	0 (0 to 0)	0·08 (0·07 to 0·09)	0 (0 to 0)	-100 (-100 to -100)	1147·85 (1038·79 to 1274·57)	120·23 (80·08 to 188·45)	2·89 (2·61 to 3·21)	0·17 (0·11 to 0·27)	-94·09 (-96·02 to -90·76)
**Executions and police conflict**	5·46 (4·51 to 6·98)	5·13 (4·77 to 5·48)	0·01 (0·01 to 0·02)	0·01 (0·01 to 0·01)	-24·07 (-42·2 to -2·58)	308·35 (267·47 to 365·74)	244·22 (227·05 to 261·12)	0·68 (0·58 to 0·82)	0·55 (0·51 to 0·6)	-18·82 (-33·76 to -2·15)

Appendix table 8. Number and age-standardised rate of YLLs and YLDs, and percentage change from 1990 to 2019 for both sex by causes. (YLL= Years of life lost, YLD= Years lived with disability)

	YLLs (95% UI)					YLDs (95% UI)				
Causes	Counts 1990	Counts 2019	Age-standardised estimates 1990	Age-standardised estimates 2019	Percentage change of age-standardised rates between 1990 to 2019 (%)	Counts 1990	Counts 2019	Age-standardised estimates 1990	Age-standardised estimates 2019	Percentage change of age-standardised rates between 1990 to 2019 (%)
All causes	7474359·99 (7351753·65 to 7604241·42)	5995772·93 (5771537·23 to 6231104·13)	22049·55 (21698·89 to 22426·32)	7784·27 (7493·34 to 8075·35)	-64·7 (-66·03 to -63·28)	4183514·38 (3057535·21 to 5426484·19)	6405562·23 (4744459·1 to 8322910·74)	10206·86 (7486·43 to 13250·89)	9407·31 (6951·42 to 12306·28)	-7·83 (-9·53 to -6·14)
Communicable, maternal, neonatal, and nutritional diseases	771657·07 (706802·57 to 857080·51)	347178·81 (264204·04 to 380913·29)	2312·95 (2110·98 to 2586·87)	648·82 (553·13 to 714·26)	-71·95 (-77·65 to -68·2)	431232·94 (302553 to 598203·14)	302411·42 (219106·49 to 402547·17)	979·62 (685·28 to 1353·51)	568·26 (412·34 to 763·16)	-41·99 (-47·26 to -35·6)
HIV/AIDS and sexually transmitted infections	8048·35 (4334·22 to 14937·54)	9114·55 (6624·24 to 13401·6)	22·8 (11·41 to 43·93)	26·27 (13·86 to 47·78)	15·23 (-13·88 to 54·57)	6680·75 (3795·73 to 11634·07)	10335·06 (5931·1 to 16649·66)	14·14 (7·93 to 25·15)	15·19 (8·92 to 24·09)	7·45 (-15·64 to 51·94)
HIV/AIDS	2410·82 (2289·98 to 2524·79)	5107·54 (4748·99 to 5492·92)	5·68 (5·42 to 5·95)	7·59 (7·12 to 8·1)	33·61 (23·58 to 44·39)	414·23 (0 to 710·02)	3778·55 (1554·51 to 7492·06)	0·86 (0 to 1·45)	4·96 (2·12 to 9·45)	476 (0 to 646·52)
HIV/AIDS - drug-susceptible tuberculosis	1068·42 (997·79 to 1123·51)	2191·74 (1870·85 to 2396·24)	2·52 (2·34 to 2·65)	3·26 (2·78 to 3·55)	29·37 (10·28 to 43·68)	228·32 (0 to 358·54)	164·23 (106·58 to 232·16)	0·48 (0 to 0·75)	0·22 (0·14 to 0·32)	-53·17 (-65·8 to 0)
HIV/AIDS– multidrug-resistant tuberculosis without extensive drug resistance	16·45 (2·32 to 63·01)	77·17 (9 to 298·03)	0·04 (0·01 to 0·15)	0·11 (0·01 to 0·44)	196·11 (-68·91 to 2424·11)	1·4 (0 to 5·03)	2·75 (0·35 to 11·43)	0 (0 to 0·01)	0 (0 to 0·02)	28·25 (-89·33 to 723·04)
HIV/AIDS - extensively drug-resistant tuberculosis	0 (0 to 0)	20·39 (2·42 to 78·39)	0 (0 to 0)	0·03 (0 to 0·12)	NA	0 (0 to 0)	0·35 (0·04 to 1·44)	0 (0 to 0)	0 (0 to 0)	0 (0 to 0)
HIV/AIDS resulting in other diseases	1325·95 (1259·49 to 1388·63)	2818·23 (2611·94 to 3059·2)	3·13 (2·98 to 3·27)	4·19 (3·92 to 4·54)	34·05 (23·67 to 46·26)	184·51 (0 to 401·61)	3611·22 (1400·1 to 7301·87)	0·38 (0 to 0·82)	4·73 (1·94 to 9·16)	1139·39 (0 to 1630·3)
Sexually transmitted infections excluding HIV	5637·53 (1903·34 to 12504·8)	4007·01 (1524·67 to 8307·14)	17·11 (5·66 to 38·19)	18·67 (6·17 to 40·2)	9·12 (-27·57 to 64·37)	6266·53 (3437·1 to 11247·78)	6556·51 (3489·65 to 12305·36)	13·27 (7·15 to 24·09)	10·23 (5·59 to 18·59)	-22·96 (-29·27 to -16·01)
Syphilis	5327·45 (1590·5 to 12171·89)	3682·33 (1204·6 to 7964·5)	16·24 (4·79 to 37·25)	18·2 (5·73 to 39·71)	12·11 (-25·47 to 72·47)	209·79 (144·56 to 290·32)	311·72 (216·17 to 424·98)	0·51 (0·36 to 0·7)	0·41 (0·29 to 0·56)	-20·14 (-30·53 to -8·21)
Chlamydial infection	47·42 (41·2 to 57·74)	55·23 (39·77 to 66·68)	0·14 (0·12 to 0·17)	0·08 (0·06 to 0·09)	-46·45 (-65·15 to -32·74)	777·38 (446·43 to 1249·21)	723·53 (420·91 to 1165·43)	1·52 (0·88 to 2·46)	1·29 (0·76 to 2·07)	-14·88 (-29·68 to 6·26)

**Gonococcal infection**	167·87 (149·63 to 192·85)	185·81 (137·94 to 219·98)	0·49 (0·43 to 0·58)	0·26 (0·21 to 0·31)	-46·21 (-62·57 to -34·74)	353·59 (206·78 to 558·54)	256·44 (152·76 to 404·86)	0·68 (0·41 to 1·08)	0·48 (0·29 to 0·74)	-30·42 (-41·73 to -17·29)
**Trichomoniasis**	NA	NA	NA	NA	NA	1085·66 (416·99 to 2268·26)	1444·13 (568·9 to 3022·07)	2·32 (0·89 to 4·84)	2·17 (0·84 to 4·51)	-6·39 (-16·11 to 4·51)
**Genital herpes**	NA	NA	NA	NA	NA	2033·25 (712·96 to 4919·33)	2081·18 (660·3 to 5107·33)	4·64 (1·6 to 11·25)	2·87 (0·93 to 7·07)	-38·12 (-47·4 to -27·67)
**Other sexually transmitted infections**	94·79 (83·96 to 110·91)	83·65 (64·24 to 96·54)	0·24 (0·21 to 0·28)	0·13 (0·11 to 0·15)	-46·08 (-60·88 to -35·83)	1806·86 (1115·26 to 2780·27)	1739·53 (1045·07 to 2705·75)	3·59 (2·21 to 5·52)	3 (1·82 to 4·64)	-16·5 (-28·36 to -3·07)
**Respiratory infections and tuberculosis**	394855·79 (375472·92 to 415454·69)	251927·8 (172949·24 to 281322·83)	1181·87 (1123·74 to 1252·13)	313·42 (221·37 to 349·18)	-73·48 (-82·28 to -70·43)	69493·27 (45694·35 to 102831·31)	61542·49 (38995·5 to 93652·14)	160·92 (106·56 to 233·94)	125·69 (78·42 to 193·4)	-21·89 (-29·99 to -14·85)
**Tuberculosis**	244028·09 (231970·23 to 256241·51)	52444·01 (47086·43 to 58695·04)	678·26 (645·42 to 713·48)	64·06 (57·67 to 71·5)	-90·56 (-91·53 to -89·33)	13859·85 (9362·99 to 19008·92)	5862·5 (3716·36 to 8234·56)	35·95 (24·17 to 48·69)	7·91 (5·03 to 11·12)	-78 (-81·69 to -73·65)
**Latent tuberculosis infection**	NA	NA	NA	NA	NA	0 (0 to 0)	0 (0 to 0)	0 (0 to 0)	0 (0 to 0)	0 (0 to 0)
**Drug-susceptible tuberculosis**	240994·09 (226440·62 to 254513·52)	50602·56 (43596·8 to 57345·97)	669·82 (629·66 to 707·71)	61·81 (53·49 to 69·76)	-90·77 (-92·07 to -89·42)	13783·1 (9327·56 to 18900·34)	5762·51 (3675·25 to 8136·04)	35·75 (24·06 to 48·59)	7·78 (4·93 to 10·85)	-78·24 (-82·11 to -73·76)
**Multidrug-resistant tuberculosis without extensive drug resistance**	3034 (420·69 to 11769·94)	1456·69 (165·83 to 5746·56)	8·44 (1·17 to 32·57)	1·78 (0·2 to 7·03)	-78·91 (-97·86 to 80·24)	76·75 (12·72 to 246·27)	88·82 (11·53 to 370·77)	0·2 (0·03 to 0·65)	0·12 (0·02 to 0·48)	-41·77 (-94·42 to 399·31)
**Extensively drug-resistant tuberculosis**	0 (0 to 0)	384·76 (44·67 to 1576·43)	0 (0 to 0)	0·47 (0·05 to 1·94)	NA	0 (0 to 0)	11·17 (1·45 to 46·62)	0 (0 to 0)	0·01 (0 to 0·06)	0 (0 to 0)
**Lower respiratory infections**	148925·14 (137705·32 to 162855·56)	199135·36 (116796·14 to 225756·3)	497·15 (459·99 to 546·98)	248·75 (152·86 to 281·05)	-49·96 (-71·63 to -42·51)	1630·67 (1072·26 to 2403·68)	1347·92 (872·02 to 1908·58)	4·1 (2·7 to 6)	2·93 (1·91 to 4·23)	-28·55 (-39·53 to -16·5)
**Upper respiratory infections**	1866·24 (370·91 to 3356·88)	338·64 (211·58 to 699·33)	6·37 (1·07 to 12·01)	0·59 (0·37 to 1·36)	-90·72 (-96·28 to -15·37)	43866·02 (26320·33 to 69102·53)	46462·23 (28026·94 to 73079·02)	98·15 (58·87 to 154·49)	98·25 (58·98 to 156·98)	0·1 (-4·3 to 5·25)
**Otitis media**	36·32 (23·56 to 46·23)	9·79 (5·4 to 16·59)	0·09 (0·06 to 0·12)	0·01 (0·01 to 0·02)	-88·27 (-93·19 to -80·14)	10136·74 (5810·83 to 16740·61)	7869·84 (4412·55 to 13150·05)	22·72 (13·03 to 38·07)	16·6 (9·29 to 27·92)	-26·91 (-42·43 to -16·69)
**Enteric infections**	23049·71 (15457·86 to 31253·04)	14897·26 (8600·98 to 25959·81)	77·2 (50·65 to 109·41)	21·66 (13·86 to 35·5)	-71·95 (-78·95 to -58·04)	14640·83 (9932·2 to 20483·13)	27506·69 (18817·46 to 37273·17)	36·11 (24·78 to 50·05)	36·46 (25·17 to 50·13)	0·98 (-9·67 to 13·07)
**Diarrheal diseases**	22351·89 (14801·91 to 30542·27)	14570·68 (8174·76 to 25693·22)	75·5 (49·29 to 107·7)	20·9 (13·09 to 34·78)	-72·31 (-79·42 to -58·39)	14629·99 (9924·48 to 20467·69)	27492·18 (18807·64 to 37253·09)	36·08 (24·76 to 50·01)	36·44 (25·15 to 50·09)	0·98 (-9·67 to 13·07)
**Typhoid and paratyphoid**	2·1 (0·52 to 5·94)	1·5 (0·39 to 3·97)	0·01 (0 to 0·01)	0 (0 to 0·01)	-10·6 (-31·68 to 9·92)	0·02 (0·01 to 0·04)	0·03 (0·01 to 0·05)	0 (0 to 0)	0 (0 to 0)	-1·08 (-18·1 to 15·89)

**Typhoid fever**	0·67 (0·15 to 1·96)	0·53 (0·13 to 1·41)	0 (0 to 0)	0 (0 to 0)	-3·57 (-34·67 to 38·82)	0·01 (0 to 0·02)	0·01 (0·01 to 0·03)	0 (0 to 0)	0 (0 to 0)	6·17 (-20·6 to 43·35)
**Paratyphoid fever**	1·42 (0·32 to 4·2)	0·98 (0·24 to 2·92)	0 (0 to 0·01)	0 (0 to 0·01)	-13·82 (-35·09 to 8·31)	0·01 (0·01 to 0·02)	0·01 (0·01 to 0·02)	0 (0 to 0)	0 (0 to 0)	-7·77 (-24·92 to 11·67)
**Invasive non-typhoidal Salmonella (iNTS)**	462·56 (214·93 to 861·96)	284·02 (127·23 to 532·31)	1·1 (0·52 to 2·01)	0·65 (0·28 to 1·26)	-41·01 (-50·36 to -31·75)	0·41 (0·05 to 1·19)	0·44 (0·05 to 1·22)	0 (0 to 0)	0 (0 to 0)	-0·1 (-0·58 to 0·35)
**Other intestinal infectious diseases**	233·16 (116·68 to 369·85)	41·06 (25·34 to 61·6)	0·59 (0·27 to 0·94)	0·1 (0·05 to 0·16)	-83·47 (-92·89 to -60·44)	10·41 (7·03 to 14·57)	14·05 (9·66 to 19·42)	0·02 (0·02 to 0·03)	0·02 (0·02 to 0·03)	-1·57 (-13·27 to 11·59)
**Neglected tropical diseases and malaria**	1261·57 (1065·45 to 1786·18)	1437·89 (356·8 to 2033·4)	3·1 (2·63 to 4·37)	1·82 (0·55 to 2·47)	-41·33 (-82·72 to -13·5)	32868·94 (22526·19 to 44866·61)	32461·49 (21609·52 to 45838·48)	80·23 (55·39 to 109)	47·46 (31·92 to 66·53)	-40·84 (-51·97 to -27·16)
**Malaria**	614·24 (531·86 to 711·17)	52·52 (44·48 to 61·17)	1·35 (1·18 to 1·55)	0·08 (0·07 to 0·1)	-93·9 (-94·67 to -92·95)	1512·09 (1092·18 to 2061·39)	40·31 (15·95 to 87·49)	3·24 (2·33 to 4·41)	0·09 (0·04 to 0·2)	-97·14 (-98·82 to -94·12)
**Chagas disease**	0 (0 to 0)	0 (0 to 0)	0 (0 to 0)	0 (0 to 0)	NA	0 (0 to 0)	0 (0 to 0)	0 (0 to 0)	0 (0 to 0)	0 (0 to 0)
**Leishmaniasis**	0 (0 to 0)	0 (0 to 0)	0 (0 to 0)	0 (0 to 0)	NA	0 (0 to 0)	0 (0 to 0)	0 (0 to 0)	0 (0 to 0)	0 (0 to 0)
**Visceral leishmaniasis**	0 (0 to 0)	0 (0 to 0)	0 (0 to 0)	0 (0 to 0)	NA	0 (0 to 0)	0 (0 to 0)	0 (0 to 0)	0 (0 to 0)	0 (0 to 0)
**Cutaneous and mucocutaneous leishmaniasis**	NA	NA	NA	NA	NA	0 (0 to 0)	0 (0 to 0)	0 (0 to 0)	0 (0 to 0)	0 (0 to 0)
**African trypanosomiasis**	0 (0 to 0)	0 (0 to 0)	0 (0 to 0)	0 (0 to 0)	NA	0 (0 to 0)	0 (0 to 0)	0 (0 to 0)	0 (0 to 0)	0 (0 to 0)
**Schistosomiasis**	0 (0 to 0)	0 (0 to 0)	0 (0 to 0)	0 (0 to 0)	NA	0 (0 to 0)	0 (0 to 0)	0 (0 to 0)	0 (0 to 0)	0 (0 to 0)
**Cysticercosis**	37·85 (18·09 to 59·07)	10·34 (5·18 to 16·82)	0·09 (0·04 to 0·13)	0·01 (0·01 to 0·02)	-82·88 (-92·09 to -62·82)	5933·16 (2830·29 to 10289·86)	8591·49 (3435·14 to 17393·15)	17·37 (8·65 to 30·32)	10·06 (4·09 to 20·72)	-42·07 (-76·08 to 25·76)
**Cystic echinococcosis**	93·96 (52·77 to 137·38)	12·68 (6·78 to 19·46)	0·24 (0·14 to 0·36)	0·02 (0·01 to 0·03)	-92·78 (-96·33 to -86·04)	4·73 (2·64 to 7·44)	6·3 (3·64 to 9·76)	0·01 (0·01 to 0·02)	0·01 (0·01 to 0·02)	-3·64 (-15·83 to 6·13)
**Lymphatic filariasis**	7·94 (0·69 to 13·58)	1·71 (0·53 to 2·98)	0·03 (0 to 0·05)	0 (0 to 0)	NA	0 (0 to 0)	0 (0 to 0)	0 (0 to 0)	0 (0 to 0)	0 (0 to 0)
**Onchocerciasis**	NA	NA	NA	NA	NA	0 (0 to 0)	0 (0 to 0)	0 (0 to 0)	0 (0 to 0)	0 (0 to 0)

**Trachoma**	NA	NA	NA	NA	NA	0 (0 to 0)	0 (0 to 0)	0 (0 to 0)	0 (0 to 0)	0 (0 to 0)
**Dengue**	NA	NA	NA	NA	-91·35 (-95·79 to 4·78)	0 (0 to 0)	0 (0 to 0)	0 (0 to 0)	0 (0 to 0)	0 (0 to 0)
**Yellow fever**	0 (0 to 0)	0 (0 to 0)	0 (0 to 0)	0 (0 to 0)	NA	0 (0 to 0)	0 (0 to 0)	0 (0 to 0)	0 (0 to 0)	0 (0 to 0)
**Rabies**	50·53 (1·24 to 75·91)	3·47 (0·59 to 5·99)	0·13 (0 to 0·2)	0 (0 to 0·01)	-96·8 (-98·19 to -61·65)	0·01 (0 to 0·01)	0 (0 to 0)	0 (0 to 0)	0 (0 to 0)	-95·69 (-97·9 to -10·7)
**Intestinal nematode infections**	83·93 (29·7 to 162·45)	6·58 (3·28 to 10·39)	0·22 (0·08 to 0·41)	0·01 (0·01 to 0·03)	-93·18 (-97·69 to -76·83)	1524·22 (847·82 to 2542·75)	679·14 (357·15 to 1184·5)	2·84 (1·57 to 4·72)	1·31 (0·69 to 2·28)	-53·64 (-73·97 to -17·51)
**Ascariasis**	83·93 (29·7 to 162·45)	6·58 (3·28 to 10·39)	0·22 (0·08 to 0·41)	0·01 (0·01 to 0·03)	-93·18 (-97·69 to -76·83)	0 (0 to 0)	0 (0 to 0)	0 (0 to 0)	0 (0 to 0)	-98·47 (-99·23 to -97·07)
**Trichuriasis**	NA	NA	NA	NA	NA	0 (0 to 0)	0 (0 to 0)	0 (0 to 0)	0 (0 to 0)	26496·63 (14555·5 to 50190·67)
**Hookworm disease**	NA	NA	NA	NA	NA	1524·22 (847·81 to 2542·75)	679·13 (357·15 to 1184·49)	2·84 (1·57 to 4·72)	1·31 (0·69 to 2·28)	-53·64 (-73·97 to -17·51)
**Food-borne trematodiases**	NA	NA	NA	NA	NA	12424·16 (8330·23 to 17462·58)	19453·03 (12916·88 to 27948·86)	30·83 (20·8 to 43·25)	26·99 (17·99 to 38·54)	-12·47 (-23·37 to -1·3)
**Leprosy**	NA	NA	NA	NA	NA	12·39 (7·51 to 18·98)	16·07 (9·88 to 24·03)	0·03 (0·02 to 0·05)	0·02 (0·01 to 0·03)	-36·89 (-42·54 to -29·27)
**Ebola virus disease**	0 (0 to 0)	0 (0 to 0)	0 (0 to 0)	0 (0 to 0)	NA	0 (0 to 0)	0 (0 to 0)	0 (0 to 0)	0 (0 to 0)	0 (0 to 0)
**Zika virus disease**	0 (0 to 0)	0 (0 to 0)	0 (0 to 0)	0 (0 to 0)	NA	0 (0 to 0)	0 (0 to 0)	0 (0 to 0)	0 (0 to 0)	0 (0 to 0)
**Guinea worm disease**	NA	NA	NA	NA	NA	0 (0 to 0)	0 (0 to 0)	0 (0 to 0)	0 (0 to 0)	0 (0 to 0)
**Other neglected tropical diseases**	373·11 (253·42 to 910·97)	1350·59 (266·21 to 1951·41)	1·04 (0·73 to 2·35)	1·68 (0·42 to 2·34)	62·2 (-70·57 to 187·95)	11458·18 (7437·31 to 17004·23)	3675·15 (2272·65 to 5845·57)	25·91 (16·88 to 38·47)	8·98 (5·43 to 14·63)	-65·36 (-73·68 to -54·17)
**Other infectious diseases**	84054·45 (53076·78 to 145231·32)	15588·13 (11571·17 to 19490·66)	230·55 (140·26 to 408·34)	35·4 (25·12 to 51·23)	-84·65 (-92·28 to -72·27)	22617·11 (15684·82 to 31387·13)	14937·86 (10127·7 to 20814·95)	50·58 (34·87 to 70·47)	27·21 (18·53 to 38·2)	-46·2 (-51·31 to -40·03)
**Meningitis**	31906·6 (28046·26 to 35567·32)	2013·77 (1791·09 to 2286·1)	84·34 (73·94 to 95·17)	4·12 (3·58 to 4·93)	-95·11 (-96 to -93·84)	5597·19 (3814·24 to 7641)	3220·36 (2179·16 to 4423·12)	12·12 (8·31 to 16·58)	5·64 (3·83 to 7·7)	-53·47 (-62·43 to -43·33)

**Encephalitis**	3924·57 (3080·29 to 6770·4)	5966·74 (4149·63 to 6958·9)	9·88 (7·73 to 17·06)	12·68 (8·69 to 15·32)	28·37 (-35·72 to 84·98)	1049·97 (718·01 to 1437·86)	1094·88 (759·67 to 1493·79)	2·27 (1·55 to 3·11)	1·89 (1·3 to 2·58)	-16·98 (-24·37 to -9·35)
**Diphtheria**	253·78 (139·69 to 417·97)	13·38 (8·07 to 20·84)	0·62 (0·34 to 1·04)	0·03 (0·02 to 0·05)	-94·75 (-97·4 to -89·2)	0·11 (0·06 to 0·19)	0·01 (0 to 0·01)	0 (0 to 0)	0 (0 to 0)	-93·59 (-96·59 to -87·61)
**Whooping cough**	34162·01 (3517·01 to 97565·72)	1769·05 (198·03 to 5104·36)	99·35 (10·24 to 283·71)	8·08 (0·9 to 23·34)	-91·87 (-99·23 to -13·07)	898·13 (506·27 to 1448·32)	145·01 (78·47 to 238·62)	2·61 (1·48 to 4·21)	0·66 (0·36 to 1·08)	-74·88 (-80·41 to -68·84)
**Tetanus**	1953·67 (1479·53 to 2634·93)	304·5 (270·45 to 349·09)	5·64 (4·27 to 7·52)	0·71 (0·63 to 0·82)	-87·39 (-90·77 to -82·65)	1·64 (0·99 to 2·5)	0·38 (0·22 to 0·63)	0 (0 to 0·01)	0 (0 to 0)	-85·01 (-89·48 to -79·55)
**Measles**	336·09 (233·08 to 474·55)	10·03 (5·59 to 16·36)	0·95 (0·66 to 1·35)	0·04 (0·02 to 0·07)	-95·31 (-96·91 to -93·42)	8·61 (5·79 to 12·24)	0·85 (0·56 to 1·21)	0·02 (0·02 to 0·03)	0 (0 to 0·01)	-84·63 (-86·43 to -82·92)
**Varicella and herpes zoster**	1653·37 (1074·97 to 2494·78)	303·56 (216·36 to 411·46)	4·96 (3·27 to 7·37)	0·75 (0·48 to 1·13)	-84·87 (-91·67 to -72·67)	1411·03 (778·11 to 2305·03)	2995·68 (1739·56 to 4726·86)	3·59 (2·05 to 5·74)	4·22 (2·46 to 6·55)	17·61 (-1·25 to 64·26)
**Acute hepatitis**	6037·01 (5453·59 to 6696·55)	884·53 (766·44 to 1041·62)	14·61 (13·07 to 16·37)	1·43 (1·22 to 1·72)	-90·18 (-92·04 to -87·49)	2782·76 (1816·23 to 4072·41)	2751·52 (1759·11 to 4027·96)	5·92 (3·87 to 8·69)	4·7 (3·03 to 6·86)	-20·55 (-32·68 to -7·79)
**Acute hepatitis A**	1415·78 (896·65 to 2163·27)	199·08 (44·42 to 294·02)	3·21 (2·08 to 5·18)	0·43 (0·1 to 0·64)	-86·74 (-97·21 to -75·15)	1468·7 (927·69 to 2173·99)	1302·43 (819·53 to 1929·32)	3·03 (1·9 to 4·48)	2·81 (1·75 to 4·17)	-7·17 (-20·9 to 9·62)
**Acute hepatitis B**	3311·9 (2688·02 to 4131·73)	598·98 (491·31 to 758·18)	8·19 (6·63 to 10·13)	0·85 (0·67 to 1·17)	-89·62 (-92·38 to -85·29)	1204·22 (727·25 to 1869·95)	1323·71 (795·87 to 2022·6)	2·64 (1·6 to 4·03)	1·67 (1·01 to 2·55)	-36·65 (-53·6 to -14·35)
**Acute hepatitis C**	1157·38 (517·83 to 1742·75)	80·5 (50·37 to 128·02)	2·78 (1·32 to 4·11)	0·14 (0·08 to 0·22)	-95·14 (-97·3 to -90·02)	28·12 (13·78 to 55·51)	36·31 (17·15 to 72·65)	0·07 (0·04 to 0·14)	0·05 (0·02 to 0·1)	-31·43 (-36·38 to -24·86)
**Acute hepatitis E**	151·95 (21·25 to 351·89)	5·98 (2·7 to 10·76)	0·43 (0·05 to 1·02)	0·02 (0·01 to 0·05)	-94·65 (-98·22 to -76·96)	81·71 (49·11 to 131·57)	89·07 (55·79 to 138·1)	0·18 (0·11 to 0·28)	0·17 (0·11 to 0·27)	-5·12 (-16·32 to 6·51)
**Other unspecified infectious diseases**	3827·35 (3157·14 to 5642·63)	4322·57 (2083·83 to 5275·69)	10·2 (8·49 to 14·92)	7·55 (3·55 to 9·53)	-25·99 (-73·61 to 2·36)	10867·67 (7025·7 to 15966·69)	4729·17 (2916·83 to 7229·13)	24·04 (15·48 to 35·41)	10·1 (6·26 to 15·73)	-57·97 (-65·58 to -48·65)
**Maternal and neonatal disorders**	250961·11 (209783·96 to 312040·7)	50060·21 (42021·37 to 58670·74)	759·5 (632·49 to 945·5)	244·8 (203·98 to 288·15)	-67·77 (-75·29 to -58·92)	62363·55 (45218·78 to 83827·97)	72007·44 (54912·42 to 90680·2)	136·14 (98·65 to 183·22)	151·2 (114·8 to 191·35)	11·06 (-11·84 to 41·53)
**Maternal disorders**	8591·09 (7682·32 to 9712·32)	2106·3 (1735·67 to 2517·5)	15·58 (13·98 to 17·54)	4·07 (3·32 to 4·9)	-73·85 (-79·13 to -67·49)	1283·06 (759·3 to 1933·83)	723·91 (433·27 to 1108·36)	2·24 (1·33 to 3·37)	1·42 (0·85 to 2·17)	-36·66 (-51·53 to -16·51)
**Maternal hemorrhage**	2047·29 (1637·08 to 2535·55)	358·43 (255·48 to 481·16)	3·69 (2·96 to 4·56)	0·7 (0·49 to 0·95)	-81·14 (-87·19 to -71·84)	616·48 (318·45 to 1005·46)	318·95 (171·75 to 541·44)	1·07 (0·56 to 1·75)	0·62 (0·33 to 1·06)	-42·25 (-63·09 to -13·56)
**Maternal sepsis and other maternal infections**	2243·52 (1837·03 to 2718·07)	24·05 (17·64 to 32·34)	4·18 (3·46 to 5·02)	0·05 (0·03 to 0·06)	-98·92 (-99·25 to -98·48)	185·82 (70·83 to 364·5)	71·68 (25·48 to 141·6)	0·32 (0·12 to 0·64)	0·14 (0·05 to 0·29)	-55·59 (-76·3 to -12·37)

**Maternal hypertensive disorders**	2413·35 (2056·91 to 2878·21)	176·59 (131·3 to 226·29)	4·28 (3·65 to 5·08)	0·35 (0·26 to 0·46)	-91·72 (-94·07 to -88·71)	83 (34·96 to 155·96)	53·38 (24·81 to 96·05)	0·15 (0·06 to 0·27)	0·1 (0·05 to 0·19)	-29·24 (-61·15 to 35·09)
**Maternal obstructed labor and uterine rupture**	86·71 (60·22 to 123·3)	24·75 (16·39 to 35·79)	0·15 (0·11 to 0·22)	0·05 (0·03 to 0·07)	-67·77 (-80·64 to -47·27)	193·45 (65·88 to 390·48)	122·81 (59·18 to 211·44)	0·34 (0·12 to 0·68)	0·24 (0·12 to 0·42)	-28·78 (-59·7 to 46·72)
**Maternal abortion and miscarriage**	769·92 (586·25 to 998·3)	20·12 (14·67 to 27·6)	1·43 (1·09 to 1·84)	0·04 (0·03 to 0·05)	-97·4 (-98·27 to -96·1)	84·69 (22·37 to 181·81)	39·86 (20·64 to 67·18)	0·15 (0·04 to 0·32)	0·08 (0·04 to 0·13)	-45·5 (-69·86 to 80·58)
**Ectopic pregnancy**	116·31 (90·96 to 145·12)	29·3 (21·02 to 39·32)	0·21 (0·16 to 0·26)	0·06 (0·04 to 0·08)	-71·98 (-80·94 to -58·36)	19·95 (10·05 to 33·12)	10·78 (5·98 to 16·6)	0·03 (0·02 to 0·06)	0·02 (0·01 to 0·03)	-39·3 (-53·84 to -17·18)
**Indirect maternal deaths**	138·92 (108·45 to 177·33)	165·57 (119·83 to 223·4)	0·25 (0·19 to 0·31)	0·34 (0·24 to 0·46)	36·14 (-9·62 to 107·07)	NA	NA	NA	NA	NA
**Late maternal deaths**	52·28 (43·12 to 65·21)	44·39 (31·02 to 62·2)	0·1 (0·08 to 0·12)	0·09 (0·06 to 0·12)	-10·36 (-36·42 to 20·62)	NA	NA	NA	NA	NA
**Maternal deaths aggravated by HIV/AIDS**	0·11 (0 to 0·23)	0·21 (0·11 to 0·36)	0 (0 to 0)	0 (0 to 0)	NA	NA	NA	NA	NA	NA
**Other maternal disorders**	722·69 (554·49 to 928·51)	1262·9 (1028·55 to 1529·59)	1·31 (1·01 to 1·67)	2·41 (1·96 to 2·95)	84·41 (32·3 to 155·83)	99·67 (58·99 to 150·17)	106·45 (63·71 to 163·07)	0·17 (0·1 to 0·26)	0·21 (0·12 to 0·32)	19·76 (-8·48 to 57·92)
**Neonatal disorders**	242370·02 (201126·03 to 302677·76)	47953·92 (39815·84 to 56669·89)	743·92 (616·82 to 929·1)	240·72 (199·74 to 284·15)	-67·64 (-75·31 to -58·65)	61080·5 (44187·83 to 82455·58)	71283·52 (54240·15 to 90063·15)	133·91 (96·86 to 180·89)	149·78 (113·92 to 189·54)	11·86 (-11·48 to 43·53)
**Neonatal preterm birth**	146836·06 (119955·23 to 184614·63)	25340·45 (20896·05 to 30227·27)	451·05 (368·21 to 567·64)	127·35 (104·93 to 152·16)	-71·77 (-79·37 to -63·67)	40052·26 (29602·23 to 52687·23)	41092 (31157·07 to 52697·68)	86·68 (64·13 to 113·01)	85·42 (64·31 to 109·32)	-1·46 (-20·54 to 22·86)
**Neonatal encephalopathy due to birth asphyxia and trauma**	37010·81 (27821·78 to 47677·68)	5822·07 (4531·4 to 7281)	113·45 (85·26 to 146·27)	29·16 (22·63 to 36·43)	-74·3 (-81·47 to -63·37)	16467·59 (8247·29 to 28369·82)	25276·77 (18448·04 to 33256·4)	35·8 (18·15 to 61·53)	51·14 (36·87 to 67·41)	42·86 (-8·37 to 158·73)
**Neonatal sepsis and other neonatal infections**	25042·46 (17541·41 to 35498·32)	6821·02 (5344·13 to 8609·18)	76·68 (53·77 to 108·65)	34·15 (26·77 to 43·13)	-55·46 (-70·38 to -30·42)	1664·81 (864·55 to 3343·86)	2636·71 (1458·14 to 4465·78)	3·63 (1·92 to 7·26)	5·17 (2·85 to 8·79)	42·64 (-3·53 to 111·6)
**Hemolytic disease and other neonatal jaundice**	1694·68 (1081·59 to 2562·32)	187·12 (140·8 to 253·05)	5·19 (3·32 to 7·85)	0·92 (0·69 to 1·24)	-82·3 (-89·41 to -69·85)	947·04 (685·95 to 1209·34)	913·19 (675·8 to 1181·58)	2·08 (1·5 to 2·66)	1·82 (1·35 to 2·36)	-12·27 (-28·12 to 8·68)
**Other neonatal disorders**	31786·01 (21962·28 to 44708·21)	9783·26 (7266·09 to 12645·57)	97·54 (67·34 to 137·16)	49·15 (36·45 to 63·49)	-49·62 (-68·62 to -18·98)	1948·8 (1392·17 to 2615·31)	1364·86 (1020·94 to 1762·06)	5·73 (4·1 to 7·69)	6·24 (4·67 to 8·05)	8·83 (-17·96 to 44·02)
**Nutritional deficiencies**	9426·08 (8815·14 to 10047·4)	4152·98 (3571·28 to 4715·2)	37·93 (34·72 to 41·07)	5·47 (4·72 to 6·19)	-85·58 (-87·36 to -83·67)	222568·48 (145489·22 to 329319·42)	83620·39 (53465·83 to 127773·71)	501·51 (332·05 to 742·03)	165·04 (103·91 to 257·2)	-67·09 (-73·13 to -59·96)
**Protein-energy malnutrition**	5865·68 (5307·7 to 6416·15)	1517·93 (1259·75 to 1832·73)	24·26 (21·46 to 26·71)	2·07 (1·73 to 2·48)	-91·48 (-92·92 to -89·55)	5991·31 (3622·21 to 8994·81)	6838 (4255·97 to 10209·99)	14·59 (8·87 to 21·69)	10·78 (6·46 to 16·51)	-26·12 (-42·58 to -0·72)

Iodine deficiency	NA	NA	NA	NA	NA	1698·9 (776·08 to 3295·99)	1552·75 (702·57 to 3023·69)	3·67 (1·69 to 6·94)	2·53 (1·14 to 4·96)	-30·93 (-37·95 to -23·19)
Vitamin A deficiency	NA	NA	NA	NA	NA	724·83 (382·74 to 1186·98)	77·17 (35·84 to 139·77)	1·86 (0·99 to 3·04)	0·33 (0·16 to 0·6)	-82·19 (-91·32 to -67·82)
Dietary iron deficiency	NA	NA	NA	NA	NA	210215·41 (136906·44 to 309356·4)	72418·92 (44958·8 to 111994·89)	472·1 (310·16 to 701·07)	147·1 (90·55 to 236·17)	-68·84 (-75·07 to -61·3)
Other nutritional deficiencies	3560·41 (3080·85 to 4200·37)	2635·04 (2189·13 to 3044·93)	13·68 (11·63 to 16·44)	3·4 (2·85 to 3·89)	-75·11 (-81·31 to -69·14)	3938·03 (2403·3 to 5921·89)	2733·56 (1685·69 to 4134·63)	9·3 (5·67 to 13·89)	4·3 (2·57 to 6·6)	-53·74 (-64·17 to -37·58)
Non-communicable diseases	4994253·1 (4781742·3 to 5073449·83)	4675563·78 (4475835·01 to 4911531·65)	15919·34 (15340·16 to 16147·7)	5638·51 (5400·62 to 5917·79)	-64·58 (-66·19 to -61·64)	3282309·82 (2423469·61 to 4302861·81)	5425562·34 (4033261·79 to 6985212·23)	8083·65 (6008·07 to 10479·15)	7896·05 (5858·24 to 10293·6)	-2·32 (-4·37 to -0·34)
Neoplasms	1467616·89 (1417086·4 to 1510356·52)	2164283·58 (2017993·6 to 2295060·56)	4187·16 (4045·05 to 4378·47)	2525·19 (2356·69 to 2677·06)	-39·69 (-44·11 to -35·61)	26110 (19169·5 to 34066·48)	103498·47 (73963·64 to 139458·14)	77·43 (57·09 to 100·83)	122·48 (87·72 to 165·36)	58·17 (33·5 to 85·4)
Lip and oral cavity cancer	7798 (7345·03 to 8333·19)	17338·69 (15603·56 to 19296·64)	22·94 (21·68 to 24·48)	20·05 (18·13 to 22·29)	-12·57 (-22·58 to -1·16)	156·64 (113·22 to 207·48)	555·74 (375·06 to 778·88)	0·48 (0·35 to 0·64)	0·64 (0·44 to 0·9)	34·06 (6·67 to 69·16)
Nasopharynx cancer	4614·48 (3840·24 to 5408·05)	5410·63 (4721·74 to 6116·85)	12·15 (10·2 to 14·19)	6·51 (5·72 to 7·33)	-46·42 (-57·7 to -32·3)	97·67 (66·39 to 136·3)	703·88 (466·32 to 998·54)	0·27 (0·18 to 0·37)	0·83 (0·56 to 1·17)	209·84 (124·13 to 328·39)
Other pharynx cancer	3221·88 (2949·26 to 3503·32)	11546·28 (9670·88 to 13610·94)	9·46 (8·65 to 10·31)	12·66 (10·59 to 14·85)	33·86 (9·9 to 60·45)	33·23 (22·56 to 44·68)	352·52 (231·9 to 493·88)	0·1 (0·07 to 0·14)	0·39 (0·26 to 0·54)	276·91 (182·1 to 394·32)
Esophageal cancer	45718·95 (31261·37 to 49661·51)	46163·57 (39428·49 to 68818·61)	136·55 (98·24 to 147·24)	50·4 (43·16 to 75·24)	-63·09 (-69·6 to -18·75)	511·22 (323·37 to 717·74)	1226·72 (798 to 1861·36)	1·61 (1·03 to 2·21)	1·34 (0·87 to 2·04)	-16·38 (-40·36 to 54·96)
Stomach cancer	470509·57 (449541·42 to 490409·84)	250946·34 (226275·05 to 276187·45)	1345·16 (1286·46 to 1400·66)	289·42 (262·55 to 317·64)	-78·48 (-80·6 to -75·96)	5122·89 (3691·69 to 6714·64)	8110·88 (5656·05 to 10998·42)	15·42 (11·08 to 20·05)	9·28 (6·46 to 12·56)	-39·81 (-51·72 to -25·6)
Colon and rectum cancer	86781·6 (82948·45 to 90966·44)	228532·87 (207362·38 to 249234·88)	264·04 (251·64 to 277·35)	260·48 (236·44 to 283·52)	-1·35 (-10·82 to 8·78)	2535·43 (1805·75 to 3323·84)	18056·7 (12539·4 to 24571·72)	8·21 (5·88 to 10·71)	20·33 (14·11 to 27·56)	147·47 (101·42 to 199·56)
Liver cancer	104612·32 (85819·24 to 127838·86)	342730·14 (304792·96 to 383579·75)	294·94 (243·49 to 359·54)	384·88 (342·95 to 429·22)	30·5 (2·22 to 66·96)	802·47 (539·21 to 1107·63)	5283·91 (3633·05 to 7291·95)	2·45 (1·64 to 3·4)	5·93 (4·11 to 8·18)	142·19 (77·48 to 229·06)
Liver cancer due to hepatitis B	72923·52 (58103·1 to 91565·62)	207307·18 (170612·23 to 246097·03)	196·36 (157·05 to 244·82)	233·72 (193·5 to 275·58)	19·03 (-8·34 to 55·53)	528·3 (347·57 to 733·78)	2954·72 (1913 to 4207·06)	1·51 (0·98 to 2·11)	3·33 (2·18 to 4·73)	121·18 (53·26 to 208·33)
Liver cancer due to hepatitis C	11607·2 (7476·49 to 16810·85)	49827·03 (33502·82 to 67662·31)	38·89 (25·82 to 55·06)	55 (37·52 to 74·04)	41·42 (7·89 to 82·92)	108·45 (60·04 to 169·24)	902·27 (531·13 to 1428·67)	0·41 (0·23 to 0·62)	1 (0·59 to 1·57)	147·09 (74·09 to 238·3)
Liver cancer due to alcohol use	12287·88 (7758·26 to 18755·52)	58696·04 (39268·38 to 82429·57)	36·54 (23·36 to 54·46)	64·76 (44·04 to 90·18)	77·23 (35·6 to 131·54)	102·9 (56·12 to 166·9)	944·72 (545·03 to 1493·29)	0·33 (0·18 to 0·54)	1·04 (0·61 to 1·66)	217·25 (126·48 to 337·98)

**Liver cancer due to NASH**	3809·73 (2502·86 to 5484·47)	16223·74 (11009·62 to 23614·88)	12·05 (7·98 to 17·2)	18·06 (12·41 to 26·26)	49·83 (13·22 to 97·31)	34·31 (19·51 to 54·9)	302·89 (177·08 to 484·87)	0·12 (0·07 to 0·19)	0·34 (0·2 to 0·54)	180·52 (102·9 to 291·1)
**Liver cancer due to other causes**	3984 (2804·52 to 5597·86)	10676·14 (7082·21 to 15287·89)	11·1 (7·8 to 15·42)	13·35 (9·4 to 18·24)	20·24 (-8·87 to 56)	28·51 (16·13 to 44·58)	179·31 (105·86 to 280·21)	0·09 (0·05 to 0·14)	0·21 (0·13 to 0·33)	145·58 (79·02 to 233·25)
**Gallbladder and biliary tract cancer**	66523·26 (54264·34 to 78027·72)	99680·91 (77495·47 to 118749·08)	213·79 (176·09 to 261·25)	111·43 (87·04 to 132·33)	-47·88 (-61·97 to -24·25)	666·17 (444·55 to 899·32)	1595·94 (1048·59 to 2289·03)	2·33 (1·57 to 3·15)	1·8 (1·18 to 2·59)	-22·72 (-49·93 to 10·18)
**Pancreatic cancer**	58362·5 (55671·66 to 61449·73)	146000·68 (133305·29 to 160753·85)	179·51 (171·49 to 188·96)	162·53 (147·93 to 179·27)	-9·46 (-18·06 to 0·2)	468·23 (324·83 to 630·2)	1639·3 (1118·1 to 2273·37)	1·57 (1·09 to 2·12)	1·84 (1·26 to 2·55)	17·07 (-7·38 to 46·93)
**Larynx cancer**	18764·7 (17139·47 to 20006·87)	10174 (8888·08 to 11919·51)	57·55 (53·18 to 61·1)	11·27 (9·85 to 13·18)	-80·41 (-83·15 to -73·56)	816·15 (569·7 to 1093·03)	1487·45 (976·57 to 2099·45)	2·49 (1·74 to 3·32)	1·67 (1·1 to 2·33)	-33·16 (-48·66 to -9·86)
**Tracheal, bronchus, and lung cancer**	194987·07 (186754·19 to 204111·86)	439405·74 (399078·99 to 479161·37)	584·13 (559·42 to 614·72)	488·61 (443·47 to 533·21)	-16·35 (-24·53 to -7·4)	1805·62 (1291·38 to 2395·7)	8770·88 (6055·44 to 11978·9)	5·72 (4·09 to 7·55)	9·76 (6·77 to 13·33)	70·75 (30·71 to 116·51)
**Malignant skin melanoma**	2445·91 (1875·11 to 4688·58)	5712·97 (2567·97 to 7274·06)	6·62 (5·14 to 12·45)	7·04 (3·33 to 8·87)	6·31 (-64·91 to 63·91)	61·4 (35·93 to 119·65)	697·28 (288·56 to 1121·58)	0·16 (0·1 to 0·32)	0·88 (0·39 to 1·41)	437·92 (67·29 to 817·96)
**lanoma skin cancer**	5593·77 (5247·45 to 5926·78)	6318·52 (5611·58 to 7030·24)	20·22 (18·72 to 21·59)	7·68 (6·79 to 8·55)	-62·03 (-66·11 to -57·34)	13·68 (8·72 to 20·17)	46·65 (29·62 to 68·83)	0·06 (0·04 to 0·09)	0·06 (0·04 to 0·08)	-13·01 (-25·06 to 1·3)
**Non-melanoma skin cancer (squamous-cell carcinoma)**	5593·77 (5247·45 to 5926·78)	6318·52 (5611·58 to 7030·24)	20·22 (18·72 to 21·59)	7·68 (6·79 to 8·55)	-62·03 (-66·11 to -57·34)	13·09 (8·31 to 19·17)	43·94 (27·42 to 65·71)	0·06 (0·04 to 0·09)	0·05 (0·03 to 0·08)	-15·22 (-27·19 to -1·24)
**Non-melanoma skin cancer (basal-cell carcinoma)**	48680 (46134·51 to 51531·71)	107403·34 (95352·54 to 119371·73)	128·26 (121·61 to 135·69)	125·39 (111·56 to 138·92)	-2·24 (-15·26 to 10·32)	0·59 (0·27 to 1·09)	2·71 (1·24 to 5·03)	0 (0 to 0)	0 (0 to 0·01)	55·31 (42·84 to 68·64)
**Breast cancer**	39661·85 (33109·64 to 46628·92)	30883·74 (25161·01 to 39138·36)	102·89 (89·76 to 128·07)	37·48 (30·66 to 47·11)	-63·57 (-69·91 to -54·03)	2897·61 (1939·05 to 4027·45)	15556·27 (10233·94 to 22522·78)	8·26 (5·52 to 11·62)	18·17 (11·97 to 26·31)	120·05 (77·72 to 171·41)
**Cervical cancer**	13913·55 (6114·95 to 16186·88)	8807·56 (6234·18 to 10709·69)	40·18 (18·81 to 46·5)	9·9 (7·02 to 11·89)	-75·36 (-80·24 to -39·29)	1462·31 (943·42 to 2057·55)	1789·27 (1165·9 to 2651·22)	3·58 (2·38 to 5·03)	2·3 (1·49 to 3·48)	-35·63 (-53·35 to -8·96)
**Uterine cancer**	11351·71 (9882·66 to 18715·99)	34167·45 (20183·54 to 39771·9)	30·34 (26·11 to 51·98)	39·92 (24·27 to 46·19)	31·58 (-60·32 to 70·42)	645·33 (235·08 to 961·71)	1072·65 (632·53 to 1637·13)	1·73 (0·66 to 2·53)	1·23 (0·73 to 1·88)	-28·65 (-49·48 to 64·78)
**Ovarian cancer**	12347·62 (10766·71 to 16156·66)	41512·81 (32309·01 to 52638·1)	46·8 (41·14 to 62·14)	46·55 (36·29 to 58·7)	-0·54 (-38·48 to 14·63)	299·18 (198·59 to 485·26)	1420·04 (796·11 to 2110·9)	0·82 (0·53 to 1·38)	1·7 (0·97 to 2·5)	107·37 (-33·86 to 212·89)
**Prostate cancer**	NA	NA	NA	NA		645·47 (441·77 to 900·48)	7297·31 (4825·1 to 10499·91)	2·46 (1·69 to 3·42)	8·05 (5·36 to 11·58)	227·59 (111·14 to 325·81)
**Testicular cancer**	812·05 (641·88 to 1027·16)	649·19 (462·96 to 875·18)	1·76 (1·43 to 2·17)	1·25 (0·87 to 1·67)	-29·14 (-53·38 to 2·21)	43·6 (20 to 85·59)	215·98 (106·53 to 379·84)	0·1 (0·04 to 0·2)	0·5 (0·23 to 0·89)	408·11 (103·17 to 1125·39)

**Kidney cancer**	8794·33 (8199·64 to 9354·41)	33210·03 (29495·49 to 37541·68)	25·83 (24·14 to 27·51)	39·13 (34·88 to 43·76)	51·49 (31·91 to 75·06)	183·79 (128·06 to 246·19)	1428·9 (951·58 to 2019·79)	0·54 (0·38 to 0·72)	1·72 (1·15 to 2·43)	219·71 (148·81 to 310·96)
**Bladder cancer**	13997·2 (13135·29 to 15047·03)	30799·67 (27292·34 to 34450·92)	50·67 (47·45 to 55·33)	34·81 (30·86 to 38·93)	-31·3 (-38·98 to -21·83)	687·94 (484·19 to 932·94)	3658·32 (2463·49 to 5034·81)	2·34 (1·66 to 3·13)	4·1 (2·78 to 5·67)	75·14 (39·71 to 116·85)
**Brain and central nervous system cancer**	43127·29 (31037·96 to 60558·77)	43566·61 (28236·2 to 52200·46)	106·55 (77·23 to 148·08)	67·83 (45·59 to 82·11)	-36·34 (-68·9 to -7·46)	582·92 (368·4 to 814·26)	2583·44 (1438·24 to 3609·85)	1·57 (0·99 to 2·2)	3·6 (2·12 to 5·06)	128·71 (18·27 to 232·06)
**Thyroid cancer**	3650·12 (3044·56 to 7772·22)	11169·83 (8015·32 to 13201·62)	11·47 (9·5 to 25·22)	13·17 (9·35 to 15·35)	14·79 (-61·8 to 50·57)	335·78 (199·56 to 729·97)	2609·52 (1483·35 to 3842·01)	0·87 (0·53 to 1·91)	3·39 (1·85 to 5·02)	288·84 (17·96 to 496·71)
**Mesothelioma**	1314·11 (1054·9 to 1806·18)	2888·78 (2374·31 to 3457·21)	3·73 (3·02 to 5·03)	3·33 (2·72 to 3·96)	-10·52 (-42·58 to 20·35)	22·27 (14·34 to 33·89)	60·67 (38·99 to 87·73)	0·07 (0·05 to 0·11)	0·07 (0·04 to 0·1)	-3·33 (-41·16 to 35·15)
**Hodgkin lymphoma**	1408·72 (1052·96 to 2345·8)	1686·03 (947·25 to 2158·15)	3·36 (2·54 to 5·54)	2·38 (1·38 to 3·04)	-29·16 (-68·03 to 12·43)	98·49 (56·64 to 169·14)	1107·21 (545·73 to 1736·63)	0·3 (0·17 to 0·52)	1·38 (0·7 to 2·15)	356·96 (89·83 to 706·83)
**Non-Hodgkin lymphoma**	28485·98 (26805·79 to 30015·17)	51148 (46608·17 to 56132·93)	71·05 (67·27 to 74·76)	64·73 (59·03 to 70·9)	-8·9 (-18·54 to 1·21)	519·25 (365·81 to 708·52)	4079·6 (2773·3 to 5578·17)	1·3 (0·91 to 1·78)	4·9 (3·36 to 6·68)	276·98 (187·46 to 391·73)
**Multiple myeloma**	7961·46 (5985·7 to 13496·9)	23031·21 (12958·92 to 27087·25)	24·97 (18·62 to 43)	25·49 (14·42 to 29·97)	2·05 (-62·95 to 54·11)	189·64 (112·35 to 337·34)	1050·64 (507·14 to 1595·11)	0·64 (0·37 to 1·17)	1·16 (0·56 to 1·75)	81·31 (-36·79 to 219·49)
**Leukemia**	90211·49 (72354·43 to 100108·52)	56514·15 (42097·45 to 66908·94)	209·5 (170·77 to 235·08)	91·14 (68·28 to 109·45)	-56·49 (-68·35 to -39·04)	1652·82 (1030·85 to 2333·53)	3531·3 (2181·35 to 5173·27)	4·18 (2·73 to 5·87)	5·48 (3·41 to 8·11)	31·12 (-11·98 to 105·29)
**Acute lymphoid leukemia**	16888·32 (8543·28 to 23395·83)	9322·7 (6107·93 to 11547·62)	37·87 (18·92 to 52·02)	20·48 (14·36 to 26·9)	-45·92 (-69·3 to 29·88)	143·92 (74·66 to 221·1)	1927·65 (1058·18 to 2890·86)	0·32 (0·17 to 0·5)	3·18 (1·8 to 4·85)	886·59 (420·48 to 1851·92)
**Chronic lymphoid leukemia**	508·34 (394·96 to 844·71)	1290·47 (878·01 to 1571·61)	1·43 (1·1 to 2·4)	1·57 (1·09 to 1·89)	9·65 (-51·89 to 54·35)	18·42 (11·42 to 31·87)	216·7 (126·09 to 318·12)	0·06 (0·03 to 0·1)	0·25 (0·15 to 0·37)	349·62 (89·5 to 599·21)
**Acute myeloid leukemia**	19484·49 (12267·4 to 25374·51)	26654·98 (17280·06 to 32009·63)	44·3 (29·76 to 57·54)	40·7 (26·33 to 49·2)	-8·14 (-41·41 to 26·36)	141·95 (88·22 to 214·59)	298·61 (169·26 to 442·34)	0·34 (0·21 to 0·5)	0·41 (0·23 to 0·62)	23·27 (-27·99 to 72·46)
**Chronic myeloid leukemia**	7829·15 (6011·98 to 13112·59)	3968·88 (2729·43 to 4920·13)	17·44 (13·41 to 29·61)	5·53 (3·83 to 6·91)	-68·29 (-85·06 to -56·04)	55·07 (35·61 to 87·76)	407·98 (241·21 to 616·85)	0·13 (0·08 to 0·21)	0·54 (0·32 to 0·81)	308·93 (89·64 to 539·35)
**Other leukemia**	45501·19 (25724·35 to 55854·24)	15277·12 (11748·04 to 20824·54)	108·46 (65·32 to 136·63)	22·87 (17·44 to 31·64)	-78·92 (-85·78 to -54·29)	1293·46 (702·25 to 1863·27)	680·37 (429·01 to 1033·15)	3·34 (1·94 to 4·82)	1·1 (0·68 to 1·7)	-66·94 (-78·82 to -27·75)
**Other malignant neoplasms**	68899·06 (55152·15 to 74659·24)	62267·21 (55613·99 to 70422·9)	173·74 (141·02 to 188)	91·75 (82·11 to 104·72)	-47·19 (-54·05 to -27·13)	1040·34 (718·65 to 1382·69)	2891·26 (2015·24 to 3933·53)	2·66 (1·86 to 3·57)	4·34 (3·01 to 5·91)	62·94 (27·7 to 110·48)
**Other neoplasms**	3066·32 (2456·8 to 4298·85)	14616·62 (9359·57 to 17822·77)	9·01 (7·3 to 12·97)	17·98 (11·46 to 21·9)	99·46 (11·09 to 178·01)	1712·5 (1091·81 to 2549·66)	4618·25 (2825·05 to 6900·44)	5·13 (3·25 to 7·67)	5·63 (3·54 to 8·37)	9·67 (-0·47 to 21·43)

Myelodysplastic, myeloproliferative, and other haematopoietic neoplasms	3066·32 (2456·8 to 4298·85)	14616·62 (9359·57 to 17822·77)	9·01 (7·3 to 12·97)	17·98 (11·46 to 21·9)	99·46 (11·09 to 178·01)	1712·5 (1091·81 to 2549·66)	4618·25 (2825·05 to 6900·44)	5·13 (3·25 to 7·67)	5·63 (3·54 to 8·37)	9·67 (-0·47 to 21·43)
Benign and in situ intestinal neoplasms	NA	NA	NA	NA	NA	0 (0 to 0)	0 (0 to 0)	0 (0 to 0)	0 (0 to 0)	0 (0 to 0)
Benign and in situ cervical and uterine neoplasms	NA	NA	NA	NA	NA	0 (0 to 0)	0 (0 to 0)	0 (0 to 0)	0 (0 to 0)	0 (0 to 0)
Other benign and in situ neoplasms	NA	NA	NA	NA	NA	0 (0 to 0)	0 (0 to 0)	0 (0 to 0)	0 (0 to 0)	0 (0 to 0)
Cardiovascular diseases	1953775·55 (1752503·88 to 2032686·71)	1152672·9 (1029917·72 to 1373739·88)	6862·19 (6173·54 to 7146·7)	1365·99 (1214·58 to 1623·85)	-80·09 (-82·1 to -73·63)	157354·68 (115123·15 to 198170·53)	256530·5 (185678·24 to 326509·22)	531·89 (389·84 to 667·71)	300·58 (217·3 to 383·11)	-43·49 (-46·58 to -40·05)
Rheumatic heart disease	9840·1 (9003·95 to 10884·09)	5523·62 (4628·17 to 6480·74)	27·74 (25·36 to 30·66)	6·8 (5·73 to 7·98)	-75·48 (-79·41 to -70·35)	564·28 (364·95 to 814·84)	1129·56 (726·38 to 1639·19)	1·62 (1·05 to 2·31)	1·4 (0·89 to 2·03)	-13·71 (-26·05 to -1·49)
Ischemic heart disease	722036·91 (666742·43 to 754599·8)	422624·51 (373375·46 to 481840·46)	2510·7 (2351·88 to 2628·39)	497·43 (438·74 to 568·12)	-80·19 (-82·34 to -76·15)	6929·52 (4572·15 to 9858·76)	17940·53 (11907·39 to 25509·42)	24·83 (16·6 to 35·1)	20·42 (13·6 to 28·88)	-17·76 (-23·81 to -10·98)
Stroke	1057447·62 (914874·95 to 1113572·72)	554132·48 (495536·24 to 698343·16)	3759·7 (3236·77 to 3959·36)	648·47 (577 to 809·62)	-82·75 (-84·66 to -75·01)	127812·69 (92028·99 to 161882·25)	181398·23 (130195·99 to 233868·21)	432·69 (312·24 to 546·6)	211·94 (151·74 to 273·3)	-51·02 (-54·17 to -47·44)
Ischemic stroke	348165·61 (273530·22 to 392132·13)	282029·2 (243427·34 to 358083·22)	1485·44 (1185·25 to 1664·02)	329·58 (282·85 to 414·75)	-77·81 (-81·42 to -66·31)	86495·37 (62611·58 to 109627·73)	131585·5 (94008·02 to 172515·49)	306·79 (223·62 to 389·81)	153·35 (109·39 to 201·16)	-50·02 (-54·22 to -45·28)
Intracerebral hemorrhage	586653·85 (526908·52 to 633629·74)	198036·09 (175497·25 to 259158·68)	1925·39 (1717·26 to 2078·56)	229·06 (202·99 to 300·04)	-88·1 (-89·66 to -82·48)	27343·7 (19278·43 to 35176·34)	25482·64 (17830·72 to 32969·81)	83·89 (59·24 to 108·83)	30·62 (21·38 to 39·68)	-63·5 (-66·92 to -59·62)
Subarachnoid hemorrhage	122628·16 (91811·97 to 151341·97)	74067·19 (65446·18 to 92228)	348·86 (258·8 to 428·59)	89·84 (79·52 to 111·03)	-74·25 (-79·33 to -63·05)	13973·63 (9745·44 to 18487·98)	24330·09 (17319·46 to 31976·88)	42·01 (29·23 to 56·34)	27·97 (20·03 to 36·77)	-33·41 (-43·9 to -23·62)
Hypertensive heart disease	71116·69 (50424·6 to 77632·23)	55005·9 (43476·33 to 84069·74)	267·87 (204·76 to 294·23)	68·32 (53·89 to 100·56)	-74·5 (-79·7 to -54·48)	2309·69 (1391·95 to 3511·05)	7692·26 (4595·78 to 11518·11)	9·78 (6 to 14·97)	9·12 (5·48 to 13·67)	-6·7 (-18·58 to 7)
Non-rheumatic valvular heart disease	4900·89 (3891·98 to 5811·9)	11726·4 (9961·08 to 13468·77)	16·17 (12·93 to 19·02)	14·09 (11·88 to 16·2)	-12·88 (-32·13 to 14·91)	1375·97 (716·9 to 2386·86)	5470·26 (2984·43 to 9408·23)	5·3 (2·91 to 9·05)	6·22 (3·4 to 10·63)	17·53 (0·38 to 38·23)
Non-rheumatic calcific aortic valve heart disease	2612·51 (1910·96 to 3321·61)	8799·72 (7466·32 to 10307·57)	9·02 (6·65 to 11·48)	10·57 (8·96 to 12·36)	17·11 (-9·92 to 62·14)	264·21 (141·56 to 468·38)	2345·77 (1253·86 to 3912·92)	1·11 (0·61 to 1·91)	2·69 (1·45 to 4·44)	142·42 (86·26 to 225·7)
Non-rheumatic degenerative mitral valve heart disease	1951·51 (1556·31 to 2437·59)	2712·26 (1956·41 to 3344·66)	6·27 (4·98 to 7·86)	3·23 (2·34 to 3·99)	-48·42 (-62·82 to -27·85)	1105·12 (563·32 to 1922·61)	3112·34 (1618·43 to 5481·08)	4·17 (2·21 to 7·21)	3·52 (1·83 to 6·18)	-15·55 (-29·23 to -1·37)
Other non-rheumatic valve heart diseases	336·87 (245·43 to 471·58)	214·43 (129·17 to 290·65)	0·88 (0·62 to 1·16)	0·29 (0·18 to 0·38)	-67·32 (-76·7 to -52·52)	6·63 (4·13 to 9·81)	12·16 (7·9 to 17·7)	0·02 (0·01 to 0·03)	0·02 (0·01 to 0·02)	-13·8 (-22·1 to -4·46)

**Cardiomyopathy and myocarditis**	24433·53 (18211·9 to 38579·38)	23889·51 (16846·21 to 28249·88)	67·95 (49·54 to 100·74)	34·7 (25·32 to 41·25)	-48·93 (-71·18 to -27·89)	1365·14 (859·04 to 2028·38)	2648·11 (1681·22 to 3923·57)	3·73 (2·41 to 5·47)	3·61 (2·3 to 5·32)	-3·38 (-15·36 to 12·25)
**Myocarditis**	5447·91 (3370·11 to 8731·21)	2203·47 (1580·28 to 3083·58)	14·09 (7·62 to 20·48)	4·69 (3·27 to 6·21)	-66·73 (-81·44 to -30·74)	289·74 (185·01 to 433·73)	390·98 (254·17 to 567·38)	0·69 (0·45 to 1·01)	0·67 (0·43 to 0·98)	-3·1 (-10·53 to 5)
**Alcoholic cardiomyopathy**	1026·58 (623·68 to 1697·19)	616·92 (416·61 to 820·94)	2·64 (1·59 to 4·35)	0·83 (0·59 to 1·11)	-68·44 (-83·32 to -44·56)	48·26 (29·42 to 73·82)	90·53 (55·22 to 138·5)	0·12 (0·07 to 0·18)	0·12 (0·07 to 0·18)	-2·09 (-12·21 to 10·91)
**Other cardiomyopathy**	17959·04 (13108·25 to 28764·02)	21069·13 (14846·93 to 24897·92)	51·21 (37·72 to 77·82)	29·17 (21·3 to 34·77)	-43·03 (-67·73 to -19·35)	1027·15 (634·49 to 1538·03)	2166·61 (1335·41 to 3268·1)	2·92 (1·85 to 4·34)	2·82 (1·78 to 4·22)	-3·5 (-17·6 to 14·93)
**Atrial fibrillation and flutter**	7513·83 (6460·77 to 8780·24)	24137·41 (20192·06 to 27487·59)	35·82 (30·72 to 41·58)	28·72 (23·85 to 32·74)	-19·81 (-34·07 to -3·03)	8623·68 (5381·06 to 12605·86)	25317·69 (15833·36 to 37469·8)	30·12 (18·93 to 44·3)	28·1 (17·63 to 41·46)	-6·73 (-13·38 to 0·42)
**Aortic aneurysm**	10417·19 (8446·01 to 12647·47)	24014·19 (20926·96 to 27308·06)	35·4 (29·24 to 43·31)	27·85 (24·31 to 31·63)	-21·32 (-37·67 to -0·97)	NA	NA			
**Peripheral artery disease**	1351·94 (1099·83 to 1738·15)	2634·59 (2158·63 to 3173·83)	5·61 (4·57 to 7·11)	3·07 (2·51 to 3·69)	-45·25 (-61·05 to -28·24)	2087·6 (969·74 to 3811·79)	3975·04 (1868·33 to 7268·54)	8·7 (4·13 to 15·65)	4·46 (2·1 to 8·17)	-48·74 (-52·68 to -44·59)
**Endocarditis**	18002·97 (8358·22 to 23656·82)	10090·34 (7756·5 to 16699·03)	56·03 (24·84 to 74·73)	12·81 (9·85 to 21·24)	-77·13 (-86·04 to -29·47)	172·07 (109·78 to 251·86)	456·09 (294·74 to 665·29)	0·66 (0·42 to 0·98)	0·57 (0·38 to 0·83)	-13·73 (-28·09 to 3·39)
**Other cardiovascular and circulatory diseases**	26713·88 (20540·11 to 31091·93)	18893·95 (16295·26 to 24365·35)	79·21 (62·54 to 90·78)	23·72 (20·33 to 31·95)	-70·05 (-74·58 to -60·27)	6114·03 (3636·35 to 9193·07)	10502·72 (6317·28 to 15594·97)	14·46 (8·7 to 21·6)	14·74 (9·03 to 21·22)	1·93 (-11·27 to 19·03)
**Chronic respiratory diseases**	211325·82 (168724·44 to 225138·7)	203582·78 (174254·25 to 229106·02)	881·63 (650·85 to 956·94)	239·94 (203·1 to 270·08)	-72·78 (-76·15 to -61·86)	111791·5 (82167·42 to 146216·2)	218071·8 (171326·44 to 267539·15)	322·01 (240·62 to 411·09)	319·75 (243·65 to 408·92)	-0·7 (-8·5 to 7·08)
**Chronic obstructive pulmonary disease**	81062·95 (66682·78 to 90705·37)	135071 (115284·17 to 158642·74)	343·07 (266·72 to 388·85)	158·16 (134·57 to 184·56)	-53·9 (-61·75 to -35·74)	41561·02 (33505·46 to 48789·56)	128712·73 (104822·12 to 149576·42)	136·87 (111·04 to 159·42)	155·69 (127·29 to 181·17)	13·75 (7·38 to 20·39)
**Pneumoconiosis**	10458·65 (8002·09 to 13025·19)	7495·75 (6034·72 to 9506·47)	31·96 (24·58 to 39·48)	8·27 (6·65 to 10·52)	-74·12 (-80·97 to -61·85)	337·01 (215·72 to 488·21)	983·17 (639·18 to 1429·54)	1·19 (0·76 to 1·7)	1·1 (0·72 to 1·61)	-7·73 (-28·4 to 21·07)
**Silicosis**	1481·35 (418·76 to 2587·73)	450·91 (192·27 to 2004·88)	4·42 (1·4 to 7·81)	0·49 (0·21 to 2·22)	-88·81 (-96·08 to -44·36)	69·72 (39·25 to 114·61)	252·46 (143·74 to 396·14)	0·27 (0·15 to 0·43)	0·28 (0·16 to 0·45)	5·56 (-36·07 to 65·86)
**Asbestosis**	29·6 (13·3 to 56·57)	134·4 (86·46 to 224·97)	0·1 (0·04 to 0·18)	0·15 (0·1 to 0·24)	50·87 (-19·29 to 265·68)	4·5 (2·23 to 8·06)	13·13 (7·43 to 21·04)	0·01 (0·01 to 0·02)	0·02 (0·01 to 0·03)	20·79 (0·99 to 49·69)
**Coal workers pneumoconiosis**	4173·22 (2532·3 to 5825·76)	5710·3 (2105·7 to 7278·86)	12·71 (7·58 to 17·8)	6·31 (2·36 to 8)	-50·39 (-77·07 to -20·55)	30·87 (19·34 to 46·18)	227·58 (142·9 to 334·47)	0·1 (0·06 to 0·15)	0·25 (0·16 to 0·37)	152·26 (98·64 to 217·09)
**Other pneumoconiosis**	4774·48 (2360·31 to 6824·59)	1200·14 (652·12 to 4494·37)	14·73 (7·6 to 20·98)	1·32 (0·72 to 4·96)	-91·04 (-95·84 to -43·07)	231·92 (140·51 to 343·6)	490 (276·46 to 775·28)	0·81 (0·48 to 1·2)	0·55 (0·31 to 0·87)	-32·35 (-54·09 to 3·49)

**Asthma**	108246·69 (76063·51 to 123379·57)	30188·99 (24323·92 to 37188·16)	473·14 (304·5 to 547·96)	37·19 (30·09 to 45·84)	-92·14 (-93·76 to -87·46)	65188·29 (41365·86 to 96917·72)	73797·58 (47863·8 to 109082·56)	172·46 (111·55 to 253·54)	141·33 (88·79 to 212·88)	-18·05 (-30·25 to -5·33)
**Interstitial lung disease and pulmonary sarcoidosis**	7283·7 (5855·94 to 11179·12)	28226·38 (14948·75 to 36859·81)	23·46 (18·88 to 34·59)	32·74 (18·13 to 42·16)	39·55 (-32·37 to 107·42)	1927·19 (1229·69 to 2857·78)	8959·68 (5920·99 to 12966·16)	5·49 (3·53 to 8·07)	10·33 (6·83 to 14·99)	88·08 (61·11 to 120·29)
**Other chronic respiratory diseases**	4273·83 (2006·36 to 6325·29)	2600·65 (1964·84 to 3986·97)	10 (4·75 to 14·78)	3·58 (2·76 to 5·45)	-64·17 (-80·39 to -16·13)	2777·99 (2112·46 to 3483·16)	5618·64 (4321·81 to 6952·79)	5·99 (4·6 to 7·46)	11·29 (8·56 to 14·42)	88·46 (59·59 to 119·66)
**Digestive diseases**	678671·61 (642880 to 701689·14)	370743·03 (333200·54 to 413198·86)	1880·96 (1796·05 to 1944·61)	439·52 (395·7 to 490)	-76·63 (-78·99 to -74·08)	99985·79 (69582·84 to 136601·72)	141878·48 (98105·2 to 197407·65)	253·06 (177·34 to 345·05)	206·43 (143·26 to 283·05)	-18·43 (-22·79 to -14·19)
**Cirrhosis and other chronic liver diseases**	550728·89 (486742·81 to 575566·39)	273559·61 (244001·8 to 306807·61)	1441·44 (1286·47 to 1502·16)	318·84 (284·48 to 357·52)	-77·88 (-80·49 to -73·43)	6140·58 (4245·71 to 8519·76)	5777·38 (3904·45 to 8112·4)	16·31 (11·31 to 22·6)	7·77 (5·22 to 11·08)	-52·34 (-57·9 to -46·36)
**Cirrhosis and other chronic liver diseases due to hepatitis B**	282031·69 (236291·36 to 327763·03)	99289·99 (77740·29 to 125026·21)	730·02 (616·56 to 844·2)	117·19 (92·17 to 145·91)	-83·95 (-86·18 to -80·54)	2971·81 (2001·65 to 4297·73)	1869·64 (1187·05 to 2766·78)	7·89 (5·29 to 11·29)	2·34 (1·51 to 3·44)	-70·32 (-75·51 to -64·45)
**Cirrhosis and other chronic liver diseases due to hepatitis C**	43372·66 (31393·19 to 58753·13)	25750·66 (17710·18 to 36448·96)	112·37 (81·82 to 151·26)	30·49 (21·14 to 42·27)	-72·87 (-76·84 to -67·19)	459·74 (278·68 to 715·92)	516·8 (289·67 to 818·36)	1·2 (0·73 to 1·88)	0·67 (0·39 to 1·03)	-44·54 (-58·47 to -27·75)
**Cirrhosis and other chronic liver diseases due to alcohol use**	194835·1 (149862·4 to 238732·04)	129816·55 (102282·36 to 158796·43)	518·22 (403·72 to 630·76)	147·99 (117·18 to 179·37)	-71·44 (-75·17 to -65·51)	1941·57 (1237·94 to 2864·23)	2661·39 (1709·7 to 3877·68)	5·27 (3·38 to 7·81)	3·09 (2·01 to 4·49)	-41·45 (-51·7 to -28·68)
**Cirrhosis and other chronic liver diseases due to NAFLD**	9524·63 (6804·65 to 12863·9)	7968·56 (5206·53 to 11946·22)	26·39 (19·24 to 35·53)	9·24 (6·15 to 13·44)	-64·98 (-71·62 to -55·78)	114·24 (68·68 to 174·36)	172·19 (95·73 to 283·46)	0·32 (0·19 to 0·5)	0·21 (0·12 to 0·34)	-35·4 (-44·81 to -23·53)
**Cirrhosis and other chronic liver diseases due to other causes**	20964·81 (16791·48 to 26207·4)	10733·85 (7749·95 to 14263·95)	54·43 (43·06 to 67·86)	13·93 (10·45 to 17·88)	-74·41 (-78·88 to -67·56)	653·22 (372·83 to 1063·67)	557·35 (313·72 to 880·37)	1·62 (0·95 to 2·63)	1·47 (0·78 to 2·43)	-9·41 (-40·94 to 33·81)
**Upper digestive system diseases**	49906·82 (45743·6 to 65683·37)	13896·08 (11506 to 16238·46)	178·46 (163·2 to 224·62)	16·8 (13·97 to 19·72)	-90·58 (-93·07 to -88·75)	29442·74 (16970·2 to 48610·91)	46564·89 (26163·55 to 79606·34)	71·05 (41·34 to 116·67)	65·06 (37·04 to 110·73)	-8·44 (-13·06 to -5·06)
**Peptic ulcer disease**	41191·54 (36813·35 to 59131·3)	12838·64 (10475·45 to 14964·47)	141·98 (125·83 to 197·21)	15·43 (12·66 to 17·97)	-89·13 (-93·01 to -86·91)	2675·89 (1742·27 to 3806·82)	3784·79 (2379·99 to 5441·19)	7·24 (4·81 to 10·11)	5·26 (3·33 to 7·64)	-27·24 (-37·39 to -15·67)
**Gastritis and duodenitis**	8715·28 (5279·87 to 10972·95)	1057·45 (757·07 to 1667·17)	36·48 (20·11 to 46·5)	1·37 (0·98 to 2·09)	-96·24 (-97·66 to -84·46)	4539·49 (2915·76 to 6736·89)	4401 (2718·69 to 6675·12)	11·41 (7·33 to 16·81)	7·28 (4·57 to 11·19)	-36·17 (-44·77 to -26·85)
**Gastroesophageal reflux disease**	NA	NA	NA	NA		22227·37 (11306·14 to 39775·7)	38379·09 (19211·07 to 69923·32)	52·4 (26·58 to 94·47)	52·51 (26·67 to 94·45)	0·2 (-1·64 to 1·9)
**Appendicitis**	8030·51 (6994·71 to 10628·64)	2132·21 (1375·39 to 2750·36)	21·14 (18·65 to 27·59)	3·04 (1·91 to 3·93)	-85·6 (-92·12 to -79·83)	2457·98 (1454·02 to 3802·55)	2241·5 (1355·84 to 3395·07)	5 (3·01 to 7·73)	5·35 (3·16 to 8·4)	6·9 (-19·7 to 43·92)
**Paralytic ileus and intestinal obstruction**	11772·88 (9577·38 to 19722·74)	22387·26 (12642·56 to 27613·8)	39·04 (31·69 to 63·91)	29·16 (17·13 to 35·88)	-25·32 (-70·05 to 6·05)	513·24 (342·69 to 712·88)	1538·03 (1027·31 to 2143·64)	1·41 (0·94 to 1·95)	2·91 (1·97 to 4·01)	106·23 (77·53 to 138·37)

**Inguinal, femoral, and abdominal hernia**	5255·19 (3254·81 to 6226·12)	1779·99 (1270·57 to 3270·91)	18·9 (11·4 to 22·07)	2·23 (1·6 to 4·08)	-88·2 (-91·77 to -72·74)	16787·06 (10952·12 to 24108·17)	18049·51 (12190·82 to 25985·03)	41·79 (27·57 to 60·33)	34·22 (22·9 to 48·74)	-18·11 (-26·82 to -7·68)
**Inflammatory bowel disease**	6844·01 (4621·47 to 8295·41)	3410·3 (2747·7 to 5131·82)	25·9 (13·01 to 32·47)	4·32 (3·46 to 6·56)	-83·33 (-88·17 to -63·7)	2653·94 (1700·06 to 3752·95)	7041·1 (4748·35 to 9672·35)	5·81 (3·7 to 8·18)	10·68 (7·15 to 14·76)	83·72 (56·49 to 120·26)
**Vascular intestinal disorders**	2953·35 (2212·98 to 4308·5)	9156·17 (6736·07 to 10930·86)	10·58 (8·09 to 14·67)	10·74 (7·98 to 12·83)	1·51 (-42·6 to 47·44)	362·76 (217·99 to 556·73)	1142·11 (700·83 to 1675·97)	0·91 (0·56 to 1·37)	1·48 (0·93 to 2·13)	63·13 (39·01 to 92·81)
**Gallbladder and biliary diseases**	21178·13 (17201·64 to 26813·19)	23832·17 (17693·84 to 29058·4)	81·53 (69·99 to 104·76)	28·95 (21·44 to 35·08)	-64·49 (-76·18 to -55·7)	38185·68 (24199·01 to 56658·65)	54296·87 (34359·55 to 81383·39)	101·31 (65·42 to 149·86)	70·86 (44·7 to 105·6)	-30·06 (-37·01 to -22·6)
**Pancreatitis**	16671·7 (10822·44 to 26179)	12020·4 (8514·27 to 15824·31)	44·9 (30·11 to 66·65)	14·92 (10·75 to 19·23)	-66·77 (-83·04 to -36·45)	957·27 (537·03 to 1567·98)	2047·47 (1087·76 to 3566·93)	2·41 (1·35 to 3·91)	2·76 (1·49 to 4·77)	14·74 (-0·97 to 30·29)
**Other digestive diseases**	5330·13 (4621·84 to 7938·81)	8568·84 (6046·03 to 10630·09)	19·05 (16·52 to 26)	10·51 (7·45 to 13·02)	-44·83 (-70·69 to -27·46)	2484·53 (1698·11 to 3394·95)	3179·61 (2198·15 to 4427·59)	7·06 (4·88 to 9·7)	5·34 (3·73 to 7·38)	-24·31 (-29·71 to -18·49)
**Neurological disorders**	110285·5 (75103·38 to 189111·05)	293819·07 (145714·46 to 604924·32)	440·2 (237·52 to 879·18)	370·19 (191·21 to 740·42)	-15·9 (-32·09 to -8·57)	312933·36 (113963·45 to 603265·74)	433088·94 (209117·77 to 764227·22)	737·27 (313·18 to 1334·13)	682·34 (301·58 to 1232·5)	-7·45 (-22·98 to 12·59)
**Alzheimer's disease and other dementias**	43688·47 (10263·58 to 119550·07)	199604·03 (50456·1 to 517540·21)	261·03 (61·94 to 694·99)	242·42 (61·39 to 619·97)	-7·13 (-16·62 to 6·11)	18944·28 (13464·99 to 25450·55)	87340·36 (61380·05 to 117213·28)	107·85 (76·12 to 144·57)	103·6 (73·01 to 138·59)	-3·94 (-10·21 to 2·47)
**Parkinson's disease**	11569·9 (10423·78 to 19058·38)	51261·83 (40084·27 to 56515·51)	50·56 (44·99 to 85·44)	57·82 (45·67 to 63·75)	14·36 (-44·04 to 32·85)	2256·6 (1528·78 to 3122·56)	10450·7 (7165·97 to 14486·05)	8·49 (5·76 to 11·66)	11·68 (7·98 to 16·14)	37·56 (13·95 to 66·42)
**Idiopathic epilepsy**	33590·29 (30014·13 to 47312·73)	16141·42 (14364·42 to 19415·5)	73·05 (65·31 to 104·91)	30·06 (26·61 to 37·03)	-58·85 (-72·84 to -47·42)	37445·32 (8087·65 to 79686·98)	31622·16 (8050·51 to 69709·42)	84·69 (17·82 to 180·16)	61·33 (15·55 to 136·34)	-27·59 (-83·02 to 211·31)
**Multiple sclerosis**	1675·47 (1408·14 to 2553·94)	2361·66 (1919·79 to 3440·14)	4·11 (3·46 to 6·2)	3·01 (2·46 to 4·26)	-26·86 (-57·04 to -1·14)	987·04 (648·25 to 1395·76)	1864·69 (1238·93 to 2608·11)	2·36 (1·57 to 3·29)	2·45 (1·62 to 3·46)	3·75 (-0·2 to 8·1)
**Motor neuron disease**	7995·21 (6434·21 to 10002·12)	2248·22 (1940·31 to 2564·64)	23·18 (18·44 to 29·23)	2·64 (2·31 to 2·98)	-88·61 (-91·32 to -85·27)	296·72 (202·68 to 407·25)	593·9 (401·97 to 818·69)	0·71 (0·48 to 0·97)	0·89 (0·6 to 1·21)	25·89 (19·81 to 32·84)
**Headache disorders**	NA	NA	NA	NA	NA	245228·05 (52814·39 to 528157·36)	292109·2 (80775·33 to 602652·7)	515·11 (118·82 to 1106·26)	483·65 (117·89 to 1028·07)	-6·11 (-12·56 to 2·7)
**Migraine**	NA	NA	NA	NA	NA	217755·91 (34070·08 to 501098·76)	251195·75 (54577·36 to 561351·44)	454·78 (77·09 to 1031·19)	420·67 (80·12 to 942·23)	-7·5 (-13·79 to 1·36)
**Tension-type headache**	NA	NA	NA	NA	NA	27472·14 (7262·52 to 100475·95)	40913·45 (11577·83 to 136214·86)	60·33 (16·51 to 213·97)	62·98 (16·51 to 228·37)	4·39 (-9·83 to 16·18)
**Other neurological disorders**	11766·15 (9878·83 to 13942·5)	22201·91 (20101·99 to 24616·74)	28·28 (23·79 to 33·44)	34·25 (31·06 to 37·95)	21·1 (-2·96 to 53·36)	7775·36 (2523·74 to 15259·74)	9107·94 (4584·74 to 15950·3)	18·07 (6·6 to 34·76)	18·76 (7·7 to 37·32)	3·81 (-60·59 to 207·01)

**Mental disorders**	64·86 (35·08 to 111·39)	48·22 (33·66 to 67·5)	0·12 (0·07 to 0·2)	0·1 (0·07 to 0·14)	-17·82 (-59·88 to 56·54)	600022·64 (439613·33 to 793323·32)	794557·63 (582675·27 to 1038962·41)	1291·31 (952·08 to 1702·96)	1334·49 (975·63 to 1758·48)	3·34 (0·28 to 6·69)
**Schizophrenia**	NA	NA	NA	NA	NA	86547·3 (59487·88 to 117170·95)	134587·19 (93721·39 to 176673·4)	187·93 (132·25 to 252·13)	194·88 (134·03 to 259·99)	3·7 (-4·43 to 13·39)
**Depressive disorders**	NA	NA	NA	NA	NA	151184·35 (102870·4 to 208817·72)	227039·43 (158456·33 to 308654·02)	329·09 (227·69 to 453·28)	357·64 (248·35 to 491·66)	8·67 (1·62 to 15·56)
**Major depressive disorder**	NA	NA	NA	NA	NA	119944·26 (80451·01 to 168315·68)	183103·41 (127196·25 to 250324·48)	261·33 (177·67 to 362·34)	289·88 (198·35 to 400·17)	10·93 (2·26 to 19·91)
**Dysthymia**	NA	NA	NA	NA	NA	31240·09 (18902·03 to 48546·36)	43936·02 (27741·08 to 66979·25)	67·76 (41·82 to 104·81)	67·75 (41·18 to 104·61)	-0·01 (-5·06 to 5·17)
**Bipolar disorder**	NA	NA	NA	NA	NA	49682·91 (28754·16 to 78551·1)	69048·24 (41586·26 to 108121·8)	105·86 (63·16 to 168·72)	109·01 (64·81 to 170·97)	2·97 (-4·91 to 12·1)
**Anxiety disorders**	NA	NA	NA	NA	NA	144988·32 (96025·38 to 206636·09)	177414·5 (118498·36 to 251197·77)	310·72 (206·99 to 440·65)	310·93 (205·17 to 439·93)	0·07 (-6·88 to 7·87)
**Eating disorders**	64·86 (35·08 to 111·39)	48·22 (33·66 to 67·5)	0·12 (0·07 to 0·2)	0·1 (0·07 to 0·14)	-17·82 (-59·88 to 56·54)	27244·36 (16907·57 to 40862·44)	35569·64 (22375·73 to 52706·2)	50·58 (31·51 to 75·55)	75·22 (47·08 to 111·69)	48·72 (34·52 to 64·7)
**Anorexia nervosa**	60·4 (32·55 to 103·14)	41·91 (28·73 to 59·51)	0·11 (0·06 to 0·19)	0·09 (0·06 to 0·13)	-22·38 (-62·76 to 48·44)	8902·4 (5332·76 to 14022·62)	10521·82 (6379·55 to 16551·62)	16·53 (9·85 to 25·79)	23·4 (14·04 to 37·51)	41·55 (17·54 to 69·95)
**Bulimia nervosa**	4·47 (1·9 to 10·06)	6·32 (2·72 to 11·26)	0·01 (0 to 0·02)	0·01 (0·01 to 0·02)	41·8 (-49·03 to 278·98)	18341·96 (10435·45 to 28924·11)	25047·83 (14437·58 to 38749·08)	34·05 (19·4 to 53·55)	51·82 (30·2 to 79·76)	52·2 (34·35 to 71·45)
**Autism spectrum disorders**	NA	NA	NA	NA	NA	37424·39 (24234·62 to 54071·88)	41017·25 (26331·87 to 59285·83)	82·55 (53·6 to 119·57)	84·14 (53·82 to 121·94)	1·93 (-3·01 to 7·55)
**Attention-deficit/hyperactivity disorder**	NA	NA	NA	NA	NA	8595·71 (4748·07 to 14802·84)	6359·01 (3561·72 to 10762·86)	17·66 (9·83 to 30·27)	16·77 (9·35 to 28·79)	-5·02 (-20·99 to 11·66)
**Conduct disorder**	NA	NA	NA	NA	NA	30729·94 (17075·29 to 48916·1)	16943·03 (9174·14 to 27190·54)	64·39 (35·47 to 102·82)	61·34 (33·04 to 98·35)	-4·73 (-14·81 to 6·43)
**Idiopathic developmental intellectual disability**	NA	NA	NA	NA	NA	11104·65 (4204·85 to 19334·95)	2229·63 (453·57 to 5006·75)	24·31 (9·3 to 42·33)	4·09 (0·57 to 9·71)	-83·18 (-93·87 to -75·26)
**Other mental disorders**	NA	NA	NA	NA	NA	52520·72 (32890·81 to 79591·15)	84349·7 (53187·63 to 126636·84)	118·23 (74·34 to 177·74)	120·48 (75·81 to 181·46)	1·9 (-3·08 to 7·16)
**Substance use disorders**	47790·96 (40470·84 to 54154·65)	36980·44 (31228·4 to 45992·45)	110·96 (95·39 to 127·14)	46·12 (39·23 to 59·51)	-58·44 (-67·5 to -25·38)	191309·71 (131474·6 to 262855·93)	212930·14 (147357·18 to 293520·41)	397·38 (272·69 to 543·21)	338·3 (230·84 to 464·68)	-14·87 (-25·18 to -0·32)

**Alcohol use disorders**	44357·51 (37184·97 to 50767·75)	33076·2 (27295·43 to 41948·48)	103·15 (87·68 to 119·3)	40·15 (33·53 to 53·31)	-61·07 (-70·47 to -25·55)	144346·76 (94045·79 to 203841·97)	156721·86 (105803·69 to 223625·6)	307·64 (202·35 to 430·54)	233·52 (153·7 to 334·5)	-24·09 (-36·39 to -6·54)
**Drug use disorders**	3433·46 (3084·93 to 3790·26)	3904·24 (3503·87 to 4368·42)	7·81 (7·13 to 8·53)	5·96 (5·34 to 6·69)	-23·64 (-34·13 to -10·78)	46962·95 (31635·13 to 65761·86)	56208·28 (38848·32 to 76938·58)	89·74 (60·54 to 124·72)	104·78 (71·47 to 143·08)	16·77 (2·84 to 31·99)
**Opioid use disorders**	2579·94 (2298·5 to 2873·32)	2500·79 (2207·98 to 2839·57)	5·82 (5·22 to 6·4)	3·98 (3·48 to 4·52)	-31·61 (-42·18 to -17·98)	8827·78 (5470·83 to 13537·3)	18492·41 (11718·63 to 26652·17)	17·71 (10·98 to 26·26)	32·15 (20·03 to 48·04)	81·51 (48·98 to 122·67)
**Cocaine use disorders**	308·52 (250·46 to 410·36)	344·11 (285·7 to 410·97)	0·72 (0·59 to 0·95)	0·48 (0·4 to 0·57)	-33·35 (-54·78 to -9·72)	9839·09 (5439·42 to 15625·73)	10533·11 (6109·93 to 16489·37)	19·22 (10·85 to 30·26)	19·3 (11·03 to 30·66)	0·44 (-17·5 to 22·1)
**Amphetamine use disorders**	166·13 (135·11 to 208·65)	173·99 (145·78 to 207·91)	0·38 (0·31 to 0·47)	0·24 (0·21 to 0·29)	-34·86 (-53·11 to -13·43)	8208·95 (4297·77 to 13833·69)	7346·82 (3894·32 to 12396·95)	14·9 (7·93 to 25·04)	14·88 (7·88 to 25·3)	-0·16 (-21·73 to 23·84)
**Cannabis use disorders**	NA	NA	NA	NA		7198·45 (3853·2 to 12250·28)	6373·94 (3527·62 to 10363·27)	13·33 (7·26 to 22·7)	13·43 (7·18 to 22·23)	0·71 (-11·24 to 15·32)
**Other drug use disorders**	378·86 (300·6 to 458·92)	885·35 (778·86 to 1020·17)	0·9 (0·73 to 1·06)	1·26 (1·1 to 1·46)	40·57 (11·18 to 92·96)	12888·68 (7786·69 to 19610·08)	13462 (8326·71 to 19980·08)	24·58 (15·03 to 36·96)	25·03 (15 to 37·34)	1·85 (-22·07 to 33·26)
**Diabetes and kidney diseases**	230958·03 (221798·63 to 246944·72)	345370·05 (311660·22 to 377025·63)	692·88 (663·08 to 779·44)	397·71 (359·95 to 433·85)	-42·6 (-50·24 to -36·28)	117006·41 (79751·87 to 158684·26)	374815·11 (252455·64 to 513503·28)	349·66 (239·69 to 475·28)	437·56 (294·99 to 598·73)	25·14 (17·92 to 32·73)
**Diabetes mellitus**	139158 (132768·03 to 154098·35)	217776·21 (194782·67 to 241464·8)	422·44 (400·9 to 499·52)	248·44 (221·99 to 274·98)	-41·19 (-51·88 to -33·52)	96370·53 (63797·42 to 133515·89)	331217·48 (217192·78 to 461196·41)	286·81 (190·02 to 397·62)	383·51 (251·47 to 534·67)	33·72 (25·08 to 42·74)
**Diabetes mellitus type 1**	18110·31 (15049·93 to 20022·2)	7117·68 (5965·45 to 9557·51)	43·85 (35·62 to 49)	10·18 (8·62 to 13·53)	-76·78 (-81·35 to -65·79)	9993·56 (6314·87 to 15070·65)	16531·57 (10579·81 to 24579·6)	22·69 (14·45 to 34·16)	25·2 (16·13 to 37·64)	11·09 (1·58 to 22·16)
**Diabetes mellitus type 2**	121047·69 (115283·3 to 137360·56)	210658·53 (188054·84 to 232358·31)	378·59 (358·79 to 457·51)	238·26 (212·64 to 262·5)	-37·07 (-50·22 to -29·56)	86376·97 (56639·34 to 120364·35)	314685·9 (206483·95 to 438124·39)	264·13 (173·25 to 367·34)	358·31 (235·19 to 495·93)	35·66 (26·17 to 45·67)
**Chronic kidney disease**	91549·72 (87266·56 to 96338·34)	127368·38 (114782·73 to 140062·42)	269·61 (257·24 to 285·55)	148·99 (134·16 to 163·65)	-44·74 (-50·15 to -38·7)	20602·04 (15059·86 to 27243·58)	43566 (32337·1 to 56983·2)	62·77 (46·46 to 83·6)	53·97 (39·9 to 70·21)	-14·02 (-22·33 to -4·84)
**Chronic kidney disease due to diabetes mellitus type 1**	13164·94 (9400·12 to 17369·74)	10969·23 (7148·48 to 15351·56)	31·81 (22·09 to 42·37)	13·29 (9·06 to 18·11)	-58·22 (-63·94 to -51·48)	1041·51 (668·9 to 1542·95)	2577·52 (1570·67 to 3826·55)	2·48 (1·58 to 3·66)	3·29 (2·02 to 4·84)	32·7 (15·08 to 52·78)
**Chronic kidney disease due to diabetes mellitus type 2**	28996·32 (24058·1 to 33223·71)	61586·94 (52609·1 to 70447·33)	96·94 (82·01 to 110·53)	69·39 (59·28 to 79·55)	-28·42 (-37·1 to -18·72)	3851·15 (2720·8 to 5163·98)	12672·37 (9236·69 to 17061)	12·85 (9·22 to 17·44)	14·05 (10·23 to 18·86)	9·3 (-4·59 to 26·06)
**Chronic kidney disease due to hypertension**	17001·29 (14126·47 to 21269·29)	35970·98 (29796·45 to 43245·8)	59·55 (50·35 to 72·29)	41·7 (34·58 to 49·95)	-29·97 (-41·84 to -16·54)	3382·07 (2349·16 to 4613·11)	8927 (6469·67 to 11979·43)	10·7 (7·65 to 14·38)	10·64 (7·71 to 14·2)	-0·54 (-11·53 to 10·49)
**Chronic kidney disease due to glomerulonephritis**	21812·07 (19783·61 to 23986·66)	7148·34 (5683·81 to 8956·96)	51·27 (46·15 to 57·01)	9·24 (7·52 to 11·48)	-81·98 (-85·03 to -78·12)	3202·11 (2144·06 to 4503·95)	4042·26 (2747·6 to 5602·78)	7·33 (4·96 to 10·2)	5·92 (4·01 to 8·25)	-19·24 (-26·16 to -11·61)

**Chronic kidney disease due to other and unspecified causes**	10575·1 (9183·12 to 11929·35)	11692·88 (8699·33 to 15163·03)	30·05 (25·54 to 34·99)	15·36 (11·7 to 19·5)	-48·87 (-56·61 to -39·88)	9125·21 (6631·19 to 12418·74)	15346·85 (11190·36 to 20701·46)	29·41 (21·26 to 39·71)	20·07 (14·73 to 26·76)	-31·76 (-39·4 to -23·12)
**Acute glomerulonephritis**	250·3 (181·77 to 322·88)	225·46 (99·86 to 284·8)	0·83 (0·61 to 1·06)	0·28 (0·13 to 0·35)	-66·07 (-82·4 to -48·99)	33·84 (18·45 to 56·03)	31·64 (17·88 to 50·81)	0·08 (0·04 to 0·13)	0·08 (0·04 to 0·13)	2·15 (-4·49 to 8·48)
**Skin and subcutaneous diseases**	4490·53 (3533·08 to 5556·11)	8935·46 (7124·7 to 10257·07)	17·42 (13·58 to 21·47)	10·99 (8·77 to 12·58)	-36·88 (-54·66 to -15·8)	251097·64 (161457·28 to 367763·27)	277060·52 (178588·75 to 413505·23)	573·11 (365·92 to 844·18)	594·79 (381·84 to 882·63)	3·78 (1·2 to 6·44)
**Dermatitis**	NA	NA	NA	NA	NA	79966·62 (43533·83 to 131458·92)	73599·61 (41336·54 to 120475·91)	189·28 (103·02 to 311·73)	190·99 (105·72 to 317·69)	0·9 (-4 to 6·43)
**Atopic dermatitis**	NA	NA	NA	NA	NA	74653·18 (39602 to 124552·31)	65736·01 (35567·77 to 111405·98)	177·09 (93·71 to 295·72)	178·79 (96·7 to 302·53)	0·96 (-4·06 to 6·62)
**Contact dermatitis**	NA	NA	NA	NA	NA	3567·82 (2225·04 to 5407·59)	5310·58 (3250·58 to 8065·91)	8·13 (5·09 to 12·24)	8·11 (5·03 to 12·14)	-0·24 (-11·44 to 12·51)
**Seborrhoeic dermatitis**	NA	NA	NA	NA	NA	1745·61 (1000·85 to 2770·66)	2553·01 (1444·31 to 4048·55)	4·07 (2·34 to 6·44)	4·09 (2·32 to 6·51)	0·48 (-7·89 to 9·38)
**Psoriasis**	NA	NA	NA	NA	NA	11349·09 (7969·66 to 15272·84)	14825·28 (10333·07 to 19862·45)	26·06 (18·42 to 34·99)	22·74 (15·84 to 30·77)	-12·75 (-22·77 to -1·29)
**Bacterial skin diseases**	1323·67 (932·53 to 1893·36)	2791·42 (2090·66 to 3468·96)	4·29 (2·99 to 6·18)	3·49 (2·66 to 4·29)	-18·45 (-43·58 to 21·45)	2737·66 (1492·82 to 4867·03)	3021·47 (1727·14 to 5211·25)	6·29 (3·49 to 11·13)	6·39 (3·48 to 11·36)	1·56 (-8·39 to 12·73)
**Cellulitis**	617·6 (348·08 to 964·27)	1290·97 (709·95 to 1660·01)	2·01 (1·12 to 3·08)	1·53 (0·85 to 1·98)	-23·68 (-54·7 to 30·88)	1054·18 (660·25 to 1550·08)	1333·05 (862·28 to 1944·4)	2·48 (1·58 to 3·67)	2·54 (1·6 to 3·81)	2·45 (-17·41 to 28·46)
**Pyoderma**	706·07 (454·94 to 1114·02)	1500·45 (1076·22 to 2266·55)	2·28 (1·44 to 3·66)	1·96 (1·46 to 2·88)	-13·83 (-43·65 to 37·47)	1683·49 (677·31 to 3544·56)	1688·42 (672·68 to 3557·95)	3·81 (1·54 to 8·01)	3·85 (1·53 to 8·12)	0·98 (-4·12 to 6·58)
**Scabies**	NA	NA	NA	NA	NA	1060·29 (572·29 to 1729·42)	1050·07 (587·46 to 1674·11)	2·29 (1·25 to 3·7)	2·33 (1·29 to 3·72)	1·85 (-10·99 to 16·1)
**Fungal skin diseases**	NA	NA	NA	NA	NA	11906·06 (4788·66 to 25485·37)	20387·62 (8288·24 to 44434·55)	30·96 (12·52 to 67·2)	30·48 (12·24 to 65·9)	-1·56 (-3·22 to -0·05)
**Viral skin diseases**	NA	NA	NA	NA	NA	52174·02 (33571·28 to 78074·85)	50950·67 (32920·15 to 74988·72)	117·64 (75·73 to 176·13)	117·8 (75·42 to 176·2)	0·13 (-2·53 to 2·82)
**Acne vulgaris**	NA	NA	NA	NA	NA	43487·72 (25912·8 to 69335·16)	39930·36 (23751·87 to 62766·92)	84·28 (50·18 to 134·28)	102·67 (60·79 to 162·86)	21·82 (13·58 to 30·91)
**Alopecia areata**	NA	NA	NA	NA	NA	4416·18 (2774·47 to 6569·25)	5551·74 (3585·22 to 8193·82)	9·41 (5·89 to 14·06)	9·28 (5·92 to 13·83)	-1·34 (-8·79 to 7·16)

**Pruritus**	NA	NA	NA	NA	NA	2992·98 (1408·84 to 5492·77)	4861·16 (2302·78 to 8714·32)	7·1 (3·37 to 12·81)	7·6 (3·56 to 13·49)	7·16 (1·36 to 13·14)
**Urticaria**	NA	NA	NA	NA	NA	19898·38 (13023·53 to 28937·45)	20866·7 (13567·55 to 29611·25)	45·68 (30·06 to 66·66)	45·39 (29·23 to 65·77)	-0·62 (-6·99 to 5·79)
**Decubitus ulcer**	2863·06 (2180·6 to 3654·7)	5094·13 (3867·98 to 6116·04)	12·21 (9·34 to 15·52)	6·19 (4·68 to 7·42)	-49·27 (-64·86 to -30·52)	640·51 (434·81 to 872·33)	2086·98 (1423·81 to 2843·91)	2·15 (1·48 to 2·9)	2·66 (1·83 to 3·63)	23·71 (8·26 to 40·76)
**Other skin and subcutaneous diseases**	303·8 (219·04 to 435·18)	1049·91 (680·27 to 1377·4)	0·92 (0·65 to 1·31)	1·3 (0·86 to 1·69)	41·76 (-16·54 to 108·48)	20468·12 (9792·65 to 37517·44)	39928·85 (19012·47 to 72086·71)	51·97 (24·99 to 94·78)	56·45 (26·88 to 102·71)	8·62 (6·26 to 10·95)
**Sense organ diseases**	NA	NA	NA	NA	NA	181740·56 (124735·18 to 255622·12)	398119·24 (271308·36 to 566719·49)	553·6 (386·76 to 774·12)	500·81 (341·93 to 705·71)	-9·54 (-13·32 to -5·95)
**Blindness and vision loss**	NA	NA	NA	NA	NA	44227·67 (30718·07 to 61529·31)	75097·55 (51936·84 to 104345·73)	129·08 (90·8 to 176·5)	104·36 (71·75 to 145·46)	-19·15 (-24·17 to -14·21)
**Glaucoma**	NA	NA	NA	NA	NA	2386·25 (1569·09 to 3464·73)	5197·8 (3510·65 to 7245·18)	10·94 (7·37 to 15·72)	6 (4·08 to 8·33)	-45·21 (-50·95 to -38·19)
**Cataract**	NA	NA	NA	NA	NA	6733·57 (4666·68 to 9355·65)	17409·29 (12073·35 to 24180·84)	26·1 (17·94 to 35·64)	20·34 (14·11 to 28·27)	-22·06 (-29·03 to -14·61)
**Age-related macular degeneration**	NA	NA	NA	NA	NA	769·37 (479·95 to 1126·17)	1815·85 (1193·33 to 2609·23)	3·4 (2·22 to 4·92)	2·08 (1·38 to 2·99)	-38·72 (-47·43 to -27·57)
**Refraction disorders**	NA	NA	NA	NA	NA	22102·18 (14400·39 to 32459·67)	31347·76 (20645·98 to 45076·78)	53·89 (35·33 to 78·06)	51·15 (33·36 to 75·17)	-5·09 (-9·56 to -0·42)
**Near vision loss**	NA	NA	NA	NA	NA	1671·39 (749·02 to 3475·27)	4638·61 (2009·83 to 9321·65)	5·71 (2·56 to 11·51)	5·27 (2·3 to 10·59)	-7·61 (-20·66 to 2·92)
**Other vision loss**	NA	NA	NA	NA	NA	10564·91 (7144·22 to 14761·9)	14688·24 (10085·63 to 20383·09)	29·03 (20·07 to 39·88)	19·51 (13·35 to 27·01)	-32·79 (-39·67 to -25·28)
**Age-related and other hearing loss**	NA	NA	NA	NA	NA	120775·52 (81641·52 to 172714·31)	285944·23 (191872·23 to 415199·51)	377·95 (259·86 to 537·11)	347·74 (234·73 to 503·09)	-7·99 (-12·59 to -3·75)
**Other sense organ diseases**	NA	NA	NA	NA	NA	16737·37 (9890·56 to 25746·75)	37077·47 (21890·76 to 58037·92)	46·57 (27·78 to 71·49)	48·71 (28·78 to 76·08)	4·58 (-0·13 to 9·96)
**Musculoskeletal disorders**	12378·3 (11005·73 to 18792·09)	20859·09 (16325·34 to 24993·8)	38·7 (34·24 to 51)	26·55 (21·29 to 32·9)	-31·4 (-44·2 to -21·43)	843257·43 (591404·88 to 1131760·4)	1679821·92 (1176406·94 to 2270147·03)	2093·43 (1469·34 to 2808·24)	2219·89 (1559·49 to 2987·67)	6·04 (3 to 9·48)
**Rheumatoid arthritis**	3198·87 (2554·91 to 4206·47)	4125·47 (3035·13 to 6006·52)	10·84 (8·41 to 13·67)	4·67 (3·44 to 6·82)	-56·95 (-68·8 to -37·82)	9514·26 (6520·35 to 13020·09)	25075·96 (17025·69 to 34573·4)	24·55 (16·83 to 33·53)	30·58 (20·84 to 42·09)	24·54 (12·78 to 38·33)

**Osteoarthritis**	NA	NA	NA	NA	NA	116173·35 (58609·89 to 235425·29)	334791·59 (169397·27 to 661648·87)	372·14 (189·15 to 746·52)	369·39 (186·93 to 729·56)	-0·74 (-3·23 to 2·03)
**Osteoarthritis hip**	NA	NA	NA	NA	NA	4263·49 (1961·28 to 8752·12)	13659·86 (6492·02 to 28490·65)	13·78 (6·39 to 28·08)	15·26 (7·25 to 31·7)	10·75 (1·91 to 19·56)
**Osteoarthritis knee**	NA	NA	NA	NA	NA	60711·95 (29724·79 to 123248·9)	179409·43 (89536·86 to 364811·07)	194·76 (96·21 to 394·45)	197·96 (98·58 to 402·76)	1·64 (-2·79 to 6·42)
**Osteoarthritis hand**	NA	NA	NA	NA	NA	45151·87 (22928·88 to 93461·21)	122864·61 (61272·73 to 256518·05)	143·48 (72·48 to 293·95)	135·2 (68·07 to 281·23)	-5·77 (-7·85 to -3·79)
**Osteoarthritis other**	NA	NA	NA	NA	NA	6046·04 (2922·37 to 12628·32)	18857·69 (9321·12 to 39159·95)	20·12 (9·94 to 41·83)	20·97 (10·38 to 43·47)	4·21 (-2·45 to 10·73)
**Low back pain**	NA	NA	NA	NA	NA	442336·83 (306566·74 to 601507·13)	714324·8 (501072·42 to 966622·63)	1054·29 (738·6 to 1428·24)	993·31 (693·92 to 1338·83)	-5·78 (-9·66 to -1·62)
**Neck pain**	NA	NA	NA	NA	NA	69942·12 (46032·26 to 100424·73)	132335·85 (86384·5 to 194651·27)	172·29 (113·62 to 247·81)	171·97 (112·56 to 247·48)	-0·19 (-3·33 to 3·02)
**Gout**	NA	NA	NA	NA	NA	6630·34 (4101·14 to 9687·02)	18854·71 (11572·42 to 27691·75)	19·19 (11·9 to 27·38)	21·96 (13·66 to 31·95)	14·44 (0·78 to 29·36)
**Other musculoskeletal disorders**	9179·43 (7700·6 to 14973·94)	16733·62 (13046·83 to 19953·59)	27·87 (23·81 to 37·46)	21·89 (17·47 to 27·03)	-21·46 (-38·46 to -9·08)	198660·54 (130683·68 to 283225·24)	454439 (305798·59 to 632444·68)	450·97 (296·67 to 635·94)	632·68 (430·88 to 878·33)	40·29 (29·39 to 53·49)
**Other non-communicable diseases**	276895·04 (203223·67 to 319252·45)	78269·15 (66324·77 to 86791·95)	807·11 (595·49 to 934·79)	216·21 (187·02 to 250·75)	-73·21 (-77·63 to -61·05)	389700·11 (268049·46 to 547545·93)	535189·57 (361613·32 to 766220·7)	903·49 (619·74 to 1256·71)	838·62 (573·17 to 1180·32)	-7·18 (-9·85 to -4·58)
**Congenital birth defects**	222587·63 (150221·4 to 260897·71)	31865·19 (27021·89 to 40536·08)	651·66 (438·74 to 767·15)	136·98 (113·99 to 177·07)	-78·98 (-83·61 to -59·92)	48399·32 (33402·4 to 67660·09)	38766·85 (26876·53 to 53386·03)	113·27 (77·56 to 159·29)	105·83 (72·87 to 147·81)	-6·57 (-13·87 to 1·88)
**Neural tube defects**	2891·44 (1707·6 to 4233·14)	2715·26 (1932·83 to 3710·68)	8·46 (4·96 to 12·48)	12·24 (8·52 to 16·9)	44·69 (-14·65 to 183·01)	1534·27 (990·3 to 2141·49)	1788·21 (1188·91 to 2489·3)	3·31 (2·14 to 4·61)	4·51 (2·97 to 6·27)	36·39 (19·94 to 56·07)
**Congenital heart anomalies**	150816·55 (75078·73 to 182939·37)	13535·34 (10903·77 to 16834·43)	441·63 (215·79 to 538·3)	56·17 (43·7 to 71·02)	-87·28 (-91·13 to -71·16)	3600·33 (1622·92 to 6120·11)	3428·52 (1503·84 to 5931·08)	8·62 (3·93 to 14·6)	8·43 (3·7 to 14·8)	-2·11 (-13·25 to 9·46)
**Orofacial clefts**	1240·24 (527·57 to 2375·15)	23·75 (15·96 to 33·44)	3·8 (1·61 to 7·26)	0·11 (0·07 to 0·16)	-97·11 (-98·56 to -92·54)	1307·4 (775·53 to 1963·19)	1524·06 (903·84 to 2274·12)	3·13 (1·87 to 4·74)	3·14 (1·87 to 4·72)	0·44 (-23·22 to 30·22)
**Down syndrome**	6563·28 (3891·66 to 8894·19)	1901·3 (1399·91 to 2495·16)	18·4 (10·84 to 25·26)	7·36 (5·11 to 9·92)	-60·02 (-75·92 to -18·94)	2138·41 (1328·55 to 3236·69)	2009·4 (1255·91 to 2968·26)	4·51 (2·81 to 6·84)	5·4 (3·35 to 7·92)	19·64 (1·63 to 42·78)
**Turner syndrome**	NA	NA	NA	NA	NA	203·5 (85·53 to 365·55)	152·97 (68·52 to 266·81)	0·42 (0·18 to 0·77)	0·36 (0·15 to 0·64)	-15·49 (-45·19 to 32·21)

**Klinefelter syndrome**	NA	NA	NA	NA	NA	1·39 (0·58 to 2·78)	1·47 (0·61 to 3)	0 (0 to 0·01)	0 (0 to 0·01)	16·49 (0·56 to 37·4)
**Other chromosomal abnormalities**	4890·28 (3186·31 to 12869·3)	3952·79 (2484·94 to 8387·16)	14·27 (9·17 to 38·3)	17·75 (10·96 to 39·98)	24·34 (-40·43 to 154·05)	3634·48 (2376·74 to 5209·79)	2957·6 (1938·56 to 4267·35)	8·23 (5·39 to 11·76)	8·67 (5·68 to 12·46)	5·33 (-8·7 to 20·43)
**Congenital musculoskeletal and limb anomalies**	5722·52 (2803·7 to 8080·11)	1508·18 (864·91 to 2103·29)	16·82 (8·17 to 23·99)	6·92 (3·88 to 9·86)	-58·85 (-79·85 to -20·71)	28112·07 (17481·99 to 42500·44)	19997·11 (12795·78 to 29334·96)	66·73 (41·58 to 101·78)	57·77 (36·13 to 88·04)	-13·42 (-24·27 to 0·1)
**Urogenital congenital anomalies**	2098·94 (1396·99 to 3249·28)	581·77 (454·61 to 767·67)	6·12 (4 to 9·63)	2·21 (1·63 to 3·07)	-63·92 (-79·24 to -35·94)	1823·32 (987·03 to 2945·95)	1378·98 (789·49 to 2245·55)	4·29 (2·35 to 6·98)	4·49 (2·53 to 7·25)	4·66 (-9·42 to 20·36)
**Digestive congenital anomalies**	14502·82 (6501·28 to 20333·26)	1975·75 (1338·44 to 2705·72)	43·35 (19·45 to 61·01)	9·11 (6·09 to 12·57)	-78·98 (-88·37 to -47·83)	1170·62 (734·14 to 1772·66)	1435·71 (909·53 to 2106·59)	2·86 (1·81 to 4·28)	3·96 (2·53 to 5·95)	38·45 (14·76 to 69·94)
**Other congenital birth defects**	33861·56 (17876·98 to 50047·74)	5671·04 (4367·6 to 7663·64)	98·81 (51·75 to 146·42)	25·12 (19·08 to 34·59)	-74·58 (-84·68 to -41·36)	4873·54 (2422·13 to 9608·78)	4092·81 (2030·14 to 8165·05)	11·17 (5·55 to 21·91)	9·1 (4·79 to 17·07)	-18·59 (-32·8 to -2·3)
**Urinary diseases and male infertility**	6943·1 (6212·04 to 10759·95)	21560·28 (11763·03 to 25476·42)	24·78 (21·49 to 42·08)	26·71 (14·45 to 31·59)	7·8 (-62·97 to 39·28)	7416·54 (4838·97 to 10681·86)	16794·01 (10755·86 to 24439·67)	19·72 (12·91 to 28·45)	21·82 (14·23 to 31·79)	10·64 (1·45 to 20·45)
**Urinary tract infections and interstitial nephritis**	4072·8 (2983·68 to 6305·4)	13200·89 (7483·04 to 18499·83)	15·2 (11·27 to 27·24)	16·24 (9·16 to 22·81)	6·87 (-62·37 to 80·42)	1556·2 (928·84 to 2378·92)	1883·83 (1156·26 to 2820·59)	3·53 (2·11 to 5·36)	3·56 (2·15 to 5·35)	0·92 (-13·73 to 16·11)
**Urolithiasis**	444·17 (303·58 to 677·61)	817·51 (460·28 to 1275·69)	1·52 (1·03 to 2·38)	0·97 (0·54 to 1·54)	-36·19 (-71·08 to 15·71)	1607·63 (985·66 to 2364)	3118·09 (1881·47 to 4618·92)	3·85 (2·37 to 5·63)	4·02 (2·52 to 5·94)	4·54 (-15·71 to 29·28)
**Benign prostatic hyperplasia**	NA	NA	NA	NA	NA	2486·32 (1411·87 to 3894·11)	9403·2 (5378·55 to 14797·98)	8·56 (4·91 to 13·3)	10·17 (5·84 to 15·91)	18·77 (5·73 to 32·67)
**Male infertility**	NA	NA	NA	NA	NA	996·7 (366·43 to 2356·73)	1163·82 (420·8 to 2682·06)	1·96 (0·72 to 4·6)	2·06 (0·75 to 4·73)	5·18 (-5·48 to 17·91)
**Other urinary diseases**	2426·13 (1919·86 to 4004·11)	7541·88 (3345·07 to 10619·18)	8·06 (6·36 to 14·11)	9·5 (4·23 to 13·33)	17·86 (-64·58 to 95·73)	769·69 (511·33 to 1086·96)	1225·07 (815·53 to 1733·44)	1·82 (1·21 to 2·56)	2 (1·32 to 2·82)	9·95 (-4·09 to 25·39)
**Gynecological diseases**	504·99 (213·06 to 692·74)	264·74 (178·26 to 642·7)	1·31 (0·55 to 1·76)	0·37 (0·25 to 0·88)	-71·69 (-80·51 to -26·85)	113413·11 (76318·65 to 161543·6)	122423·23 (83595·66 to 171576·54)	227·52 (154·49 to 321·77)	210·59 (142·76 to 300·9)	-7·44 (-11·31 to -3·49)
**Uterine fibroids**	235·33 (111·54 to 374·27)	86·93 (55·77 to 209·68)	0·63 (0·3 to 0·98)	0·11 (0·07 to 0·27)	-82·37 (-89·26 to -57·81)	4629·62 (2139·12 to 8835·56)	6590·18 (3070·29 to 12584·74)	9·75 (4·55 to 18·59)	10·04 (4·63 to 19·51)	3 (-5·48 to 11·98)
**Polycystic ovarian syndrome**	NA	NA	NA	NA	NA	4280·25 (1772·46 to 9001·31)	6937·74 (2905·16 to 14689·35)	8·2 (3·41 to 17·33)	13·01 (5·47 to 27·44)	58·66 (40·43 to 80·6)
**Female infertility**	NA	NA	NA	NA	NA	458·7 (39·06 to 1592·22)	551·09 (41·3 to 2000·37)	0·96 (0·08 to 3·43)	0·9 (0·07 to 3·23)	-6·26 (-52·55 to 74·27)

**Endometriosis**	33·3<br>(5 to 85·72)	3·97<br>(0·97 to 11·55)	0·07<br>(0·01 to 0·17)	0·01<br>(0 to 0·02)	-90·51<br>(-97·81 to -46·05)	17895·01<br>(10269·53 to 28932·85)	16550·27<br>(9531·88 to 26104·66)	34·92<br>(20·26 to 55·25)	28·94<br>(16·96 to 46·19)	-17·14<br>(-27·34 to -6·77)
**Genital prolapse**	177·09<br>(56·1 to 249·67)	105·74<br>(66·02 to 256·59)	0·45<br>(0·14 to 0·62)	0·16<br>(0·1 to 0·38)	-63·94<br>(-77·93 to 6·63)	744·95<br>(345·7 to 1420·41)	1731·46<br>(811·77 to 3337·27)	2·52<br>(1·19 to 4·8)	1·93<br>(0·9 to 3·67)	-23·49<br>(-31·26 to -14·76)
**Premenstrual syndrome**	NA	NA	NA	NA	NA	46747·65<br>(28298·31 to 74168·39)	44106·27<br>(26475·27 to 69450·02)	91·6<br>(54·96 to 144·83)	82·92<br>(49·79 to 130·55)	-9·47<br>(-14·58 to -4·3)
**Other gynecological diseases**	59·26<br>(20·1 to 91·73)	68·1<br>(36·19 to 165·46)	0·16<br>(0·05 to 0·24)	0·09<br>(0·05 to 0·22)	-42·88<br>(-66·98 to 68·81)	38656·94<br>(24106 to 58544·77)	45956·2<br>(28512·44 to 68276·81)	79·57<br>(49·44 to 118·27)	72·86<br>(44·94 to 109·38)	-8·44<br>(-14·71 to -2·78)
**Hemoglobinopathies and hemolytic anaemias**	10360·05<br>(9639·25 to 11140·4)	6464·31<br>(5764·63 to 7127·31)	24·93<br>(23·28 to 26·76)	10·14<br>(9·09 to 11·24)	-59·34<br>(-64·66 to -53·8)	17419·9<br>(11299·19 to 25400·98)	7397·83<br>(4565·29 to 11175·76)	39·06<br>(25·59 to 56·92)	13·61<br>(8·52 to 21·31)	-65·16<br>(-71·45 to -58·05)
**Thalassemias**	4085·47<br>(3476·21 to 4734·94)	889·25<br>(713·07 to 1075·88)	9·09<br>(7·72 to 10·52)	2·99<br>(2·39 to 3·65)	-67·1<br>(-74·55 to -57·87)	95·99<br>(51·94 to 161·66)	41<br>(23·96 to 65·36)	0·24<br>(0·13 to 0·41)	0·16<br>(0·09 to 0·26)	-32·67<br>(-47·91 to -12·08)
**Thalassemias trait**	NA	NA	NA	NA	NA	2048·81<br>(1228·02 to 3184·62)	1541·55<br>(884·7 to 2453·07)	4·03<br>(2·4 to 6·25)	3<br>(1·75 to 4·81)	-25·58<br>(-41·13 to -5·21)
**Sickle cell disorders**	102·64<br>(65·67 to 147·96)	47·38<br>(29·6 to 68·14)	0·23<br>(0·15 to 0·33)	0·11<br>(0·07 to 0·16)	-49·88<br>(-62·42 to -33·62)	2·11<br>(1·19 to 3·33)	1·37<br>(0·75 to 2·22)	0<br>(0 to 0·01)	0<br>(0 to 0·01)	-11·54<br>(-25·34 to 3·53)
**Sickle cell trait**	NA	NA	NA	NA	NA	936·55<br>(535·85 to 1491·12)	494·64<br>(266·73 to 796)	1·87<br>(1·08 to 2·97)	0·97<br>(0·52 to 1·56)	-48·24<br>(-60·61 to -28·85)
**G6PD deficiency**	619·42<br>(391·46 to 878·26)	418·79<br>(277·21 to 584·72)	1·25<br>(0·8 to 1·75)	0·68<br>(0·44 to 0·96)	-45·51<br>(-54·28 to -34·62)	320<br>(156·88 to 552·29)	103·1<br>(44·89 to 186·92)	0·61<br>(0·31 to 1·03)	0·21<br>(0·09 to 0·38)	-65·6<br>(-84·04 to -31·32)
**G6PD trait**	NA	NA	NA	NA	NA	116·68<br>(59·59 to 228·43)	46·75<br>(21·79 to 85·92)	0·23<br>(0·12 to 0·42)	0·09<br>(0·04 to 0·17)	-59·65<br>(-81·11 to -22·61)
**Other hemoglobinopathies and hemolytic anemias**	5552·53<br>(5036·34 to 6129·32)	5108·89<br>(4494·6 to 5673·97)	14·37<br>(13·23 to 15·63)	6·35<br>(5·63 to 7·07)	-55·77<br>(-61·2 to -50·21)	13899·75<br>(9100·1 to 20522·51)	5169·42<br>(3141·54 to 8054·57)	32·07<br>(21·03 to 47·16)	9·17<br>(5·63 to 14·36)	-71·41<br>(-77·27 to -64·54)
**Endocrine, metabolic, blood, and immune disorders**	21651·77<br>(17314·83 to 29604·89)	14958·76<br>(11384·65 to 20009·29)	59·17<br>(47·04 to 78·21)	26·39<br>(20·39 to 36·5)	-55·39<br>(-69·25 to -36·49)	117546·3<br>(78377·84 to 165710·36)	186400·55<br>(122183·32 to 263889·5)	276·07<br>(184·04 to 385·09)	268·14<br>(177·16 to 380·93)	-2·87<br>(-8·4 to 3·34)
**Oral disorders**	NA	NA	NA	NA	NA	85504·95<br>(49439·21 to 138763·49)	163407·11<br>(94062·08 to 267144·96)	227·86<br>(134·28 to 367·8)	218·64<br>(125·63 to 352·33)	-4·05<br>(-7·76 to -0·73)
**Caries of deciduous teeth**	NA	NA	NA	NA	NA	1232·62<br>(539·22 to 2541·91)	748·67<br>(329·73 to 1561·78)	3·25<br>(1·41 to 6·73)	3·18<br>(1·39 to 6·66)	-2·06<br>(-11·12 to 8·84)
**Caries of permanent teeth**	NA	NA	NA	NA	NA	12466·26<br>(5555·56 to 24175·18)	14749·24<br>(6631·22 to 29317·57)	26·74<br>(12·01 to 52·27)	24·94<br>(11·29 to 49·42)	-6·74<br>(-13·47 to -0·1)

**Periodontal diseases**	NA	NA	NA	NA	NA	22993·75 (8674·65 to 54730·82)	55147·9 (21844·29 to 126643·82)	60·37 (23·27 to 141·14)	66·46 (26·01 to 156·95)	10·1 (4·5 to 16·54)
**Edentulism**	NA	NA	NA	NA	NA	24844·04 (15819·75 to 37349·55)	61086·37 (38862·5 to 90611·25)	84·31 (53·6 to 124·29)	70·89 (44·7 to 105·63)	-15·92 (-20·42 to -11·86)
**Other oral disorders**	NA	NA	NA	NA	NA	23968·28 (14846 to 35507·53)	31674·93 (19552·01 to 46607·2)	53·19 (33·13 to 78·79)	53·17 (32·99 to 78·59)	-0·05 (-3·15 to 3·23)
**Sudden infant death syndrome**	14847·49 (4357·51 to 29986·23)	3155·87 (1109·6 to 5762·44)	45·26 (13·28 to 91·38)	15·62 (5·49 to 28·5)	-65·49 (-84·92 to -18·99)	NA	NA			
**Injuries**	1708449·82 (1643760·76 to 1881038·18)	973030·34 (827670·17 to 1045991·73)	3817·27 (3653·41 to 4236·31)	1496·93 (1324·49 to 1596·45)	-60·79 (-67·48 to -57·23)	469971·62 (330892·22 to 653006·47)	677588·47 (468538·09 to 952603·18)	1143·59 (804·3 to 1582·88)	943 (652·71 to 1328·26)	-17·54 (-20·69 to -14·36)
**Transport injuries**	815045·34 (755281·4 to 863737·39)	230658·17 (204080·21 to 311720·23)	1803·04 (1666·04 to 1913·65)	367·2 (322·34 to 509·14)	-79·63 (-82·38 to -72·49)	79028·82 (56984·62 to 104985·78)	74581·34 (53065·58 to 99686·53)	199·92 (144·51 to 265·12)	97·45 (69·11 to 130·53)	-51·26 (-52·86 to -49·76)
**Road injuries**	758970·2 (703310·12 to 805484·83)	210869·52 (185503·51 to 286147·64)	1682·11 (1553·71 to 1789·18)	339·31 (296·64 to 472·09)	-79·83 (-82·58 to -72·63)	72576·93 (52189·8 to 97029·75)	65872·36 (46561·71 to 88536·77)	182·67 (131·58 to 243·01)	85·59 (60·61 to 114·94)	-53·15 (-54·7 to -51·75)
**Pedestrian road injuries**	290542·61 (211793·42 to 347055·07)	82764·75 (71618·11 to 107780·96)	705·42 (527·41 to 831·83)	123·52 (106·53 to 158·16)	-82·49 (-86·35 to -71·33)	15931·39 (11014·68 to 21511·47)	11480·62 (7847·5 to 15676·45)	42·41 (29·83 to 57·27)	14·55 (10·1 to 19·85)	-65·69 (-67·2 to -64·14)
**Cyclist road injuries**	45704·22 (36922·18 to 61568·19)	11476·86 (8232·47 to 13985·49)	102·79 (83·33 to 139·61)	16·92 (11·7 to 20·55)	-83·54 (-91·2 to -78·01)	16331·22 (11254·37 to 22086·48)	16734·76 (11454·16 to 22872·47)	40·58 (28·11 to 54·98)	22·05 (15·09 to 30·01)	-45·65 (-47·64 to -43·53)
**Motorcyclist road injuries**	184417·62 (147814·01 to 242646·47)	38959·72 (24100·45 to 76958·04)	370·68 (296·75 to 489·07)	69·23 (40·48 to 136·65)	-81·32 (-90·53 to -63·09)	16134·26 (11505·58 to 21973·07)	13433·07 (9436·28 to 18493·15)	39·04 (27·7 to 52·97)	17·7 (12·51 to 24·4)	-54·66 (-56·72 to -52·65)
**Motor vehicle road injuries**	229388·94 (173891·28 to 273785·43)	77065·46 (63585·91 to 115930·27)	483·67 (355·34 to 577·43)	128·56 (104·89 to 205·99)	-73·42 (-81·15 to -51·66)	22987·01 (16188·3 to 30983·92)	22717·87 (15749·14 to 30889·31)	57·66 (40·11 to 77·7)	29·28 (20·46 to 39·77)	-49·22 (-51·02 to -47·55)
**Other road injuries**	8916·8 (6332·81 to 12866·29)	602·73 (393·5 to 746·88)	19·55 (13·94 to 28·17)	1·08 (0·67 to 1·33)	-94·47 (-97·52 to -91·88)	1193·05 (822·71 to 1650·69)	1506·04 (1028·75 to 2105·68)	2·99 (2·09 to 4·13)	2 (1·37 to 2·78)	-32·98 (-35·57 to -30·37)
**Other transport injuries**	56075·14 (50823·95 to 60955·53)	19788·65 (16831·51 to 26383·09)	120·93 (110·27 to 131·18)	27·9 (23·82 to 38·42)	-76·93 (-80·47 to -69·21)	6451·89 (4579·58 to 8632·67)	8708·98 (6147·98 to 11764·31)	17·24 (12·44 to 22·88)	11·86 (8·28 to 16·22)	-31·24 (-35·23 to -27·01)
**Unintentional injuries**	599828·96 (510735·82 to 642415·71)	210480·89 (183266·41 to 240770·7)	1402·2 (1242·01 to 1497·45)	323·63 (289·96 to 375·86)	-76·92 (-79·68 to -71·79)	365951·09 (255042·16 to 519389·51)	564634 (387806·91 to 808112·63)	887·76 (617·05 to 1253·61)	790·22 (540·73 to 1136·34)	-10·99 (-14·04 to -8·18)
**Falls**	140655·28 (94364·6 to 157674·41)	105130·94 (83825·78 to 133637·24)	336·19 (239·42 to 394·03)	144·74 (120·03 to 194·76)	-56·95 (-65·99 to -25·96)	158527·18 (110293·46 to 225351)	284360·26 (197209·58 to 402897)	396·27 (276·54 to 558·31)	383·89 (265·4 to 544·62)	-3·12 (-6·33 to 0·07)
**Drowning**	153360·23 (111054·5 to 167693·16)	24498·35 (20171·53 to 28199·18)	337·93 (253·86 to 369·84)	46·02 (37·83 to 53·54)	-86·38 (-88·79 to -81·71)	682·27 (487·1 to 909·46)	2690·66 (1893·33 to 3651·66)	1·71 (1·23 to 2·26)	3·81 (2·68 to 5·17)	122·17 (107·66 to 136·33)

**Fire, heat, and hot substances**	43178·75 (31522·07 to 50759·23)	14840·22 (11220·94 to 22221·05)	100·15 (76·95 to 122·76)	24·43 (18·59 to 37·36)	-75·61 (-80·99 to -56·35)	31327·35 (20919·46 to 45276·66)	32268·09 (18976·34 to 52155·73)	71·78 (47·74 to 104·01)	46·4 (27·51 to 73·74)	-35·35 (-45·05 to -25·69)
**Poisonings**	45741·79 (31478·7 to 51367·36)	6336·59 (5355·44 to 10650·98)	102·55 (68·9 to 114·96)	9·72 (8·13 to 17·32)	-90·52 (-92·59 to -75·11)	6916·1 (4619·17 to 9754·74)	6991·21 (4583·02 to 9917·26)	15·41 (10·32 to 21·74)	12·05 (7·74 to 17·3)	-21·78 (-29·31 to -13·83)
**Poisoning by carbon monoxide**	31727·32 (18195·62 to 39078·41)	4204·63 (3348·49 to 7758·73)	68·69 (38·25 to 84·67)	6·84 (5·41 to 13·3)	-90·04 (-93·15 to -71·58)	1517·22 (945·73 to 2265·17)	1222·22 (745·3 to 1906·81)	3·35 (2·11 to 4·96)	2·1 (1·26 to 3·31)	-37·54 (-44·56 to -29·24)
**Poisoning by other means**	14014·47 (8966·34 to 27180·43)	2131·96 (1043·19 to 3413·18)	33·86 (21·37 to 63·61)	2·88 (1·3 to 5·16)	-91·49 (-97·53 to -80·09)	5398·88 (3427·76 to 7691·23)	5768·99 (3624·01 to 8387·9)	12·05 (7·7 to 17·26)	9·96 (6·17 to 14·64)	-17·39 (-25·32 to -8·8)
**Exposure to mechanical forces**	41062·24 (34398·23 to 47391·83)	19121·55 (14233·73 to 22158·93)	89·83 (74·75 to 103·27)	28·46 (22·79 to 33·66)	-68·32 (-74·6 to -59·77)	78783·52 (52544·71 to 114064·9)	127109·68 (83905·38 to 187126·77)	187·53 (126·75 to 269·83)	179·13 (117·7 to 266·61)	-4·48 (-8·94 to -0·37)
**Unintentional firearm injuries**	2159·68 (829·33 to 3552·89)	327·86 (237·77 to 536·01)	4·43 (1·85 to 7·24)	0·74 (0·52 to 1·12)	-83·33 (-91·02 to -46·35)	1534·53 (1072·3 to 2061·58)	2804·13 (1979·87 to 3814·93)	3·62 (2·55 to 4·84)	3·92 (2·75 to 5·3)	8·29 (3·78 to 12·93)
**Other exposure to mechanical forces**	38902·56 (32316·52 to 45510·38)	18793·69 (13952·21 to 21859·08)	85·4 (70·96 to 98·75)	27·72 (22·15 to 32·72)	-67·54 (-74·41 to -58·87)	77248·99 (51395·01 to 112009·44)	124305·56 (81313·05 to 183691·21)	183·91 (123·94 to 265·46)	175·22 (114·76 to 261·38)	-4·73 (-9·3 to -0·58)
**Adverse effects of medical treatment**	6168·33 (5122·51 to 8848·59)	4639·79 (3068·51 to 5474·26)	16·76 (13·96 to 24·04)	7·34 (4·66 to 8·9)	-56·19 (-76·84 to -40·04)	434·08 (260·47 to 684·41)	503·54 (310·6 to 759·35)	1·09 (0·66 to 1·7)	1·13 (0·69 to 1·77)	4·12 (-2·09 to 9·79)
**Animal contact**	2178·26 (1940·26 to 2693·16)	1602·76 (1180·58 to 1858·06)	5·61 (5·01 to 6·91)	2·28 (1·75 to 2·62)	-59·28 (-73·15 to -51·42)	4095·91 (2713·38 to 5986·68)	5356·04 (3483·35 to 7986·36)	9·67 (6·47 to 14·15)	8·2 (5·36 to 12·19)	-15·27 (-19·19 to -11·94)
**Venomous animal contact**	1308·65 (1123·21 to 1537·89)	1235·12 (886·82 to 1458·3)	3·34 (2·87 to 3·9)	1·62 (1·21 to 1·88)	-51·58 (-67·4 to -40·46)	1771·29 (1161·67 to 2466·01)	2109·8 (1402·5 to 2983·67)	4·04 (2·66 to 5·63)	3·4 (2·21 to 4·82)	-15·82 (-20·98 to -10·51)
**Non-venomous animal contact**	869·61 (719·54 to 1178·79)	367·65 (282·75 to 434·69)	2·27 (1·87 to 3·07)	0·67 (0·53 to 0·79)	-70·57 (-81·15 to -61·58)	2324·61 (1470·81 to 3664·98)	3246·24 (1964·62 to 5177·64)	5·63 (3·58 to 8·86)	4·79 (2·94 to 7·62)	-14·87 (-19·34 to -11·5)
**Foreign body**	86184·81 (65392·74 to 99050·43)	20631·19 (18100·53 to 24584·81)	236·07 (181·88 to 275·15)	40·69 (34·76 to 49·21)	-82·76 (-86·09 to -72·88)	8758·22 (6089·42 to 12332·28)	12560·2 (8712·66 to 17745·76)	20·74 (14·53 to 28·7)	18·96 (12·97 to 26·97)	-8·6 (-12·85 to -4·66)
**Pulmonary aspiration and foreign body in airway**	85208·59 (64809·92 to 98234·14)	20324·73 (17838·58 to 24202·04)	233·77 (180·47 to 272·95)	40 (34·14 to 48·31)	-82·89 (-86·22 to -73·19)	1330·17 (941·55 to 1778·64)	1048·42 (735·4 to 1429·94)	3·21 (2·28 to 4·28)	1·63 (1·14 to 2·23)	-49·25 (-52·96 to -45·64)
**Foreign body in eyes**	NA	NA	NA	NA	NA	2085·93 (933·85 to 3774·63)	2759·49 (1447·31 to 4655·01)	4·64 (2·19 to 8·2)	4·65 (2·2 to 8·23)	0·33 (-1·05 to 1·45)
**Foreign body in other body part**	976·21 (544 to 1525·74)	306·46 (244·8 to 406·66)	2·3 (1·31 to 3·61)	0·69 (0·54 to 0·94)	-70·05 (-84·51 to -26·86)	5342·12 (3814·17 to 7220·47)	8752·29 (6168·31 to 12187·83)	12·9 (9·18 to 17·51)	12·68 (8·84 to 17·62)	-1·69 (-6·24 to 2·85)
**Environmental heat and cold exposure**	13259·91 (11014·87 to 22527·42)	7431·99 (3560·61 to 9120·76)	32·2 (27·02 to 56·28)	9·81 (4·95 to 11·98)	-69·54 (-83·26 to -59·88)	4353·96 (3055·71 to 6083·05)	5954·57 (4074·2 to 8381·25)	10·5 (7·37 to 14·62)	8·48 (5·84 to 12·05)	-19·28 (-23·35 to -15·29)

**Exposure to forces of nature**	8576·6 (7787·5 to 9436·71)	0 (0 to 0)	18·86 (17·12 to 20·75)	0 (0 to 0)	-100 (-100 to -100)	450·99 (329·76 to 610·97)	590·14 (425·64 to 815·42)	1 (0·74 to 1·34)	0·8 (0·56 to 1·1)	-20·43 (-33·23 to -5·34)
**Other unintentional injuries**	59462·78 (29377·13 to 67489·03)	6247·51 (5128·19 to 11002·95)	126·06 (64·77 to 142·35)	10·14 (8·32 to 18·59)	-91·96 (-93·93 to -69·99)	71621·53 (46023·29 to 105653·95)	86249·6 (54262·33 to 128871·5)	172·05 (110·48 to 252·43)	127·37 (79·82 to 191·88)	-25·97 (-29·74 to -22·25)
**Self-harm and interpersonal violence**	293575·51 (256257·34 to 531291·73)	531891·27 (345358·66 to 595069·48)	612·03 (533·64 to 1128·56)	806·1 (539·63 to 894·78)	31·71 (-45·53 to 63·27)	24991·71 (18872·1 to 31767·78)	38373·14 (28157·93 to 49683·31)	55·92 (42·14 to 71·36)	55·33 (41·1 to 70·95)	-1·05 (-5·19 to 3·23)
**Self-harm**	247777·81 (211214·17 to 488094·41)	503614·56 (318323·45 to 565443·55)	514·88 (437·68 to 1035·4)	756·49 (489·99 to 844·98)	46·93 (-44·8 to 88·01)	8468·76 (5858·24 to 11447·59)	19036·52 (13088·89 to 25737·46)	19·9 (13·81 to 26·92)	24·51 (16·8 to 33·34)	23·2 (12·77 to 34·25)
**Self-harm by firearm**	1893·6 (1099·4 to 4857·86)	867·75 (443·94 to 1321·84)	3·72 (2·19 to 9·78)	1·49 (0·77 to 2·25)	-59·92 (-85·94 to -16·89)	93·17 (66·14 to 125·19)	320·1 (229·41 to 431)	0·24 (0·17 to 0·32)	0·38 (0·28 to 0·51)	56·92 (47·92 to 66·13)
**Self-harm by other specified means**	245884·21 (209727·97 to 485168·04)	502746·81 (317917·55 to 564424·42)	511·16 (433·92 to 1027·29)	755 (488·98 to 843·39)	47·7 (-44·31 to 89·05)	8375·6 (5799·04 to 11334·41)	18716·42 (12863·99 to 25356·15)	19·65 (13·63 to 26·6)	24·13 (16·55 to 32·86)	22·78 (12·27 to 33·94)
**Interpersonal violence**	44418·57 (39973·27 to 48563·92)	28032·83 (24676·45 to 31558·98)	93·76 (85·06 to 102·02)	49·06 (42·87 to 55·78)	-47·67 (-55·3 to -39·27)	16445·87 (12432·33 to 21025·68)	19216·05 (14269 to 24627·78)	35·84 (27·06 to 45·68)	30·64 (23·16 to 39·35)	-14·49 (-17·55 to -11·67)
**Physical violence by firearm**	1164·18 (783·42 to 1527·52)	561·16 (425·39 to 687·46)	2·32 (1·59 to 2·99)	1·05 (0·75 to 1·29)	-54·91 (-67·62 to -37·84)	353·33 (242·12 to 477·1)	951·83 (660·04 to 1306·24)	0·78 (0·54 to 1·05)	1·42 (0·98 to 1·96)	82·29 (69·35 to 95·88)
**Physical violence by sharp object**	20539·3 (13853·06 to 24102·24)	6832·73 (5613·52 to 8696·57)	41·22 (27·96 to 48·32)	10·99 (8·98 to 13·99)	-73·33 (-79·71 to -60·69)	2062·26 (1397·47 to 2830·15)	1956·95 (1317·97 to 2750·53)	4·72 (3·21 to 6·45)	2·83 (1·91 to 3·97)	-40·11 (-43·74 to -36·42)
**Sexual violence**	NA	NA	NA	NA	NA	5821·8 (3785·31 to 8252·24)	5659·18 (3757·68 to 8036·76)	11·45 (7·47 to 16·2)	11·32 (7·38 to 16·01)	-1·13 (-1·29 to -0·99)
**Physical violence by other means**	22715·09 (19599·29 to 26745·9)	20638·94 (17412·59 to 23463·56)	50·22 (43·63 to 58·77)	37·02 (30·59 to 42·55)	-26·29 (-42·49 to -10·72)	8208·48 (5711·01 to 11398·95)	10648·08 (7326·04 to 14902·57)	18·89 (13·18 to 26·18)	15·08 (10·33 to 21·07)	-20·17 (-24·03 to -16·81)
**Conflict and terrorism**	1070·77 (972·25 to 1178·15)	0 (0 to 0)	2·71 (2·46 to 2·98)	0 (0 to 0)	-100 (-100 to -100)	77·08 (53 to 107·54)	120·23 (80·08 to 188·45)	0·18 (0·13 to 0·26)	0·17 (0·11 to 0·27)	-6·61 (-29·92 to 20·99)
**Executions and police conflict**	308·35 (267·47 to 365·74)	243·88 (226·78 to 260·76)	0·68 (0·58 to 0·82)	0·55 (0·51 to 0·6)	-18·91 (-33·84 to -2·28)	0 (0 to 0)	0·34 (0·23 to 0·49)	0 (0 to 0)	0 (0 to 0)	0 (0 to 0)

Appendix table 9. Age-standardised deaths and DALY rates, and percentage change from 1990 to 2019 attributable to risk factors for communicable, maternal, neonatal, and nutritional (CMNN) diseases

Risk factors	Death (95% UI)						DALYs (95% UI)					
	Age-standardised percent 1990	Age-standardised percent 2019	Change of age-standardised percent between 1990 to 2019 (%)	Age-standardised estimates 1990	Age-standardised estimates 2019	Percentage change of age-standardised rates between 1990 to 2019 (%)	Age-standardised percent 1990	Age-standardised percent 2019	Change of age-standardised percent between 1990 to 2019 (%)	Age-standardised estimates 1990	Age-standardised estimates 2019	Percentage change of age-standardised rates between 1990 to 2019 (%)
**Environmental/occupational risks**	13.58 (10.61 to 17.06)	23.53 (18.33 to 28.6)	73.32 (38.08 to 112.36)	9.45 (7.18 to 12.12)	6.96 (4.13 to 8.9)	-26.33 (-61.75 to -5.31)	7.68 (5.77 to 9.79)	8.97 (6.28 to 11.33)	16.77 (-13.24 to 53.69)	252.54 (191.74 to 324.78)	108.9 (74.15 to 134.78)	-56.88 (-72.69 to -42.36)
Unsafe water, sanitation, and handwashing	2.92 (1.64 to 4.73)	2.06 (1.11 to 3.24)	-29.26 (-54.92 to 0.43)	2.03 (1.12 to 3.35)	0.61 (0.31 to 1)	-70.03 (-81.5 to -55.36)	1.84 (1.18 to 2.64)	1.26 (0.74 to 1.97)	-31.25 (-50.43 to -6.95)	60.39 (38.03 to 87.74)	15.36 (8.68 to 24.82)	-74.57 (-81.73 to -65.89)
Unsafe water source	1.4 (0.44 to 2.96)	0.61 (0.16 to 1.45)	-56.78 (-77.12 to -14.45)	0.98 (0.3 to 2.07)	0.18 (0.05 to 0.43)	-81.75 (-90.08 to -66.04)	0.97 (0.35 to 1.75)	0.65 (0.21 to 1.32)	-32.78 (-61.03 to 10.06)	31.99 (11.45 to 58.5)	7.97 (2.52 to 16.25)	-75.1 (-85.61 to -59.71)
Unsafe sanitiona	NA	NA	NA	NA	NA	NA	NA	NA	NA	NA	NA	NA
No access to ahndwashing facility	NA	NA	NA	NA	NA	NA	NA	NA	NA	NA	NA	NA
Air pollution	5.01 (2.33 to 8.63)	10.09 (6.17 to 14.76)	101.57 (24.47 to 275.36)	3.48 (1.63 to 6.03)	2.99 (1.55 to 4.5)	-14.29 (-56.15 to 66.85)	3.26 (1.58 to 5.31)	4.23 (2.44 to 6.06)	29.49 (-24.46 to 154.12)	107.36 (52.09 to 175.4)	51.3 (29.72 to 74.33)	-52.22 (-73.49 to -6.23)
Particulate matter pollution	5.01 (2.33 to 8.63)	10.09 (6.17 to 14.76)	101.57 (24.47 to 275.36)	3.48 (1.63 to 6.03)	2.99 (1.55 to 4.5)	-14.29 (-56.15 to 66.85)	3.26 (1.58 to 5.31)	4.23 (2.44 to 6.06)	29.49 (-24.46 to 154.12)	107.36 (52.09 to 175.4)	51.3 (29.72 to 74.33)	-52.22 (-73.49 to -6.23)
Ambient ozone pollution	NA	NA	NA	NA	NA	NA	NA	NA	NA	NA	NA	NA
Non-optimal temperature	6.8 (4.97 to 8.85)	13.72 (9.77 to 17.62)	101.87 (53.56 to 119.97)	4.73 (3.45 to 6.28)	4.06 (2.31 to 5.46)	-14.12 (-58.16 to 0.88)	3.11 (2.21 to 4.17)	4.2 (2.73 to 5.75)	35.1 (-10.3 to 56.51)	102.12 (74.43 to 133.83)	50.98 (30.88 to 67.54)	-50.08 (-71.59 to -42.31)
High temperature	0.03 (-0.08 to 0.17)	0.11 (-0.38 to 0.6)	294.73 (-275.33 to 1081.2)	0.02 (-0.06 to 0.11)	0.03 (-0.11 to 0.18)	68.01 (-183.28 to 388.71)	0.01 (-0.04 to 0.08)	0.03 (-0.11 to 0.19)	165.19 (-215.19 to 675.04)	0.41 (-1.25 to 2.51)	0.4 (-1.37 to 2.22)	-2.39 (-146.58 to 179.85)

Low temperature	6.77 (4.86 to 8.88)	13.63 (9.51 to 17.75)	101.23 (52.31 to 119.05)	4.71 (3.39 to 6.34)	4.03 (2.29 to 5.5)	-14.4 (-58.74 to - 0.24)	3.1 (2.18 to 4.18)	4.18 (2.68 to 5.81)	34.66 (-10.62 to 55.37)	101.78 (72.5 to 134.59)	50.64 (30.41 to 68.11)	-50.24 (-71.69 to - 42.71)
Other environmental risks	NA	NA	NA	NA	NA	NA	NA	NA	NA	NA	NA	NA
Residential radon	NA	NA	NA	NA	NA	NA	NA	NA	NA	NA	NA	NA
Lead exposure	NA	NA	NA	NA	NA	NA	NA	NA	NA	NA	NA	NA
Occupational risks	NA	NA	NA	NA	NA	NA	NA	NA	NA	NA	NA	NA
Occupational carcinogens	NA	NA	NA	NA	NA	NA	NA	NA	NA	NA	NA	NA
Occupational asthmagens	NA	NA	NA	NA	NA	NA	NA	NA	NA	NA	NA	NA
Occupational particulate matter, gases, and fumes	NA	NA	NA	NA	NA	NA	NA	NA	NA	NA	NA	NA
Occupational noise	NA	NA	NA	NA	NA	NA	NA	NA	NA	NA	NA	NA
Occupational injuries	NA	NA	NA	NA	NA	NA	NA	NA	NA	NA	NA	NA
Occupational ergonomic factors	NA	NA	NA	NA	NA	NA	NA	NA	NA	NA	NA	NA
**Behavioral risks**	46.36 (42.51 to 50.38)	34.14 (29.37 to 38.87)	-26.36 (-35.19 to - 16.64)	32.22 (29.07 to 36.49)	10.04 (7.82 to 11.67)	-68.85 (-78.55 to - 63.7)	57.05 (53.19 to 60.26)	48.01 (45.14 to 50.98)	-15.85 (-21.29 to - 9.4)	1878.73 (1641.59 to 2166.44)	584.24 (500.52 to 685.73)	-68.9 (-73.31 to - 65.19)
Child and maternal malnutrition	14.12 (12.37 to 16.14)	8.24 (6.68 to 11.55)	-41.66 (-52.42 to - 20.9)	9.82 (8.39 to 11.78)	2.41 (2.04 to 2.81)	-75.48 (-80.6 to - 69.92)	39.62 (35.91 to 43.17)	36.51 (33.48 to 40.09)	-7.85 (-16.9 to 2.54)	1306.42 (1085.71 to 1563.74)	444.73 (370.04 to 544.73)	-65.96 (-71 to - 60.83)
Suboptimal breastfeeding	0.32 (0.23 to 0.43)	0.07 (0.04 to 0.1)	-79.52 (-86.27 to - 67.54)	0.22 (0.15 to 0.31)	0.02 (0.01 to 0.03)	-91.38 (-94.16 to - 87)	0.62 (0.44 to 0.83)	0.17 (0.12 to 0.23)	-72.36 (-80.38 to - 59.95)	20.3 (14.04 to 27.52)	2.07 (1.49 to 2.78)	-89.79 (-92.9 to - 85.1)

Child growth failure	3.8 (3.13 to 4.45)	0.79 (0.62 to 1.16)	-79.17 (-83.64 to -65.5)	2.64 (2.2 to 3.14)	0.23 (0.18 to 0.28)	-91.24 (-92.93 to -89.47)	3.32 (2.36 to 4.36)	1.52 (1.15 to 1.96)	-54.33 (-65.63 to -37.03)	109.1 (77.91 to 146.65)	18.45 (13.23 to 24.47)	-83.09 (-87.39 to -77.05)
Low birth weight and short gestation	10.1 (8.54 to 12.04)	7.38 (5.9 to 10.19)	-26.89 (-42.11 to -4.58)	7.03 (5.81 to 8.89)	2.16 (1.79 to 2.55)	-69.28 (-77.39 to -60.96)	21.59 (18.25 to 25.48)	22.84 (18.87 to 26.4)	5.75 (-9.94 to 23.86)	710.3 (600.56 to 876.47)	276.95 (238.44 to 321.28)	-61.01 (-69.88 to -52.28)
Iron deficiency	0.07 (0.03 to 0.12)	0.03 (0.01 to 0.06)	-53.15 (-64.7 to -27.84)	0.05 (0.02 to 0.08)	0.01 (0 to 0.02)	-80.28 (-84.61 to -74.61)	14.35 (10.3 to 19.24)	12.03 (8.24 to 16.83)	-16.15 (-32.31 to 3.94)	475.42 (313.14 to 703.21)	147.84 (91.31 to 236.93)	-68.9 (-75.13 to -61.39)
Vitamin A deficiency	0 (0 to 0.01)	0 (0 to 0)	-91.64 (-96.59 to -79.88)	0 (0 to 0)	0 (0 to 0)	-96.48 (-98.59 to -91.7)	0.06 (0.04 to 0.09)	0.03 (0.01 to 0.05)	-54.77 (-77.37 to -20.37)	2.03 (1.13 to 3.21)	0.34 (0.16 to 0.61)	-83.22 (-91.64 to -70.69)
Zinc deficiency	0 (0 to 0)	0 (0 to 0)	-77.4 (-100 to -43.74)	0 (0 to 0)	0 (0 to 0)	-90.47 (-100 to -75.98)	0 (0 to 0)	0 (0 to 0)	-47.22 (-100 to -2.35)	0.04 (0 to 0.14)	0.01 (0 to 0.03)	-80.46 (-100 to -62.74)
Tobacco	21.37 (18.54 to 24.51)	17.45 (14.05 to 21)	-18.32 (-32.09 to -2.55)	14.85 (12.86 to 17.11)	5.15 (3.34 to 6.41)	-65.32 (-80.6 to -57.84)	11.34 (9.48 to 13.28)	6.67 (4.94 to 8.31)	-41.23 (-54.39 to -30.09)	372.21 (326.76 to 420.44)	80.79 (57.02 to 97.19)	-78.29 (-85.59 to -74.51)
Smoking	18.4 (15.76 to 21.25)	14.57 (11.31 to 17.99)	-20.82 (-36.41 to -0.52)	12.78 (11.01 to 14.89)	4.3 (2.84 to 5.46)	-66.4 (-81.07 to -58.27)	9.24 (7.68 to 10.85)	5.51 (3.99 to 6.98)	-40.38 (-54.47 to -26.43)	303.24 (265.26 to 346.08)	66.75 (46.84 to 81.57)	-77.99 (-85.52 to -73.35)
Chewing tobacco	NA	NA	NA	NA	NA	NA	NA	NA	NA	NA	NA	NA
Secondhand smoke	3.38 (2.15 to 4.63)	3.39 (1.98 to 4.95)	0.49 (-21.67 to 17.13)	2.35 (1.47 to 3.35)	1 (0.51 to 1.51)	-57.24 (-77.95 to -47.42)	2.26 (1.46 to 3.07)	1.35 (0.82 to 1.95)	-40.28 (-52.29 to -29.88)	74.26 (48.62 to 100.86)	16.4 (9.68 to 23.56)	-77.92 (-84.62 to -73.69)
Alcohol use	17.16 (12.68 to 21.31)	10.54 (6.67 to 14.32)	-38.58 (-58.27 to -14.82)	11.93 (8.67 to 14.78)	3.09 (1.94 to 4.35)	-74.05 (-83.32 to -63.84)	9.7 (7.11 to 12.06)	4.41 (2.97 to 5.92)	-54.5 (-66.5 to -40.29)	318.44 (233.7 to 384.93)	53.43 (37.21 to 69.54)	-83.22 (-87.85 to -78.03)
Drug use	0.04 (0.02 to 0.06)	0.01 (0.01 to 0.02)	-75.54 (-86.53 to -41.28)	0.03 (0.01 to 0.04)	0 (0 to 0)	-89.73 (-94.14 to -80.3)	0.04 (0.02 to 0.06)	0.01 (0.01 to 0.02)	-65.44 (-79.88 to -29.92)	1.18 (0.56 to 1.92)	0.15 (0.1 to 0.22)	-87.19 (-92.61 to -74.4)
Dietary risks	NA	NA	NA	NA	NA	NA	NA	NA	NA	NA	NA	NA
Diet low in fruits	NA	NA	NA	NA	NA	NA	NA	NA	NA	NA	NA	NA
Diet low in vegetables	NA	NA	NA	NA	NA	NA	NA	NA	NA	NA	NA	NA

Diet low in legumes	NA	NA	NA	NA	NA	NA	NA	NA	NA	NA	NA	NA
Diet low in whole grains	NA	NA	NA	NA	NA	NA	NA	NA	NA	NA	NA	NA
Diet low in nuts and seeds	NA	NA	NA	NA	NA	NA	NA	NA	NA	NA	NA	NA
Diet low in milk	NA	NA	NA	NA	NA	NA	NA	NA	NA	NA	NA	NA
Diet high in red meat	NA	NA	NA	NA	NA	NA	NA	NA	NA	NA	NA	NA
Diet high in processed meat	NA	NA	NA	NA	NA	NA	NA	NA	NA	NA	NA	NA
Diet high in sugar-sweetened beverages	NA	NA	NA	NA	NA	NA	NA	NA	NA	NA	NA	NA
Diet low in fiber	NA	NA	NA	NA	NA	NA	NA	NA	NA	NA	NA	NA
Diet low in calcium	NA	NA	NA	NA	NA	NA	NA	NA	NA	NA	NA	NA
Diet low in seafood omega-3 fatty acids	NA	NA	NA	NA	NA	NA	NA	NA	NA	NA	NA	NA
Diet low in polyunsaturated fatty acids	NA	NA	NA	NA	NA	NA	NA	NA	NA	NA	NA	NA
Diet high in trans fatty acids	NA	NA	NA	NA	NA	NA	NA	NA	NA	NA	NA	NA
Diet high in sodium	NA	NA	NA	NA	NA	NA	NA	NA	NA	NA	NA	NA
Intimate partner violence	0 (0 to 0.01)	0 (0 to 0.01)	NA	0 (0 to 0)	0 (0 to 0)	NA	0 (0 to 0)	0.01 (0 to 0.02)	NA	0.08 (0 to 0.16)	0.11 (0.05 to 0.19)	NA
Childhood sexual abuse and bullying	NA	NA	NA	NA	NA	NA	NA	NA	NA	NA	NA	NA

Childhood sexual abuse	NA	NA	NA	NA	NA	NA	NA	NA	NA	NA	NA	NA
Unsafe sex	0.23 (0.21 to 0.26)	0.64 (0.56 to 0.85)	174.72 (135.41 to 290.4)	0.16 (0.15 to 0.18)	0.19 (0.17 to 0.2)	15.63 (4.06 to 25.9)	0.59 (0.43 to 0.86)	1.77 (1.37 to 2.32)	198.64 (141.56 to 291.97)	19.58 (13.29 to 30.61)	21.59 (15.4 to 30.58)	10.26 (-7.76 to 40.13)
Low physical activity	NA	NA	NA	NA	NA	NA	NA	NA	NA	NA	NA	NA
**Metabolic risks**	3.19 (1.8 to 4.91)	1.46 (0.69 to 2.51)	-54.32 (-65.59 to -30.39)	2.22 (1.24 to 3.43)	0.43 (0.21 to 0.7)	-80.79 (-85.29 to -75.21)	1.59 (0.95 to 2.34)	0.63 (0.35 to 0.97)	-60.38 (-68.28 to -49.14)	52.17 (31.89 to 75.25)	7.62 (4.39 to 11.52)	-85.4 (-88.07 to -82)
High fasting plasma glucose	3.19 (1.8 to 4.91)	1.46 (0.69 to 2.51)	-54.32 (-65.59 to -30.39)	2.22 (1.24 to 3.43)	0.43 (0.21 to 0.7)	-80.79 (-85.29 to -75.21)	1.59 (0.95 to 2.34)	0.63 (0.35 to 0.97)	-60.38 (-68.28 to -49.14)	52.17 (31.89 to 75.25)	7.62 (4.39 to 11.52)	-85.4 (-88.07 to -82)
High LDL cholesterol	NA	NA	NA	NA	NA	NA	NA	NA	NA	NA	NA	NA
High systolic blood pressure	NA	NA	NA	NA	NA	NA	NA	NA	NA	NA	NA	NA
High body-mass index	NA	NA	NA	NA	NA	NA	NA	NA	NA	NA	NA	NA
Low bone mineral density	NA	NA	NA	NA	NA	NA	NA	NA	NA	NA	NA	NA
Kidney dysfuntion	NA	NA	NA	NA	NA	NA	NA	NA	NA	NA	NA	NA

Appendix table 10. Age-standardised deaths and DALY rates, and percentage change from 1990 to 2019 attributable to risk factors for non-communicable diseases (NCDs)

Risk factors	Death (95% UI)						DALYs (95% UI)					
	Age-standardised percent 1990	Age-standardised percent 2019	Change of age-standardised percent between 1990 to 2019 (%)	Age-standardised estimates 1990	Age-standardised estimates 2019	Percentage change of age-standardised rates between 1990 to 2019 (%)	Age-standardised percent 1990	Age-standardised percent 2019	Change of age-standardised percent between 1990 to 2019 (%)	Age-standardised estimates 1990	Age-standardised estimates 2019	Percentage change of age-standardised rates between 1990 to 2019 (%)
**Environmental/occupational risks**	16.27 (12.56 to 20.23)	13.45 (11.32 to 15.49)	-17.36 (-33.86 to 6.36)	126.07 (97.37 to 157.16)	42.9 (36.39 to 49.92)	-65.97 (-72.97 to -55.04)	12.23 (9.42 to 14.91)	8.18 (7.12 to 9.33)	-33.09 (-45.67 to -13.76)	2931.8 (2249.32 to 3635.71)	1104.05 (950.6 to 1288.45)	-62.34 (-69.67 to -51.36)
Unsafe water, sanitation, and handwashing	NA	NA	NA	NA	NA	NA	NA	NA	NA	NA	NA	NA
Unsafe water source	NA	NA	NA	NA	NA	NA	NA	NA	NA	NA	NA	NA
Unsafe sanitiona	NA	NA	NA	NA	NA	NA	NA	NA	NA	NA	NA	NA
No access to ahndwashing facility	NA	NA	NA	NA	NA	NA	NA	NA	NA	NA	NA	NA
Air pollution	9.28 (5.29 to 13.56)	7.69 (6.14 to 9.21)	-17.12 (-44.89 to 43.73)	71.87 (41.13 to 105.43)	24.53 (19.78 to 29.65)	-65.87 (-77.32 to -40.22)	6.76 (3.86 to 9.84)	4.2 (3.35 to 5.12)	-37.81 (-58.01 to 6.96)	1620.85 (930.1 to 2365.04)	566.52 (462.74 to 691.48)	-65.05 (-76.7 to -39.51)
Particulate matter pollution	9.07 (5.07 to 13.42)	7.2 (5.76 to 8.65)	-20.63 (-47.91 to 41.78)	70.27 (39.7 to 104.19)	22.97 (18.47 to 28.07)	-67.31 (-78.5 to -41.35)	6.67 (3.78 to 9.72)	4.07 (3.25 to 4.99)	-39.01 (-59.47 to 6.59)	1598.21 (903.16 to 2342.4)	547.91 (446.31 to 671.08)	-65.72 (-77.31 to -39.94)
Ambient ozone pollution	0.25 (0.1 to 0.43)	0.6 (0.27 to 0.95)	140.31 (81.55 to 257.47)	1.94 (0.79 to 3.33)	1.92 (0.9 to 3.04)	-1.18 (-24.68 to 49.92)	0.11 (0.05 to 0.19)	0.17 (0.08 to 0.27)	49.13 (16.11 to 115.79)	27.37 (11.88 to 46.43)	22.9 (10.7 to 36.73)	-16.33 (-34.81 to 21.3)
Non-optimal temperature	6 (4.59 to 7.52)	4.47 (3.57 to 5.53)	-25.41 (-34.6 to -14.38)	46.46 (35.55 to 58.05)	14.27 (11.31 to 17.75)	-69.29 (-73.05 to -63.3)	3.35 (2.51 to 4.28)	1.5 (1.12 to 1.99)	-55.21 (-61.14 to -45.85)	803.27 (616.71 to 1000.69)	201.9 (161.85 to 251.31)	-74.86 (-77.83 to -68.6)
High temperature	0.03 (-0.02 to 0.09)	0.06 (0.01 to 0.14)	100.28 (-848.93 to 913.55)	0.25 (-0.12 to 0.71)	0.2 (0.04 to 0.44)	-17.52 (-406.72 to 310.76)	0.02 (-0.01 to 0.05)	0.02 (0 to 0.04)	20.28 (-520.37 to 533.56)	4.2 (-2.25 to 12.3)	2.84 (0.47 to 6.24)	-32.46 (-339.15 to 258.14)

Low temperature	5.97 (4.52 to 7.51)	4.42 (3.49 to 5.49)	-26.01 (-35.13 to -15.02)	46.23 (35.11 to 58.01)	14.08 (11.11 to 17.58)	-69.53 (-73.26 to -63.68)	3.34 (2.47 to 4.27)	1.48 (1.1 to 1.97)	-55.56 (-61.38 to -46.38)	799.3 (609.17 to 1000.96)	199.32 (158.97 to 249.72)	-75.06 (-77.94 to -68.95)
Other environmental risks	1.56 (0.63 to 2.55)	1.17 (0.45 to 2.02)	-24.82 (-47.77 to 17.41)	12.06 (4.93 to 19.9)	3.73 (1.41 to 6.4)	-69.04 (-78.41 to -51.28)	1.06 (0.45 to 1.74)	0.47 (0.17 to 0.84)	-55.63 (-70.48 to -28.96)	252.97 (109.86 to 410.89)	63.04 (22.13 to 110.92)	-75.08 (-83.2 to -61.19)
Residential radon	0.17 (0.02 to 0.43)	0.45 (0.06 to 1.18)	169.73 (137.27 to 195.74)	1.28 (0.16 to 3.36)	1.43 (0.18 to 3.79)	11.02 (-1.11 to 23.89)	0.13 (0.02 to 0.35)	0.2 (0.03 to 0.53)	50.34 (32.41 to 67.64)	32 (3.97 to 83.61)	27 (3.45 to 71.55)	-15.61 (-23.74 to -6.42)
Lead exposure	1.39 (0.49 to 2.34)	0.72 (0.23 to 1.31)	-48.01 (-57.75 to -38.17)	10.77 (3.79 to 18.16)	2.31 (0.74 to 4.19)	-78.58 (-82.71 to -72.9)	0.92 (0.34 to 1.55)	0.27 (0.08 to 0.51)	-70.99 (-79.07 to -64.95)	220.97 (82.65 to 372.02)	36.03 (10.45 to 67.36)	-83.69 (-88.16 to -80.12)
Occupational risks	0.96 (0.79 to 1.13)	1.51 (1.16 to 1.9)	57.81 (27.44 to 92.29)	7.42 (6.12 to 8.75)	4.82 (3.73 to 6.11)	-35 (-47.07 to -17.98)	2.03 (1.72 to 2.33)	2.55 (2.2 to 2.91)	25.61 (15.51 to 37.05)	488.63 (382.23 to 598.04)	345.99 (266.5 to 433.61)	-29.19 (-35.01 to -23.34)
Occupational carcinogens	0.34 (0.23 to 0.47)	0.91 (0.63 to 1.25)	167.45 (97.22 to 238.51)	2.65 (1.75 to 3.61)	2.92 (1.98 to 4)	10.26 (-17.51 to 41.51)	0.28 (0.18 to 0.38)	0.4 (0.26 to 0.56)	44.05 (11.41 to 81.19)	66.04 (43.73 to 89.84)	53.46 (36.38 to 73.42)	-19.05 (-37.23 to 2.22)
Occupational asthmagens	0.13 (0.1 to 0.15)	0.02 (0.02 to 0.03)	-83.87 (-87.47 to -76.55)	1 (0.76 to 1.17)	0.07 (0.05 to 0.08)	-93.36 (-94.87 to -90.05)	0.15 (0.13 to 0.18)	0.09 (0.06 to 0.12)	-42.48 (-55.88 to -25.98)	36.32 (29.26 to 43.69)	11.82 (7.89 to 17.36)	-67.44 (-76.1 to -56.65)
Occupational particulate matter, gases, and fumes	0.49 (0.36 to 0.6)	0.58 (0.43 to 0.74)	18.37 (-5.4 to 51.31)	3.77 (2.78 to 4.69)	1.84 (1.38 to 2.39)	-51.29 (-61.01 to -36.36)	0.35 (0.28 to 0.42)	0.32 (0.25 to 0.4)	-8.39 (-22.05 to 9.71)	83.66 (66.78 to 98.1)	43.05 (34.72 to 52.32)	-48.53 (-56.17 to -37.71)
Occupational noise	NA	NA	NA	NA	NA	NA	0.18 (0.13 to 0.24)	0.26 (0.2 to 0.34)	46.81 (33.73 to 62.75)	43.07 (29.42 to 61)	35.68 (24.03 to 52.17)	-17.17 (-22.29 to -11.93)
Occupational injuries	NA	NA	NA	NA	NA	NA	NA	NA	NA	NA	NA	NA
Occupational ergonomic factors	NA	NA	NA	NA	NA	NA	1.08 (0.8 to 1.37)	1.49 (1.15 to 1.84)	37.96 (23.99 to 56.68)	259.55 (175.82 to 357.8)	201.98 (137.06 to 273.28)	-22.18 (-29.03 to -14.59)
**Behavioral risks**	43.89 (39.59 to 49.05)	37.75 (34.03 to 41.71)	-14 (-19.28 to -8.42)	340.02 (305.84 to 380.17)	120.43 (108.15 to 135.77)	-64.58 (-67.05 to -60.45)	35.15 (31.59 to 39.2)	24 (21.36 to 26.73)	-31.7 (-36.17 to -27.11)	8423.7 (7642.7 to 9271.49)	3237.14 (2891.05 to 3617.31)	-61.57 (-64.72 to -57.7)
Child and maternal malnutrition	0 (0 to 0.01)	0 (0 to 0.01)	22.28 (-43.9 to 168.39)	0.03 (0.01 to 0.05)	0.01 (0 to 0.02)	-49.78 (-77.17 to 8.77)	0.01 (0 to 0.02)	0.01 (0 to 0.02)	-10.46 (-59.69 to 96.84)	2.28 (0.8 to 4.48)	1.15 (0.42 to 2.14)	-49.78 (-77.17 to 8.77)
Suboptimal breastfeeding	NA	NA	NA	NA	NA	NA	NA	NA	NA	NA	NA	NA

Child growth failure	NA	NA	NA	NA	NA	NA	NA	NA	NA	NA	NA	NA
Low birth weight and short gestation	0 (0 to 0.01)	0 (0 to 0.01)	22.28 (-43.9 to 168.39)	0.03 (0.01 to 0.05)	0.01 (0 to 0.02)	-49.78 (-77.17 to 8.77)	0.01 (0 to 0.02)	0.01 (0 to 0.02)	-10.46 (-59.69 to 96.84)	2.28 (0.8 to 4.48)	1.15 (0.42 to 2.14)	-49.78 (-77.17 to 8.77)
Iron deficiency	NA	NA	NA	NA	NA	NA	NA	NA	NA	NA	NA	NA
Vitamin A deficiency	NA	NA	NA	NA	NA	NA	NA	NA	NA	NA	NA	NA
Zinc deficiency	NA	NA	NA	NA	NA	NA	NA	NA	NA	NA	NA	NA
Tobacco	19.44 (18.18 to 20.71)	17.99 (16.89 to 19.05)	-7.46 (-14.42 to 0.19)	150.62 (140.28 to 161.1)	57.4 (53.07 to 62.34)	-61.89 (-65.11 to -57.19)	16.2 (14.78 to 17.62)	10.91 (9.54 to 12.3)	-32.68 (-38.01 to -27.12)	3882.42 (3624.14 to 4146.89)	1469.46 (1331.99 to 1610.96)	-62.15 (-65.14 to -57.93)
Smoking	17.8 (16.59 to 19.11)	17.15 (16.05 to 18.27)	-3.65 (-11.48 to 4.96)	137.9 (127.55 to 148.32)	54.72 (50.47 to 59.43)	-60.32 (-63.93 to -55.27)	14.96 (13.6 to 16.35)	10.31 (8.97 to 11.66)	-31.06 (-36.93 to -24.94)	3585.44 (3339.19 to 3840.53)	1389.66 (1263.21 to 1522.59)	-61.24 (-64.47 to -56.8)
Chewing tobacco	0.01 (0.01 to 0.01)	0.01 (0.01 to 0.02)	42.24 (0.3 to 114.21)	0.07 (0.04 to 0.1)	0.04 (0.02 to 0.06)	-41.43 (-58.7 to -8.84)	0.01 (0 to 0.01)	0.01 (0 to 0.01)	-7.57 (-35.44 to 46.41)	1.68 (0.99 to 2.55)	0.87 (0.55 to 1.34)	-48.14 (-63.81 to -15.53)
Secondhand smoke	2.18 (1.73 to 2.58)	1.2 (0.91 to 1.5)	-44.93 (-53.86 to -36.26)	16.86 (13.46 to 20.05)	3.82 (2.88 to 4.78)	-77.32 (-80.93 to -73.1)	1.73 (1.37 to 2.07)	0.82 (0.6 to 1.06)	-52.65 (-61.5 to -44.85)	414.2 (331.53 to 494.88)	110.42 (79.84 to 144.37)	-73.34 (-78.48 to -68.24)
Alcohol use	8.67 (7.07 to 10.28)	6.9 (5.62 to 8.18)	-20.34 (-35.12 to -1.57)	67.15 (54.66 to 79.78)	22.03 (17.86 to 26.36)	-67.19 (-73.8 to -59.07)	9.57 (8.17 to 10.88)	6.18 (5.24 to 7.15)	-35.39 (-44.24 to -24.45)	2294.88 (1945.16 to 2620.31)	834.56 (701.3 to 977.61)	-63.63 (-69.04 to -56.94)
Drug use	0.3 (0.2 to 0.44)	0.75 (0.53 to 1.05)	148.2 (96.56 to 216)	2.34 (1.57 to 3.44)	2.39 (1.68 to 3.31)	2.2 (-19.48 to 31.01)	0.73 (0.56 to 0.92)	1.25 (1.01 to 1.52)	71.92 (47.07 to 103.69)	174.86 (131.42 to 229.57)	169.26 (131.36 to 214.27)	-3.2 (-17.17 to 13.67)
Dietary risks	20.37 (14.74 to 27.05)	14.31 (10.75 to 19)	-29.72 (-37.31 to -19.59)	157.79 (114.47 to 209.53)	45.67 (34.25 to 60.95)	-71.06 (-74.11 to -65.68)	13.06 (9.28 to 17.43)	6.95 (5.27 to 8.99)	-46.76 (-52.35 to -37.78)	3128.76 (2272.28 to 4153.47)	937.12 (728.09 to 1202.77)	-70.05 (-73.51 to -63.99)
Diet low in fruits	3.01 (1.96 to 4.16)	1.66 (1.12 to 2.22)	-44.75 (-56.01 to -30.83)	23.33 (15.25 to 32.31)	5.31 (3.58 to 7.16)	-77.25 (-81.85 to -70.85)	2.17 (1.45 to 3.03)	0.84 (0.56 to 1.16)	-61.26 (-69.51 to -50.26)	518.6 (350.45 to 715.44)	113.01 (76.64 to 155.69)	-78.21 (-82.77 to -71.56)
Diet low in vegetables	0.56 (0.3 to 0.85)	0.49 (0.27 to 0.76)	-11.95 (-31.05 to 13.88)	4.33 (2.34 to 6.63)	1.57 (0.85 to 2.39)	-63.74 (-71.66 to -52.19)	0.2 (0.12 to 0.32)	0.15 (0.07 to 0.24)	-26.97 (-44.43 to -3.96)	48.59 (28.04 to 77.83)	19.93 (10.21 to 33.13)	-58.98 (-68.55 to -46.32)

Diet low in legumes	2.25 (0.38 to 3.79)	1.46 (0.37 to 2.43)	-35.23 (-45.59 to 12.43)	17.45 (2.93 to 29.29)	4.65 (1.18 to 7.74)	-73.34 (-77.74 to -54.44)	1.33 (0.19 to 2.25)	0.54 (0.13 to 0.93)	-59.22 (-66.05 to -24.47)	319.69 (45.93 to 524.54)	73.25 (15.52 to 122.32)	-77.09 (-80.81 to -57.62)
Diet low in whole grains	2.45 (1.32 to 3.3)	2.29 (1.45 to 2.98)	-6.39 (-22.17 to 21.44)	18.97 (10.31 to 25.51)	7.31 (4.64 to 9.56)	-61.47 (-68.06 to -49.72)	1.53 (0.86 to 2.07)	1.06 (0.72 to 1.39)	-30.87 (-44.02 to -6.51)	366.3 (198.15 to 493.02)	142.38 (93.52 to 185.22)	-61.13 (-68.44 to -47.31)
Diet low in nuts and seeds	1.2 (0.48 to 1.76)	0.61 (0.27 to 0.95)	-49.09 (-62.09 to -31.74)	9.27 (3.68 to 13.65)	1.94 (0.87 to 3.04)	-79.04 (-84.36 to -71.47)	0.68 (0.26 to 1.04)	0.22 (0.09 to 0.38)	-67.38 (-78.19 to -47.63)	163.95 (63.71 to 250.14)	30.1 (12.56 to 50.21)	-81.64 (-87.75 to -70.55)
Diet low in milk	0.28 (0.19 to 0.37)	0.77 (0.5 to 1.05)	170.18 (131.05 to 203.1)	2.21 (1.49 to 2.91)	2.45 (1.58 to 3.36)	11.23 (-3.71 to 25.4)	0.21 (0.14 to 0.28)	0.37 (0.23 to 0.51)	74.33 (49.85 to 98.51)	50.38 (33.94 to 66.15)	49.33 (31.59 to 66.94)	-2.09 (-15.54 to 10.45)
Diet high in red meat	1.9 (0.89 to 2.84)	1.8 (1.16 to 2.48)	-5.04 (-25.16 to 47.55)	14.72 (6.91 to 21.97)	5.76 (3.74 to 7.84)	-60.88 (-69.25 to -38.3)	1.44 (0.68 to 2.15)	1.32 (0.98 to 1.71)	-7.89 (-29.3 to 55.41)	344.15 (163.95 to 502.66)	178.49 (127.17 to 231.98)	-48.14 (-61.07 to -13.75)
Diet high in processed meat	0.41 (0.18 to 0.83)	0.61 (0.35 to 0.95)	49.84 (0.91 to 155.34)	3.15 (1.36 to 6.46)	1.94 (1.11 to 3.03)	-38.32 (-58.67 to 5.96)	0.37 (0.18 to 0.69)	0.51 (0.34 to 0.69)	36.34 (-11.53 to 120.83)	89.68 (43.67 to 164.86)	68.93 (44.04 to 96.76)	-23.14 (-50.52 to 24.77)
Diet high in sugar-sweetened beverages	0.33 (0.2 to 0.48)	0.29 (0.17 to 0.42)	-11 (-43.48 to 35.46)	2.55 (1.56 to 3.68)	0.93 (0.55 to 1.33)	-63.34 (-77.01 to -44.52)	0.21 (0.13 to 0.29)	0.2 (0.11 to 0.27)	-6.3 (-40.5 to 35.36)	49.9 (31.3 to 70.12)	26.38 (14.28 to 38.32)	-47.14 (-65.71 to -22.85)
Diet low in fiber	3.13 (1.78 to 4.45)	2.36 (1.52 to 3.16)	-24.55 (-36.33 to -4.64)	24.28 (13.82 to 34.52)	7.54 (4.86 to 10.14)	-68.92 (-73.86 to -59.27)	1.94 (1.03 to 2.85)	1.12 (0.73 to 1.51)	-42.25 (-51.11 to -25.2)	464.43 (257.69 to 674.06)	150.89 (98.07 to 203.28)	-67.51 (-72.99 to -56.7)
Diet low in calcium	0.26 (0.19 to 0.34)	0.62 (0.41 to 0.88)	136.67 (98.73 to 171.43)	2.02 (1.49 to 2.66)	1.97 (1.3 to 2.8)	-2.52 (-17.91 to 12.39)	0.18 (0.13 to 0.25)	0.27 (0.17 to 0.4)	44.95 (18.82 to 71.84)	44.26 (32.44 to 58.65)	36.04 (22.98 to 52.27)	-18.57 (-32.56 to -3.87)
Diet low in seafood omega-3 fatty acids	0.68 (0.23 to 0.92)	0.36 (0.2 to 0.48)	-46.37 (-55.1 to -15.84)	5.26 (1.72 to 7.17)	1.16 (0.65 to 1.54)	-77.92 (-81.55 to -64.19)	0.42 (0.13 to 0.58)	0.14 (0.08 to 0.19)	-66.38 (-71.79 to -43.86)	99.82 (31.89 to 135.46)	18.84 (10.41 to 24.6)	-81.13 (-84.09 to -68.4)
Diet low in polyunsaturated fatty acids	0.56 (0.06 to 1.22)	0.13 (0.03 to 0.29)	-77.48 (-84.32 to -49.14)	4.31 (0.5 to 9.4)	0.4 (0.09 to 0.93)	-90.73 (-93.58 to -78.73)	0.34 (0.04 to 0.76)	0.04 (0.01 to 0.1)	-87.41 (-91.26 to -68.81)	82.69 (8.75 to 182.59)	5.85 (1.39 to 13.65)	-92.93 (-95.15 to -82.87)
Diet high in trans fatty acids	1.85 (0.13 to 2.5)	1.01 (0.07 to 1.4)	-45.43 (-52.35 to -37.38)	14.35 (0.99 to 19.42)	3.22 (0.21 to 4.46)	-77.55 (-80.58 to -72.96)	1.13 (0.07 to 1.56)	0.39 (0.02 to 0.56)	-65.51 (-69.89 to -58.54)	271.04 (17.91 to 365.56)	52.46 (3.28 to 71.89)	-80.65 (-82.97 to -76.07)
Diet high in sodium	7.92 (1.51 to 16.18)	4.62 (0.84 to 9.89)	-41.63 (-56.02 to -22.48)	61.35 (11.81 to 125.67)	14.76 (2.7 to 31.73)	-75.94 (-81.84 to -67.55)	5 (0.94 to 10.3)	1.98 (0.38 to 4.17)	-60.37 (-69.9 to -47.16)	1198.09 (229.07 to 2482.92)	266.92 (51.09 to 572.54)	-77.72 (-83.12 to -69.79)
Intimate partner violence	NA	NA	NA	NA	NA	NA	0.05 (0 to 0.13)	0.08 (0 to 0.2)	48.3 (21.42 to 82.21)	12.98 (0.04 to 30.54)	10.86 (0.04 to 26.12)	-16.36 (-30.33 to 0.26)
Childhood sexual abuse and bullying	0.04 (0.01 to 0.09)	0.04 (0.01 to 0.09)	1.88 (-31.86 to 70.31)	0.29 (0.04 to 0.66)	0.12 (0.02 to 0.29)	-58.01 (-69.88 to -27.38)	0.35 (0.15 to 0.64)	0.54 (0.25 to 0.96)	54.62 (28.5 to 92.41)	84.86 (35.22 to 163.28)	74.05 (31.91 to 135.07)	-12.75 (-26.67 to 7.37)

Childhood sexual abuse	0.04 (0.01 to 0.09)	0.04 (0.01 to 0.09)	1.88 (-31.86 to 70.31)	0.29 (0.04 to 0.66)	0.12 (0.02 to 0.29)	-58.01 (-69.88 to -27.38)	0.24 (0.08 to 0.5)	0.32 (0.12 to 0.63)	32.85 (13.52 to 72.02)	58.11 (18.29 to 123.09)	43.59 (15.74 to 86.65)	-24.99 (-36 to - 3.29)
Unsafe sex	0.44 (0.39 to 0.63)	0.47 (0.36 to 0.58)	7.49 (-26.1 to 33.13)	3.38 (3.04 to 4.84)	1.5 (1.19 to 1.86)	-55.72 (-68.41 to -45.21)	0.44 (0.38 to 0.57)	0.3 (0.23 to 0.38)	-33.44 (-47.02 to -15.58)	106.47 (93.42 to 132.16)	39.78 (32.54 to 49.93)	-62.63 (-69.2 to - 52.63)
Low physical activity	2.14 (0.88 to 4.15)	2.1 (1.09 to 3.56)	-2.21 (-20.86 to 38.5)	16.61 (6.81 to 31.75)	6.69 (3.5 to 11.48)	-59.74 (-67.38 to -43.37)	1.11 (0.48 to 2.19)	0.89 (0.46 to 1.57)	-19.27 (-37 to 16.17)	264.93 (116.08 to 525.08)	120.34 (61.24 to 212.97)	-54.58 (-64.85 to -33.87)
**Metabolic risks**	40.4 (35.73 to 44.93)	31.69 (26.92 to 36.89)	-21.56 (-29.26 to -12.99)	312.99 (276.58 to 347.19)	101.11 (85.45 to 118.78)	-67.7 (-71.04 to -62.31)	26.89 (23.61 to 30.33)	16.96 (14.27 to 19.95)	-36.95 (-42.07 to -29.55)	6444.64 (5735.25 to 7118.93)	2287.82 (1920.34 to 2726.19)	-64.5 (-68.21 to -58.91)
High fasting plasma glucose	10.35 (8.18 to 13.49)	14.56 (11.04 to 19.46)	40.64 (16.64 to 66.08)	80.17 (63.15 to 104.71)	46.46 (35.47 to 62.45)	-42.06 (-52.06 to -31.09)	7.85 (6.57 to 9.48)	8.72 (7.27 to 10.64)	11.03 (-4.44 to 25.79)	1883.22 (1575.89 to 2252.72)	1177.54 (938.23 to 1466.47)	-37.47 (-46.46 to -28.25)
High LDL cholesterol	8.3 (5.8 to 11.72)	6.01 (3.91 to 9.04)	-27.58 (-38.05 to -16.46)	64.32 (44.83 to 90.93)	19.18 (12.43 to 28.67)	-70.18 (-74.61 to -63.94)	5.28 (4.02 to 6.86)	2.5 (1.79 to 3.5)	-52.6 (-59.02 to -42.89)	1264.75 (976.52 to 1658.81)	336.86 (248.08 to 458.92)	-73.37 (-76.87 to -67.22)
High systolic blood pressure	27.58 (23.46 to 31.31)	14.16 (11.03 to 17.08)	-48.68 (-56.23 to -39.83)	213.68 (181.67 to 242.88)	45.15 (35.43 to 55.71)	-78.87 (-82.06 to -73.8)	16.96 (14.43 to 19.5)	5.92 (4.69 to 7.32)	-65.11 (-69.5 to - 56.16)	4062.22 (3510.71 to 4545.92)	796.35 (663.09 to 958.39)	-80.4 (-82.88 to -74.72)
High body-mass index	6.22 (2.12 to 11.8)	7.17 (3.23 to 11.82)	15.19 (-5.62 to 76.98)	48.19 (16.52 to 91.75)	22.87 (10.37 to 37.65)	-52.54 (-61.42 to -25.55)	5.54 (1.94 to 10.14)	5.57 (2.72 to 8.8)	0.39 (-16.73 to 50.51)	1328.83 (469.09 to 2423.44)	752.12 (362.34 to 1202.67)	-43.4 (-54.09 to -14.34)
Low bone mineral density	NA	NA	NA	NA	NA	NA	NA	NA	NA	NA	NA	NA
Kidney dysfuntion	NA	NA	NA	NA	NA	NA	NA	NA	NA	NA	NA	NA

Appendix table 11. Age-standardised deaths and DALY rates, and percentage change from 1990 to 2019 attributable to risk factors for injuries

	**Death (95% UI)**						**DALYs (95% UI)**					
**Risk factors**	**Age-standardised percent 1990**	**Age-standardised percent 2019**	**Change of age-standardised percent between 1990 to 2019 (%)**	**Age-standardised estimates 1990**	**Age-standardised estimates 2019**	**Percentage change of age-standardised rates between 1990 to 2019 (%)**	**Age-standardised percent 1990**	**Age-standardised percent 2019**	**Change of age-standardised percent between 1990 to 2019 (%)**	**Age-standardised estimates 1990**	**Age-standardised estimates 2019**	**Percentage change of age-standardised rates between 1990 to 2019 (%)**

**Environmental/occupational risks**	10.27 (7.59 to 12.58)	-2.44 (-4.14 to -0.83)	-123.73 (-148.48 to -107)	8.64 (6.83 to 10.41)	-1.04 (-1.82 to -0.33)	-112.07 (-125.45 to -103.43)	12.58 (10.42 to 14.8)	0.58 (-0.96 to 2.02)	-95.36 (-108.71 to -84.63)	624.29 (502.43 to 747.24)	14.68 (-23.87 to 52.07)	-97.65 (-104.17 to -92.26)
Unsafe water, sanitation, and handwashing	NA	NA	NA	NA	NA	NA	NA	NA	NA	NA	NA	NA
Unsafe water source	NA	NA	NA	NA	NA	NA	NA	NA	NA	NA	NA	NA
Unsafe sanitiona	NA	NA	NA	NA	NA	NA	NA	NA	NA	NA	NA	NA
No access to ahndwashing facility	NA	NA	NA	NA	NA	NA	NA	NA	NA	NA	NA	NA
Air pollution	NA	NA	NA	NA	NA	NA	NA	NA	NA	NA	NA	NA
Particulate matter pollution	NA	NA	NA	NA	NA	NA	NA	NA	NA	NA	NA	NA
Ambient ozone pollution	NA	NA	NA	NA	NA	NA	NA	NA	NA	NA	NA	NA
Non-optimal temperature	-5.12 (-7.1 to -3.13)	-4.31 (-5.94 to -2.69)	-15.75 (-37.47 to 15.54)	-4.31 (-5.94 to -2.64)	-1.84 (-2.59 to -1.13)	-57.41 (-69.63 to -39.03)	-4.41 (-6.02 to -2.83)	-2.98 (-4.2 to -1.86)	-32.45 (-49.87 to -8.57)	-218.36 (-294.97 to -140.1)	-72.36 (-100.23 to -46.14)	-66.86 (-75.45 to -53.8)
High temperature	0.1 (0.04 to 0.21)	0.14 (0.06 to 0.24)	31.63 (-13.06 to 145.41)	0.09 (0.03 to 0.18)	0.06 (0.03 to 0.1)	-33.53 (-58.46 to 26.78)	0.09 (0.03 to 0.18)	0.1 (0.04 to 0.16)	4.07 (-30.95 to 93.52)	4.56 (1.49 to 8.89)	2.33 (1.09 to 4.01)	-48.95 (-66.97 to -4.74)
Low temperature	-5.24 (-7.25 to -3.27)	-4.46 (-6.16 to -2.8)	-14.8 (-36.76 to 16.78)	-4.42 (-6.04 to -2.72)	-1.9 (-2.68 to -1.17)	-56.93 (-69.16 to -38.65)	-4.52 (-6.16 to -2.91)	-3.08 (-4.35 to -1.92)	-31.72 (-49.28 to -7.76)	-223.76 (-300.04 to -145.24)	-74.95 (-103.41 to -48.01)	-66.5 (-75.04 to -53.1)
Other environmental risks	NA	NA	NA	NA	NA	NA	NA	NA	NA	NA	NA	NA
Residential radon	NA	NA	NA	NA	NA	NA	NA	NA	NA	NA	NA	NA
Lead exposure	NA	NA	NA	NA	NA	NA	NA	NA	NA	NA	NA	NA

Occupational risks	14.65 (12.21 to 16.3)	1.79 (1.34 to 2.34)	-87.81 (-91 to -82.66)	12.33 (11.2 to 13.48)	0.76 (0.6 to 0.93)	-93.85 (-95.17 to -92.41)	16.36 (14.7 to 17.98)	3.49 (2.66 to 4.45)	-78.68 (-83.94 to -72.6)	811.23 (713.47 to 919.27)	85.23 (61.05 to 116.44)	-89.49 (-92.26 to -86.26)
Occupational carcinogens	NA	NA	NA	NA	NA	NA	NA	NA	NA	NA	NA	NA
Occupational asthmagens	NA	NA	NA	NA	NA	NA	NA	NA	NA	NA	NA	NA
Occupational particulate matter, gases, and fumes	NA	NA	NA	NA	NA	NA	NA	NA	NA	NA	NA	NA
Occupational noise	NA	NA	NA	NA	NA	NA	NA	NA	NA	NA	NA	NA
Occupational injuries	14.65 (12.21 to 16.3)	1.79 (1.34 to 2.34)	-87.81 (-91 to -82.66)	12.33 (11.2 to 13.48)	0.76 (0.6 to 0.93)	-93.85 (-95.17 to -92.41)	16.36 (14.7 to 17.98)	3.49 (2.66 to 4.45)	-78.68 (-83.94 to -72.6)	811.23 (713.47 to 919.27)	85.23 (61.05 to 116.44)	-89.49 (-92.26 to -86.26)
Occupational ergonomic factors	NA	NA	NA	NA	NA	NA	NA	NA	NA	NA	NA	NA
**Behavioral risks**	12.61 (9.43 to 16.15)	17.72 (12.53 to 22.86)	40.52 (3.77 to 83.47)	10.67 (7.86 to 15)	7.57 (5.08 to 9.99)	-29.07 (-58.54 to -3.71)	11.26 (8.44 to 14.4)	15.75 (11.39 to 20.11)	39.96 (6.82 to 79.62)	558.79 (413.82 to 755.92)	383.82 (274.4 to 493.39)	-31.31 (-53.16 to -11.95)
Child and maternal malnutrition	NA	NA	NA	NA	NA	NA	NA	NA	NA	NA	NA	NA
Suboptimal breastfeeding	NA	NA	NA	NA	NA	NA	NA	NA	NA	NA	NA	NA
Child growth failure	NA	NA	NA	NA	NA	NA	NA	NA	NA	NA	NA	NA
Low birth weight and short gestation	NA	NA	NA	NA	NA	NA	NA	NA	NA	NA	NA	NA
Iron deficiency	NA	NA	NA	NA	NA	NA	NA	NA	NA	NA	NA	NA
Vitamin A deficiency	NA	NA	NA	NA	NA	NA	NA	NA	NA	NA	NA	NA

Zinc deficiency	NA	NA	NA	NA	NA	NA	NA	NA	NA	NA	NA	NA
Tobacco	0.88 (0.65 to 1.17)	0.88 (0.63 to 1.17)	-0.62 (-24.81 to 26.67)	0.75 (0.54 to 1.04)	0.37 (0.26 to 0.52)	-49.87 (-67.35 to -32.43)	0.62 (0.46 to 0.79)	0.63 (0.46 to 0.83)	1.84 (-11.48 to 18.14)	30.83 (22.59 to 40.52)	15.46 (10.66 to 21.97)	-49.87 (-57.61 to -40.91)
Smoking	0.88 (0.65 to 1.17)	0.88 (0.63 to 1.17)	-0.62 (-24.81 to 26.67)	0.75 (0.54 to 1.04)	0.37 (0.26 to 0.52)	-49.87 (-67.35 to -32.43)	0.62 (0.46 to 0.79)	0.63 (0.46 to 0.83)	1.84 (-11.48 to 18.14)	30.83 (22.59 to 40.52)	15.46 (10.66 to 21.97)	-49.87 (-57.61 to -40.91)
Chewing tobacco	NA	NA	NA	NA	NA	NA	NA	NA	NA	NA	NA	NA
Secondhand smoke	NA	NA	NA	NA	NA	NA	NA	NA	NA	NA	NA	NA
Alcohol use	11.59 (8.47 to 15.18)	16.44 (11.17 to 21.8)	41.88 (2.98 to 88.65)	9.8 (7.02 to 14.04)	7.02 (4.58 to 9.51)	-28.37 (-58.53 to -1.34)	10.42 (7.59 to 13.54)	14.65 (10.31 to 19.11)	40.59 (5.17 to 83.69)	517.16 (369.99 to 707.74)	356.79 (251.04 to 466.1)	-31.01 (-53.6 to -9.72)
Drug use	0.12 (0.05 to 0.26)	0.38 (0.18 to 0.68)	210.51 (39.05 to 326.69)	0.1 (0.04 to 0.25)	0.16 (0.07 to 0.3)	55.53 (-44.78 to 122.12)	0.12 (0.05 to 0.25)	0.34 (0.16 to 0.64)	197.77 (25.28 to 323.81)	5.75 (2.41 to 13.25)	8.37 (3.88 to 15.4)	45.61 (-45.17 to 108)
Dietary risks	NA	NA	NA	NA	NA	NA	NA	NA	NA	NA	NA	NA
Diet low in fruits	NA	NA	NA	NA	NA	NA	NA	NA	NA	NA	NA	NA
Diet low in vegetables	NA	NA	NA	NA	NA	NA	NA	NA	NA	NA	NA	NA
Diet low in legumes	NA	NA	NA	NA	NA	NA	NA	NA	NA	NA	NA	NA
Diet low in whole grains	NA	NA	NA	NA	NA	NA	NA	NA	NA	NA	NA	NA
Diet low in nuts and seeds	NA	NA	NA	NA	NA	NA	NA	NA	NA	NA	NA	NA
Diet low in milk	NA	NA	NA	NA	NA	NA	NA	NA	NA	NA	NA	NA
Diet high in red meat	NA	NA	NA	NA	NA	NA	NA	NA	NA	NA	NA	NA

Diet high in processed meat	NA	NA	NA	NA	NA	NA	NA	NA	NA	NA	NA	NA
Diet high in sugar-sweetened beverages	NA	NA	NA	NA	NA	NA	NA	NA	NA	NA	NA	NA
Diet low in fiber	NA	NA	NA	NA	NA	NA	NA	NA	NA	NA	NA	NA
Diet low in calcium	NA	NA	NA	NA	NA	NA	NA	NA	NA	NA	NA	NA
Diet low in seafood omega-3 fatty acids	NA	NA	NA	NA	NA	NA	NA	NA	NA	NA	NA	NA
Diet low in polyunsaturated fatty acids	NA	NA	NA	NA	NA	NA	NA	NA	NA	NA	NA	NA
Diet high in trans fatty acids	NA	NA	NA	NA	NA	NA	NA	NA	NA	NA	NA	NA
Diet high in sodium	NA	NA	NA	NA	NA	NA	NA	NA	NA	NA	NA	NA
Intimate partner violence	0.18 (0.12 to 0.24)	0.23 (0.16 to 0.33)	30.25 (2.16 to 77.77)	0.15 (0.11 to 0.2)	0.1 (0.07 to 0.14)	-34.2 (-47.81 to -17.29)	0.23 (0.15 to 0.32)	0.33 (0.22 to 0.46)	43.9 (22.65 to 70.6)	11.25 (7.61 to 15.82)	7.95 (5.35 to 11.2)	-29.33 (-37.95 to -18.59)
Childhood sexual abuse and bullying	NA	NA	NA	NA	NA	NA	NA	NA	NA	NA	NA	NA
Childhood sexual abuse	NA	NA	NA	NA	NA	NA	NA	NA	NA	NA	NA	NA
Unsafe sex	NA	NA	NA	NA	NA	NA	NA	NA	NA	NA	NA	NA
Low physical activity	NA	NA	NA	NA	NA	NA	NA	NA	NA	NA	NA	NA
**Metabolic risks**	8.27 (6.91 to 9.66)	10.44 (8.61 to 11.89)	26.29 (2.81 to 40.13)	6.99 (5.73 to 9.21)	4.45 (3.48 to 5.28)	-36.26 (-58.25 to -24.15)	5.76 (4.89 to 6.37)	7.79 (6.53 to 8.77)	35.4 (27.25 to 45.07)	285.8 (233.45 to 337.81)	190.55 (147.48 to 240.89)	-33.33 (-42.05 to -27.07)
High fasting plasma glucose	NA	NA	NA	NA	NA	NA	NA	NA	NA	NA	NA	NA

High LDL cholesterol	NA	NA	NA	NA	NA	NA	NA	NA	NA	NA	NA	NA
High systolic blood pressure	NA	NA	NA	NA	NA	NA	NA	NA	NA	NA	NA	NA
High body-mass index	NA	NA	NA	NA	NA	NA	NA	NA	NA	NA	NA	NA
Low bone mineral density	8.27 (6.91 to 9.66)	10.44 (8.61 to 11.89)	26.29 (2.81 to 40.13)	6.99 (5.73 to 9.21)	4.45 (3.48 to 5.28)	-36.26 (-58.25 to -24.15)	5.76 (4.89 to 6.37)	7.79 (6.53 to 8.77)	35.4 (27.25 to 45.07)	285.8 (233.45 to 337.81)	190.55 (147.48 to 240.89)	-33.33 (-42.05 to -27.07)
Kidney dysfuntion	NA	NA	NA	NA	NA	NA	NA	NA	NA	NA	NA	NA

Appendix table 12. Comparison of estimates between GBD and national report for the top 10 Level-3 all-ages death rates in South Korea, 2019.

GBD 2019 top 10 Level-3 leading causes of deaths		GBD estimation (per 100,000)	National estimation (per 100,000)
1	Stroke	68.4	42.0
2	Ischemic heart disease	52.96	26.7
3	Lung cancer	43.6	36.2
4	Alzheimer's disease	34.6	13.1
5	Lower respiratory infection	29.28	45.1
6	Self-harm	27.3	26.9
7	Liver cancer	27.11	20.6
8	Diabetes	24.1	15.8
9	Stomach cancer	22.9	14.9
10	Colorectal cancer	22.5	17.5

Reference: " Statistics Korea (South Korea). Causes of Death Statistics in 2019"

Appendix table 13. Comparison of lists of International Classification of Diseases (ICD) codes mapped to GBD study and Causes of Death Statistics from Statistics Korea along with the top 10 Level-3 all-ages death rates in South Korea in 2019.

GBD 2019 top 10 Level-3 leading causes of deaths		GBD 2019 study (ICD10/ICD9)	Statistics Korea (ICD10)
1	Stroke	G45-G46.8, I60-I63.9, I65-I66.9, I67.0-I67.3, I67.5-I67.6, I68.1-I68.2, I69.0-I69.3 / 430-435.9, 437.0-437.2, 437.5-437.8	I60-I69
2	Ischemic heart disease	I20-I25.9 / 410-414.9	I20-I25
3	Lung cancer	C33-C34.9, D02.1-D02.3, D14.2-D14.3, D38.1 / 162-162.9, 212.2-212.3, 231.1-231.2, 235.7	C33-C34
4	Alzheimer's disease	F00-F02.0, F02.8-F03.9, G30-G31.1, G31.8-G31.9 / 290-290.9, 294.1-294.9, 331-331.2	G30
5	Lower respiratory infection	A48.1, A70, B97.4-B97.6, J09-J15.8, J16-J16.9, J20-J21.9, J91.0, P23.0-P23.4, U04-U04.9 / 079.6, 466-469, 470.0, 480-482.8, 483.0-483.9, 484.1-484.2, 484.6-484.7, 487-489	J12-J18
6	Self-harm	X60-X64.9, X66-X83.9, Y87.0 / E950-E959	X60-X84
7	Liver cancer	C22-C22.8, D13.4 / 155-155.1, 155.3-155.9, 211.5	C22
8	Diabetes	E10-E10.1, E10.3-E11.1, E11.3-E11.9, P70.2 / 775.1	E10-E14
9	Stomach cancer	C16-C16.9, D00.2, D13.1, D37.1 / 151-151.9, 211.1, 230.2	C16
10	Colorectal cancer	C18-C21.9, D01.0-D01.3, D12-D12.9, D37.3-D37.5 / 153-154.9, 209.1, 209.5, 211.3-211.4, 230.3-230.6, 569.0	C18-C21

Appendix figures

Appendix figure 1. Trends in deaths [(A) total number, (B) all-age rates, and (C) age-standardised rates] in South Korea from 1990 to 2019, by GBD Level 1 cause group: communicable, maternal, neonatal, and nutritional (CMNN) diseases; non-communicable diseases (NCDs); and injuries.

The difference in trends between the number of total deaths and the all-age death rate is caused by population growth, and the difference between all-age and age-standardised rates is caused by changes in the percentage distribution of population by age. Shaded areas show 95% uncertainty intervals.

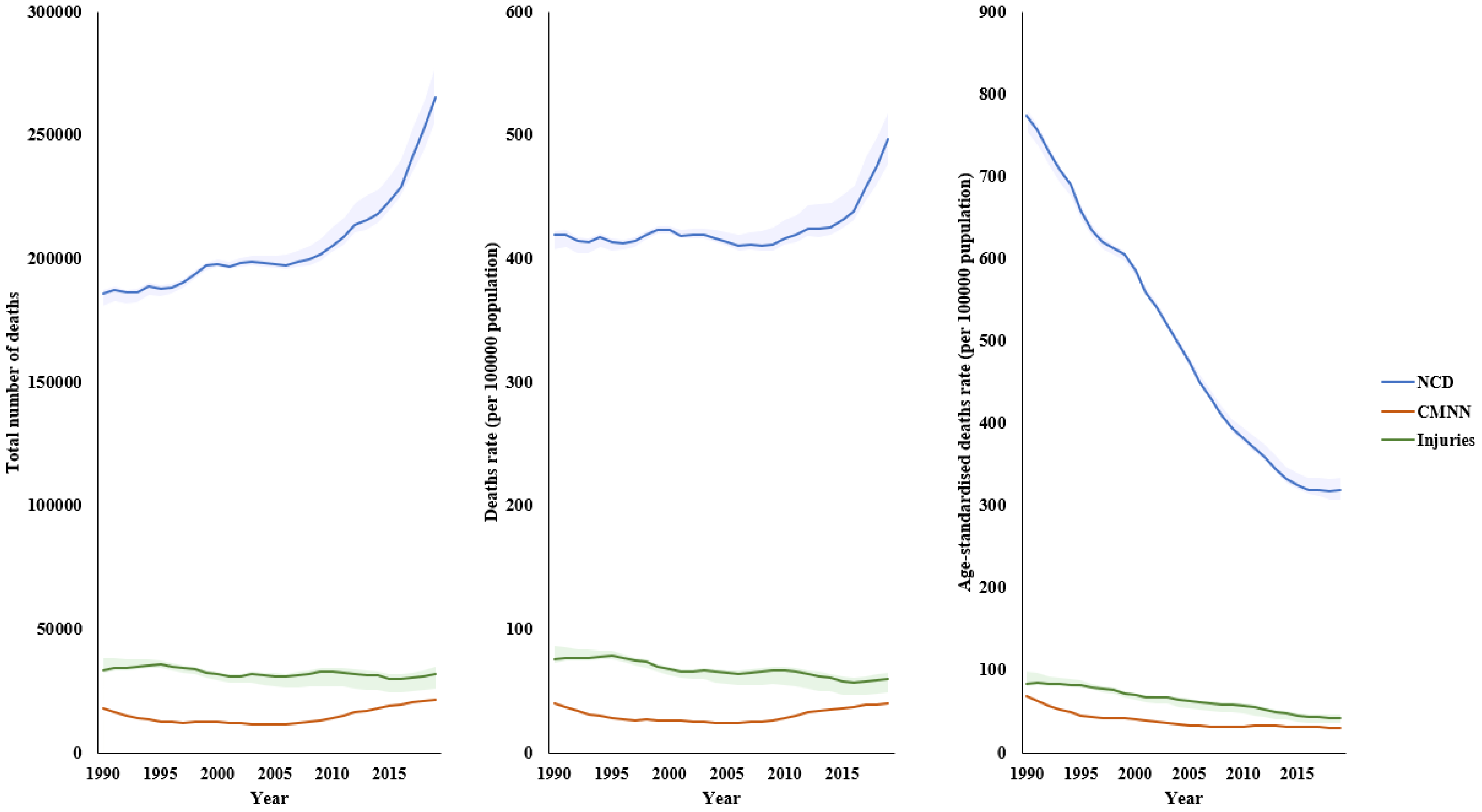

Appendix figure 2. Leading 30 Level-3 prevalence in 1990 and 2019 in South Korea, with rates of all-age prevalence and percentage of all-age prevalence; and percentage change in number of prevalence, all-age prevalence, and age-standardised prevalence. Causes of prevalence cases are connected by arrows between time periods; solid lines are increases in rank and dashed lines are decreases. Non-communicable diseases are labelled in blue, communicable, maternal, neonatal, and nutritional diseases are labelled in red, and injuries are labelled in green.

STIs= Sexually transmitted infections excluding HIV, Cirrhosis=Cirrhosis and other chronic liver diseases, Age-related hearing loss=Age-related and other hearing loss, Intestinal nematode=Intestinal nematode infections, Hemoglobinopathies=Hemoglobinopathies and hemolytic anemias, Mechanical forces=Exposure to mechanical forces, Other skin diseases=Other skin and subcutaneous diseases, Endo/metab/blood/immune=Endocrine, metabolic, blood, and immune disorders, Upper digestive diseases=Upper digestive system diseases, Other unintentional=Other unintentional injuries, Other musculoskeletal=Other musculoskeletal disorders, Gallbladder & biliary=Gallbladder and biliary diseases, Upper respiratory infect=Upper respiratory infections, Diabetes=Diabetes mellitus.

Leading causes 1990	Prevalence 1990 (per 100·000)	Percentage of prevalence 1990 (%)	Leading causes 2019	Prevalence 2019 (per 100·000)	Percentage of prevalence 2019 (%)	Mean % change in number of prevalences 1990-2019	Mean % change in rate of all-age prevalences 1990-2019	Mean % change in rate of age-standardised prevalences 1990-2019
1 Oral disorders	41772·99 (37560·36 to 46435·25)	44 (39·73 to 48·78)	1 Oral disorders	45319·26 (40953·9 to 50051·41)	47·39 (42·89 to 52·24)	30·66 (22·8 to 39·46)	8·49 (1·96 to 15·79)	-2·99 (-6·51 to 0·56)
2 Tuberculosis	39185·51 (36787·55 to 41493·59)	41·27 (38·75 to 43·67)	2 Headahce disorders	39932·65 (36748·63 to 43129·96)	41·76 (38·53 to 45·01)	31·89 (23·54 to 40·34)	9·51 (2·58 to 16·53)	3·22 (-1·04 to 8·11)
3 Headahce disorders	36464·33 (32966·39 to 39979·25)	38·41 (34·78 to 42·06)	3 Other neoplasms	22050·92 (18842·18 to 25521·53)	23·06 (19·69 to 26·74)	49·42 (37·45 to 63·02)	24·07 (14·13 to 35·36)	-0·42 (-1·15 to 0·29)
4 STIs	19426·35 (18746·74 to 20100·52)	20·46 (19·73 to 21·17)	4 Age-related hearing loss	19755·63 (18721·18 to 20815·77)	20·66 (19·58 to 21·78)	148·6 (140·18 to 159·15)	106·42 (99·43 to 115·18)	0·4 (-1·62 to 3·15)
5 Dietary iron deficiency	18623·33 (17418·37 to 19826·2)	19·62 (18·39 to 20·92)	5 Tuberculosis	18433·09 (15937·65 to 21532·17)	19·28 (16·66 to 22·49)	-43·35 (-49·78 to -35·79)	-52·96 (-58·3 to -46·68)	-60·57 (-64·78 to -55·56)
6 Other neoplasms	17772·91 (15116·29 to 20774·84)	18·72 (15·97 to 21·79)	6 STIs	17853·18 (15569·79 to 20336·02)	18·67 (16·28 to 21·31)	10·68 (-1·85 to 24·29)	-8·1 (-18·5 to 3·2)	-33·01 (-40·67 to -25·5)
7 Gynecological diseases	16317·88 (14406·17 to 18290·78)	17·19 (15·22 to 19·3)	7 Osteoarthritis	15792·87 (14316·79 to 17565·59)	16·52 (14·96 to 18·38)	182·58 (175·77 to 190·31)	134·63 (128·98 to 141·05)	-1·09 (-2·98 to 0·91)
8 Cirrhosis	12979·59 (12048·44 to 14011·22)	13·67 (12·69 to 14·72)	8 Cirrhosis	15075·37 (13912·59 to 16438·18)	15·77 (14·55 to 17·19)	39·88 (32·34 to 47·79)	16·15 (9·88 to 22·72)	-17·98 (-21·2 to -14·67)
9 Age-related hearing loss	9570·58 (9025·18 to 10108·45)	10·08 (9·5 to 10·65)	9 Gynecological diseases	14928·16 (13280·28 to 16600·53)	15·61 (13·93 to 17·35)	10·18 (4·98 to 16·78)	-5·82 (-8·08 to -3·36)	-8·52 (-12·83 to -3·04)
10 Intestinal nematode	9491·62 (6795·15 to 12980·32)	10 (7·18 to 13·67)	10 Other skin diseases	13725·36 (13361·31 to 14129·22)	14·35 (13·97 to 14·8)	97·25 (93·05 to 101·81)	63·78 (60·29 to 67·57)	8·13 (6·37 to 9·84)
11 Low back pain	8767·02 (7595·71 to 10072·53)	9·23 (8 to 10·59)	11 Falls	13070·83 (11436·11 to 15197·02)	13·67 (11·99 to 15·87)	82·46 (75·33 to 90·39)	51·5 (45·58 to 58·09)	1·48 (-1·05 to 4·59)
12 Hemoglobinopathies	8732·86 (7883·63 to 9632·86)	9·2 (8·3 to 10·14)	12 Mechnical forces	12430·16 (10692·73 to 14245·9)	13 (11·19 to 14·9)	76·17 (71·23 to 81·47)	46·28 (42·18 to 50·68)	4·19 (1·09 to 7·48)
13 Falls	8627·62 (7380·39 to 10206·32)	9·09 (7·78 to 10·74)	13 Low back pain	11781·8 (10244·93 to 13566·11)	12·32 (10·73 to 14·18)	61·85 (50·32 to 74·05)	34·39 (24·82 to 44·52)	-6·49 (-10·09 to -2·6)
14 Mechnical forces	8497·58 (7326·8 to 9793·47)	8·95 (7·73 to 10·31)	14 Chronic kidney disease	10359·01 (9795·09 to 10939·29)	10·83 (10·23 to 11·44)	128·7 (115·75 to 142·74)	89·89 (79·14 to 101·56)	-2·12 (-6·63 to 2·78)
15 Other skin diseases	8380·17 (8151·05 to 8641·08)	8·83 (8·58 to 9·11)	15 Endo/metab/blood/immune	9963·44 (8422·86 to 11856·38)	10·42 (8·81 to 12·38)	57·17 (42·47 to 71·75)	30·5 (18·29 to 42·6)	-4·54 (-9·09 to 0·75)
16 Endo/metab/blood/immune	7634·78 (6633·47 to 8850·65)	8·04 (7 to 9·32)	16 Other musculoskeletal	9918·77 (8280·5 to 11845·03)	10·37 (8·65 to 12·37)	130·99 (107·12 to 159·6)	91·8 (71·97 to 115·55)	39·25 (28·98 to 51·61)
17 Osteoarthritis	6730·82 (6059·24 to 7524·63)	7·09 (6·38 to 7·92)	17 Upper digest disease	9206·18 (8162·19 to 10508·7)	9·63 (8·52 to 10·99)	70·96 (60·31 to 81·85)	41·95 (33·11 to 50·99)	-1·57 (-2·88 to -0·47)
18 Upper digest disease	6485·32 (5634·6 to 7352·14)	6·83 (5·94 to 7·76)	18 Dietary iron deficiency	7986·26 (6817·12 to 9266·42)	8·35 (7·12 to 9·68)	-48·35 (-56·72 to -39·45)	-57·12 (-64·06 to -49·72)	-57·54 (-64·42 to -49·78)
19 Other unitentional	5870·19 (4866·77 to 7182·68)	6·18 (5·15 to 7·55)	19 Diabetes	7896·21 (7273·8 to 8608·54)	8·26 (7·6 to 9·01)	229·33 (207·72 to 250·75)	173·45 (155·51 to 191·23)	34·87 (26·07 to 43·26)
20 Chronic kidney disease	5455·17 (5025·97 to 5904·51)	5·75 (5·29 to 6·22)	20 Hemoglobinopathies	7707·52 (6866·13 to 8560·76)	8·06 (7·19 to 8·93)	6·29 (1·75 to 11·13)	-11·74 (-15·52 to -7·72)	-14·56 (-18·4 to -10·66)
21 Other musculoskeletal	5171·43 (4195·72 to 6229·22)	5·45 (4·41 to 6·56)	21 Fungal skin diseases	6931·2 (6174·98 to 7817·72)	7·25 (6·47 to 8·18)	74·4 (61·75 to 87·84)	44·81 (34·3 to 55·97)	-1·94 (-2·93 to -1·22)
22 Fungal skin diseases	4786·5 (4248·32 to 5442·34)	5·04 (4·47 to 5·72)	22 Other unintentional	6246·71 (5128·41 to 7706·25)	6·53 (5·35 to 8·05)	28·16 (22·33 to 33·9)	6·41 (1·57 to 11·18)	-20·98 (-24·77 to -16·65)
23 Acne vulgaris	4578·67 (4033·63 to 5167·44)	4·82 (4·24 to 5·43)	23 Gallbladder & biliary	4827·86 (4065·33 to 5844·07)	5·05 (4·25 to 6·13)	43·64 (28·97 to 60·5)	19·27 (7·09 to 33·27)	-30·75 (-36·84 to -24)
24 Dermatitis	4376·66 (4137·92 to 4622·76)	4·61 (4·36 to 4·88)	24 Asthma	3587·84 (3017·93 to 4353·14)	3·75 (3·16 to 4·55)	14·46 (1·94 to 30·28)	-4·96 (-15·36 to 8·18)	-19·65 (-32·15 to -7·23)
25 Gallbladder & biliary	4047·94 (3430·61 to 4758·74)	4·26 (3·61 to 5·01)	25 Dermatitis	3546·01 (3371·28 to 3717·43)	3·71 (3·53 to 3·89)	-2·42 (-6·44 to 1·72)	-18·98 (-22·32 to -15·54)	0·52 (-3·06 to 4·44)
26 Upper respiratory infect	3942 (3494·21 to 4496·76)	4·15 (3·68 to 4·74)	26 Acne vulgaris	3496·9 (3175·67 to 3822·57)	3·66 (3·32 to 4)	-8·02 (-13·18 to -1·83)	-23·63 (-27·91 to -18·49)	21·56 (15·59 to 28·4)
27 Viral skin diseases	3812·24 (3655·12 to 3964·63)	4·02 (3·85 to 4·18)	27 Upper respiratory infect	3496·02 (3143·08 to 3921·93)	3·66 (3·29 to 4·1)	6·81 (-0·6 to 15·51)	-0·17 (-4·15 to 4·44)	-11·31 (-17·47 to -4·09)
28 Asthma	3775·09 (3194·31 to 4517·14)	3·98 (3·37 to 4·75)	28 Anxiety disoders	3487·42 (2770·64 to 4386·49)	3·65 (2·9 to 4·58)	24·08 (9·76 to 41·68)	3·03 (-8·87 to 17·64)	-0·52 (-6·46 to 6·41)
29 Anxiety disoders	3384·92 (2628·83 to 4260·38)	3·57 (2·77 to 4·5)	29 Blindness and vision loss	3285·64 (2922·97 to 3693·38)	3·44 (3·05 to 3·87)	83·74 (70·68 to 98·01)	52·57 (41·72 to 64·41)	-8·23 (-11·42 to -4·75)
30 Alcohol use disorders	3242·22 (2653·58 to 3882·3)	3·42 (2·79 to 4·09)	30 Intestinal nematode	3214·63 (2272·36 to 4459·61)	3·36 (2·37 to 4·65)	-56·19 (-72·19 to -31·32)	-59·21 (-74·21 to -35·99)	-66·13 (-78·58 to -46·85)
31 Diabetes	2887·61 (2674·14 to 3109·85)		32 Viral skin diseases	3125·56 (3012·96 to 3240·26)				
34 Blindness and vision loss	2153·56 (1926·39 to 2388·32)							

Appendix figure 3. Leading 30 Level-3 incidences in 1990 and 2019 in South Korea, with rates of all-age new cases and percentage of all-age new cases; and percentage change in number of new cases, all-age new cases, and age-standardised new cases for both sexes combined for all ages. Causes of new cases are connected by arrows between time periods; solid lines are increases in rank and dashed lines are decreases. Non-communicable diseases are labelled in blue, communicable, maternal, neonatal, and nutritional diseases are labelled in red, and injuries are labelled in green.

Upper respiratory infect=Upper respiratory infections, Bacterial skin=Bacterial skin diseases, Other skin diseases=Other skin and subcutaneous diseases, Urinary diseases=Urinary diseases and male infertility, STIs=Sexually transmitted infections excluding HIV, Mechanical forces=Exposure to mechanical forces, Upper digestive diseases=Upper digestive system diseases, Other unintentional=Other unintentional injuries, Lower respiratory infect=Lower respiratory infections, Varicella=Varicella and herpes zoster, Gallbladder & biliary=Gallbladder and biliary diseases

Leading causes 1990	New cases 1990 (per 100·000)	Percentage of new cases 1990 (%)	Leading causes 2019	New cases 2019 (per 100·000)	Percentage of new cases 2019 (%)	Mean % change in number of new cases 1990-2019	Mean % change in rate of all-age new cases 1990-2019	Mean % change in rate of age-standardised new cases 1990-2019
1 Upper respiratory infect	286079·51(253212·15 to 326378·73)	57·21 (54·14 to 60·45)	1 Upper respiratory infect	253824·85 (228203·82 to 284971·1)	53·21 (50·52 to 56·27)	6·86 (-0·44 to 15·57)	**-11·27 (-17·33 to -4·04)**	-0·17 (-4·23 to 4·45)
2 Oral disorders	57027·11 (49968·16 to 64087·67)	11·43 (9·98 to 12·9)	2 Oral disorders	48935·08 (43861·45 to 54457·16)	10·27 (9·15 to 11·45)	3·35 (-4·83 to 13·54)	**-14·19 (-20·98 to -5·72)**	1·58 (-3·64 to 6·56)
3 Diarrheal diseases	19308·27 (16982·96 to 21839·38)	3·87 (3·33 to 4·49)	3 Diarrheal diseases	28775·47 (25254·42 to 32618·9)	6·04 (5·3 to 6·94)	**79·49 (62·96 to 96·47)**	**49·03 (35·31 to 63·14)**	-3·36 (-11·93 to 5·44)
4 Bacterial skin	18100·81 (17441·74 to 18794·2)	3·63 (3·34 to 3·94)	4 Fungal skin diseases	23114·1 (20533·98 to 25880·11)	4·85 (4·28 to 5·45)	**76·18 (63·83 to 89·91)**	**46·29 (36·03 to 57·69)**	**-1·4 (-2·05 to -0·87)**
5 Fungal skin diseases	15800·24 (14195·6 to 17822·35)	3·17 (2·77 to 3·65)	5 Bacterial skin	15037·35 (14575 to 15543·74)	3·16 (2·94 to 3·38)	0·05 (-2·72 to 2·9)	**-16·92 (-19·23 to -14·56)**	0·49 (-2·14 to 3)
6 Headache disorders	11817·61 (10409·71 to 13274·35)	2·37 (2·03 to 2·7)	6 Other skin diseases	14224·23 (13846·65 to 14653·36)	2·99 (2·78 to 3·17)	**96·61 (92·3 to 101·19)**	**63·25 (59·67 to 67·06)**	**8·16 (6·44 to 9·88)**
7 Other neoplasms	9234·08 (7731·88 to 11129·74)	1·85 (1·53 to 2·28)	7 Headache disorders	11739·31 (10409·03 to 13145·5)	2·47 (2·15 to 2·8)	**19·64 (11·62 to 29·84)**	-0·66 (-7·32 to 7·81)	0·55 (-3·99 to 5·22)
8 Other skin diseases	8713·03 (8483·75 to 8975·01)	1·75 (1·6 to 1·89)	8 Other neoplasms	11096·5 (9240·37 to 13429·36)	2·33 (1·94 to 2·84)	**44·73 (31·49 to 59·01)**	**20·17 (9·18 to 32·03)**	**-0·3 (-0·51 to -0·11)**
9 Urinary diseases	6836·52 (6100·38 to 7612·13)	1·37 (1·2 to 1·55)	9 Urinary diseases	7788·73 (6896·47 to 8654·16)	1·64 (1·44 to 1·85)	**37·21 (26·4 to 49·91)**	**13·93 (4·95 to 24·48)**	1·2 (-5·14 to 7·94)
10 STIs	6786·57 (5832·66 to 7947·29)	1·36 (1·13 to 1·61)	10 STIs	6517·88 (5493·68 to 7613·53)	1·37 (1·14 to 1·6)	**15·67 (7·41 to 24·59)**	-3·96 (-10·82 to 3·45)	**-5·21 (-8·74 to -1·56)**
11 Gynecological diseases	6239·14 (5362·54 to 7302·44)	1·25 (1·05 to 1·47)	11 Gynecological diseases	5200·48 (4477·86 to 5969·58)	1·09 (0·92 to 1·28)	0·39 (-5·19 to 7·24)	**-16·65 (-21·28 to -10·96)**	**-6·89 (-8·88 to -4·77)**
12 Meachnical forces	5133·05 (4209 to 6192·69)	1·03 (0·83 to 1·24)	12 Falls	4632·47 (4110·83 to 5215·93)	0·97 (0·85 to 1·11)	**10·46 (3·25 to 18·64)**	**-8·29 (-14·27 to -1·49)**	0·63 (-6·37 to 7·58)
13 Falls	5051·02 (4481·25 to 5702·85)	1·01 (0·87 to 1·17)	13 Mechanical forces	4395·48 (3586·61 to 5245·16)	0·92 (0·75 to 1·09)	3·13 (-7·48 to 14·38)	**-14·37 (-23·18 to -5·02)**	0·66 (-8·83 to 9·74)
14 Acute hepatits	3441·13 (3084·91 to 3831·58)	0·69 (0·6 to 0·79)	14 Upper digest diseases	3948·44 (3474·88 to 4556·49)	0·83 (0·72 to 0·96)	**0·65 (0·54 to 0·75)**	**0·37 (0·28 to 0·45)**	**-0·02 (-0·03 to -0·02)**
15 Dermatitis	3394·23 (3095·72 to 3694·26)	0·68 (0·6 to 0·76)	15 Low back pain	3458·65 (3016·94 to 3949)	0·73 (0·63 to 0·85)	**54·63 (42·19 to 68·09)**	**28·39 (18·06 to 39·57)**	**-3·03 (-5·89 to -0·17)**
16 Upper digest diseases	2886·62 (2506·49 to 3314·02)	0·58 (0·49 to 0·67)	16 Dermatitis	3406·49 (3096·08 to 3725·26)	0·72 (0·64 to 0·79)	**20·87 (15·83 to 26·34)**	0·36 (-3·82 to 4·9)	-0·22 (-0·94 to 0·51)
17 Otitis media	2742·19 (2048·74 to 3551·38)	0·55 (0·41 to 0·72)	17 Depressive disorders	2657·51 (2363·78 to 2961·52)	0·56 (0·49 to 0·63)	**52·66 (38·21 to 69)**	**26·76 (14·76 to 40·33)**	**9·67 (2·53 to 17·09)**
18 Low back pain	2693·78 (2353·37 to 3083·14)	0·54 (0·46 to 0·64)	18 Otitis media	2204·93 (1656·89 to 2834·85)	0·46 (0·35 to 0·6)	-3·16 (-12·11 to 7·24)	**-19·59 (-27·02 to -10·96)**	**23·82 (11·87 to 38·13)**
19 Other unintentional	2529·47 (2105·19 to 3006·11)	0·51 (0·41 to 0·61)	19 Acute hapatitis	2179·57(2000·95 to 2425·25)	0·46 (0·41 to 0·51)	**-23·72 (-32·76 to -13·63)**	**-36·66 (-44·17 to -28·29)**	**-28·29 (-35·58 to -21·29)**
20 Lower respiratory infect	2464·54 (2191·24 to 2738·95)	0·49 (0·43 to 0·56)	20 Lower respiratory infect	1816·72 (1662·57 to 1979·29)	0·38 (0·34 to 0·42)	**-11·22 (-18·21 to -4·45)**	**-26·29 (-32·09 to -20·66)**	**-27·73 (-32·16 to -23·24)**
21 Acne vulgaris	2288·74 (1962·91 to 2676·28)	0·46 (0·38 to 0·55)	21 Acne vulgaris	1654·18 (1458·61 to 1872·58)	0·35 (0·3 to 0·4)	**-12·96 (-18·47 to -6·26)**	**-27·73 (-32·3 to -22·17)**	**22·34 (14·98 to 31·92)**
22 Depressive disorders	2096·5 (1825·89 to 2397·34)	0·42 (0·36 to 0·49)	22 Other unintentional	1482·87 (1182·35 to 1830·14)	0·31 (0·25 to 0·39)	**-29·4 (-37·27 to -21·53)**	**-41·38 (-47·91 to -34·84)**	**-23·39 (-32·14 to -15·18)**
23 Viral skin diseases	1624·52 (1557·44 to 1693·53)	0·33 (0·3 to 0·35)	23 Gallbladder & biliary	1475·95 (1249·12 to 1774·82)	0·31 (0·26 to 0·38)	**98·24 (78·33 to 120·68)**	**64·6 (48·07 to 83·24)**	-0·45 (-9·01 to 8·23)
24 Urticaria	1307·89 (1146·62 to 1498·42)	0·26 (0·23 to 0·3)	24 Viral skin diseases	1209·58 (1168·81 to 1253·28)	0·25 (0·24 to 0·27)	**-10·33 (-11·94 to -8·64)**	**-25·54 (-26·88 to -24·14)**	0·01 (-0·04 to 0·06)
25 Alcohol use disorders	1301·02 (994·88 to 1578·93)	0·26 (0·2 to 0·32)	25 Osteoarthritis	1191·99 (1058·62 to 1354·61)	0·25 (0·22 to 0·29)	**132·31 (123·87 to 141·42)**	**92·9 (85·88 to 100·45)**	0·31 (-2·3 to 2·84)
26 Road injuries	1246·84 (1089·34 to 1424·27)	0·25 (0·21 to 0·29)	26 Urticaria	1149·5 (1025·02 to 1278·86)	0·24 (0·21 to 0·27)	5·85 (-0·41 to 12·42)	**-12·11 (-17·31 to -6·66)**	**-1·02 (-1·17 to -0·86)**
27 Varicella	1086·07 (977·49 to 1183·41)	0·22 (0·19 to 0·24)	27 Alcohol use disorders	1128·55 (885·8 to 1383·51)	0·24 (0·18 to 0·3)	4·47 (-14·06 to 34·71)	-13·26 (-28·64 to 11·86)	-18·57 (-32·72 to 0·02)
28 Gallbladder & biliary	896·69 (763·52 to 1045·13)	0·18 (0·15 to 0·21)	28 Varicella	1029·11 (897·91 to 1174·92)	0·22 (0·19 to 0·25)	**14·12 (2·39 to 32·14)**	-5·24 (-14·98 to 9·72)	**5·12 (0·9 to 16·61)**
29 Animal contact	887·49 (696·7 to 1171·94)	0·18 (0·14 to 0·23)	29 Animal contact	725·7 (575·53 to 925·29)	0·15 (0·12 to 0·2)	-1·52 (-11·62 to 11·35)	**-18·23 (-26·61 to -7·55)**	**-9·08 (-13·26 to -4·79)**
30 Foreign body	837·19 (674·25 to 1039·29)	0·17 (0·13 to 0·21)	30 Foreign body	711·92 (580·96 to 876·45)	0·15 (0·12 to 0·19)	2·41 (-6·1 to 12·21)	**-14·96 (-22·03 to -6·83)**	1·81 (-0·4 to 4·02)
32 Osteoarthritis	837·19 (674·25 to 1039·29)	0·12 (0·11 to 0·14)	32 Road injuries	648·59 (559 to 743·66)	0·14 (0·12 to 0·16)			

Appendix figure 4. Trends in age-standardised death and DALY rates of (A) communicable, maternal, neonatal, and nutritional (CMNN) diseases, (B) non-communicable diseases (NCDs), and (C) injuries attributable to GBD level 1 risk factors in South Korea and Global from 1990 to 2019, by GBD Level 1 risk factor group: environmental/occupational risks; behavioral risks; and metabolic risks.

(A) Communicable, maternal, neonatal, and nutritional diseases

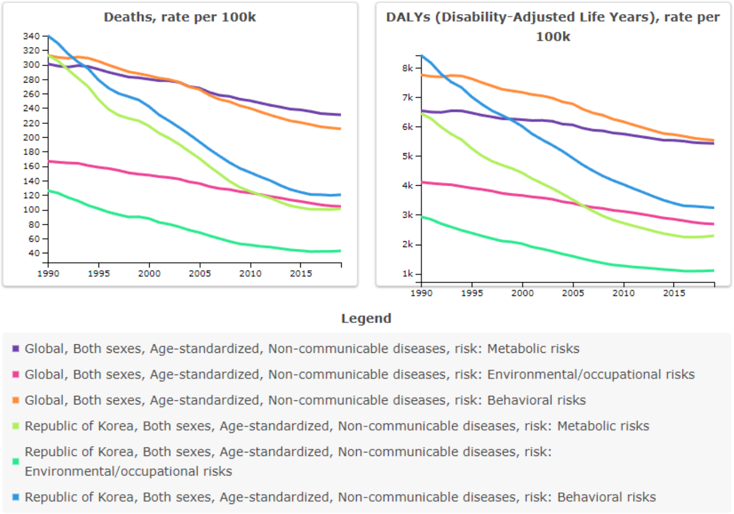

(B) Non-communicable diseases

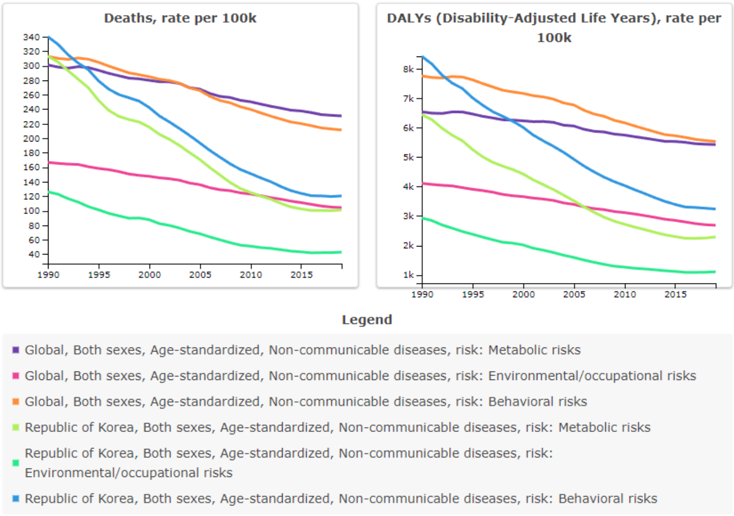

(C) Injuries

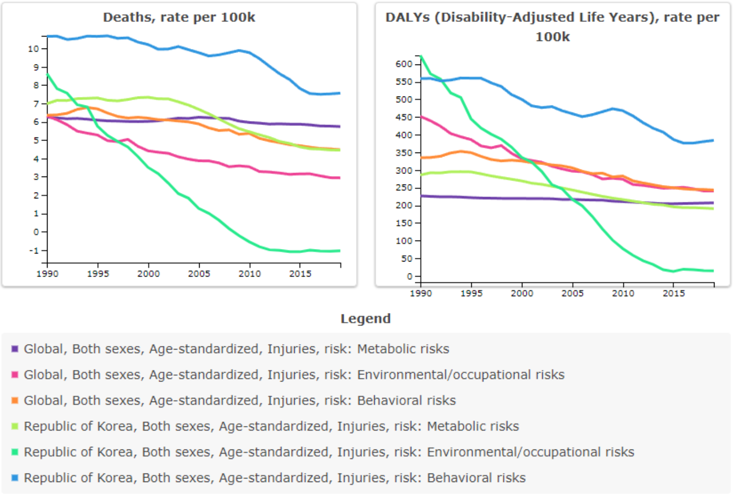

Appendix method 1. Summary of methodology for "Causes of Death Statistics in 2019, South Korea"[1]

Data source and unspecified death

- The results are aggregated based on death certificates submitted to the local administrative welfare centers in towns, townships, and neighborhoods, as well as city and district offices (overseas citizens submit to diplomatic missions) according to the Statistics Act and the Act on Family Relations Registration. The cause of death statistics is compiled based on the underlying cause (the fundamental factor leading to death) in accordance with the World Health Organization (WHO) guidelines for cause classification.
- Deaths caused by imprecise causes or external factors (e.g., accidental deaths) are supplemented with cause of death information obtained from other administrative records, including those from the National Cancer Center, National Health Insurance Corporation, National Forensic Service, Korean National Police Agency, and the Ministry of National Defense.
- The death certificate must be written by a physician, with only a few exceptions. However, due to a lack of education on how to properly designate the cause of death, some doctors arbitrarily write the cause of death rather than following the death certificate writing guidelines.[2] Such practices have raised an issue with the reliability of the cause of death statistics in South Korea.[2] Moreover, an autopsy is not necessarily required to write death certificates, and many times doctors do not have access to electronic medical records of a deceased person, making it hard to report a reliable cause of death. Altogether, such limitations in the collecting system increased the portion of garbage code (any recorded cause of death on a death certificate that cannot officially result in death)[3] and unknown cause of death (14%).[2] Large portion with unknown cause of death directly influences the national death statistics that relies on collected death certificates.[2]

Definition of cause-specific death rate

The cause-specific death rate is calculated by dividing the annual number of deaths due to a specific cause by the mid-year population for the respective year and is presented per 100,000 individuals.

$$(Cause-specific\ death\ rate) = \frac{(The\ number\ of\ death\ due\ to\ the\ specific\ cause)}{(Population\ of\ the\ middle\ of\ year)} \times 100{,}000$$

Population of the middle of the year: population on July 1 in the year.

Reference:

1. Statistics Korea (South Korea). Causes of Death Statistics in 2019
2. Koo HJ, Lee TY. The primary factors of uncertain cause of death between countries and the statistical influence of postmortem investigation system. Korean Criminological Review. 2011;22(1):173-99.
3. Vos T, Lim SS, Abbafati C, Abbas KM, Abbasi M, Abbasifard M, Abbasi-Kangevari M, Abbastabar H, Abd-Allah F, Abdelalim A, Abdollahi M. Global burden of 369 diseases and injuries in 204 countries and territories, 1990–2019: a systematic analysis for the Global Burden of Disease Study 2019. The Lancet. 2020 Oct 17;396(10258):1204-22.

Appendix method 2. Summary of redistribution process for cause-of-death modeling in GBD 2019 study[1,2]

Input data were obtained from various sources, including vital registration, censuses, household surveys, civil registration and others. The Cause of Death Ensemble model (CODEm) was employed to calculate cause-specific death rates and cause fractions.

By contrast, before being put into the CODEm model, GBD data undergo several processing and correction steps. One important step is garbage code redistribution. A "garbage code" refers to any recorded cause of death on a death certificate that cannot officially result in death. In certain cases, death certificates indicate an "intermediate" cause of death, such as heart failure, which represents a modality of dying rather than an etiological factor leading to death. The GBD approach assesses the percentage of deaths with garbage coding by country and year. The garbage codes are reassigned to the most likely underlying cause of death based on a variety sources, including published literature, expert opinions, guidelines from the International Classification of Diseases (ICD), and disease-specific knowledge (the figure below illustrates this process). This reassignment process has a significant impact on the reported causes of death. More information on this process can be found here: https://www.healthdata.org/acting-data/determining-causes-death-how-we-reclassify-miscoded-deaths

References:

1. Vos T, Lim SS, Abbafati C, Abbas KM, Abbasi M, Abbasifard M, Abbasi-Kangevari M, Abbastabar H, Abd-Allah F, Abdelalim A, Abdollahi M. Global burden of 369 diseases and injuries in 204 countries and territories, 1990–2019: a systematic analysis for the Global Burden of Disease Study 2019. The Lancet. 2020 Oct 17;396(10258):1204-22.

2. Naghavi M, Abajobir AA, Abbafati C, Abbas KM, Abd-Allah F, Abera SF, Aboyans V, Adetokunboh O, Afshin A, Agrawal A, Ahmadi A. Global, regional, and national age-sex specific mortality for 264 causes of death, 1980–2016: a systematic analysis for the Global Burden of Disease Study 2016. The lancet. 2017 Sep 16;390(10100):1151-210.

Appendix method 3. Plausible reasons behind the discrepancies between GBD estimate and national report

To validate how well does the GBD estimates line up with estimates from the national report at the same timepoint, we compared the 10 leading causes of death in this report to that of national report (Causes of Death Statistics in 2019). We observed non-trivial differences between the death estimates reported in GBD 2019 versus the national report; these differences are reported in Appendix table 12.

The discrepancies observed between the estimates from GBD and Statistics Korea could be attributed to several factors. First, the definitions of diseases differ between the national report and GBD 2019. Compared to the national report, the GBD study employed a more extensive range of ICD codes, including ICD-9, for mapping each disease (Appendix table 13). Consequently, estimates could differ between the two databases due to the lack of precise alignment between ICD codes for each disease category. Second, differences exist in the processing of raw input data. GBD estimates are generated through a complex modeling process, which aims to provide a comprehensive and consistent assessment of health data across countries and time periods. The GBD approach involves various stages for estimating causes of death, including input standardization, mapping, age-sex splitting, correcting age-sex violations, garbage code redistribution, noise reduction, and adjustment for local-level covariates (a detailed description of the GBD methodology for causes of disease can be found in the capstone paper1 and Appendix method 2). In contrast, the methodologies used to generate the national estimates did not utilize such extensive processing and correction steps (a detailed description of the methodologies used in the "Causes of Death Statistics in 2019, South Korea" report can be found in Appendix method 1). As such, a national report on the cause of death has been influenced by a high proportion of unknown cause of death in input data, as mentioned in Appendix method 1 (how GBD tried to minimize the influence of this large proportion of unknown cause of death is described in Appendix method 2)

Determining the superiority of one estimate over another is challenging—and even pointless. We suggest using both estimates from GBD and national reports complementarily. Given that GBD estimated the burden of disease employing consistent approaches distinct from national reports, it may function as a valuable supplementary reference for South Korea governments and health authorities. The consistent modeling inherent in GBD results facilitates comparisons of health outcomes with other countries and places the disease burden in South Korea within a broader context. Integrating GBD results on top of national estimates would encourage a more robust understanding of the national healthcare performance.

Distinct features of GBD compared to national report is that consistent modeling inherent across GBD results facilitates comparisons of health outcomes with other countries and places the disease burden in South Korea within a broader context. Also, GBD provides health measures—i.e., YLL, YLD, DALY—that are not reported in national statistics; this GBD study incorporated more than 500 input data sources (listed in the Appendix table 2) to estimate diverse measures (prevalence, YLL, YLD, DALY, etc) across > 300 disease burdens.

References:

1. Vos T, Lim SS, Abbafati C, Abbas KM, Abbasi M, Abbasifard M, Abbasi-Kangevari M, Abbastabar H, Abd-Allah F, Abdelalim A, Abdollahi M. Global burden of 369 diseases and injuries in 204 countries and territories, 1990–2019: a systematic analysis for the Global Burden of Disease Study 2019. The Lancet. 2020 Oct 17;396(10258):1204-22.

2. Koo HJ, Lee TY. The primary factors of uncertain cause of death between countries and the statistical influence of postmortem investigation system. Korean Criminological Review. 2011;22(1):173-99.

Author contribution

Providing data or critical feedback on data sources

Dong-Woo Choi, Sung Hwi Hong, Mehdi Hosseinzadeh, Cho-il Kim, Gyu Ri Kim, Jihee Kim, Min Seo Kim, Sang-woong Lee, Seung Won Lee, Yo Han Lee, Ali H Mokdad, Christopher J L Murray, Akinkunmi Paul Okekunle, Seoyeon Park, Navid Rabiee, Jae Il Shin, Youn Ho Shin, and Dong Keon Yon.

Developing methods or computational machinery

Mehdi Hosseinzadeh, Sang-woong Lee, Seung Won Lee, Ali H Mokdad, Christopher J L Murray, Seoyeon Park, Jae Il Shin, Youn Ho Shin, and Dong Keon Yon.

Providing critical feedback on methods or results

Suneth Buddhika Agampodi, Dong-Woo Choi, Simon I Hay, Sung Hwi Hong, Mehdi Hosseinzadeh, Louis Jacob, Cho-il Kim, Gyu Ri Kim, Jihee Kim, Kwanghyun Kim, Min Seo Kim, Sungroul Kim, Ai Koyanagi, Doo Woong Lee, Hankil Lee, Sang-woong Lee, Seung Won Lee, Yo Han Lee, Susan A McLaughlin, Ali H Mokdad, Christopher J L Murray, Akinkunmi Paul Okekunle, Eun-Cheol Park, Seoyeon Park, Navid Rabiee, Jae Il Shin, Youn Ho Shin, Lee Smith, Joseph L Ward, and Dong Keon Yon.

Drafting the work or revising it critically for important intellectual content

Suneth Buddhika Agampodi, Maryam Beiranvand, Simon I Hay, Sung Hwi Hong, Louis Jacob, Jihee Kim, Kwanghyun Kim, Min Seo Kim, Sungroul Kim, Ai Koyanagi, Doo Woong Lee, Seung Won Lee, Susan A McLaughlin, Max L Mehlman, Ali H Mokdad, Christopher J L Murray, Seoyeon Park, Jae Il Shin, Youn Ho Shin, Lee Smith, Joseph L Ward, and Dong Keon Yon.

Managing the estimation or publications process

Simon I Hay, Min Seo Kim, Seung Won Lee, Susan A McLaughlin, Ali H Mokdad, Christopher J L Murray, Seoyeon Park, Jae Il Shin, and Dong Keon Yon.

*Seoyeon Park, Min Seo Kim, Dong Keon Yon, Seung Won Lee, Joseph Ward, Susan A McLaughlin contributed equally to this work
